# Supplementary material for: MARLOWE: taxonomic characterization of unknown samples for forensics using de novo peptide identification
Source: Sci Rep. 2026 Aug 20;16:26181. doi: 10.1038/s41598-026-50102-3 (PMC13494011; doi:10.1038/s41598-026-50102-3)
Supplement: Supplementary file 1 [file 41598_2026_50102_MOESM1_ESM.docx]

Supporting Information:

**MARLOWE – Taxonomic Characterization of Unknown Samples for Forensics Using *De Novo* Peptide Identification**

**Sarah C. Jenson^1†^, Fanny Chu^1†^*, Gelio Alves^2^, Aleksey Y. Ogurtsov^2^, Anthony S. Barente^1,3^, Dustin L. Crockett^4^, Natalie C. Lamar^5^, Eric D. Merkley^1^, Yi-Kuo Yu^2^, Kristin H. Jarman^1‡^**

**Affiliations:**

^1^Chemical & Biological Signatures Group, Pacific Northwest National Laboratory, Richland, WA 99354

^2^Division of Intramural Research, National Library of Medicine, National Institutes of Health, Bethesda, MD 20854

^3^Department of Genome Sciences, University of Washington, Seattle, WA 98194

^4^Applied Decisions Systems and Analytics, Group, Pacific Northwest National Laboratory, Richland, WA 99354

^5^Applied Statistics and Computational Modeling Group, Pacific Northwest National Laboratory, Richland, WA 99354

^†^These authors contributed equally to this work.

***Corresponding author:**

Fanny Chu

fanny.chu@pnnl.gov

509.372.4819

**^‡^Current affiliation:**

Kristin H. Jarman

Karius Inc.

975 Island Dr., Suite 101

Redwood City, CA, 94065

**Table S1. ProteomeXchange Datasets Used in this Study**

| **ProteomeXchange Identifier** | **Reference** | **Organisms** |
| --- | --- | --- |
| PXD001860 | Payne, S. H. et al. The Pacific Northwest National Laboratory library of bacterial and archaeal proteomic biodiversity. *Scientific Data* 2, 150041, doi:10.1038/sdata.2015.41 (2015). | 109 bacterial organisms |
| PXD003669 | Pfrunder, S. et al. Bacillus cereus Group-Type Strain-Specific Diagnostic Peptides. *J. Proteome Res.* 15, 3098-3107, doi:10.1021/acs.jproteome.6b00216 (2016). | *Bacillus cereus* group members |
| PXD014522 | Karlsson, R. et al. Discovery of Species-unique Peptide Biomarkers of Bacterial Pathogens by Tandem Mass Spectrometry-based Proteotyping. *Mol. Cell. Proteomics* 19, 518-528, doi:https://doi.org/10.1074/mcp.RA119.001667 (2020). | *Haemophilus influenzae, Moraxella catarrhalis, Staphylococcus aureus, Streptococcus pneumoniae* (strain ATCC BAA-255 / R6) |
| PXD023033 | Kondori, N. et al. Mass Spectrometry Proteotyping-Based Detection and Identification of Staphylococcus aureus, Escherichia coli, and Candida albicans in Blood. *Frontiers in Cellular and Infection Microbiology* 11, doi:10.3389/fcimb.2021.634215 (2021). | *Candida albicans, Escherichia coli, Staphylococcus aureus* |

Table S2. Datafiles from ProteomeXchange Datasets Used in this Study

| **ProteomeXchange Identifier** | **Filename** | **Species Name** | **Strain Names** | **Cysteine Carbamylated?** | **Used in ROC Analysis?** |
| --- | --- | --- | --- | --- | --- |
| PXD014522 | Hi_QEHF_171201_21.raw | Haemophilus influenzae | NA | No | Yes |
| PXD014522 | Hi_QE_170112_37.raw | Haemophilus influenzae | NA | No | Yes |
| PXD014522 | Hi_QE_170113_37.raw | Haemophilus influenzae | NA | No | Yes |
| PXD014522 | Hi_QE_170113_38.raw | Haemophilus influenzae | NA | No | Yes |
| PXD014522 | Hi_QE_170524_80.raw | Haemophilus influenzae | NA | No | Yes |
| PXD014522 | Hi_QE_171019_07.raw | Haemophilus influenzae | NA | No | Yes |
| PXD014522 | Hi_QE_171019_34.raw | Haemophilus influenzae | NA | No | Yes |
| PXD014522 | Hi_QE_171019_57.raw | Haemophilus influenzae | NA | No | Yes |
| PXD014522 | Hi_QE_171110_44.raw | Haemophilus influenzae | NA | No | Yes |
| PXD014522 | Hi_QE_171110_65.raw | Haemophilus influenzae | NA | No | Yes |
| PXD014522 | Hi_QE_171110_71.raw | Haemophilus influenzae | NA | No | Yes |
| PXD014522 | Hi_QE_171110_77.raw | Haemophilus influenzae | NA | No | Yes |
| PXD014522 | Hi_QE_180126_33.raw | Haemophilus influenzae | NA | No | Yes |
| PXD014522 | Mc_QEHF_171201_18.raw | Moraxella catarrhalis | NA | No | Yes |
| PXD014522 | Mc_QEHF_171201_24.raw | Moraxella catarrhalis | NA | No | Yes |
| PXD014522 | Mc_QE_170110_14.raw | Moraxella catarrhalis | NA | No | No |
| PXD014522 | Mc_QE_170110_20.raw | Moraxella catarrhalis | NA | No | No |
| PXD014522 | Mc_QE_171019_11.raw | Moraxella catarrhalis | NA | No | Yes |
| PXD014522 | Mc_QE_171019_61.raw | Moraxella catarrhalis | NA | No | Yes |
| PXD014522 | Mc_QE_171019_85.raw | Moraxella catarrhalis | NA | No | Yes |
| PXD014522 | Mc_QE_171019_86.raw | Moraxella catarrhalis | NA | No | Yes |
| PXD014522 | Sa_QEHF_171201_69.raw | Staphylococcus aureus | NA | No | Yes |
| PXD014522 | Sa_QEHF_171201_81.raw | Staphylococcus aureus | NA | No | No |
| PXD014522 | Sa_QEHF_171201_84.raw | Staphylococcus aureus | NA | No | No |
| PXD014522 | Sa_QEHF_181205_23.raw | Staphylococcus aureus | NA | No | Yes |
| PXD014522 | Sa_QEHF_181205_27.raw | Staphylococcus aureus | NA | No | Yes |
| PXD014522 | Sa_QEHF_181205_29.raw | Staphylococcus aureus | NA | No | Yes |
| PXD014522 | Sa_QEHF_190218_71.raw | Staphylococcus aureus | NA | No | Yes |
| PXD014522 | Sa_QE_170412_11.raw | Staphylococcus aureus | NA | No | Yes |
| PXD014522 | Sa_QE_180126_21.raw | Staphylococcus aureus | NA | No | Yes |
| PXD014522 | Sp_QEHF_171201_27.raw | Streptococcus pneumoniae | NA | No | Yes |
| PXD014522 | Sp_QEHF_181205_49.raw | Streptococcus pneumoniae | NA | No | Yes |
| PXD014522 | Sp_QEHF_190218_83.raw | Streptococcus pneumoniae | NA | No | Yes |
| PXD014522 | Sp_QE_170110_08.raw | Streptococcus pneumoniae | NA | No | Yes |
| PXD014522 | Sp_QE_170110_20.raw | Streptococcus pneumoniae | NA | No | No |
| PXD014522 | Sp_QE_170112_12.raw | Streptococcus pneumoniae | NA | No | Yes |
| PXD014522 | Sp_QE_170112_13.raw | Streptococcus pneumoniae | NA | No | Yes |
| PXD014522 | Sp_QE_170112_21.raw | Streptococcus pneumoniae | NA | No | Yes |
| PXD014522 | Sp_QE_170112_28.raw | Streptococcus pneumoniae | NA | No | No |
| PXD014522 | Sp_QE_171019_23.raw | Streptococcus pneumoniae | NA | No | Yes |
| PXD014522 | Sp_QE_171019_26.raw | Streptococcus pneumoniae | NA | No | Yes |
| PXD014522 | Sp_QE_171019_69.raw | Streptococcus pneumoniae | NA | No | Yes |
| PXD014522 | Sp_QE_171019_70.raw | Streptococcus pneumoniae | NA | No | Yes |
| PXD014522 | Sp_QE_171110_38.raw | Streptococcus pneumoniae | NA | No | Yes |
| PXD023033 | QEHF_190520_37.raw | Staphylococcus aureus | S. aureus (CCUG 41582) | No | Yes |
| PXD023033 | QEHF_190520_38.raw | Staphylococcus aureus | S. aureus (CCUG 41582) | No | Yes |
| PXD023033 | QEHF_190614_42.raw | Staphylococcus aureus | S. aureus (CCUG 41582) | No | Yes |
| PXD023033 | QEHF_190614_43.raw | Staphylococcus aureus | S. aureus (CCUG 41582) | No | Yes |
| PXD023033 | QEHF_190614_47.raw | Staphylococcus aureus | S. aureus (CCUG 41582) | No | Yes |
| PXD023033 | QEHF_190614_48.raw | Staphylococcus aureus | S. aureus (CCUG 41582) | No | Yes |
| PXD023033 | QEHF_190614_49.raw | Staphylococcus aureus | S. aureus (CCUG 41582) | No | Yes |
| PXD023033 | QEHF_190520_41.raw | Staphylococcus aureus | S. aureus (CCUG 41582) | No | Yes |
| PXD023033 | QEHF_190614_54.raw | Staphylococcus aureus | S. aureus (CCUG 41582) | No | Yes |
| PXD023033 | QEHF_190614_55.raw | Staphylococcus aureus | S. aureus (CCUG 41582) | No | Yes |
| PXD023033 | QEHF_190523_08.raw | Escherichia coli | E. coli (CCUG 49263) | No | Yes |
| PXD023033 | QEHF_190614_62.raw | Escherichia coli | E. coli (CCUG 49263) | No | Yes |
| PXD023033 | QEHF_190614_63.raw | Escherichia coli | E. coli (CCUG 49263) | No | Yes |
| PXD023033 | QEHF_190614_64.raw | Escherichia coli | E. coli (CCUG 49263) | No | Yes |
| PXD023033 | QEHF_190614_68.raw | Escherichia coli | E. coli (CCUG 49263) | No | Yes |
| PXD023033 | QEHF_190614_69.raw | Escherichia coli | E. coli (CCUG 49263) | No | Yes |
| PXD023033 | QEHF_190614_70.raw | Escherichia coli | E. coli (CCUG 49263) | No | Yes |
| PXD023033 | QEHF_190614_74.raw | Escherichia coli | E. coli (CCUG 49263) | No | Yes |
| PXD023033 | QEHF_190614_75.raw | Escherichia coli | E. coli (CCUG 49263) | No | Yes |
| PXD023033 | QEHF_190614_76.raw | Escherichia coli | E. coli (CCUG 49263) | No | Yes |
| PXD023033 | QEHF_190703_25.raw | Candida albicans | C. albicans (CCUG 32723) | No | Yes |
| PXD023033 | QEHF_190703_32.raw | Candida albicans | C. albicans (CCUG 32723) | No | Yes |
| PXD023033 | QEHF_190703_34.raw | Candida albicans | C. albicans (CCUG 32723) | No | Yes |
| PXD023033 | QEHF_190624_14.raw | Candida albicans | C. albicans (CCUG 32723) | No | Yes |
| PXD023033 | QEHF_190703_09.raw | Candida albicans | C. albicans (CCUG 32723) | No | Yes |
| PXD023033 | QEHF_190703_17.raw | Candida albicans | C. albicans (CCUG 32723) | No | Yes |
| PXD023033 | QEHF_190703_26.raw | Candida albicans | C. albicans (CCUG 32723) | No | Yes |
| PXD023033 | QEHF_190703_10.raw | Candida albicans | C. albicans (CCUG 32723) | No | Yes |
| PXD023033 | QEHF_190703_18.raw | Candida albicans | C. albicans (CCUG 32723) | No | Yes |
| PXD023033 | QEHF_190703_27.raw | Candida albicans | C. albicans (CCUG 32723) | No | Yes |
| PXD023033 | QEHF_190703_11.raw | Candida albicans | C. albicans (CCUG 32723) | No | Yes |
| PXD023033 | QEHF_190703_19.raw | Candida albicans | C. albicans (CCUG 32723) | No | Yes |
| PXD023033 | QEHF_190703_28.raw | Candida albicans | C. albicans (CCUG 32723) | No | Yes |
| PXD023033 | QEHF_190703_12.raw | Candida albicans | C. albicans (CCUG 32723) | No | Yes |
| PXD023033 | QEHF_190703_20.raw | Candida albicans | C. albicans (CCUG 32723) | No | Yes |
| PXD023033 | QEHF_190703_29.raw | Candida albicans | C. albicans (CCUG 32723) | No | Yes |
| PXD023033 | QEHF_190816_03.raw | Escherichia coli | E. coli (CCUG 49263) | No | Yes |
| PXD023033 | QEHF_190816_04.raw | Escherichia coli | E. coli (CCUG 49263) | No | Yes |
| PXD023033 | QEHF_190816_05.raw | Escherichia coli | E. coli (CCUG 49263) | No | Yes |
| PXD023033 | QEHF_190816_06.raw | Escherichia coli | E. coli (CCUG 49263) | No | Yes |
| PXD023033 | QEHF_190816_07.raw | Escherichia coli | E. coli (CCUG 49263) | No | Yes |
| PXD023033 | QEHF_190816_08.raw | Escherichia coli | E. coli (CCUG 49263) | No | Yes |
| PXD023033 | QEHF_190816_10.raw | Escherichia coli | E. coli (CCUG 49263) | No | Yes |
| PXD023033 | QEHF_181106_06.raw | Escherichia coli | E. coli (CCUG 49263) | No | Yes |
| PXD023033 | QEHF_181106_20.raw | Escherichia coli | E. coli (CCUG 49263) | No | Yes |
| PXD023033 | QEHF_181106_07.raw | Escherichia coli | E. coli (CCUG 49263) | No | Yes |
| PXD023033 | QEHF_181106_28.raw | Escherichia coli | E. coli (CCUG 49263) | No | Yes |
| PXD023033 | QEHF_181106_08.raw | Escherichia coli | E. coli (CCUG 49263) | No | Yes |
| PXD023033 | QEHF_181106_21.raw | Escherichia coli | E. coli (CCUG 49263) | No | Yes |
| PXD023033 | QEHF_181106_09.raw | Escherichia coli | E. coli (CCUG 49263) | No | Yes |
| PXD023033 | QEHF_181106_22.raw | Escherichia coli | E. coli (CCUG 49263) | No | Yes |
| PXD023033 | QEHF_181106_10.raw | Escherichia coli | E. coli (CCUG 49263) | No | Yes |
| PXD023033 | QEHF_181106_23.raw | Escherichia coli | E. coli (CCUG 49263) | No | Yes |
| PXD023033 | QEHF_181106_11.raw | Escherichia coli | E. coli (CCUG 49263) | No | Yes |
| PXD023033 | QEHF_181106_24.raw | Escherichia coli | E. coli (CCUG 49263) | No | Yes |
| PXD023033 | QEHF_181106_12.raw | Escherichia coli | E. coli (CCUG 49263) | No | Yes |
| PXD023033 | QEHF_181106_25.raw | Escherichia coli | E. coli (CCUG 49263) | No | Yes |
| PXD023033 | QEHF_181106_13.raw | Escherichia coli | E. coli (CCUG 49263) | No | Yes |
| PXD023033 | QEHF_181106_26.raw | Escherichia coli | E. coli (CCUG 49263) | No | Yes |
| PXD023033 | Lumos_201104_13.raw | Staphylococcus aureus | S. aureus (CCUG 41582) | No | Yes |
| PXD023033 | Lumos_201104_14.raw | Staphylococcus aureus | S. aureus (CCUG 41582) | No | Yes |
| PXD023033 | Lumos_201104_15.raw | Staphylococcus aureus | S. aureus (CCUG 41582) | No | Yes |
| PXD023033 | Lumos_201104_16.raw | Staphylococcus aureus | S. aureus (CCUG 41582) | No | Yes |
| PXD023033 | Lumos_201104_17.raw | Staphylococcus aureus | S. aureus (CCUG 41582) | No | Yes |
| PXD023033 | Lumos_201104_18.raw | Staphylococcus aureus | S. aureus (CCUG 41582) | No | Yes |
| PXD023033 | Lumos_201104_19.raw | Staphylococcus aureus | S. aureus (CCUG 41582) | No | Yes |
| PXD023033 | Lumos_201104_20.raw | Staphylococcus aureus | S. aureus (CCUG 41582) | No | Yes |
| PXD023033 | Lumos_201104_21.raw | Staphylococcus aureus | S. aureus (CCUG 41582) | No | Yes |
| PXD023033 | Lumos_201104_22.raw | Staphylococcus aureus | S. aureus (CCUG 41582) | No | Yes |
| PXD023033 | Lumos_201104_23.raw | Staphylococcus aureus | S. aureus (CCUG 41582) | No | Yes |
| PXD023033 | Lumos_201104_24.raw | Staphylococcus aureus | S. aureus (CCUG 41582) | No | Yes |
| PXD023033 | Lumos_201104_25.raw | Staphylococcus aureus | S. aureus (CCUG 41582) | No | Yes |
| PXD023033 | Lumos_201104_26.raw | Staphylococcus aureus | S. aureus (CCUG 41582) | No | Yes |
| PXD023033 | Lumos_201104_33.raw | Staphylococcus aureus | S. aureus (CCUG 41582) | No | Yes |
| PXD023033 | Lumos_201104_34.raw | Staphylococcus aureus | S. aureus (CCUG 41582) | No | Yes |
| PXD023033 | Lumos_201104_35.raw | Staphylococcus aureus | S. aureus (CCUG 41582) | No | Yes |
| PXD023033 | Lumos_201104_36.raw | Staphylococcus aureus | S. aureus (CCUG 41582) | No | Yes |
| PXD023033 | Lumos_201104_37.raw | Staphylococcus aureus | S. aureus (CCUG 41582) | No | Yes |
| PXD023033 | Lumos_201104_38.raw | Staphylococcus aureus | S. aureus (CCUG 41582) | No | Yes |
| PXD023033 | Lumos_201104_39.raw | Staphylococcus aureus | S. aureus (CCUG 41582) | No | Yes |
| PXD023033 | Lumos_201104_40.raw | Staphylococcus aureus | S. aureus (CCUG 41582) | No | Yes |
| PXD023033 | Lumos_201104_41.raw | Staphylococcus aureus | S. aureus (CCUG 41582) | No | Yes |
| PXD023033 | Lumos_201104_42.raw | Staphylococcus aureus | S. aureus (CCUG 41582) | No | Yes |
| PXD023033 | Lumos_201104_43.raw | Staphylococcus aureus | S. aureus (CCUG 41582) | No | Yes |
| PXD023033 | Lumos_201104_44.raw | Staphylococcus aureus | S. aureus (CCUG 41582) | No | Yes |
| PXD023033 | Lumos_201104_45.raw | Staphylococcus aureus | S. aureus (CCUG 41582) | No | Yes |
| PXD023033 | Lumos_201104_46.raw | Staphylococcus aureus | S. aureus (CCUG 41582) | No | Yes |
| PXD023033 | QEHF_181122_19.raw | Candida albicans | C. albicans (CCUG 32723) | No | Yes |
| PXD023033 | QEHF_181122_20.raw | Candida albicans | C. albicans (CCUG 32723) | No | Yes |
| PXD023033 | QEHF_181122_21.raw | Candida albicans | C. albicans (CCUG 32723) | No | Yes |
| PXD023033 | QEHF_181122_22.raw | Candida albicans | C. albicans (CCUG 32723) | No | Yes |
| PXD023033 | QEHF_181122_23.raw | Candida albicans | C. albicans (CCUG 32723) | No | Yes |
| PXD023033 | QEHF_181122_24.raw | Candida albicans | C. albicans (CCUG 32723) | No | No |
| PXD023033 | QEHF_181122_25.raw | Candida albicans | C. albicans (CCUG 32723) | No | Yes |
| PXD023033 | QEHF_181122_26.raw | Candida albicans | C. albicans (CCUG 32723) | No | Yes |
| PXD003669 | 20150219sp_6843_B12_a1_1.raw | Bacillus mycoides | Bacillus cereus (DSM 31) | Yes | Yes |
| PXD003669 | 20150219sp_6843_B12_a1_2.raw | Bacillus mycoides | Bacillus cereus (DSM 31) | Yes | Yes |
| PXD003669 | 20150219sp_6843_B12_a2_1.raw | Bacillus mycoides | Bacillus cereus (DSM 31) | Yes | Yes |
| PXD003669 | 20150219sp_6843_B12_a2_2.raw | Bacillus mycoides | Bacillus cereus (DSM 31) | Yes | Yes |
| PXD003669 | 20150219sp_6843_B12_a3_1.raw | Bacillus mycoides | Bacillus cereus (DSM 31) | Yes | Yes |
| PXD003669 | 20150219sp_6843_B12_a3_2.raw | Bacillus mycoides | Bacillus cereus (DSM 31) | Yes | Yes |
| PXD003669 | 20150224sp_6836_B18_a1_1.raw | Bacillus cereus | Bacillus cereus (DSM 31) | Yes | Yes |
| PXD003669 | 20150224sp_6836_B18_a1_2.raw | Bacillus cereus | Bacillus cereus (DSM 31) | Yes | Yes |
| PXD003669 | 20150224sp_6836_B18_a2_1.raw | Bacillus cereus | Bacillus cereus (DSM 31) | Yes | Yes |
| PXD003669 | 20150224sp_6836_B18_a2_2.raw | Bacillus cereus | Bacillus cereus (DSM 31) | Yes | Yes |
| PXD003669 | 20150224sp_6836_B18_a3_1.raw | Bacillus cereus | Bacillus cereus (DSM 31) | Yes | Yes |
| PXD003669 | 20150224sp_6836_B18_a3_2.raw | Bacillus cereus | Bacillus cereus (DSM 31) | Yes | Yes |
| PXD003669 | 20150224sp_6841_B17_a1_1.raw | Bacillus thuringiensis | Bacillus toyonensis (CECT 876) | Yes | Yes |
| PXD003669 | 20150224sp_6841_B17_a1_2.raw | Bacillus thuringiensis | Bacillus toyonensis (CECT 876) | Yes | Yes |
| PXD003669 | 20150224sp_6841_B17_a2_1.raw | Bacillus thuringiensis | Bacillus toyonensis (CECT 876) | Yes | Yes |
| PXD003669 | 20150224sp_6841_B17_a2_2.raw | Bacillus thuringiensis | Bacillus toyonensis (CECT 876) | Yes | Yes |
| PXD003669 | 20150224sp_6841_B17_a3_1.raw | Bacillus thuringiensis | Bacillus toyonensis (CECT 876) | Yes | Yes |
| PXD003669 | 20150224sp_6841_B17_a3_2.raw | Bacillus thuringiensis | Bacillus toyonensis (CECT 876) | Yes | Yes |
| PXD003669 | 20150306sp_7007_B9_a1_1.raw | Bacillus mycoides;Bacillus weihenstephanensis | B. pseudomycoides (DSM 12442) | Yes | Yes |
| PXD003669 | 20150306sp_7007_B9_a1_2.raw | Bacillus mycoides;Bacillus weihenstephanensis | B. pseudomycoides (DSM 12442) | Yes | Yes |
| PXD003669 | 20150306sp_7007_B9_a2_1.raw | Bacillus mycoides;Bacillus weihenstephanensis | B. pseudomycoides (DSM 12442) | Yes | Yes |
| PXD003669 | 20150306sp_7007_B9_a2_2.raw | Bacillus mycoides;Bacillus weihenstephanensis | B. pseudomycoides (DSM 12442) | Yes | Yes |
| PXD003669 | 20150306sp_7007_B9_a3_1.raw | Bacillus mycoides;Bacillus weihenstephanensis | B. pseudomycoides (DSM 12442) | Yes | Yes |
| PXD003669 | 20150306sp_7007_B9_a3_2.raw | Bacillus mycoides;Bacillus weihenstephanensis | B. pseudomycoides (DSM 12442) | Yes | Yes |
| PXD003669 | 20150309sp_7008_B13_a1_1.raw | Bacillus pseudomycoides | Bacillus toyonensis (CECT 876) | Yes | Yes |
| PXD003669 | 20150309sp_7008_B13_a1_2.raw | Bacillus pseudomycoides | Bacillus toyonensis (CECT 876) | Yes | Yes |
| PXD003669 | 20150309sp_7008_B13_a2_1.raw | Bacillus pseudomycoides | Bacillus toyonensis (CECT 876) | Yes | Yes |
| PXD003669 | 20150309sp_7008_B13_a2_2.raw | Bacillus pseudomycoides | Bacillus toyonensis (CECT 876) | Yes | Yes |
| PXD003669 | 20150309sp_7008_B13_a3_1.raw | Bacillus pseudomycoides | Bacillus toyonensis (CECT 876) | Yes | Yes |
| PXD003669 | 20150309sp_7008_B13_a3_2.raw | Bacillus pseudomycoides | Bacillus toyonensis (CECT 876) | Yes | Yes |
| PXD003669 | 20150417sp_7165_B20_a1_1.raw | Bacillus cytotoxicus | Bacillus cereus (DSM 31) | Yes | Yes |
| PXD003669 | 20150417sp_7165_B20_a1_2.raw | Bacillus cytotoxicus | Bacillus cereus (DSM 31) | Yes | Yes |
| PXD003669 | 20150417sp_7165_B20_a2_1.raw | Bacillus cytotoxicus | Bacillus cereus (DSM 31) | Yes | Yes |
| PXD003669 | 20150417sp_7165_B20_a2_2.raw | Bacillus cytotoxicus | Bacillus cereus (DSM 31) | Yes | Yes |
| PXD003669 | 20150417sp_7165_B20_a3_1.raw | Bacillus cytotoxicus | Bacillus cereus (DSM 31) | Yes | Yes |
| PXD003669 | 20150417sp_7165_B20_a3_2.raw | Bacillus cytotoxicus | Bacillus cereus (DSM 31) | Yes | Yes |
| PXD003669 | 20150507sp_7221_B21a1_1.raw | Bacillus toyonensis | B. weihenstephanensis (DSM 11821) | Yes | Yes |
| PXD003669 | 20150507sp_7221_B21a1_2.raw | Bacillus toyonensis | B. weihenstephanensis (DSM 11821) | Yes | Yes |
| PXD003669 | 20150507sp_7221_B21a2_1.raw | Bacillus toyonensis | B. weihenstephanensis (DSM 11821) | Yes | Yes |
| PXD003669 | 20150507sp_7221_B21a2_2.raw | Bacillus toyonensis | B. weihenstephanensis (DSM 11821) | Yes | Yes |
| PXD003669 | 20150507sp_7221_B21a3_1.raw | Bacillus toyonensis | B. weihenstephanensis (DSM 11821) | Yes | Yes |
| PXD003669 | 20150507sp_7221_B21a3_2.raw | Bacillus toyonensis | B. weihenstephanensis (DSM 11821) | Yes | No |
| PXD001860 | Biodiversity_A_cryptum_FeTSB_anaerobic_2_01Jun16_Pippin_16-03-39.raw | Acidiphilium cryptum | Acidiphilium cryptum JF-5 | No | No |
| PXD001860 | Biodiversity_A_muciniphila_test_27Feb17_Pippin_16-11-03.raw | Akkermansia muciniphila | Akkermansia muciniphila ATCC BAA-835 | No | Yes |
| PXD001860 | Biodiversity_A_tumefaciens_R2A_aerobic_2_23Nov16_Pippin_16-09-11.raw | Agrobacterium tumefaciens | Agrobacterium tumefaciens | No | No |
| PXD001860 | Biodiversity_A_tumefaciens_R2A_aerobic_3_23Nov16_Pippin_16-09-11.raw | Agrobacterium tumefaciens | Agrobacterium tumefaciens | No | No |
| PXD001860 | Biodiversity_A_faecalis_LB_aerobic_02_26Feb16_Arwen_16-01-01.raw | Alcaligenes faecalis | Alcaligenes faecalis | No | No |
| PXD001860 | Biodiversity_A_faecalis_LB_aerobic_01_26Feb16_Arwen_16-01-01.raw | Alcaligenes faecalis | Alcaligenes faecalis | No | No |
| PXD001860 | Biodiversity_B_cereus_PN_L_CL_3_09Oct16_Pippin_16-05-06.raw | Bacillus cereus | Bacillus cereus ATCC 14579 | No | Yes |
| PXD001860 | Biodiversity_B_cereus_PN_Z_CL_3_rr_11Oct16_Pippin_16-05-06.raw | Bacillus cereus | Bacillus cereus ATCC 14579 | No | Yes |
| PXD001860 | Biodiversity_Bacillus_subtilis_LB_02_27Dec15_Arwen_15-07-13.raw | Bacillus subtilis | Bacillus subtilis subsp. subtilis str. 168 | No | Yes |
| PXD001860 | Biodiversity_B_subtilis_NCIB3610_plates_1_03May16_Samwise_16-03-32.raw | Bacillus subtilis | Bacillus subtilis subsp. subtilis NCIB 3610 = ATCC 6051 | No | Yes |
| PXD001860 | Biodiversity_B_fragilis_Carb_01_28Oct15_Arwen_15-07-13.raw | Bacteroides fragilis | Bacteroides fragilis 638R | No | No |
| PXD001860 | Biodiversity_B_fragilis_CMgluc_anaerobic_03_01Feb16_Arwen_15-07-13.raw | Bacteroides fragilis | Bacteroides fragilis 638R | No | Yes |
| PXD001860 | Biodiversity_B_thet_CMgluc_anaerobic_03_01Feb16_Arwen_15-07-13.raw | Bacteroides thetaiotaomicron | Bacteroides thetaiotaomicron VPI-5482 | No | No |
| PXD001860 | Biodiversity_B_bifidum_CMcarb_anaerobic_01_26Feb16_Arwen_16-01-01.raw | Bifidobacterium bifidum | Bifidobacterium bifidum ATCC 29521 = JCM 1255 = DSM 20456 | No | Yes |
| PXD001860 | Biodiversity_B_bifidum_CMcarb_anaerobic_03_26Feb16_Arwen_16-01-01.raw | Bifidobacterium bifidum | Bifidobacterium bifidum ATCC 29521 = JCM 1255 = DSM 20456 | No | Yes |
| PXD001860 | Biodiversity_B_infantis_CMcarb_anaerobic_03_26Feb16_Arwen_16-01-01.raw | Bifidobacterium longum | Bifidobacterium longum subsp. infantis ATCC 15697 = JCM 1222 = DSM 20088 | No | Yes |
| PXD001860 | Biodiversity_Cellulomonas_gilvus_GS2_02_27Dec15_Arwen_15-07-13.raw | Cellulomonas gilvus | Cellulomonas gilvus ATCC 13127 | No | Yes |
| PXD001860 | Biodiversity_Cellulomonas_gilvus_GS2_03_27Dec15_Arwen_15-07-13.raw | Cellulomonas gilvus | Cellulomonas gilvus ATCC 13127 | No | Yes |
| PXD001860 | Biodiversity_C_Baltica_T240_R1_C_27Jan16_Arwen_15-07-13.raw | Cellulophaga baltica | Cellulophaga baltica 18 | No | Yes |
| PXD001860 | Biodiversity_C_Baltica_T240_R3_Inf_27Jan16_Arwen_15-07-13.raw | Cellulophaga baltica | Cellulophaga baltica 18 | No | Yes |
| PXD001860 | Biodiversity_C_indologenes_LIB_aerobic_01_03May16_Samwise_16-03-32.raw | Chryseobacterium indologenes | Chryseobacterium indologenes | No | No |
| PXD001860 | Biodiversity_C_indologenes_LIB_aerobic_03_03May16_Samwise_16-03-32.raw | Chryseobacterium indologenes | Chryseobacterium indologenes | No | No |
| PXD001860 | Biodiversity_C_freundii_LIB_01_28Oct15_Arwen_15-07-13.raw | Citrobacter freundii | Citrobacter freundii | No | No |
| PXD001860 | Biodiversity_Cibrobacter_freundii_LB_aerobic_03_01Feb16_Arwen_15-07-13.raw | Citrobacter freundii | Citrobacter freundii | No | No |
| PXD001860 | Biodiversity_C_ljungdahlii_Fructose_anaerobic_1_04Oct16_Pippin_16-05-06.raw | Clostridium ljungdahlii | Clostridium ljungdahlii DSM 13528 | No | Yes |
| PXD001860 | Biodiversity_C_ljungdahlii_Fructose_anaerobic_2_04Oct16_Pippin_16-05-06.raw | Clostridium ljungdahlii | Clostridium ljungdahlii DSM 13528 | No | Yes |
| PXD001860 | Biodiversity_C_necator_R2A_aerobic_2_23Nov16_Pippin_16-09-11.raw | Cupriavidus necator | Cupriavidus necator N-1 | No | Yes |
| PXD001860 | Biodiversity_C_necator_R2A_aerobic_1_23Nov16_Pippin_16-09-11.raw | Cupriavidus necator | Cupriavidus necator N-1 | No | Yes |
| PXD001860 | Biodiversity_HL69_HLA_aerobic_2_05Oct16_Pippin_16-05-06.raw | Cyanobacterium sp. HL-69 | Cyanobacterium sp. HL-69 | No | Yes |
| PXD001860 | Biodiversity_HL69_HLA_aerobic_1_05Oct16_Pippin_16-05-06.raw | Cyanobacterium sp. HL-69 | Cyanobacterium sp. HL-69 | No | Yes |
| PXD001860 | Biodiversity_D_acidovorans_TGY_aerobic_02_29Apr16_Samwise_16-03-32_renamed.raw | Delftia acidovorans | Delftia acidovorans SPH-1 | No | Yes |
| PXD001860 | Biodiversity_D_acidovorans_TGY_aerobic_03_29Apr16_Samwise_16-03-32_renamed.raw | Delftia acidovorans | Delftia acidovorans SPH-1 | No | Yes |
| PXD001860 | Biodiversity_Ecoli_PmrA_3d_TB7_4c_QEP_15Aug18_Wally_18-07-04.raw | Escherichia coli | Escherichia coli K-12 | No | Yes |
| PXD001860 | Biodiversity_Ecoli_PmrA_24h_TB7gluc_1b_QEP_15Aug18_Wally_18-07-04.raw | Escherichia coli | Escherichia coli K-12 | No | Yes |
| PXD001860 | Biodiversity_F_prausnitzii_Carb_01_28Oct15_Arwen_15-07-13.raw | Faecalibacterium prausnitzii | Faecalibacterium prausnitzii | No | Yes |
| PXD001860 | Biodiversity_F_prausnitzii_LIB_01_28Oct15_Arwen_15-07-13.raw | Faecalibacterium prausnitzii | Faecalibacterium prausnitzii | No | No |
| PXD001860 | Biodiversity_F_succinogenes_MDM_03_27Dec15_Arwen_15-07-13.raw | Fibrobacter succinogenes | Fibrobacter succinogenes subsp. succinogenes S85 | No | Yes |
| PXD001860 | Biodiversity_F_succinogenes_MDM_02_27Dec15_Arwen_15-07-13.raw | Fibrobacter succinogenes | Fibrobacter succinogenes subsp. succinogenes S85 | No | Yes |
| PXD001860 | Biodiversity_F_novicida_TSB_aerobic_03_01Feb16_Arwen_15-07-13.raw | Francisella tularensis | Francisella tularensis subsp. novicida U112 | No | Yes |
| PXD001860 | Biodiversity_F_novicida_TSB_aerobic_01_01Feb16_Arwen_15-07-13.raw | Francisella tularensis | Francisella tularensis subsp. novicida U112 | No | Yes |
| PXD001860 | Biodiversity_Lactobacillus_casei_MRS_02_27Dec15_Arwen_15-07-13.raw | Lactobacillus casei | Lactobacillus casei | No | Yes |
| PXD001860 | Biodiversity_Lactobacillus_casei_MRS_03_27Dec15_Arwen_15-07-13.raw | Lactobacillus casei | Lactobacillus casei | No | Yes |
| PXD001860 | Biodiversity_L_monocytogenes_BHI_aerobic_01_27Feb17_Pippin_16-11-03.raw | Listeria monocytogenes | Listeria monocytogenes 10403S | No | Yes |
| PXD001860 | Biodiversity_L_monocytogenes_BHI_aerobic_03_27Feb17_Pippin_16-11-03.raw | Listeria monocytogenes | Listeria monocytogenes 10403S | No | Yes |
| PXD001860 | Biodiversity_M_luteus_LIB_aerobic_03_26Feb16_Arwen_16-01-01.raw | Micrococcus luteus | Micrococcus luteus | No | Yes |
| PXD001860 | Biodiversity_M_luteus_LIB_aerobic_01_26Feb16_Arwen_16-01-01.raw | Micrococcus luteus | Micrococcus luteus | No | Yes |
| PXD001860 | Biodiversity_M_smegmatis_BHI_aerobic_2_05Oct16_Pippin_16-05-06.raw | Mycolicibacterium smegmatis | Mycolicibacterium smegmatis | No | Yes |
| PXD001860 | Biodiversity_M_smegmatis_BHI_aerobic_1_05Oct16_Pippin_16-05-06.raw | Mycolicibacterium smegmatis | Mycolicibacterium smegmatis | No | Yes |
| PXD001860 | Biodiversity_M_xanthus_DZ2_48h_plates_2_13Jun16_Pippin_16-03-39.raw | Myxococcus xanthus | Myxococcus xanthus DZ2 | No | Yes |
| PXD001860 | Biodiversity_M_xanthus_DZ2_24h_plates_2_13Jun16_Pippin_16-03-39.raw | Myxococcus xanthus | Myxococcus xanthus DZ2 | No | Yes |
| PXD001860 | Biodiversity_P_polymyxa_TBS_aerobic_2_17July16_Samwise_16-04-10.raw | Paenibacillus polymyxa | Paenibacillus polymyxa ATCC 842 | No | Yes |
| PXD001860 | Biodiversity_P_polymyxa_TBS_aerobic_3_17July16_Samwise_16-04-10.raw | Paenibacillus polymyxa | Paenibacillus polymyxa ATCC 842 | No | Yes |
| PXD001860 | Biodiversity_P_denitrificans_LIB_aerobic_02_29Apr16_Samwise_16-03-32_renamed.raw | Paracoccus denitrificans | Paracoccus denitrificans | No | Yes |
| PXD001860 | Biodiversity_P_denitrificans_LIB_aerobic_03_29Apr16_Samwise_16-03-32_renamed.raw | Paracoccus denitrificans | Paracoccus denitrificans | No | Yes |
| PXD001860 | Biodiversity_P_ruminicola_MDM_anaerobic_2_09Jun16_Pippin_16-03-39.raw | Prevotella ruminicola | Prevotella ruminicola 23 | No | No |
| PXD001860 | Biodiversity_P_ruminicola_MDM_anaerobic_1_09Jun16_Pippin_16-03-39.raw | Prevotella ruminicola | Prevotella ruminicola 23 | No | Yes |
| PXD001860 | Biodiversity_R_jostii_R2A_aerobic_1_23Nov16_Pippin_16-09-11.raw | Rhodococcus jostii | Rhodococcus jostii RHA1 | No | Yes |
| PXD001860 | Biodiversity_R_jostii_R2A_aerobic_2_23Nov16_Pippin_16-09-11.raw | Rhodococcus jostii | Rhodococcus jostii RHA1 | No | Yes |
| PXD001860 | Biodiversity_R_palustris_PMnitro_anaerobic_2_01Jun16_Pippin_16-03-39.raw | Rhodopseudomonas palustris | Rhodopseudomonas palustris | No | Yes |
| PXD001860 | Biodiversity_R_palustris_PMnonnitro_anaerobic_3_01Jun16_Pippin_16-03-39.raw | Rhodopseudomonas palustris | Rhodopseudomonas palustris | No | Yes |
| PXD001860 | Biodiversity_S_aurantiaca_CYE_aerobic_3_17July16_Samwise_16-04-10.raw | Stigmatella aurantiaca | Stigmatella aurantiaca DW4/3-1 | No | Yes |
| PXD001860 | Biodiversity_S_aurantiaca_CYE_aerobic_2_17July16_Samwise_16-04-10.raw | Stigmatella aurantiaca | Stigmatella aurantiaca DW4/3-1 | No | Yes |
| PXD001860 | Biodiversity_S_agalactiae_LIB_aerobic_03_26Feb16_Arwen_16-01-01.raw | Streptococcus agalactiae | Streptococcus agalactiae | No | Yes |
| PXD001860 | Biodiversity_S_agalactiae_LIB_aerobic_01_26Feb16_Arwen_16-01-01.raw | Streptococcus agalactiae | Streptococcus agalactiae | No | Yes |
| PXD001860 | Biodiversity_S_elongatus_BG11_aerobic_3_14July16_Pippin_16-05-01.raw | Synechococcus elongatus | Synechococcus elongatus PCC 7942 = FACHB-805 | No | Yes |
| PXD001860 | Biodiversity_S_elongatus_BG11NaCl_aerobic_3_05Oct16_Pippin_16-05-06.raw | Synechococcus elongatus | Synechococcus elongatus PCC 7942 = FACHB-805 | No | Yes |

Table S3. Organisms included in the MARLOWE database used in this study. MiCId used an identical database. Total number of organisms in the database - 5851, species – 4023 (taxon identifiers as of July 2019).

| **NCBI Taxon**  **Identifier** | **Organism Name** |
| --- | --- |
| 1257118 | Acanthamoeba castellanii str Neff T02667 |
| 133434 | Acanthaster planci T05078 |
| 329726 | Acaryochloris marina MBIC11017 T00617 |
| 1511 | Acetoanaerobium sticklandii DSM 519 T01334 |
| 435 | Acetobacter aceti T04735 |
| 481146 | Acetobacter ascendens T05523 |
| 1633874 | Acetobacter oryzifermentans T04478 |
| 1266844 | Acetobacter pasteurianus 386B T02786 |
| 634458 | Acetobacter pasteurianus IFO 3283 01 42C T01818 |
| 634452 | Acetobacter pasteurianus IFO 3283 01 T00988 |
| 634453 | Acetobacter pasteurianus IFO 3283 03 T01812 |
| 634454 | Acetobacter pasteurianus IFO 3283 07 T01813 |
| 634459 | Acetobacter pasteurianus IFO 3283 12 T01814 |
| 634455 | Acetobacter pasteurianus IFO 3283 22 T01815 |
| 634456 | Acetobacter pasteurianus IFO 3283 26 T01816 |
| 634457 | Acetobacter pasteurianus IFO 3283 32 T01817 |
| 1076596 | Acetobacter persici T04751 |
| 65959 | Acetobacter pomorum T05180 |
| 446692 | Acetobacter senegalensis T04217 |
| 2259883 | Acetobacter sp JWB T05544 |
| 104102 | Acetobacter tropicalis T05181 |
| 931626 | Acetobacterium woodii DSM 1030 T01732 |
| 574087 | Acetohalobium arabaticum DSM 5501 T01290 |
| 891968 | Acetomicrobium mobile DSM 13181 T02151 |
| 61635 | Acholeplasma brassicae T03095 |
| 441768 | Acholeplasma laidlawii PG 8A T00659 |
| 35623 | Acholeplasma oculi T03695 |
| 1318466 | Acholeplasma palmae J233 T03096 |
| 32002 | Achromobacter denitrificans T04244 |
| 217204 | Achromobacter insolitus T04671 |
| 1758194 | Achromobacter sp AONIH1 T05288 |
| 2282475 | Achromobacter sp B7 T05703 |
| 217203 | Achromobacter spanius T05274 |
| 762376 | Achromobacter xylosoxidans A8 T01341 |
| 1216976 | Achromobacter xylosoxidans NBRC 15126 ATCC 27061 T02964 |
| 1167634 | Achromobacter xylosoxidans NH44784 1996 T02785 |
| 85698 | Achromobacter xylosoxidans T04522 |
| 591001 | Acidaminococcus fermentans DSM 20731 T01151 |
| 568816 | Acidaminococcus intestini RyC MR95 T01639 |
| 41673 | Acidianus brierleyi T05472 |
| 933801 | Acidianus hospitalis W1 T01493 |
| 282676 | Acidianus manzaensis T04836 |
| 619593 | Acidianus sulfidivorans JP7 T05487 |
| 1281578 | Acidiferrobacter sp SPIII 3 T05469 |
| 160660 | Acidihalobacter prosperus T04495 |
| 666510 | Acidilobus saccharovorans 345 15 T01283 |
| 1577685 | Acidilobus sp 7A T05595 |
| 525909 | Acidimicrobium ferrooxidans DSM 10331 T00956 |
| 349163 | Acidiphilium cryptum JF 5 T00527 |
| 926570 | Acidiphilium multivorum AIU301 T01446 |
| 1171373 | Acidipropionibacterium acidipropionici ATCC 4875 T02345 |
| 1748 | Acidipropionibacterium acidipropionici T04523 |
| 1749 | Acidipropionibacterium jensenii T05836 |
| 2057246 | Acidipropionibacterium virtanenii T05538 |
| 2211140 | Acidisarcina polymorpha T05576 |
| 637389 | Acidithiobacillus caldus ATCC 51756 T03561 |
| 990288 | Acidithiobacillus caldus SM 1 T01581 |
| 1232575 | Acidithiobacillus ferridurans T05847 |
| 743299 | Acidithiobacillus ferrivorans SS3 T01595 |
| 243159 | Acidithiobacillus ferrooxidans ATCC 23270 T00810 |
| 380394 | Acidithiobacillus ferrooxidans ATCC 53993 T00758 |
| 240015 | Acidobacterium capsulatum ATCC 51196 T00868 |
| 351607 | Acidothermus cellulolyticus 11B T00426 |
| 643561 | Acidovorax avenae subsp avenae ATCC 19860 T01445 |
| 553814 | Acidovorax carolinensis T04914 |
| 553814 | Acidovorax carolinensis T04962 |
| 553814 | Acidovorax carolinensis T04997 |
| 553814 | Acidovorax carolinensis T04998 |
| 397945 | Acidovorax citrulli AAC00 1 T00451 |
| 535289 | Acidovorax ebreus TPSY T00853 |
| 2478662 | Acidovorax sp 1608163 T05680 |
| 232721 | Acidovorax sp JS42 T00455 |
| 358220 | Acidovorax sp KKS102 T02290 |
| 1842533 | Acidovorax sp RAC01 T04481 |
| 439481 | Aciduliprofundum boonei T469 T01180 |
| 673860 | Aciduliprofundum sp MAR08 339 T02396 |
| 696749 | Acinetobacter baumannii 1656 2 T01819 |
| 480119 | Acinetobacter baumannii AB0057 T00793 |
| 557600 | Acinetobacter baumannii AB307 0294 T00795 |
| 405416 | Acinetobacter baumannii ACICU T00710 |
| 400667 | Acinetobacter baumannii ATCC 17978 T00486 |
| 509173 | Acinetobacter baumannii AYE T00667 |
| 1096995 | Acinetobacter baumannii BJAB07104 T02726 |
| 1096996 | Acinetobacter baumannii BJAB0715 T02727 |
| 1096997 | Acinetobacter baumannii BJAB0868 T02728 |
| 945556 | Acinetobacter baumannii D1279779 T02491 |
| 1455315 | Acinetobacter baumannii LAC 4 T03519 |
| 889738 | Acinetobacter baumannii MDR TJ T02045 |
| 497978 | Acinetobacter baumannii MDR ZJ06 T01820 |
| 509170 | Acinetobacter baumannii SDF T00660 |
| 470 | Acinetobacter baumannii T03374 |
| 470 | Acinetobacter baumannii T03375 |
| 470 | Acinetobacter baumannii T03376 |
| 470 | Acinetobacter baumannii T03377 |
| 980514 | Acinetobacter baumannii TCDC AB0715 T01908 |
| 1100841 | Acinetobacter baumannii TYTH 1 T02261 |
| 1400867 | Acinetobacter baumannii ZW85 1 T02954 |
| 62977 | Acinetobacter baylyi ADP1 T00185 |
| 471 | Acinetobacter calcoaceticus T05452 |
| 1871111 | Acinetobacter defluvii T05474 |
| 1324350 | Acinetobacter equi T04103 |
| 29430 | Acinetobacter haemolyticus T04618 |
| 756892 | Acinetobacter indicus T05342 |
| 1242245 | Acinetobacter johnsonii XBB1 T04178 |
| 40215 | Acinetobacter junii T04674 |
| 1785128 | Acinetobacter lactucae T05220 |
| 1789224 | Acinetobacter larvae T04708 |
| 106654 | Acinetobacter nosocomialis T04196 |
| 436717 | Acinetobacter oleivorans DR1 T01263 |
| 871585 | Acinetobacter pittii PHEA 2 T01684 |
| 40216 | Acinetobacter radioresistens T05620 |
| 108981 | Acinetobacter schindleri T04787 |
| 487316 | Acinetobacter soli T04684 |
| 1809055 | Acinetobacter sp DUT 2 T04353 |
| 1407071 | Acinetobacter sp TGL Y2 T04425 |
| 1646498 | Acinetobacter sp TTH0 4 T04075 |
| 1879050 | Acinetobacter wuhouensis T05823 |
| 32536 | Acinonyx jubatus T04657 |
| 103372 | Acromyrmex echinatior T03917 |
| 70779 | Acropora digitifera T04352 |
| 1612552 | Actinoalloteichus fjordicus T04639 |
| 1470176 | Actinoalloteichus hoggarensis T04975 |
| 340345 | Actinoalloteichus hymeniacidonis T04472 |
| 2072503 | Actinoalloteichus sp AHMU CJ021 T05306 |
| 1612551 | Actinoalloteichus sp GBA129 24 T04619 |
| 202947 | Actinobacillus equuli subsp equuli T03501 |
| 434271 | Actinobacillus pleuropneumoniae serovar 3 str JL03 T00644 |
| 416269 | Actinobacillus pleuropneumoniae serovar 5b str L20 T00476 |
| 537457 | Actinobacillus pleuropneumoniae serovar 7 str AP76 T00717 |
| 189834 | Actinobacillus porcitonsillarum T05443 |
| 339671 | Actinobacillus succinogenes 130Z T00558 |
| 743972 | Actinobacillus suis ATCC 33415 T03246 |
| 696748 | Actinobacillus suis H91 0380 T02262 |
| 1650658 | Actinobacteria bacterium IMCC26256 T03963 |
| 2495645 | Actinobaculum sp 313 T05430 |
| 1960083 | Actinomyces gaoshouyii T04911 |
| 1655 | Actinomyces naeslundii T05566 |
| 544580 | Actinomyces oris T04280 |
| 111015 | Actinomyces radicidentis T04288 |
| 1851395 | Actinomyces sp Chiba101 T05279 |
| 712122 | Actinomyces sp oral taxon 414 T04083 |
| 2081702 | Actinomyces sp oral taxon 897 T05399 |
| 1912795 | Actinomyces tangfeifanii T04541 |
| 1246995 | Actinoplanes friuliensis DSM 7358 T02892 |
| 512565 | Actinoplanes missouriensis 431 T01795 |
| 649831 | Actinoplanes sp N902 109 T02655 |
| 134676 | Actinoplanes sp SE50 110 T02044 |
| 2033844 | Actinoplanes sp SE50 T05392 |
| 414996 | Actinopolyspora erythraea T05020 |
| 446462 | Actinosynnema mirum DSM 43827 T00957 |
| 42197 | Actinosynnema pretiosum T05092 |
| 59505 | Actinotignum schaalii T03214 |
| 7029 | Acyrthosiphon pisum T01076 |
| 1384484 | Adlercreutzia equolifaciens DSM 19450 T02875 |
| 1036672 | Advenella kashmirensis WT001 T02117 |
| 1247726 | Advenella mimigardefordensis DPN7 T03016 |
| 7159 | Aedes aegypti T01053 |
| 169297 | Aegilops tauschii subsp tauschii T04345 |
| 2494375 | Aequorivita sp H23M31 T05794 |
| 746697 | Aequorivita sublithincola DSM 14238 T02132 |
| 33936 | Aeribacillus pallidus T04993 |
| 87541 | Aerococcus christensenii T04262 |
| 119206 | Aerococcus sanguinicola T04253 |
| 2976812 | Aerococcus urinae ACS 120 V Col10a T01447 |
| 1376 | Aerococcus urinae T04528 |
| 51665 | Aerococcus urinaeequi T04249 |
| 128944 | Aerococcus urinaehominis T04284 |
| 1377 | Aerococcus viridans T04270 |
| 2041 | Aeromicrobium erythreum T04236 |
| 2079793 | Aeromicrobium sp 592 T05410 |
| 2107713 | Aeromicrobium sp A1 2 T05642 |
| 648 | Aeromonas caviae T05190 |
| 196024 | Aeromonas dhakensis T05186 |
| 1321367 | Aeromonas hydrophila AL09 71 T03192 |
| 1419584 | Aeromonas hydrophila J 1 T03574 |
| 1288394 | Aeromonas hydrophila ML09 119 T02683 |
| 1418107 | Aeromonas hydrophila pc104A T03193 |
| 380703 | Aeromonas hydrophila subsp hydrophila ATCC 7966 T00424 |
| 644 | Aeromonas hydrophila T03748 |
| 644 | Aeromonas hydrophila T03886 |
| 644 | Aeromonas hydrophila T04629 |
| 1448139 | Aeromonas hydrophila YL17 T03186 |
| 1208104 | Aeromonas media WS T03075 |
| 948519 | Aeromonas rivipollensis T05453 |
| 382245 | Aeromonas salmonicida subsp salmonicida A449 T00500 |
| 645 | Aeromonas salmonicida T04990 |
| 652 | Aeromonas schubertii T04186 |
| 1636608 | Aeromonas sp ASNIH3 T05427 |
| 1758179 | Aeromonas sp ASNIH5 T05873 |
| 2033033 | Aeromonas sp CU5 T05327 |
| 998088 | Aeromonas veronii B565 T01462 |
| 654 | Aeromonas veronii T04529 |
| 1198449 | Aeropyrum camini SY1 JCM 12091 T02858 |
| 272557 | Aeropyrum pernix K1 T00023 |
| 936046 | Agaricus bisporus var bisporus H97 T03140 |
| 597362 | Agaricus bisporus var burnettii JB137 S8 T03136 |
| 754507 | Aggregatibacter actinomycetemcomitans ANH9381 T01651 |
| 668336 | Aggregatibacter actinomycetemcomitans D11S 1 T00993 |
| 694569 | Aggregatibacter actinomycetemcomitans D7S 1 T02046 |
| 272556 | Aggregatibacter actinomycetemcomitans HK1651 T03184 |
| 1407647 | Aggregatibacter actinomycetemcomitans NUM4039 T04531 |
| 714 | Aggregatibacter actinomycetemcomitans T04753 |
| 634176 | Aggregatibacter aphrophilus NJ8700 T00929 |
| 732 | Aggregatibacter aphrophilus T04521 |
| 176299 | Agrobacterium fabrum str C58 T00070 |
| 311403 | Agrobacterium radiobacter K84 T00850 |
| 359 | Agrobacterium rhizogenes T04767 |
| 358 | Agrobacterium sp H13 3 T01432 |
| 1842536 | Agrobacterium sp RAC06 T04501 |
| 358 | Agrobacterium tumefaciens T03816 |
| 358 | Agrobacterium tumefaciens T04213 |
| 311402 | Agrobacterium vitis S4 T00851 |
| 453304 | Agromyces aureus T04413 |
| 2509455 | Agromyces protaetiae T05835 |
| 2080742 | Agromyces sp MF30 A T05411 |
| 2021234 | Ahniella affigens T05483 |
| 9646 | Ailuropoda melanoleuca T01329 |
| 1679444 | Akkermansia glycaniphila T04551 |
| 349741 | Akkermansia muciniphila ATCC BAA 835 T00736 |
| 323284 | Alcaligenes aquatilis T05678 |
| 511 | Alcaligenes faecalis T04157 |
| 511 | Alcaligenes faecalis T05446 |
| 393595 | Alcanivorax borkumensis SK2 T00380 |
| 930169 | Alcanivorax dieselolei B5 T02259 |
| 391936 | Alcanivorax pacificus W11 5 T03580 |
| 1113728 | Alcanivorax sp NBRC 101098 T03879 |
| 1094342 | Alcanivorax xenomutans T04770 |
| 1736674 | Algibacter alginicilyticus T04100 |
| 1727163 | Algoriphagus sanaruensis T04334 |
| 596153 | Alicycliphilus denitrificans BC T01397 |
| 596154 | Alicycliphilus denitrificans K601 T01463 |
| 521098 | Alicyclobacillus acidocaldarius subsp acidocaldarius DSM 446 T00985 |
| 1048834 | Alicyclobacillus acidocaldarius subsp acidocaldarius Tc 4 1 T01821 |
| 312309 | Aliivibrio fischeri ES114 T00236 |
| 388396 | Aliivibrio fischeri MJ11 T00754 |
| 316275 | Aliivibrio salmonicida LFI1238 T00782 |
| 80852 | Aliivibrio wodanis T03808 |
| 679935 | Alistipes finegoldii DSM 17242 T02133 |
| 717959 | Alistipes shahii WAL 8301 T02578 |
| 187272 | Alkalilimnicola ehrlichii MLHE 1 T00391 |
| 293826 | Alkaliphilus metalliredigens QYMF T00551 |
| 350688 | Alkaliphilus oremlandii OhILAs T00607 |
| 889453 | Alkalitalea saponilacus T04906 |
| 8496 | Alligator mississippiensis T03087 |
| 38654 | Alligator sinensis T02922 |
| 1653480 | Alloactinosynnema sp L 07 T04489 |
| 572477 | Allochromatium vinosum DSM 180 T01168 |
| 594679 | Allofrancisella guangzhouensis T03527 |
| 2516557 | Allopseudarcicella aquatilis T05454 |
| 859653 | Alpha proteobacterium HIMB5 T02250 |
| 744985 | Alpha proteobacterium HIMB59 T02249 |
| 1267766 | Altererythrobacter atlanticus T03925 |
| 692370 | Altererythrobacter dongtanensis T04466 |
| 361183 | Altererythrobacter epoxidivorans T04070 |
| 543877 | Altererythrobacter marensis T03951 |
| 645517 | Altererythrobacter namhicola T04447 |
| 2060312 | Altererythrobacter sp B11 T05671 |
| 5599 | Alternaria alternata T05291 |
| 233316 | Alteromonas addita T04403 |
| 589873 | Alteromonas australica T03220 |
| 589873 | Alteromonas australica T03858 |
| 529120 | Alteromonas macleodii ATCC 27126 T02246 |
| 1004787 | Alteromonas macleodii str Balearic Sea AD45 T02253 |
| 1004785 | Alteromonas macleodii str Black Sea 11 T02260 |
| 1004788 | Alteromonas macleodii str English Channel 673 T02254 |
| 1300253 | Alteromonas mediterranea 615 T02719 |
| 1774373 | Alteromonas mediterranea DE T00747 |
| 1004786 | Alteromonas mediterranea DE1 T02340 |
| 1300254 | Alteromonas mediterranea MED64 T02958 |
| 1300255 | Alteromonas mediterranea U4 T02720 |
| 1300256 | Alteromonas mediterranea U7 T02721 |
| 1300257 | Alteromonas mediterranea U8 T02722 |
| 1300259 | Alteromonas mediterranea UM4b T02723 |
| 1300258 | Alteromonas mediterranea UM7 T02724 |
| 715451 | Alteromonas naphthalenivorans T01513 |
| 1777491 | Alteromonas sp Mac1 T05687 |
| 2267264 | Alteromonas sp RKMC 009 T05653 |
| 1160720 | Alteromonas stellipolaris LMG 21856 T04153 |
| 233316 | Alteromonas stellipolaris T04545 |
| 13333 | Amborella trichopoda T02990 |
| 2507160 | Aminipila sp JN 18 T05806 |
| 83263 | Aminobacter aminovorans T04343 |
| 374606 | Aminobacter sp MSH1 T05420 |
| 572547 | Aminobacterium colombiense DSM 12261 T01202 |
| 429009 | Ammonifex degensii KC4 T00994 |
| 698758 | Amphibacillus xylanus NBRC 15112 T02266 |
| 400682 | Amphimedon queenslandica T02284 |
| 1804986 | Amycolatopsis albispora T05547 |
| 208439 | Amycolatopsis japonica T03225 |
| 1221524 | Amycolatopsis mediterranei RB T02804 |
| 713604 | Amycolatopsis mediterranei S699 T01822 |
| 713604 | Amycolatopsis mediterranei S699 T02190 |
| 749927 | Amycolatopsis mediterranei U32 T01269 |
| 1068978 | Amycolatopsis methanolica 239 T03230 |
| 129921 | Amycolatopsis orientalis HCCB10007 T02678 |
| 1896961 | Amycolatopsis sp AA4 T05278 |
| 1911175 | Amycolatopsis sp BJA 103 T05296 |
| 272123 | Anabaena cylindrica PCC 7122 T02435 |
| 46234 | Anabaena sp 90 T02341 |
| 1647413 | Anabaena sp WA102 T04059 |
| 39488 | Anaerobutyricum hallii T05310 |
| 525919 | Anaerococcus prevotii DSM 20548 T00964 |
| 926569 | Anaerolinea thermophila UNI 1 T01410 |
| 1889813 | Anaerolineaceae bacterium oral taxon 439 T04463 |
| 455488 | Anaeromyxobacter dehalogenans 2CP 1 T00833 |
| 290397 | Anaeromyxobacter dehalogenans 2CP C T00326 |
| 404589 | Anaeromyxobacter sp Fw109 5 T00559 |
| 447217 | Anaeromyxobacter sp K T00748 |
| 649756 | Anaerostipes hadrus T02612 |
| 991789 | Anaerotignum propionicum DSM 1682 T04574 |
| 574556 | Anaplasma centrale str Israel T01133 |
| 1412841 | Anaplasma marginale str Dawn T02898 |
| 320483 | Anaplasma marginale str Florida T00855 |
| 1412840 | Anaplasma marginale str Gypsy Plains T02900 |
| 234826 | Anaplasma marginale str St Maries T00216 |
| 1248439 | Anaplasma ovis str Haibei T05659 |
| 1184254 | Anaplasma phagocytophilum str Dog2 T02752 |
| 212042 | Anaplasma phagocytophilum str HZ T00327 |
| 1184253 | Anaplasma phagocytophilum str HZ2 T02753 |
| 1173064 | Anaplasma phagocytophilum str JM T02754 |
| 8839 | Anas platyrhynchos T02816 |
| 1500254 | Aneurinibacillus soli T05174 |
| 1450761 | Aneurinibacillus sp XH2 T04247 |
| 28377 | Anolis carolinensis T01608 |
| 7165 | Anopheles gambiae str PEST T01036 |
| 294699 | Anoxybacillus amylolyticus T04397 |
| 491915 | Anoxybacillus flavithermus WK1 T00790 |
| 198467 | Anoxybacillus gonensis T04000 |
| 1490052 | Anoxybacillus sp B2M1 T04370 |
| 1490057 | Anoxybacillus sp B7M1 T04426 |
| 1323375 | Anoxybacter fermentans T05768 |
| 8845 | Anser cygnoides domesticus T04896 |
| 74033 | Antarctobacter heliothermus T04983 |
| 1277687 | Anthracocystis flocculosa PF 1 T03141 |
| 80765 | Aphis gossypii T05869 |
| 7460 | Apis mellifera T01057 |
| 202946 | Apteryx mantelli mantelli T04298 |
| 1296669 | Aquabacterium olei T05429 |
| 1938604 | Aquaspirillum sp LM1 T04696 |
| 224324 | Aquifex aeolicus VF5 T00013 |
| 1714848 | Aquimarina sp AD1 T05625 |
| 1714849 | Aquimarina sp AD10 T05712 |
| 1714860 | Aquimarina sp BL5 T05615 |
| 332411 | Aquitalea magnusonii T05698 |
| 1590041 | Aquitalea sp USM4 T05865 |
| 81972 | Arabidopsis lyrata subsp lyrata T01578 |
| 3702 | Arabidopsis thaliana T00041 |
| 1850526 | Arachidicoccus sp BS20 T04406 |
| 2341117 | Arachidicoccus sp KIS59 12 T05648 |
| 130453 | Arachis duranensis T04300 |
| 130454 | Arachis ipaensis T04301 |
| 644284 | Arcanobacterium haemolyticum DSM 20595 T01242 |
| 224325 | Archaeoglobus fulgidus DSM 4304 T00011 |
| 1344584 | Archaeoglobus fulgidus DSM 8774 T03231 |
| 572546 | Archaeoglobus profundus DSM 5631 T01152 |
| 387631 | Archaeoglobus sulfaticallidus PM70 1 T02642 |
| 693661 | Archaeoglobus veneficus SNP6 T01451 |
| 1579370 | Archaeon GW2011 AR10 T03835 |
| 1579378 | Archaeon GW2011 AR20 T03843 |
| 48 | Archangium gephyra T03948 |
| 1036172 | Arcobacter butzleri 7h1h T02755 |
| 944546 | Arcobacter butzleri ED 1 T01823 |
| 367737 | Arcobacter butzleri RM4018 T00600 |
| 197482 | Arcobacter halophilus T05822 |
| 1032238 | Arcobacter mytili LMG 24559 T05895 |
| 572480 | Arcobacter nitrofigilis DSM 7299 T01235 |
| 28200 | Arcobacter skirrowii T05743 |
| 944547 | Arcobacter sp L T01834 |
| 1850254 | Arcobacter sp LPB0137 T04722 |
| 1784714 | Arcticibacterium luteifluviistationis T05496 |
| 616991 | Arenibacter algicola T04973 |
| 76114 | Aromatoleum aromaticum EbN1 T00222 |
| 1658671 | Arsenicicoccus sp oral taxon 190 T04014 |
| 235559 | Arsenophonus endosymbiont of Aleurodicus dispersus T05672 |
| 656366 | Arthrobacter alpinus T04095 |
| 656366 | Arthrobacter alpinus T04228 |
| 37928 | Arthrobacter crystallopoietes T05322 |
| 1771959 | Arthrobacter sp ATCC 21022 T04477 |
| 1704044 | Arthrobacter sp ERGS1 01 T04085 |
| 290399 | Arthrobacter sp FB24 T00421 |
| 1588023 | Arthrobacter sp Hiyo8 T04181 |
| 1690248 | Arthrobacter sp LS16 T04078 |
| 1494608 | Arthrobacter sp PAMC 25486 T03487 |
| 2079227 | Arthrobacter sp PGP41 T05450 |
| 1357915 | Arthrobacter sp QXT 31 T05056 |
| 1118963 | Arthrobacter sp Rue61a T02215 |
| 1795 | Arthrobacter sp SLBN 53 T05013 |
| 1849032 | Arthrobacter sp U41 T05053 |
| 1652545 | Arthrobacter sp YC RL1 T04187 |
| 2020486 | Arthrobacter sp YN T05171 |
| 1806905 | Arthrobacter sp ZXY 2 T05201 |
| 696747 | Arthrospira platensis NIES 39 T02608 |
| 1231624 | Asaia bogorensis NBRC 16594 T04138 |
| 4686 | Asparagus officinalis T05243 |
| 344612 | Aspergillus clavatus NRRL 1 T01148 |
| 331117 | Aspergillus fischeri NRRL 181 T01070 |
| 332952 | Aspergillus flavus NRRL3357 T01090 |
| 330879 | Aspergillus fumigatus Af293 T01017 |
| 227321 | Aspergillus nidulans FGSC A4 T01016 |
| 425011 | Aspergillus niger CBS 513 88 T01030 |
| 510516 | Aspergillus oryzae RIB40 T01018 |
| 322098 | Aster yellows witches broom phytoplasma AYWB T00314 |
| 573065 | Asticcacaulis excentricus CB 48 T01378 |
| 7994 | Astyanax mexicanus T05042 |
| 194338 | Athene cunicularia T05777 |
| 565 | Atlantibacter hermannii T05790 |
| 521095 | Atopobium parvulum DSM 20469 T00983 |
| 12957 | Atta cephalotes T04923 |
| 708131 | Aurantimicrobium minutum T05175 |
| 1987356 | Aurantimicrobium sp MWH Mo1 T05480 |
| 2079575 | Aurantimicrobium sp MWH Uga1 T05539 |
| 1349819 | Aureimonas sp AU20 T04161 |
| 2094025 | Aureitalea sp RR4 38 T05335 |
| 44056 | Aureococcus anophagefferens T04134 |
| 1817405 | Auricoccus indicus T04690 |
| 717982 | Auricularia subglabra TFB 10046 SS5 T03129 |
| 2170745 | Auritidibacter sp NML130574 T05590 |
| 3075 | Auxenochlorella protothecoides T04132 |
| 728 | Avibacterium paragallinarum T05728 |
| 41977 | Azoarcus communis T05456 |
| 418699 | Azoarcus olearius T04423 |
| 418699 | Azoarcus sp BH72 T00441 |
| 198107 | Azoarcus sp CIB T04019 |
| 356837 | Azoarcus sp DN11 T05691 |
| 748247 | Azoarcus sp KH32C T02502 |
| 2067960 | Azoarcus sp SY39 T05265 |
| 438753 | Azorhizobium caulinodans ORS 571 T00609 |
| 640081 | Azospira oryzae PS T01657 |
| 1064539 | Azospirillum brasilense Sp245 T02152 |
| 192 | Azospirillum brasilense T03288 |
| 192 | Azospirillum brasilense T04566 |
| 1226968 | Azospirillum humicireducens T04409 |
| 862719 | Azospirillum lipoferum 4B T01658 |
| 682998 | Azospirillum ramasamyi T05500 |
| 137722 | Azospirillum sp B510 T01169 |
| 664962 | Azospirillum sp TSH58 T05449 |
| 528244 | Azospirillum thiophilum T04091 |
| 1328314 | Azotobacter chroococcum NCIMB 8003 T03640 |
| 1283330 | Azotobacter vinelandii CA T02633 |
| 1283331 | Azotobacter vinelandii CA6 T02634 |
| 322710 | Azotobacter vinelandii DJ T00885 |
| 484906 | Babesia bovis T2Bo T01085 |
| 1133968 | Babesia microti strain RI T05554 |
| 293387 | Bacillus altitudinis T05199 |
| 1412898 | Bacillus amyloliquefaciens CC178 T02893 |
| 692420 | Bacillus amyloliquefaciens DSM 7 ATCC 23350 T01321 |
| 1091041 | Bacillus amyloliquefaciens IT 45 T02462 |
| 1415165 | Bacillus amyloliquefaciens LFB112 T02965 |
| 1001582 | Bacillus amyloliquefaciens LL3 T01835 |
| 999891 | Bacillus amyloliquefaciens TA208 T01824 |
| 1034836 | Bacillus amyloliquefaciens XH7 T01836 |
| 1155777 | Bacillus amyloliquefaciens Y2 T02108 |
| 592021 | Bacillus anthracis str A0248 T00892 |
| 743835 | Bacillus anthracis str A16 T02998 |
| 673518 | Bacillus anthracis str A16R T02999 |
| 261594 | Bacillus anthracis str Ames Ancestor T00178 |
| 198094 | Bacillus anthracis str Ames T00129 |
| 568206 | Bacillus anthracis str CDC 684 T00886 |
| 768494 | Bacillus anthracis str H9401 T02028 |
| 260799 | Bacillus anthracis str Sterne T00184 |
| 1392837 | Bacillus anthracis str SVA11 T03162 |
| 261591 | Bacillus anthracis str Vollum T03475 |
| 1392 | Bacillus anthracis T03394 |
| 720555 | Bacillus atrophaeus 1942 T01339 |
| 632773 | Bacillus beveridgei T05114 |
| 1330043 | Bacillus bombysepticus str Wang T03597 |
| 649639 | Bacillus cellulosilyticus DSM 2522 T01389 |
| 572264 | Bacillus cereus 03BB102 T00869 |
| 405534 | Bacillus cereus AH187 T00799 |
| 405535 | Bacillus cereus AH820 T00814 |
| 222523 | Bacillus cereus ATCC 10987 T00162 |
| 226900 | Bacillus cereus ATCC 14579 T00127 |
| 405532 | Bacillus cereus B4264 T00805 |
| 637380 | Bacillus cereus biovar anthracis str CI T01279 |
| 288681 | Bacillus cereus E33L T00198 |
| 347495 | Bacillus cereus F837 76 T01703 |
| 1217984 | Bacillus cereus FRI 35 T02291 |
| 405531 | Bacillus cereus G9842 T00815 |
| 334406 | Bacillus cereus NC7401 T01686 |
| 361100 | Bacillus cereus Q1 T00852 |
| 1396 | Bacillus cereus T03417 |
| 66692 | Bacillus clausii KSM K16 T00228 |
| 941639 | Bacillus coagulans 2 6 T01527 |
| 345219 | Bacillus coagulans 36D1 T01628 |
| 1121088 | Bacillus coagulans DSM 1 ATCC 7050 T03712 |
| 315749 | Bacillus cytotoxicus NVH 391 98 T00560 |
| 1402861 | Bacillus endophyticus T03983 |
| 86664 | Bacillus flexus T04803 |
| 79881 | Bacillus gibsonii T04820 |
| 1664069 | Bacillus glycinifermentans T04537 |
| 272558 | Bacillus halodurans C 125 T00039 |
| 79883 | Bacillus horikoshii T04904 |
| 1367477 | Bacillus infantis NRRL B 14911 T02860 |
| 96241 | Bacillus intestinalis T05513 |
| 859143 | Bacillus kochii T05194 |
| 199441 | Bacillus krulwichiae T04947 |
| 1246626 | Bacillus lehensis G1 T03204 |
| 279010 | Bacillus licheniformis DSM 13 ATCC 14580 T00197 |
| 279010 | Bacillus licheniformis DSM 13 ATCC 14580 T00200 |
| 592022 | Bacillus megaterium DSM 319 T01219 |
| 1348623 | Bacillus megaterium NBRC 15308 ATCC 14581 T03825 |
| 545693 | Bacillus megaterium QM B1551 T01208 |
| 1006007 | Bacillus megaterium WSH 002 T01890 |
| 796606 | Bacillus methanolicus MGA3 T03209 |
| 264697 | Bacillus muralis T05340 |
| 315730 | Bacillus mycoides KBAB4 T00642 |
| 1405 | Bacillus mycoides T03453 |
| 1405 | Bacillus mycoides T03798 |
| 1196031 | Bacillus oceanisediminis 2691 T04330 |
| 766760 | Bacillus paralicheniformis ATCC 9945a T02694 |
| 398511 | Bacillus pseudofirmus OF4 T01165 |
| 64104 | Bacillus pseudomycoides T03239 |
| 315750 | Bacillus pumilus SAFR 032 T00599 |
| 1408 | Bacillus pumilus T03175 |
| 1408 | Bacillus pumilus T04776 |
| 561879 | Bacillus safensis T05386 |
| 439292 | Bacillus selenitireducens MLS10 T01243 |
| 1478 | Bacillus simplex T04304 |
| 1479 | Bacillus smithii T03984 |
| 666686 | Bacillus sp 1NLA3E T02643 |
| 1570330 | Bacillus sp BH072 T03582 |
| 1806506 | Bacillus sp BS34A T03833 |
| 1127744 | Bacillus sp JS T02027 |
| 1628753 | Bacillus sp LM 4 2 T03908 |
| 98228 | Bacillus sp OxB 1 T03635 |
| 1446792 | Bacillus sp Pc3 T03544 |
| 1774743 | Bacillus sp SDLI1 T05207 |
| 756828 | Bacillus sp WP8 T03530 |
| 1565991 | Bacillus sp X1 T03242 |
| 1574141 | Bacillus sp YP1 T03701 |
| 936156 | Bacillus subtilis BSn5 T01417 |
| 1415167 | Bacillus subtilis PY79 T02937 |
| 1220533 | Bacillus subtilis QB928 T02292 |
| 645657 | Bacillus subtilis subsp natto BEST195 T02607 |
| 655816 | Bacillus subtilis subsp spizizenii str W23 T01302 |
| 1052585 | Bacillus subtilis subsp spizizenii TU B 10 T01635 |
| 1147161 | Bacillus subtilis subsp subtilis 6051 HGW T02503 |
| 224308 | Bacillus subtilis subsp subtilis str 168 T00010 |
| 1221328 | Bacillus subtilis subsp subtilis str AG1839 T03442 |
| 1302650 | Bacillus subtilis subsp subtilis str BAB 1 T02520 |
| 1192196 | Bacillus subtilis subsp subtilis str BSP1 T02384 |
| 1232554 | Bacillus subtilis subsp subtilis str JH642 substr AG174 T03443 |
| 1404258 | Bacillus subtilis subsp subtilis str OH 131 1 T03469 |
| 1052588 | Bacillus subtilis subsp subtilis str RO NN 1 T01837 |
| 1233100 | Bacillus subtilis XF 1 T02459 |
| 714359 | Bacillus thuringiensis BMB171 T01239 |
| 527021 | Bacillus thuringiensis Bt407 T02277 |
| 1218175 | Bacillus thuringiensis HD 771 T02293 |
| 1217737 | Bacillus thuringiensis HD 789 T02411 |
| 1195464 | Bacillus thuringiensis MC28 T02263 |
| 541229 | Bacillus thuringiensis serovar chinensis CT 43 T01838 |
| 930170 | Bacillus thuringiensis serovar finitimus YBT 020 T01839 |
| 281309 | Bacillus thuringiensis serovar konkukian str 97 27 T00179 |
| 1261129 | Bacillus thuringiensis serovar kurstaki str HD 1 T03473 |
| 1279365 | Bacillus thuringiensis serovar kurstaki str HD73 T02460 |
| 570416 | Bacillus thuringiensis serovar kurstaki str YBT 1520 T03435 |
| 1286404 | Bacillus thuringiensis serovar thuringiensis str IS5056 T02474 |
| 412694 | Bacillus thuringiensis str Al Hakam T00429 |
| 1428 | Bacillus thuringiensis T03675 |
| 1428 | Bacillus thuringiensis T04752 |
| 529122 | Bacillus thuringiensis YBT 1518 T02930 |
| 1415784 | Bacillus toyonensis BCT 7112 T02901 |
| 72361 | Bacillus vallismortis T04948 |
| 1225788 | Bacillus velezensis AS43 3 T02382 |
| 1114958 | Bacillus velezensis CAU B946 T01692 |
| 326423 | Bacillus velezensis FZB42 T00582 |
| 1385727 | Bacillus velezensis NAU B3 T02859 |
| 1423138 | Bacillus velezensis SQR9 T03384 |
| 492670 | Bacillus velezensis T03423 |
| 1449088 | Bacillus velezensis TrigoCor1448 T03161 |
| 1338518 | Bacillus velezensis UCMB5033 T02795 |
| 1150475 | Bacillus velezensis UCMB5036 T02480 |
| 1150476 | Bacillus velezensis UCMB5113 T02796 |
| 1155777 | Bacillus velezensis YAU B9601 Y2 T02050 |
| 1547283 | Bacillus weihaiensis T04822 |
| 2494319 | Bacillus wiedmannii bv thuringiensis T05831 |
| 1178537 | Bacillus xiamenensis T04852 |
| 1249553 | Bacterioplanes sanyensis T04982 |
| 960 | Bacteriovorax stolpii T05263 |
| 1898108 | Bacterium AB1 T04673 |
| 1400053 | Bacteroidales bacterium CF T02861 |
| 47678 | Bacteroides caccae T05064 |
| 1796613 | Bacteroides caecimuris T05202 |
| 246787 | Bacteroides cellulosilyticus T04116 |
| 357276 | Bacteroides dorei T03262 |
| 357276 | Bacteroides dorei T03263 |
| 862962 | Bacteroides fragilis 638R T01691 |
| 272559 | Bacteroides fragilis NCTC 9343 T00239 |
| 817 | Bacteroides fragilis T03897 |
| 295405 | Bacteroides fragilis YCH46 T00202 |
| 693979 | Bacteroides helcogenes P 36 108 T01393 |
| 28113 | Bacteroides heparinolyticus T05405 |
| 28116 | Bacteroides ovatus T04113 |
| 667015 | Bacteroides salanitronis DSM 18170 T01433 |
| 818 | Bacteroides thetaiotaomicron T04540 |
| 226186 | Bacteroides thetaiotaomicron VPI 5482 T00122 |
| 435590 | Bacteroides vulgatus ATCC 8482 T00546 |
| 657309 | Bacteroides xylanisolvens XB1A T02579 |
| 28119 | Bacteroides zoogleoformans T05371 |
| 1690483 | Bacteroidetes bacterium UKL13 3 T04336 |
| 310752 | Balaenoptera acutorostrata scammoni T03086 |
| 880074 | Barnesiella viscericola DSM 18177 T02986 |
| 1318743 | Bartonella ancashensis T04086 |
| 1686310 | Bartonella apis T05086 |
| 1094489 | Bartonella australis Aust NH1 T02469 |
| 360095 | Bartonella bacilliformis KC583 T00456 |
| 696125 | Bartonella clarridgeiae 73 T01394 |
| 634504 | Bartonella grahamii as4aup T00922 |
| 283166 | Bartonella henselae str Houston 1 T00180 |
| 38323 | Bartonella henselae T03068 |
| 38323 | Bartonella henselae T03309 |
| 1225179 | Bartonella quintana RM 11 T02294 |
| 283165 | Bartonella quintana str Toulouse T00181 |
| 515256 | Bartonella sp 1 1C T05463 |
| 1933910 | Bartonella sp A1379B T05185 |
| 1933906 | Bartonella sp JB15 T05113 |
| 1933907 | Bartonella sp JB63 T05552 |
| 1933912 | Bartonella sp Raccoon60 T05326 |
| 1933904 | Bartonella sp WD16 2 T05203 |
| 382640 | Bartonella tribocorum CIP 105476 T00636 |
| 85701 | Bartonella tribocorum T03233 |
| 1094497 | Bartonella vinsonii subsp berkhoffii str Winnie T02470 |
| 1072685 | Basilea psittacipulmonis DSM 24701 T03226 |
| 41875 | Bathycoccus prasinos T03089 |
| 717646 | Baudoinia panamericana UAMH 10762 T03106 |
| 374463 | Baumannia cicadellinicola str Hc T00349 |
| 264462 | Bdellovibrio bacteriovorus HD100 T00159 |
| 1069642 | Bdellovibrio bacteriovorus str Tiberius T02356 |
| 959 | Bdellovibrio bacteriovorus T03079 |
| 765869 | Bdellovibrio bacteriovorus W T03036 |
| 1184267 | Bdellovibrio exovorus JSS T02492 |
| 1916293 | Bdellovibrio sp qaytius T05880 |
| 288004 | Beggiatoa leptomitoformis T04090 |
| 395963 | Beijerinckia indica subsp indica ATCC 9039 T00688 |
| 866536 | Belliella baltica DSM 15883 T02134 |
| 1618337 | Berkelbacteria bacterium GW2011 GWE1 39 12 T03992 |
| 880071 | Bernardetia litoralis DSM 6794 T02149 |
| 543913 | Beta proteobacterium CB T02483 |
| 3555 | Beta vulgaris subsp vulgaris T03548 |
| 1904640 | Betaproteobacteria bacterium GR16 43 T04726 |
| 1690485 | Betaproteobacteria bacterium UKL13 2 T04342 |
| 471853 | Beutenbergia cavernae DSM 12333 T00893 |
| 1263829 | Bibersteinia trehalosi USDA ARS USMARC 188 T03017 |
| 1263831 | Bibersteinia trehalosi USDA ARS USMARC 189 T03018 |
| 1263832 | Bibersteinia trehalosi USDA ARS USMARC 190 T03019 |
| 1171377 | Bibersteinia trehalosi USDA ARS USMARC 192 T02504 |
| 1437605 | Bifidobacterium actinocoloniiforme DSM 22766 T04036 |
| 367928 | Bifidobacterium adolescentis ATCC 15703 T00437 |
| 1680 | Bifidobacterium adolescentis T03260 |
| 1680 | Bifidobacterium adolescentis T03774 |
| 518635 | Bifidobacterium angulatum DSM 20098 JCM 7096 T03930 |
| 703613 | Bifidobacterium animalis subsp animalis ATCC 25527 T02047 |
| 442563 | Bifidobacterium animalis subsp lactis AD011 T00819 |
| 1167629 | Bifidobacterium animalis subsp lactis ATCC 27673 T02862 |
| 1168290 | Bifidobacterium animalis subsp lactis B420 T02079 |
| 552531 | Bifidobacterium animalis subsp lactis BB 12 T01840 |
| 742729 | Bifidobacterium animalis subsp lactis Bi 07 T02080 |
| 580050 | Bifidobacterium animalis subsp lactis Bl 04 T00923 |
| 1281781 | Bifidobacterium animalis subsp lactis Bl12 T02712 |
| 1075106 | Bifidobacterium animalis subsp lactis BLC1 T01841 |
| 1042403 | Bifidobacterium animalis subsp lactis CNCM I 2494 T01842 |
| 555970 | Bifidobacterium animalis subsp lactis DSM 10140 T00924 |
| 573236 | Bifidobacterium animalis subsp lactis V9 T01843 |
| 28025 | Bifidobacterium animalis T03481 |
| 1147128 | Bifidobacterium asteroides PRL2011 T02271 |
| 484020 | Bifidobacterium bifidum BGN4 T02135 |
| 702459 | Bifidobacterium bifidum PRL2010 T01340 |
| 883062 | Bifidobacterium bifidum S17 T01328 |
| 1385938 | Bifidobacterium breve 12L T03118 |
| 1385942 | Bifidobacterium breve 689b T03119 |
| 866777 | Bifidobacterium breve ACS 071 V Sch8b T01844 |
| 518634 | Bifidobacterium breve DSM 20213 JCM 1192 T04525 |
| 1385939 | Bifidobacterium breve JCM 7017 T03120 |
| 1385940 | Bifidobacterium breve JCM 7019 T03121 |
| 1385941 | Bifidobacterium breve NCFB 2258 T03122 |
| 936351 | Bifidobacterium breve S27 T03123 |
| 326426 | Bifidobacterium breve UCC2003 T02534 |
| 566552 | Bifidobacterium catenulatum DSM 16992 JCM 1194 LMG 11043 T03931 |
| 1150460 | Bifidobacterium catenulatum subsp kashiwanohense JCM 15439 DSM 21854 T04223 |
| 35760 | Bifidobacterium choerinum T05748 |
| 1687 | Bifidobacterium coryneforme T03236 |
| 401473 | Bifidobacterium dentium Bd1 T01143 |
| 1150423 | Bifidobacterium dentium JCM 1195 DSM 20436 T04222 |
| 1341694 | Bifidobacterium indicum LMG 11587 DSM 20214 T03564 |
| 1447716 | Bifidobacterium kashiwanohense PV20 2 T03496 |
| 205913 | Bifidobacterium longum DJO10A T00707 |
| 206672 | Bifidobacterium longum NCC2705 T00094 |
| 565040 | Bifidobacterium longum subsp infantis 157F T01420 |
| 391904 | Bifidobacterium longum subsp infantis ATCC 15697 JCM 1222 DSM 20088 T00794 |
| 391904 | Bifidobacterium longum subsp infantis ATCC 15697 JCM 1222 DSM 20088 T02092 |
| 890402 | Bifidobacterium longum subsp longum BBMN68 T01345 |
| 722911 | Bifidobacterium longum subsp longum F8 T02580 |
| 1300227 | Bifidobacterium longum subsp longum GT15 T03452 |
| 565042 | Bifidobacterium longum subsp longum JCM 1217 T01419 |
| 759350 | Bifidobacterium longum subsp longum JDM301 T01240 |
| 1035817 | Bifidobacterium longum subsp longum KACC 91563 T01845 |
| 216816 | Bifidobacterium longum T03387 |
| 547043 | Bifidobacterium pseudocatenulatum DSM 20438 JCM 1200 LMG 10505 T03932 |
| 1447715 | Bifidobacterium pseudolongum PV8 2 T03529 |
| 1150461 | Bifidobacterium scardovii JCM 12489 DSM 13734 T03975 |
| 1254439 | Bifidobacterium thermophilum RBL67 T02505 |
| 930090 | Bipolaris oryzae ATCC 44560 T03125 |
| 665912 | Bipolaris sorokiniana ND90Pr T03124 |
| 930089 | Bipolaris zeicola 26 R 13 T03110 |
| 2233851 | Blastochloris tepida T05837 |
| 1079 | Blastochloris viridis T04109 |
| 1146883 | Blastococcus saxobsidens DD2 T01752 |
| 1550728 | Blastomonas fulva T05612 |
| 1842535 | Blastomonas sp RAC04 T04450 |
| 1229512 | Blattabacterium cuenoti BPAA T02525 |
| 331104 | Blattabacterium sp str Bge T00995 |
| 600809 | Blattabacterium sp str BPLAN T00996 |
| 1075399 | Blattabacterium sp str Cpu T01660 |
| 1074889 | Blattabacterium sp str MADAR T01642 |
| 1072467 | Blattabacterium sp str Tarazona T02446 |
| 1186051 | Blattabacterium sp T02109 |
| 1316444 | Blattabacterium sp T02863 |
| 298656 | Blattabacterium sp T05708 |
| 537007 | Blautia hansenii DSM 20583 T05095 |
| 657314 | Blautia obeum A2 162 T02605 |
| 33035 | Blautia producta T05894 |
| 1912897 | Blautia sp N6H1 15 T05518 |
| 1796616 | Blautia sp YL58 T04458 |
| 1505597 | Blochmannia endosymbiont of Camponotus obliquus T03906 |
| 1505596 | Blochmannia endosymbiont of Polyrhachis turneri T03907 |
| 150288 | Boleophthalmus pectinirostris T05002 |
| 132113 | Bombus impatiens T04362 |
| 30195 | Bombus terrestris T04364 |
| 7091 | Bombyx mori T02817 |
| 360910 | Bordetella avium 197N T00695 |
| 463025 | Bordetella bronchialis T04591 |
| 568707 | Bordetella bronchiseptica 253 T02344 |
| 1208658 | Bordetella bronchiseptica MO149 T02295 |
| 257310 | Bordetella bronchiseptica RB50 T00141 |
| 518 | Bordetella bronchiseptica T03637 |
| 463014 | Bordetella flabilis T04592 |
| 463040 | Bordetella genomosp 13 T05441 |
| 103855 | Bordetella hinzii T04004 |
| 1172205 | Bordetella holmesii 44057 T03379 |
| 1247649 | Bordetella holmesii ATCC 51541 T03052 |
| 257311 | Bordetella parapertussis 12822 T00140 |
| 1208660 | Bordetella parapertussis Bpp5 T02297 |
| 1403053 | Bordetella pertussis 137 T03754 |
| 568706 | Bordetella pertussis 18323 T02296 |
| 743277 | Bordetella pertussis B1917 T03638 |
| 1017264 | Bordetella pertussis CS T01846 |
| 257313 | Bordetella pertussis Tohama I T00139 |
| 340100 | Bordetella petrii DSM 12804 T00637 |
| 1331258 | Bordetella pseudohinzii T04945 |
| 1697043 | Bordetella sp H567 T04979 |
| 2163011 | Bordetella sp HZ20 T05532 |
| 1977852 | Bordetella sp J329 T05850 |
| 123899 | Bordetella trematum T04322 |
| 1365188 | Borrelia anserina Es T04727 |
| 1155096 | Borrelia crocidurae str Achema T02051 |
| 412419 | Borrelia duttonii Ly T00768 |
| 1329908 | Borrelia hermsii CC1 T03871 |
| 314723 | Borrelia hermsii DAH T00764 |
| 1674146 | Borrelia mayonii T04886 |
| 1302858 | Borrelia miyamotoi LB 2001 T02797 |
| 47466 | Borrelia miyamotoi T03577 |
| 1435045 | Borrelia parkeri HR1 T03987 |
| 412418 | Borrelia recurrentis A1 T00769 |
| 1104446 | Borrelia turcica IST7 T05670 |
| 314724 | Borrelia turicatae 91E135 T00737 |
| 1239934 | Borreliella afzelii HLJ01 T02298 |
| 411555 | Borreliella afzelii K78 T03895 |
| 390236 | Borreliella afzelii PKo T00383 |
| 390236 | Borreliella afzelii PKo T02093 |
| 1398490 | Borreliella afzelii Tom3107 T03256 |
| 290434 | Borreliella bavariensis PBi T00194 |
| 521010 | Borreliella bissettii DN127 T01593 |
| 224326 | Borreliella burgdorferi B31 T00012 |
| 1328311 | Borreliella burgdorferi CA382 T02794 |
| 521008 | Borreliella burgdorferi JD1 T02081 |
| 521007 | Borreliella burgdorferi N40 T02075 |
| 445985 | Borreliella burgdorferi ZS7 T00807 |
| 1245910 | Borreliella chilensis T03543 |
| 1081646 | Borreliella garinii BgVir T02048 |
| 1234596 | Borreliella garinii NMJW1 T02299 |
| 1421551 | Borreliella garinii SZ T03259 |
| 29519 | Borreliella garinii T04731 |
| 1398491 | Borreliella valaisiana Tom4006 T03221 |
| 9915 | Bos indicus T04792 |
| 72004 | Bos mutus T02919 |
| 9913 | Bos taurus T01008 |
| 1792307 | Bosea sp PAMC 26642 T04264 |
| 1842539 | Bosea sp RAC05 T04462 |
| 1867715 | Bosea sp Tri 49 T05753 |
| 1526658 | Bosea vaviloviae T04483 |
| 332648 | Botrytis cinerea B05 10 T01072 |
| 2017485 | Brachybacterium avium T04950 |
| 446465 | Brachybacterium faecium DSM 4810 T00965 |
| 1331682 | Brachybacterium ginsengisoli T05099 |
| 556288 | Brachybacterium saurashtrense T05573 |
| 1903186 | Brachybacterium sp P6 10 X1 T04713 |
| 2017484 | Brachybacterium vulturis T05108 |
| 15368 | Brachypodium distachyon T01717 |
| 1287055 | Brachyspira hampsonii T05655 |
| 1266923 | Brachyspira hyodysenteriae ATCC 27164 T04742 |
| 565034 | Brachyspira hyodysenteriae WA1 T00865 |
| 1045858 | Brachyspira intermedia PWS A T01847 |
| 526224 | Brachyspira murdochii DSM 12563 T01229 |
| 759914 | Brachyspira pilosicoli 95 1000 T01278 |
| 1133568 | Brachyspira pilosicoli B2904 T02225 |
| 1042417 | Brachyspira pilosicoli P43 6 78 T02387 |
| 1161918 | Brachyspira pilosicoli WesB T02412 |
| 1548548 | Bradymonas sediminis T05497 |
| 1404768 | Bradyrhizobium amphicarpaeae T05700 |
| 224911 | Bradyrhizobium diazoefficiens USDA 110 T00109 |
| 1274631 | Bradyrhizobium icense T04485 |
| 375 | Bradyrhizobium japonicum T03509 |
| 1037409 | Bradyrhizobium japonicum USDA 6 T01848 |
| 1245469 | Bradyrhizobium oligotrophicum S58 T02484 |
| 931866 | Bradyrhizobium ottawaense T05524 |
| 288000 | Bradyrhizobium sp BTAi1 T00528 |
| 1223566 | Bradyrhizobium sp CCGE LA001 T04273 |
| 114615 | Bradyrhizobium sp ORS 278 T00516 |
| 115808 | Bradyrhizobium sp ORS 285 T04956 |
| 1404864 | Bradyrhizobium sp S23321 T01796 |
| 2057741 | Bradyrhizobium sp SK17 T05325 |
| 376 | Bradyrhizobium sp T04337 |
| 7739 | Branchiostoma floridae T01074 |
| 3708 | Brassica napus T04128 |
| 109376 | Brassica oleracea var oleracea T04124 |
| 3711 | Brassica rapa T03449 |
| 1109412 | Brenneria goodwinii T05061 |
| 2304600 | Breoghania sp L A4 T05594 |
| 1986204 | Brevefilum fermentans T04881 |
| 51101 | Brevibacillus agri T05855 |
| 358681 | Brevibacillus brevis NBRC 100599 T00879 |
| 54913 | Brevibacillus formosus T04939 |
| 1042163 | Brevibacillus laterosporus LMG 15441 T03762 |
| 273384 | Brevibacterium aurantiacum T04775 |
| 1232385 | Brevibacterium flavum ZL 1 T04445 |
| 1703 | Brevibacterium linens T04319 |
| 1280413 | Breviolum minutum Mf 1 05b 01 T04349 |
| 293 | Brevundimonas diminuta T05851 |
| 588932 | Brevundimonas naejangsanensis T04375 |
| 1532555 | Brevundimonas sp DS20 T04101 |
| 1827469 | Brevundimonas sp GW460 12 10 14 LB2 T04395 |
| 1938605 | Brevundimonas sp LM2 T04750 |
| 633149 | Brevundimonas subvibrioides ATCC 15264 T01291 |
| 41276 | Brevundimonas vesicularis T04934 |
| 2756 | Brochothrix thermosphacta T05089 |
| 359391 | Brucella abortus 2308 T00304 |
| 1104320 | Brucella abortus A13334 T01704 |
| 262698 | Brucella abortus bv 1 str 9 941 T00241 |
| 520450 | Brucella abortus bv 2 str 86 8 59 T03321 |
| 520454 | Brucella abortus bv 6 str 870 T03392 |
| 520455 | Brucella abortus bv 9 str C68 T03474 |
| 430066 | Brucella abortus S19 T00703 |
| 235 | Brucella abortus T03461 |
| 235 | Brucella abortus T03462 |
| 235 | Brucella abortus T03685 |
| 235 | Brucella abortus T03686 |
| 483179 | Brucella canis ATCC 23365 T00623 |
| 1104321 | Brucella canis HSK A52141 T01705 |
| 1408887 | Brucella canis str Oliveri T03030 |
| 36855 | Brucella canis T03270 |
| 36855 | Brucella canis T03305 |
| 1423891 | Brucella ceti TE10759 12 T02934 |
| 1407053 | Brucella ceti TE28753 12 T02935 |
| 546272 | Brucella melitensis ATCC 23457 T00867 |
| 224914 | Brucella melitensis bv 1 str 16M T00072 |
| 224914 | Brucella melitensis bv 1 str 16M T03831 |
| 520466 | Brucella melitensis bv 3 str Ether T03393 |
| 941967 | Brucella melitensis M28 T01852 |
| 703352 | Brucella melitensis M5 90 T01849 |
| 1029825 | Brucella melitensis NI T01853 |
| 568815 | Brucella microti CCM 4915 T00955 |
| 444178 | Brucella ovis ATCC 25840 T00534 |
| 520461 | Brucella pinnipedialis B2 94 T01589 |
| 120576 | Brucella pinnipedialis T03261 |
| 1891098 | Brucella sp 2002734562 T05682 |
| 204722 | Brucella suis 1330 T00096 |
| 204722 | Brucella suis 1330 T02103 |
| 470137 | Brucella suis ATCC 23445 T00638 |
| 1004954 | Brucella suis bv 1 str S2 T03181 |
| 645170 | Brucella suis bv 2 T03367 |
| 645170 | Brucella suis bv 2 T03503 |
| 645170 | Brucella suis bv 2 T03504 |
| 645170 | Brucella suis bv 2 T03741 |
| 520487 | Brucella suis bv 3 str 686 T03266 |
| 29461 | Brucella suis T03426 |
| 29461 | Brucella suis T03427 |
| 1112912 | Brucella suis VBI22 T01706 |
| 981386 | Brucella vulpis T04257 |
| 6279 | Brugia malayi T01050 |
| 372461 | Buchnera aphidicola BCc T00423 |
| 563178 | Buchnera aphidicola str 5A T00820 |
| 1005090 | Buchnera aphidicola str Ak T01825 |
| 107806 | Buchnera aphidicola str APS T00036 |
| 224915 | Buchnera aphidicola str Bp T00111 |
| 1009859 | Buchnera aphidicola str F009 T03020 |
| 1009858 | Buchnera aphidicola str G002 T03021 |
| 713600 | Buchnera aphidicola str JF98 T01826 |
| 713601 | Buchnera aphidicola str JF99 T02354 |
| 713603 | Buchnera aphidicola str LL01 T01828 |
| 198804 | Buchnera aphidicola str Sg T00087 |
| 713602 | Buchnera aphidicola str TLW03 T01829 |
| 561501 | Buchnera aphidicola str Tuc7 T00821 |
| 1005057 | Buchnera aphidicola str Ua T01830 |
| 1009856 | Buchnera aphidicola str USDA T03022 |
| 1009857 | Buchnera aphidicola str W106 T03023 |
| 261317 | Buchnera aphidicola T01528 |
| 1265350 | Buchnera aphidicola T04764 |
| 339670 | Burkholderia ambifaria AMMD T00398 |
| 398577 | Burkholderia ambifaria MC40 6 T00684 |
| 331271 | Burkholderia cenocepacia AU 1054 T00363 |
| 1055524 | Burkholderia cenocepacia H111 T03804 |
| 331272 | Burkholderia cenocepacia HI2424 T00415 |
| 216591 | Burkholderia cenocepacia J2315 T00771 |
| 406425 | Burkholderia cenocepacia MC0 3 T00669 |
| 95486 | Burkholderia cenocepacia T03308 |
| 95486 | Burkholderia cenocepacia T03488 |
| 983594 | Burkholderia cepacia ATCC 25416 T04772 |
| 1009846 | Burkholderia cepacia GG4 T02205 |
| 292 | Burkholderia cepacia T03302 |
| 488447 | Burkholderia contaminans T03969 |
| 488732 | Burkholderia diffusa T04698 |
| 350701 | Burkholderia dolosa AU0158 T03793 |
| 999541 | Burkholderia gladioli BSR3 T01464 |
| 28095 | Burkholderia gladioli T03826 |
| 626418 | Burkholderia glumae BGR1 T00905 |
| 1176492 | Burkholderia glumae LMG 2196 ATCC 33617 T03820 |
| 758793 | Burkholderia insecticola T02684 |
| 482957 | Burkholderia lata T00286 |
| 488446 | Burkholderia latens T04819 |
| 243160 | Burkholderia mallei ATCC 23344 T00204 |
| 412022 | Burkholderia mallei NCTC 10229 T00471 |
| 320389 | Burkholderia mallei NCTC 10247 T00479 |
| 320389 | Burkholderia mallei NCTC 10247 T03524 |
| 320388 | Burkholderia mallei SAVP1 T00457 |
| 13373 | Burkholderia mallei T03323 |
| 13373 | Burkholderia mallei T03489 |
| 13373 | Burkholderia mallei T03490 |
| 13373 | Burkholderia mallei T03522 |
| 13373 | Burkholderia mallei T03523 |
| 13373 | Burkholderia mallei T03872 |
| 488729 | Burkholderia metallica T04871 |
| 395019 | Burkholderia multivorans ATCC 17616 T00624 |
| 395019 | Burkholderia multivorans ATCC 17616 T00740 |
| 985079 | Burkholderia multivorans ATCC BAA 247 T03881 |
| 87883 | Burkholderia multivorans T03292 |
| 441162 | Burkholderia oklahomensis C6786 T03861 |
| 342113 | Burkholderia oklahomensis T03284 |
| 41899 | Burkholderia plantarii T03632 |
| 41899 | Burkholderia plantarii T04139 |
| 884204 | Burkholderia pseudomallei 1026b T02049 |
| 357348 | Burkholderia pseudomallei 1106a T00480 |
| 320372 | Burkholderia pseudomallei 1710b T00289 |
| 320373 | Burkholderia pseudomallei 668 T00481 |
| 1439855 | Burkholderia pseudomallei A79A T03498 |
| 1229785 | Burkholderia pseudomallei BPC006 T02301 |
| 1306418 | Burkholderia pseudomallei HBPUB10134a T03422 |
| 272560 | Burkholderia pseudomallei K96243 T00203 |
| 1249471 | Burkholderia pseudomallei MSHR146 T03024 |
| 1335307 | Burkholderia pseudomallei MSHR305 T02756 |
| 536230 | Burkholderia pseudomallei MSHR346 T00894 |
| 1249474 | Burkholderia pseudomallei MSHR511 T03012 |
| 1249659 | Burkholderia pseudomallei MSHR520 T03163 |
| 1249477 | Burkholderia pseudomallei NAU35A 3 T03476 |
| 1241583 | Burkholderia pseudomallei NCTC 13179 T02894 |
| 28450 | Burkholderia pseudomallei T03428 |
| 1207504 | Burkholderia pseudomultivorans T04870 |
| 60550 | Burkholderia pyrrocinia T03962 |
| 488731 | Burkholderia seminalis T04851 |
| 1468409 | Burkholderia sp 2002721687 T03759 |
| 430531 | Burkholderia sp Bp5365 T04266 |
| 640510 | Burkholderia sp CCGE1001 T01424 |
| 640512 | Burkholderia sp CCGE1003 T01314 |
| 1678678 | Burkholderia sp HB1 T04094 |
| 416344 | Burkholderia sp KJ006 T02110 |
| 1417228 | Burkholderia sp OLGA172 T05111 |
| 1795043 | Burkholderia sp PAMC 26561 T05481 |
| 1795874 | Burkholderia sp PAMC 28687 T05699 |
| 758796 | Burkholderia sp RPE67 T03604 |
| 1097668 | Burkholderia sp YI23 T01711 |
| 95485 | Burkholderia stabilis T04946 |
| 1503054 | Burkholderia stagnalis T04885 |
| 1503055 | Burkholderia territorii T04843 |
| 1249660 | Burkholderia thailandensis 2002721643 T03882 |
| 1241582 | Burkholderia thailandensis 2002721723 T03111 |
| 1249665 | Burkholderia thailandensis E254 T03380 |
| 271848 | Burkholderia thailandensis E264 T00311 |
| 1249662 | Burkholderia thailandensis E444 T03112 |
| 1249663 | Burkholderia thailandensis H0587 T03113 |
| 1249661 | Burkholderia thailandensis MSMB121 T02644 |
| 1250339 | Burkholderia thailandensis MSMB59 T03416 |
| 57975 | Burkholderia thailandensis T03763 |
| 1249667 | Burkholderia thailandensis USAMRU Malaysia 20 T03391 |
| 1249668 | Burkholderia ubonensis MSMB22 T03794 |
| 269482 | Burkholderia vietnamiensis G4 T00493 |
| 1449978 | Burkholderia vietnamiensis LMG 10929 T03893 |
| 1469502 | Burkholderiales bacterium GJ E10 T03654 |
| 1834205 | Burkholderiales bacterium YL45 T04607 |
| 2479367 | Buttiauxella sp 3AFRM03 T05676 |
| 245012 | Butyrate producing bacterium SM4 1 T02610 |
| 245014 | Butyrate producing bacterium SS3 4 T02611 |
| 2093856 | Butyricimonas faecalis T05785 |
| 657324 | Butyrivibrio fibrisolvens 16 4 T02581 |
| 185008 | Butyrivibrio hungatei T04542 |
| 515622 | Butyrivibrio proteoclasticus B316 T01292 |
| 6238 | Caenorhabditis briggsae T01054 |
| 6239 | Caenorhabditis elegans T00019 |
| 3821 | Cajanus cajan T04868 |
| 273068 | Caldanaerobacter subterraneus subsp tengcongensis MB4 T00081 |
| 521460 | Caldicellulosiruptor bescii DSM 6725 T00857 |
| 632292 | Caldicellulosiruptor hydrothermalis 108 T01346 |
| 632335 | Caldicellulosiruptor kristjanssonii I77R1B T01356 |
| 632348 | Caldicellulosiruptor kronotskyensis 2002 T01357 |
| 632516 | Caldicellulosiruptor lactoaceticus 6A T01600 |
| 608506 | Caldicellulosiruptor obsidiansis OB47 T01293 |
| 632518 | Caldicellulosiruptor owensensis OL T01347 |
| 351627 | Caldicellulosiruptor saccharolyticus DSM 8903 T00506 |
| 926550 | Caldilinea aerophila DSM 14535 NBRC 104270 T01797 |
| 1653476 | Caldimicrobium thiodismutans T04224 |
| 511051 | Caldisericum exile AZM16c01 T01804 |
| 1056495 | Caldisphaera lagunensis DSM 15908 T02380 |
| 768670 | Calditerrivibrio nitroreducens DSM 19672 T01364 |
| 880073 | Caldithrix abyssi DSM 13497 T04549 |
| 397948 | Caldivirga maquilingensis IC 167 T00611 |
| 9483 | Callithrix jacchus T03264 |
| 7868 | Callorhinchus milii T03088 |
| 1337936 | Calothrix sp 336 3 T03937 |
| 1170562 | Calothrix sp PCC 6303 T02372 |
| 99598 | Calothrix sp PCC 7507 T02358 |
| 90675 | Camelina sativa T05044 |
| 9838 | Camelus dromedarius T04642 |
| 419612 | Camelus ferus T02979 |
| 104421 | Camponotus floridanus T03919 |
| 522484 | Campylobacter avium LMG 24591 T05533 |
| 1358410 | Campylobacter coli 15 537360 T02895 |
| 1367491 | Campylobacter coli 76339 T02811 |
| 1273173 | Campylobacter coli CVM N29710 T02838 |
| 1183378 | Campylobacter coli RM1875 T03164 |
| 1183379 | Campylobacter coli RM4661 T03166 |
| 1183380 | Campylobacter coli RM5611 T03167 |
| 195 | Campylobacter coli T03870 |
| 195 | Campylobacter coli T04527 |
| 360104 | Campylobacter concisus 13826 T00593 |
| 199 | Campylobacter concisus T04635 |
| 1121267 | Campylobacter cuniculorum DSM 23162 LMG 24588 T05046 |
| 360105 | Campylobacter curvus 525 92 T00576 |
| 1240980 | Campylobacter fetus subsp fetus 04 554 T03330 |
| 360106 | Campylobacter fetus subsp fetus 82 40 T00430 |
| 1244528 | Campylobacter fetus subsp testudinum 03 427 T02899 |
| 1507806 | Campylobacter fetus subsp testudinum T03583 |
| 1093099 | Campylobacter fetus subsp venerealis 97 608 T03351 |
| 1273266 | Campylobacter fetus subsp venerealis cfvi03 293 T03000 |
| 688354 | Campylobacter fetus subsp venerealis str 84 112 T03740 |
| 824 | Campylobacter gracilis T04020 |
| 28898 | Campylobacter helveticus T04807 |
| 1813019 | Campylobacter hepaticus T05589 |
| 360107 | Campylobacter hominis ATCC BAA 381 T00577 |
| 1031746 | Campylobacter hyointestinalis subsp hyointestinalis LMG 9260 T04651 |
| 1244531 | Campylobacter iguaniorum T03252 |
| 1031564 | Campylobacter insulaenigrae NCTC 12927 T03555 |
| 1338035 | Campylobacter jejuni 32488 T02759 |
| 1347340 | Campylobacter jejuni 4031 T02864 |
| 195099 | Campylobacter jejuni RM1221 T00227 |
| 360109 | Campylobacter jejuni subsp doylei 269 97 T00573 |
| 1357994 | Campylobacter jejuni subsp jejuni 00 2425 T02844 |
| 1380767 | Campylobacter jejuni subsp jejuni 00 2426 T02841 |
| 1380768 | Campylobacter jejuni subsp jejuni 00 2538 T02842 |
| 1383068 | Campylobacter jejuni subsp jejuni 00 2544 T02843 |
| 354242 | Campylobacter jejuni subsp jejuni 81 176 T00461 |
| 407148 | Campylobacter jejuni subsp jejuni 81116 T00597 |
| 567106 | Campylobacter jejuni subsp jejuni IA3902 T01854 |
| 757425 | Campylobacter jejuni subsp jejuni ICDCCJ07001 T01372 |
| 645464 | Campylobacter jejuni subsp jejuni M1 T01855 |
| 192222 | Campylobacter jejuni subsp jejuni NCTC 11168 ATCC 700819 T00026 |
| 1211776 | Campylobacter jejuni subsp jejuni NCTC 11168 BN148 T02302 |
| 1201032 | Campylobacter jejuni subsp jejuni PT14 T02264 |
| 1295010 | Campylobacter jejuni subsp jejuni R14 T03187 |
| 718271 | Campylobacter jejuni subsp jejuni S3 T01856 |
| 32022 | Campylobacter jejuni subsp jejuni T03388 |
| 32022 | Campylobacter jejuni subsp jejuni T03744 |
| 32022 | Campylobacter jejuni subsp jejuni T03745 |
| 32022 | Campylobacter jejuni subsp jejuni T03791 |
| 32022 | Campylobacter jejuni subsp jejuni T03792 |
| 1031753 | Campylobacter lanienae NCTC 13004 T05075 |
| 1388750 | Campylobacter lari CCUG 22395 T03551 |
| 1388749 | Campylobacter lari NCTC 11845 T03552 |
| 1453989 | Campylobacter lari RM16701 T03550 |
| 1453988 | Campylobacter lari RM16712 T03556 |
| 306263 | Campylobacter lari RM2100 T00858 |
| 1031536 | Campylobacter lari subsp concheus LMG 11760 T03557 |
| 1388753 | Campylobacter peloridis LMG 23910 T03559 |
| 1874362 | Campylobacter pinnipediorum subsp caledonicus T05033 |
| 1660076 | Campylobacter sp RM12175 T05727 |
| 1500960 | Campylobacter sp RM16704 T03560 |
| 1031920 | Campylobacter sputorum bv faecalis CCUG 20703 T04992 |
| 1388751 | Campylobacter subantarcticus LMG 24374 T03571 |
| 1388752 | Campylobacter subantarcticus LMG 24377 T03572 |
| 1032069 | Campylobacter ureolyticus RIGS 9880 T04021 |
| 580033 | Campylobacter volucris LMG 24379 T03558 |
| 237561 | Candida albicans SC5314 T00189 |
| 498019 | Candida auris T04850 |
| 573826 | Candida dubliniensis CD36 T01140 |
| 284593 | Candida glabrata CBS 138 T01024 |
| 1136231 | Candida orthopsilosis Co 90 125 T02488 |
| 294747 | Candida tropicalis MYA 3404 T01115 |
| 1620412 | Candidate division Kazan bacterium GW2011 GWA1 50 15 T03988 |
| 1643353 | Candidate division SR1 bacterium Aalborg AAW 1 T04487 |
| 1394709 | Candidate division SR1 bacterium RAAC1 SR1 1 T02946 |
| 1619077 | Candidate division TM6 bacterium GW2011 GWF2 28 16 T04457 |
| 1394710 | Candidate division WWE3 bacterium RAAC2 WWE3 1 T02947 |
| 522306 | Candidatus Accumulibacter phosphatis clade IIA str UW 1 T00966 |
| 452471 | Candidatus Amoebophilus asiaticus 5a2 T00730 |
| 634113 | Candidatus Arsenophonus lipoptenae T04248 |
| 1029718 | Candidatus Arthromitus sp SFB mouse Japan T01591 |
| 1508644 | Candidatus Arthromitus sp SFB mouse NL T03243 |
| 1041809 | Candidatus Arthromitus sp SFB mouse Yit T01857 |
| 1041504 | Candidatus Arthromitus sp SFB rat Yit T01624 |
| 1453429 | Candidatus Atelocyanobacterium thalassa isolate ALOHA T01159 |
| 511995 | Candidatus Azobacteroides pseudotrichonymphae genomovar CFP2 T00792 |
| 673862 | Candidatus Babela massiliensis T02948 |
| 1700835 | Candidatus Bathyarchaeota archaeon BA1 T04065 |
| 1700836 | Candidatus Bathyarchaeota archaeon BA2 T04066 |
| 186490 | Candidatus Baumannia cicadellinicola T03277 |
| 186490 | Candidatus Baumannia cicadellinicola T04544 |
| 1618372 | Candidatus Beckwithbacteria bacterium GW2011 GWC1 49 16 T04022 |
| 2026885 | Candidatus Bipolaricaulis anaerobius T05525 |
| 2501609 | Candidatus Bipolaricaulis sibiricus T05809 |
| 1240471 | Candidatus Blochmannia chromaiodes str 640 T02430 |
| 203907 | Candidatus Blochmannia floridanus T00138 |
| 291272 | Candidatus Blochmannia pennsylvanicus str BPEN T00272 |
| 859654 | Candidatus Blochmannia vafer str BVAF T01398 |
| 311458 | Candidatus Caldiarchaeum subterraneum T02904 |
| 1618633 | Candidatus Campbellbacteria bacterium GW2011 OD1 34 28 T04488 |
| 247481 | Candidatus Cardinium hertigii T05479 |
| 1202536 | Candidatus Carsonella ruddii CE isolate Thao2000 T02194 |
| 1202537 | Candidatus Carsonella ruddii CS isolate Thao2000 T02195 |
| 667013 | Candidatus Carsonella ruddii DC T02774 |
| 1202538 | Candidatus Carsonella ruddii HC isolate Thao2000 T02197 |
| 1202539 | Candidatus Carsonella ruddii HT isolate Thao2000 T02196 |
| 1202540 | Candidatus Carsonella ruddii PC isolate NHV T02198 |
| 387662 | Candidatus Carsonella ruddii PV T00403 |
| 459349 | Candidatus Cloacimonas acidaminovorans str Evry T04769 |
| 2054173 | Candidatus Coxiella mudrowiae T03998 |
| 1457365 | Candidatus Cyclonatronum proteinivorum T05709 |
| 477974 | Candidatus Desulforudis audaxviator MP104C T00655 |
| 1725232 | Candidatus Desulfovibrio trichonymphae T05578 |
| 1778262 | Candidatus Doolittlea endobia T04480 |
| 1193729 | Candidatus Endolissoclinum faulkneri L2 T02365 |
| 1401328 | Candidatus Endolissoclinum faulkneri L5 T04221 |
| 1408204 | Candidatus Endomicrobium trichonymphae T05087 |
| 1495769 | Candidatus Evansia muelleri T03696 |
| 1608628 | Candidatus Filomicrobium marinum T03856 |
| 1608628 | Candidatus Filomicrobium marinum T03857 |
| 1798018 | Candidatus Fluviicola riflensis T05019 |
| 1802984 | Candidatus Fokinia solitaria T05415 |
| 2716812 | Candidatus Frankia datiscae T01530 |
| 1070130 | Candidatus Gullanella endobia T04502 |
| 572265 | Candidatus Hamiltonella defensa 5AT T00911 |
| 1427984 | Candidatus Hepatoplasma crinochetorum Av T03053 |
| 1778263 | Candidatus Hoaglandella endobia T04484 |
| 573234 | Candidatus Hodgkinia cicadicola Dsem T00938 |
| 573658 | Candidatus Hodgkinia cicadicola T03202 |
| 573658 | Candidatus Hodgkinia cicadicola T03595 |
| 573658 | Candidatus Hodgkinia cicadicola T03596 |
| 476281 | Candidatus Ishikawaella capsulata Mpkobe T04149 |
| 1541959 | Candidatus Izimaplasma sp HR1 T03291 |
| 1208923 | Candidatus Kinetoplastibacterium blastocrithidii T02427 |
| 1208922 | Candidatus Kinetoplastibacterium blastocrithidii TCC012E T02463 |
| 1267577 | Candidatus Kinetoplastibacterium crithidii T02413 |
| 1208918 | Candidatus Kinetoplastibacterium crithidii TCC036E T02464 |
| 1208919 | Candidatus Kinetoplastibacterium desouzaii TCC079E T02465 |
| 1208921 | Candidatus Kinetoplastibacterium galatii TCC219 T02466 |
| 1208920 | Candidatus Kinetoplastibacterium oncopeltii TCC290E T02471 |
| 1576550 | Candidatus Kinetoplastibacterium sorsogonicusi T05428 |
| 374847 | Candidatus Korarchaeum cryptofilum OPF8 T00668 |
| 204669 | Candidatus Koribacter versatilis Ellin345 T00352 |
| 174633 | Candidatus Kuenenia stuttgartiensis T05435 |
| 1277257 | Candidatus Liberibacter africanus PTSAPSY T03953 |
| 1261131 | Candidatus Liberibacter americanus str Sao Paulo T02902 |
| 1174529 | Candidatus Liberibacter asiaticus str gxpsy T02506 |
| 931202 | Candidatus Liberibacter asiaticus str Ishi 1 T03607 |
| 537021 | Candidatus Liberibacter asiaticus str psy62 T00948 |
| 658172 | Candidatus Liberibacter solanacearum CLso ZC1 T01373 |
| 1920749 | Candidatus Mancarchaeum acidiphilum T05015 |
| 1899017 | Candidatus Melainabacteria bacterium MEL A1 T04546 |
| 1295009 | Candidatus Methanomassiliicoccus intestinalis Issoire Mx1 T02692 |
| 1236689 | Candidatus Methanomethylophilus alvus Mx1201 T02624 |
| 1577791 | Candidatus Methanoplasma termitum T03540 |
| 671143 | Candidatus Methylomirabilis oxyfera T02155 |
| 1581557 | Candidatus Methylopumilus planktonicus T03869 |
| 1581680 | Candidatus Methylopumilus turicensis T03844 |
| 696127 | Candidatus Midichloria mitochondrii IricVA T01561 |
| 1778264 | Candidatus Mikella endobia T04510 |
| 903503 | Candidatus Moranella endobia PCIT T01562 |
| 1234603 | Candidatus Moranella endobia PCVAL T02613 |
| 1318617 | Candidatus Mycoplasma girerdii T03441 |
| 1212765 | Candidatus Mycoplasma haemolamae str Purdue T02329 |
| 1116213 | Candidatus Mycoplasma haemominutum Birmingham 1 T02582 |
| 1884916 | Candidatus Nanopelagicus abundans T05091 |
| 1884915 | Candidatus Nanopelagicus hibericus T05090 |
| 1884634 | Candidatus Nanopelagicus limnes T05073 |
| 1577684 | Candidatus Nanopusillus acidilobi T04255 |
| 1343077 | Candidatus Nasuia deltocephalinicola str NAS ALF T02782 |
| 2058097 | Candidatus Nitrosocaldus cavascurensis T05329 |
| 1353260 | Candidatus Nitrosocosmicus oleophilus T04185 |
| 1630141 | Candidatus Nitrosoglobus terrae T05173 |
| 1898749 | Candidatus Nitrosomarinus catalina T04846 |
| 1410606 | Candidatus Nitrosopelagicus brevis T03537 |
| 1229908 | Candidatus Nitrosopumilus koreensis AR1 T02304 |
| 1229909 | Candidatus Nitrosopumilus sediminis T02303 |
| 1459636 | Candidatus Nitrososphaera evergladensis SR1 T03224 |
| 1237085 | Candidatus Nitrososphaera gargensis Ga9 2 T02305 |
| 1078905 | Candidatus Nitrosotalea devanaterra T04889 |
| 1603555 | Candidatus Nitrosotenuis cloacae T03883 |
| 1715989 | Candidatus Nitrospira inopinata T04169 |
| 1414854 | Candidatus Nucleicultrix amoebiphila FS5 T04833 |
| 1235990 | Candidatus Pantoea carbekii T02865 |
| 1235990 | Candidatus Pantoea carbekii T03853 |
| 91604 | Candidatus Paracaedibacter acanthamoebae T03232 |
| 244581 | Candidatus Paracaedimonas acanthamoebae T03240 |
| 1002672 | Candidatus Pelagibacter sp IMCC9063 T01465 |
| 335992 | Candidatus Pelagibacter ubique HTCC1062 T00257 |
| 1735162 | Candidatus Peribacter riflensis T04147 |
| 2115978 | Candidatus Phycorickettsia trachydisci T05372 |
| 59748 | Candidatus Phytoplasma australiense T00752 |
| 37692 | Candidatus Phytoplasma mali T00729 |
| 69896 | Candidatus Phytoplasma solani T02881 |
| 135727 | Candidatus Phytoplasma ziziphi T05675 |
| 1884914 | Candidatus Planktophila dulcis T05051 |
| 1884913 | Candidatus Planktophila lacus T05050 |
| 573600 | Candidatus Planktophila limnetica T05067 |
| 1884904 | Candidatus Planktophila sulfonica T05158 |
| 1884907 | Candidatus Planktophila vernalis T05319 |
| 1884905 | Candidatus Planktophila versatilis T05250 |
| 1206109 | Candidatus Portiera aleyrodidarum BT B HRs T02206 |
| 1206109 | Candidatus Portiera aleyrodidarum BT B HRs T02267 |
| 1239881 | Candidatus Portiera aleyrodidarum BT QVLC T02306 |
| 1239881 | Candidatus Portiera aleyrodidarum BT QVLC T02307 |
| 1163752 | Candidatus Portiera aleyrodidarum MED T03834 |
| 91844 | Candidatus Portiera aleyrodidarum T03736 |
| 91844 | Candidatus Portiera aleyrodidarum T03737 |
| 91844 | Candidatus Portiera aleyrodidarum T03738 |
| 1297582 | Candidatus Portiera aleyrodidarum TV T02521 |
| 669502 | Candidatus Profftella armatura T02777 |
| 1806508 | Candidatus Promineofilum breve T05438 |
| 264201 | Candidatus Protochlamydia amoebophila UWE25 T00172 |
| 389348 | Candidatus Protochlamydia naegleriophila T04212 |
| 1302376 | Candidatus Pseudomonas adelgestsugas T05857 |
| 488538 | Candidatus Puniceispirillum marinum IMCC1322 T01207 |
| 472834 | Candidatus Purcelliella pentastirinorum T05580 |
| 535712 | Candidatus Rhodoluna planktonica T04564 |
| 676208 | Candidatus Rickettsiella viridis T05765 |
| 515618 | Candidatus Riesia pediculicola USDA T01218 |
| 1719125 | Candidatus Riesia sp GBBU T04782 |
| 413404 | Candidatus Ruthia magnifica str Cm T00438 |
| 1619070 | Candidatus Saccharibacteria bacterium GW2011 GWC2 44 17 T04605 |
| 1394711 | Candidatus Saccharibacteria bacterium RAAC3 TM7 1 T02942 |
| 2093824 | Candidatus Saccharibacteria oral taxon TM7x T03502 |
| 1332188 | Candidatus Saccharimonas aalborgensis T02673 |
| 2342 | Candidatus Sodalis pierantonius str SOPE T03006 |
| 234267 | Candidatus Solibacter usitatus Ellin6076 T00412 |
| 706194 | Candidatus Sulcia muelleri CARI T01303 |
| 641892 | Candidatus Sulcia muelleri DMIN T01203 |
| 444179 | Candidatus Sulcia muelleri GWSS T00630 |
| 1189303 | Candidatus Sulcia muelleri PSPU T03672 |
| 595499 | Candidatus Sulcia muelleri SMDSEM T00958 |
| 1343076 | Candidatus Sulcia muelleri str Sulcia ALF T02783 |
| 336810 | Candidatus Sulcia muelleri T03352 |
| 336810 | Candidatus Sulcia muelleri T03536 |
| 336810 | Candidatus Sulcia muelleri T03664 |
| 1249480 | Candidatus Sulfuricurvum sp RIFRC 1 T02537 |
| 946483 | Candidatus Symbiobacter mobilis CR T02877 |
| 1410383 | Candidatus Tachikawaea gelatinosa T03624 |
| 1748243 | Candidatus Tenderia electrophaga T04171 |
| 1166950 | Candidatus Thiodictyon syntrophicum T05211 |
| 1705394 | Candidatus Thioglobus autotrophicus T04079 |
| 1125411 | Candidatus Thioglobus singularis PS1 T04071 |
| 2508687 | Candidatus Thioglobus sp NP1 T05818 |
| 1902579 | Candidatus Tokpelaia hoelldoblerii T04701 |
| 1266371 | Candidatus Tremblaya phenacola PAVE T02908 |
| 891398 | Candidatus Tremblaya princeps PCIT T01563 |
| 1053648 | Candidatus Tremblaya princeps PCVAL T01858 |
| 1133592 | Candidatus Uzinura diaspidicola str ASNER T02439 |
| 412965 | Candidatus Vesicomyosocius okutanii T00530 |
| 881286 | Candidatus Vidania fulgoroideae T05569 |
| 1415657 | Candidatus Walczuchella monophlebidarum T03207 |
| 1618600 | Candidatus Woesebacteria bacterium GW2011 GWF1 31 35 T04464 |
| 1619007 | Candidatus Wolfebacteria bacterium GW2011 GWB1 47 1 T04023 |
| 1704307 | Candidatus Xiphinematobacter sp Idaho Grape T04108 |
| 871271 | Candidatus Zinderia insecticola CARI T01304 |
| 9615 | Canis lupus familiaris T01007 |
| 860228 | Capnocytophaga canimorsus Cc5 T01582 |
| 28189 | Capnocytophaga cynodegmi T05499 |
| 1017 | Capnocytophaga gingivalis T05260 |
| 45243 | Capnocytophaga haemolytica T04287 |
| 327575 | Capnocytophaga leadbetteri T05313 |
| 521097 | Capnocytophaga ochracea DSM 7271 T00961 |
| 2708117 | Capnocytophaga sp ChDC OS43 T04940 |
| 1945657 | Capnocytophaga sp H2931 T05889 |
| 1945658 | Capnocytophaga sp H4358 T05519 |
| 1705617 | Capnocytophaga sp oral taxon 323 T04076 |
| 1019 | Capnocytophaga sputigena T05393 |
| 1848904 | Capnocytophaga stomatis T05529 |
| 9925 | Capra hircus T02910 |
| 2507162 | Caproiciproducens sp NJN 50 T05807 |
| 81985 | Capsella rubella T02984 |
| 4072 | Capsicum annuum T04646 |
| 178899 | Carboxydocella thermautotrophica T05389 |
| 246194 | Carboxydothermus hydrogenoformans Z 2901 T00287 |
| 1231626 | Cardinium endosymbiont cEper1 of Encarsia pergandiella T02243 |
| 650378 | Cardinium endosymbiont of Sogatella furcifera T05597 |
| 2718 | Cardiobacterium hominis T05791 |
| 3649 | Carica papaya T05151 |
| 2748 | Carnobacterium divergens T05644 |
| 1266845 | Carnobacterium inhibens subsp gilichinskyi T02884 |
| 1234679 | Carnobacterium maltaromaticum LMA28 T02342 |
| 208596 | Carnobacterium sp 17 4 T01452 |
| 1564681 | Carnobacterium sp CP1 T04230 |
| 1437824 | Castellaniella defragrans 65Phen T03058 |
| 51338 | Castor canadensis T04791 |
| 2172099 | Catenovulum sp CCB QB4 T05408 |
| 479433 | Catenulispora acidiphila DSM 44928 T00967 |
| 1679497 | Caulobacter flavus T05702 |
| 69395 | Caulobacter henricii T04110 |
| 69666 | Caulobacter mirabilis T05166 |
| 509190 | Caulobacter segnis ATCC 21756 T01221 |
| 366602 | Caulobacter sp K31 T00651 |
| 190650 | Caulobacter vibrioides CB15 T00049 |
| 565050 | Caulobacter vibrioides NA1000 T00841 |
| 1759059 | Caulobacteraceae bacterium OTSz A 272 T04422 |
| 261658 | Cavenderia fasciculata T02668 |
| 158823 | Cedecea lapagei T05810 |
| 158822 | Cedecea neteri T03298 |
| 158822 | Cedecea neteri T03328 |
| 158822 | Cedecea neteri T03337 |
| 1758178 | Celeribacter ethanolicus T05094 |
| 1208324 | Celeribacter indicus T03575 |
| 1411902 | Celeribacter manganoxidans T05128 |
| 1397108 | Celeribacter marinus T04098 |
| 590998 | Cellulomonas fimi ATCC 484 T01490 |
| 446466 | Cellulomonas flavigena DSM 20109 T01230 |
| 593907 | Cellulomonas gilvus ATCC 13127 T01537 |
| 2003551 | Cellulomonas sp PSBB021 T04985 |
| 688270 | Cellulophaga algicola DSM 14237 T01404 |
| 1348584 | Cellulophaga baltica 18 T03565 |
| 1348585 | Cellulophaga baltica NN016038 T03562 |
| 867900 | Cellulophaga lytica DSM 7489 T01434 |
| 979 | Cellulophaga lytica T03279 |
| 642492 | Cellulosilyticum lentocellum DSM 5427 T01448 |
| 1710 | Cellulosimicrobium cellulans T04858 |
| 1980001 | Cellulosimicrobium sp TH 20 T04804 |
| 498211 | Cellvibrio japonicus Ueda107 T00721 |
| 1987723 | Cellvibrio sp PSBB006 T04891 |
| 1945512 | Cellvibrio sp PSBB023 T04725 |
| 414004 | Cenarchaeum symbiosum A T01380 |
| 759272 | Chaetomium thermophilum var thermophilum DSM 1495 T03100 |
| 1173020 | Chamaesiphon minutus PCC 6605 T02414 |
| 1441930 | Chania multitudinisentens RB 25 T02987 |
| 266779 | Chelativorans sp BNC1 T00374 |
| 444444 | Chelatococcus daeguensis T04583 |
| 1702325 | Chelatococcus sp CO 6 T04040 |
| 8469 | Chelonia mydas T02989 |
| 63459 | Chenopodium quinoa T05764 |
| 2029983 | Chitinophaga caeni T05360 |
| 485918 | Chitinophaga pinensis DSM 2588 T00959 |
| 218497 | Chlamydia abortus S26 3 T00242 |
| 83555 | Chlamydia abortus T03773 |
| 1229831 | Chlamydia avium 10DC88 T03069 |
| 227941 | Chlamydia caviae GPIC T00125 |
| 264202 | Chlamydia felis Fe C 56 T00332 |
| 1143323 | Chlamydia gallinacea 08 1274 3 T04276 |
| 1460373 | Chlamydia muridarum str Nigg 2 MCR T03206 |
| 1434773 | Chlamydia muridarum str Nigg CM972 T03671 |
| 243161 | Chlamydia muridarum str Nigg T00028 |
| 1434770 | Chlamydia muridarum str Nigg3 CMUT3 5 T03684 |
| 83560 | Chlamydia muridarum T03404 |
| 83560 | Chlamydia muridarum T03405 |
| 83560 | Chlamydia muridarum T03505 |
| 331635 | Chlamydia pecorum E58 T01466 |
| 1234369 | Chlamydia pecorum P787 T02853 |
| 1234367 | Chlamydia pecorum PV3056 3 T02854 |
| 1234368 | Chlamydia pecorum W73 T02855 |
| 115711 | Chlamydia pneumoniae AR39 T00029 |
| 115713 | Chlamydia pneumoniae CWL029 T00021 |
| 138677 | Chlamydia pneumoniae J138 T00032 |
| 406984 | Chlamydia pneumoniae LPCoLN T01859 |
| 182082 | Chlamydia pneumoniae TW 183 T00136 |
| 1112252 | Chlamydia psittaci 01DC11 T01860 |
| 1221877 | Chlamydia psittaci 01DC12 T02343 |
| 1112254 | Chlamydia psittaci 02DC15 T01861 |
| 1112267 | Chlamydia psittaci 08DC60 T01862 |
| 331636 | Chlamydia psittaci 6BC T01481 |
| 331636 | Chlamydia psittaci 6BC T02097 |
| 1218176 | Chlamydia psittaci 84 55 T02308 |
| 1112250 | Chlamydia psittaci C19 98 T01863 |
| 1050219 | Chlamydia psittaci CP3 T02251 |
| 1218353 | Chlamydia psittaci GR9 T02309 |
| 1218357 | Chlamydia psittaci M56 T02310 |
| 500464 | Chlamydia psittaci Mat116 T02450 |
| 1218354 | Chlamydia psittaci MN T02311 |
| 1050221 | Chlamydia psittaci NJ1 T02252 |
| 929557 | Chlamydia psittaci RD1 T02153 |
| 1218355 | Chlamydia psittaci VS225 T02312 |
| 1218358 | Chlamydia psittaci WC T02313 |
| 1218356 | Chlamydia psittaci WS RT E30 T02314 |
| 1967783 | Chlamydia sp S15 834C T05716 |
| 471472 | Chlamydia trachomatis 434 Bu T00645 |
| 1071753 | Chlamydia trachomatis A 363 T02562 |
| 1071754 | Chlamydia trachomatis A 5291 T02563 |
| 1071755 | Chlamydia trachomatis A 7249 T02564 |
| 315277 | Chlamydia trachomatis A HAR 13 T00280 |
| 580047 | Chlamydia trachomatis A2497 T01707 |
| 580047 | Chlamydia trachomatis A2497 T02094 |
| 580049 | Chlamydia trachomatis B Jali20 OT T00960 |
| 672161 | Chlamydia trachomatis B TZ1A828 OT T01327 |
| 1431547 | Chlamydia trachomatis C TW 3 T02966 |
| 759363 | Chlamydia trachomatis D EC T01900 |
| 759364 | Chlamydia trachomatis D LC T01901 |
| 1071756 | Chlamydia trachomatis D SotonD1 T02551 |
| 1071757 | Chlamydia trachomatis D SotonD5 T02560 |
| 1071758 | Chlamydia trachomatis D SotonD6 T02561 |
| 272561 | Chlamydia trachomatis D UW 3 CX T00017 |
| 707183 | Chlamydia trachomatis E 11023 T01902 |
| 707184 | Chlamydia trachomatis E 150 T01903 |
| 596777 | Chlamydia trachomatis E Bour T02547 |
| 1100832 | Chlamydia trachomatis E C599 T03168 |
| 1071760 | Chlamydia trachomatis E SotonE4 T02555 |
| 1071761 | Chlamydia trachomatis E SotonE8 T02556 |
| 1071759 | Chlamydia trachomatis E SW3 T02164 |
| 1340853 | Chlamydia trachomatis F 11 96 T02805 |
| 1071764 | Chlamydia trachomatis F SotonF3 T02552 |
| 1071762 | Chlamydia trachomatis F SW4 T02165 |
| 1071763 | Chlamydia trachomatis F SW5 T02166 |
| 1100833 | Chlamydia trachomatis F SWFPminus T03169 |
| 707187 | Chlamydia trachomatis G 11074 T01904 |
| 707186 | Chlamydia trachomatis G 11222 T01905 |
| 718219 | Chlamydia trachomatis G 9301 T01906 |
| 707185 | Chlamydia trachomatis G 9768 T01907 |
| 1071765 | Chlamydia trachomatis G SotonG1 T02553 |
| 1071766 | Chlamydia trachomatis Ia SotonIa1 T02565 |
| 1071767 | Chlamydia trachomatis Ia SotonIa3 T02566 |
| 1260222 | Chlamydia trachomatis IU824 T02493 |
| 1260223 | Chlamydia trachomatis IU888 T02540 |
| 907272 | Chlamydia trachomatis J 6276tet1 T02762 |
| 1071768 | Chlamydia trachomatis K SotonK1 T02546 |
| 1071770 | Chlamydia trachomatis L1 115 T02569 |
| 1075087 | Chlamydia trachomatis L1 1322 p2 T02557 |
| 1071771 | Chlamydia trachomatis L1 224 T02570 |
| 1071769 | Chlamydia trachomatis L1 440 LN T02571 |
| 1071772 | Chlamydia trachomatis L2 25667R T02559 |
| 1262673 | Chlamydia trachomatis L2 434 Bu T02621 |
| 1263406 | Chlamydia trachomatis L2 434 Bu T02622 |
| 1071777 | Chlamydia trachomatis L2b 795 T02572 |
| 1071773 | Chlamydia trachomatis L2b 8200 07 T02573 |
| 1071778 | Chlamydia trachomatis L2b Ams1 T02558 |
| 1071779 | Chlamydia trachomatis L2b Ams2 T02574 |
| 1071780 | Chlamydia trachomatis L2b Ams3 T02575 |
| 1071781 | Chlamydia trachomatis L2b Ams4 T02576 |
| 1071782 | Chlamydia trachomatis L2b Ams5 T02577 |
| 1075085 | Chlamydia trachomatis L2b Canada1 T02567 |
| 1075086 | Chlamydia trachomatis L2b Canada2 T02568 |
| 1071776 | Chlamydia trachomatis L2b CV204 T02554 |
| 1071775 | Chlamydia trachomatis L2b LST T02549 |
| 471473 | Chlamydia trachomatis L2b UCH 1 proctitis T00646 |
| 1071774 | Chlamydia trachomatis L2b UCH 2 T02548 |
| 887712 | Chlamydia trachomatis L2c T01574 |
| 1071783 | Chlamydia trachomatis L3 404 LN T02550 |
| 907269 | Chlamydia trachomatis RC F 342 T02764 |
| 907263 | Chlamydia trachomatis RC F 69 T02763 |
| 907265 | Chlamydia trachomatis RC F 852 T02765 |
| 907270 | Chlamydia trachomatis RC J 122 T02770 |
| 907266 | Chlamydia trachomatis RC J 943 T02766 |
| 907267 | Chlamydia trachomatis RC J 953 T02767 |
| 907271 | Chlamydia trachomatis RC J 966 T02768 |
| 1007871 | Chlamydia trachomatis RC J 971 T02769 |
| 907268 | Chlamydia trachomatis RC L2 3 T02772 |
| 907264 | Chlamydia trachomatis RC L2 46 T02773 |
| 1007870 | Chlamydia trachomatis RC L2 55 T02771 |
| 634464 | Chlamydia trachomatis Sweden2 T01966 |
| 3055 | Chlamydomonas reinhardtii T01039 |
| 981222 | Chloracidobacterium thermophilum B T01629 |
| 554065 | Chlorella variabilis T02924 |
| 274537 | Chlorobaculum limnaeum T04492 |
| 517417 | Chlorobaculum parvum NCIB 8327 T00725 |
| 194439 | Chlorobaculum tepidum TLS T00088 |
| 340177 | Chlorobium chlorochromatii CaD3 T00290 |
| 290315 | Chlorobium limicola DSM 245 T00766 |
| 331678 | Chlorobium phaeobacteroides BS1 T00709 |
| 290317 | Chlorobium phaeobacteroides DSM 266 T00439 |
| 290318 | Chlorobium phaeovibrioides DSM 265 T00499 |
| 60711 | Chlorocebus sabaeus T04361 |
| 326427 | Chloroflexus aggregans DSM 9485 T00824 |
| 324602 | Chloroflexus aurantiacus J 10 fl T00639 |
| 480224 | Chloroflexus aurantiacus Y 400 fl T00859 |
| 517418 | Chloroherpeton thalassium ATCC 35110 T00726 |
| 2005460 | Chondrocystis sp NIES 4102 T05085 |
| 52 | Chondromyces crocatus T04010 |
| 2769 | Chondrus crispus T02916 |
| 626937 | Christensenella minuta T05665 |
| 1778675 | Chromobacterium rhizoryzae T05688 |
| 2059672 | Chromobacterium sp ATCC 53434 T05234 |
| 2202141 | Chromobacterium sp IIBBL 112 1 T05549 |
| 2202141 | Chromobacterium sp IIBBL 274 1 T05744 |
| 1108595 | Chromobacterium vaccinii T04565 |
| 243365 | Chromobacterium violaceum ATCC 12472 T00147 |
| 290398 | Chromohalobacter salexigens DSM 3043 T00347 |
| 251229 | Chroococcidiopsis thermalis PCC 7203 T02360 |
| 8478 | Chrysemys picta bellii T04662 |
| 651561 | Chryseobacterium arthrosphaerae T05746 |
| 1324352 | Chryseobacterium gallinarum T03954 |
| 1685010 | Chryseobacterium glaciei T04575 |
| 253 | Chryseobacterium indologenes T04932 |
| 558152 | Chryseobacterium piperi T05071 |
| 2478663 | Chryseobacterium sp 3008163 T05679 |
| 2039166 | Chryseobacterium sp 6424 T05704 |
| 1721091 | Chryseobacterium sp IHB B 17019 T04193 |
| 878220 | Chryseobacterium sp StRB126 T03602 |
| 2015076 | Chryseobacterium sp T16E 39 T04952 |
| 536441 | Chryseobacterium taklimakanense T05512 |
| 2321403 | Chryseolinea soli T05641 |
| 1303518 | Chthonomonas calidirosea T49 T02699 |
| 3827 | Cicer arietinum T02819 |
| 79782 | Cimex lectularius T05603 |
| 7719 | Ciona intestinalis T01056 |
| 35703 | Citrobacter amalonaticus T04738 |
| 1261127 | Citrobacter amalonaticus Y19 T03934 |
| 57706 | Citrobacter braakii T04825 |
| 67824 | Citrobacter farmeri T05012 |
| 1333848 | Citrobacter freundii CFNIH1 T03077 |
| 290338 | Citrobacter koseri ATCC BAA 895 T00592 |
| 1563222 | Citrobacter pasteurii T05874 |
| 637910 | Citrobacter rodentium ICC168 T01145 |
| 1920110 | Citrobacter sp CFNIH10 T05324 |
| 1703250 | Citrobacter sp CRE 46 T05520 |
| 1702170 | Citrobacter sp FDAARGOS 156 T04313 |
| 67827 | Citrobacter werkmanii T05129 |
| 133448 | Citrobacter youngae T05526 |
| 1634516 | Citromicrobium sp JL477 T04089 |
| 85681 | Citrus clementina T02982 |
| 2711 | Citrus sinensis T02983 |
| 1874630 | Clavibacter michiganensis subsp capsici T05227 |
| 33014 | Clavibacter michiganensis subsp insidiosus T03862 |
| 443906 | Clavibacter michiganensis subsp michiganensis NCPPB 382 T00529 |
| 1097677 | Clavibacter michiganensis subsp nebraskensis NCPPB 2581 T02530 |
| 31964 | Clavibacter michiganensis subsp sepedonicus T00678 |
| 306902 | Clavispora lusitaniae ATCC 42720 T01149 |
| 36911 | Clavispora lusitaniae T05147 |
| 1197717 | Cloacibacillus porcorum T04453 |
| 237258 | Cloacibacterium normanense T05730 |
| 272563 | Clostridioides difficile 630 T00487 |
| 272563 | Clostridioides difficile 630 T03885 |
| 645462 | Clostridioides difficile CD196 T00997 |
| 645463 | Clostridioides difficile R20291 T00998 |
| 1496 | Clostridioides difficile T03785 |
| 84022 | Clostridium aceticum T03971 |
| 272562 | Clostridium acetobutylicum ATCC 824 T00056 |
| 991791 | Clostridium acetobutylicum DSM 1731 T01548 |
| 863638 | Clostridium acetobutylicum EA 2018 T01864 |
| 29341 | Clostridium argentinense T05123 |
| 1341692 | Clostridium autoethanogenum DSM 10061 T02883 |
| 1415775 | Clostridium baratii str Sullivan T03506 |
| 864803 | Clostridium beijerinckii ATCC 35702 T03415 |
| 290402 | Clostridium beijerinckii NCIMB 8052 T00547 |
| 1520 | Clostridium beijerinckii T04773 |
| 1216932 | Clostridium bornimense T03165 |
| 441770 | Clostridium botulinum A str ATCC 19397 T00574 |
| 413999 | Clostridium botulinum A str ATCC 3502 T00533 |
| 441771 | Clostridium botulinum A str Hall T00575 |
| 536232 | Clostridium botulinum A2 str Kyoto T00883 |
| 498214 | Clostridium botulinum A3 str Loch Maree T00670 |
| 935198 | Clostridium botulinum B str Eklund 17B T00696 |
| 498213 | Clostridium botulinum B1 str Okra T00671 |
| 515621 | Clostridium botulinum Ba4 str 657 T00895 |
| 929506 | Clostridium botulinum BKT015925 T01475 |
| 508767 | Clostridium botulinum E3 str Alaska E43 T00767 |
| 758678 | Clostridium botulinum F str 230613 T01865 |
| 441772 | Clostridium botulinum F str Langeland T00563 |
| 941968 | Clostridium botulinum H04402 065 T02076 |
| 1492 | Clostridium butyricum T04197 |
| 536227 | Clostridium carboxidivorans P7 T03979 |
| 29343 | Clostridium cellulosi T03806 |
| 573061 | Clostridium cellulovorans 743B T01294 |
| 717608 | Clostridium cf saccharolyticum K10 T02614 |
| 46867 | Clostridium chauvoei T05083 |
| 332101 | Clostridium drakei T05484 |
| 1552 | Clostridium estertheticum subsp estertheticum T04811 |
| 1497 | Clostridium formicaceticum T05005 |
| 182773 | Clostridium isatidis T05663 |
| 431943 | Clostridium kluyveri DSM 555 T00564 |
| 583346 | Clostridium kluyveri NBRC 12016 T00832 |
| 748727 | Clostridium ljungdahlii DSM 13528 T01276 |
| 386415 | Clostridium novyi NT T00431 |
| 86416 | Clostridium pasteurianum BC1 T02645 |
| 1262449 | Clostridium pasteurianum DSM 525 ATCC 6013 T03753 |
| 1262449 | Clostridium pasteurianum DSM 525 ATCC 6013 T03811 |
| 195103 | Clostridium perfringens ATCC 13124 T00378 |
| 289380 | Clostridium perfringens SM101 T00379 |
| 195102 | Clostridium perfringens str 13 T00074 |
| 1345695 | Clostridium saccharobutylicum DSM 13864 T02876 |
| 610130 | Clostridium saccharolyticum WM1 T01288 |
| 931276 | Clostridium saccharoperbutylacetonicum N1 4 T02467 |
| 1548 | Clostridium scatologenes T03803 |
| 755731 | Clostridium sp BNL1100 T01708 |
| 1042156 | Clostridium sp SY8519 T01565 |
| 1509 | Clostridium sporogenes T03905 |
| 394958 | Clostridium taeniosporum T04957 |
| 1231072 | Clostridium tetani 12124569 T02905 |
| 212717 | Clostridium tetani E88 T00112 |
| 1519 | Clostridium tyrobutyricum T04331 |
| 1619308 | Cnuibacter physcomitrellae T04797 |
| 28258 | Cobetia marina T04789 |
| 246410 | Coccidioides immitis RS T01114 |
| 222929 | Coccidioides posadasii C735 delta SOWgp T01332 |
| 574566 | Coccomyxa subellipsoidea C 169 T02914 |
| 2674991 | Cohnella candidum T05696 |
| 1445577 | Colletotrichum fioriniae PJ7 T04796 |
| 279058 | Collimonas arenae T03512 |
| 1005048 | Collimonas fungivorans Ter331 T01583 |
| 279113 | Collimonas pratensis T04306 |
| 74426 | Collinsella aerofaciens T05143 |
| 8932 | Columba livia T02912 |
| 1967665 | Colwellia beringensis T05628 |
| 167879 | Colwellia psychrerythraea 34H T00258 |
| 2161872 | Colwellia sp Arc7 D T05422 |
| 58049 | Colwellia sp MT41 T04159 |
| 1816218 | Colwellia sp PAMC 20917 T04511 |
| 1816219 | Colwellia sp PAMC 21821 T04816 |
| 225992 | Comamonas kerstersii T04758 |
| 1082851 | Comamonas serinivorans T04892 |
| 1392005 | Comamonas testosteroni TK102 T03218 |
| 363952 | Comamonas thiooxydans T01109 |
| 2070537 | Commensalibacter sp AMU001 T05684 |
| 469383 | Conexibacter woesei DSM 14684 T01153 |
| 1609966 | Confluentimicrobium sp EMB200 NS6 T04096 |
| 741705 | Coniophora puteana RWD 64 598 SS2 T03134 |
| 240176 | Coprinopsis cinerea okayama7 130 T01275 |
| 717962 | Coprococcus catus GD 7 T02584 |
| 751585 | Coprococcus sp ART55 1 T02583 |
| 309798 | Coprothermobacter proteolyticus DSM 5265 T00773 |
| 583355 | Coraliomargarita akajimensis DSM 45221 T01204 |
| 1144275 | Corallococcus coralloides DSM 2259 T01780 |
| 983644 | Cordyceps militaris CM01 T03108 |
| 1531429 | Coriobacteriaceae bacterium 68 1 3 T03545 |
| 700015 | Coriobacterium glomerans PW2 T01453 |
| 932674 | Corvus cornix cornix T03546 |
| 649754 | Corynebacterium ammoniagenes DSM 20306 T05164 |
| 1431546 | Corynebacterium aquilae DSM 44791 T05112 |
| 1348662 | Corynebacterium argentoratense DSM 44202 T02821 |
| 191610 | Corynebacterium atypicum T03227 |
| 548476 | Corynebacterium aurimucosum ATCC 700975 T00887 |
| 1121353 | Corynebacterium callunae DSM 20147 T02507 |
| 161896 | Corynebacterium camporealensis T03923 |
| 1285583 | Corynebacterium casei LMG S 19264 T03041 |
| 1652495 | Corynebacterium crudilactis T04829 |
| 931089 | Corynebacterium deserti GIMN1 010 T04068 |
| 698966 | Corynebacterium diphtheriae 241 T01687 |
| 698962 | Corynebacterium diphtheriae 31A T01697 |
| 698973 | Corynebacterium diphtheriae BH8 T01698 |
| 698963 | Corynebacterium diphtheriae C7 T01699 |
| 698965 | Corynebacterium diphtheriae CDCE 8392 T01700 |
| 698967 | Corynebacterium diphtheriae HC01 T01693 |
| 698968 | Corynebacterium diphtheriae HC02 T01694 |
| 698969 | Corynebacterium diphtheriae HC03 T01695 |
| 698970 | Corynebacterium diphtheriae HC04 T01696 |
| 698972 | Corynebacterium diphtheriae INCA 402 T01688 |
| 257309 | Corynebacterium diphtheriae NCTC 13129 T00151 |
| 698964 | Corynebacterium diphtheriae PW8 T01701 |
| 1717 | Corynebacterium diphtheriae T04861 |
| 698971 | Corynebacterium diphtheriae VA01 T01702 |
| 558173 | Corynebacterium doosanense CAU 212 DSM 45436 T03413 |
| 196164 | Corynebacterium efficiens YS 314 T00103 |
| 1050174 | Corynebacterium epidermidicanis T03955 |
| 1451189 | Corynebacterium falsenii DSM 44353 T03037 |
| 28028 | Corynebacterium flavescens T05018 |
| 1437875 | Corynebacterium frankenforstense DSM 45800 T04872 |
| 187491 | Corynebacterium glaucum T05058 |
| 196627 | Corynebacterium glutamicum ATCC 13032 T00102 |
| 196627 | Corynebacterium glutamicum ATCC 13032 T00244 |
| 1204414 | Corynebacterium glutamicum K051 T02508 |
| 1310161 | Corynebacterium glutamicum MB001 T02791 |
| 340322 | Corynebacterium glutamicum R T00501 |
| 1232381 | Corynebacterium glutamicum SCgG1 T02690 |
| 1232383 | Corynebacterium glutamicum SCgG2 T02691 |
| 1718 | Corynebacterium glutamicum T03361 |
| 1718 | Corynebacterium glutamicum T03362 |
| 1718 | Corynebacterium glutamicum T03668 |
| 1404245 | Corynebacterium glyciniphilum AJ 3170 T03073 |
| 1121362 | Corynebacterium halotolerans YIM 70093 DSM 44683 T02472 |
| 1223515 | Corynebacterium humireducens NBRC 106098 DSM 45392 T03576 |
| 156978 | Corynebacterium imitans T03237 |
| 306537 | Corynebacterium jeikeium K411 T00255 |
| 645127 | Corynebacterium kroppenstedtii DSM 44385 T00896 |
| 35755 | Corynebacterium kutscheri T03866 |
| 1408189 | Corynebacterium lactis RW2 5 T04042 |
| 1224162 | Corynebacterium marinum DSM 44953 T03633 |
| 1224163 | Corynebacterium maris DSM 45190 T02784 |
| 38301 | Corynebacterium minutissimum T05574 |
| 571915 | Corynebacterium mustelae T03941 |
| 161895 | Corynebacterium phocae T05007 |
| 1087454 | Corynebacterium pseudotuberculosis 1 06 A T02052 |
| 679896 | Corynebacterium pseudotuberculosis 1002 T01876 |
| 1168865 | Corynebacterium pseudotuberculosis 258 T02118 |
| 1089446 | Corynebacterium pseudotuberculosis 267 T01934 |
| 1087452 | Corynebacterium pseudotuberculosis 3 99 5 T01689 |
| 1087451 | Corynebacterium pseudotuberculosis 31 T02029 |
| 1074485 | Corynebacterium pseudotuberculosis 316 T01753 |
| 1087453 | Corynebacterium pseudotuberculosis 42 02 A T02053 |
| 681645 | Corynebacterium pseudotuberculosis C231 T01877 |
| 935697 | Corynebacterium pseudotuberculosis CIP 52 97 T02054 |
| 1161911 | Corynebacterium pseudotuberculosis Cp162 T02154 |
| 765874 | Corynebacterium pseudotuberculosis FRC41 T01277 |
| 889513 | Corynebacterium pseudotuberculosis I19 T01878 |
| 1117942 | Corynebacterium pseudotuberculosis P54B96 T01771 |
| 935298 | Corynebacterium pseudotuberculosis PAT10 T01879 |
| 1719 | Corynebacterium pseudotuberculosis T03482 |
| 1719 | Corynebacterium pseudotuberculosis T03483 |
| 1719 | Corynebacterium pseudotuberculosis T03484 |
| 662755 | Corynebacterium resistens DSM 45100 T01538 |
| 146827 | Corynebacterium simulans T04316 |
| 161899 | Corynebacterium singulare T03630 |
| 1487956 | Corynebacterium sp ATCC 6931 T03283 |
| 1437874 | Corynebacterium sphenisci DSM 44792 T05122 |
| 1705 | Corynebacterium stationis T04724 |
| 43770 | Corynebacterium striatum T05059 |
| 1200352 | Corynebacterium terpenotabidum Y 11 T02714 |
| 136857 | Corynebacterium testudinoris T03956 |
| 996634 | Corynebacterium ulcerans 0102 T02171 |
| 945711 | Corynebacterium ulcerans 809 T01880 |
| 945712 | Corynebacterium ulcerans BR AD22 T01549 |
| 1006579 | Corynebacterium ulcerans FRC11 T03408 |
| 65058 | Corynebacterium ulcerans T03406 |
| 65058 | Corynebacterium ulcerans T03414 |
| 65058 | Corynebacterium ulcerans T03420 |
| 65058 | Corynebacterium ulcerans T03899 |
| 504474 | Corynebacterium urealyticum DSM 7109 T00685 |
| 1267754 | Corynebacterium urealyticum DSM 7111 T02457 |
| 401472 | Corynebacterium ureicelerivorans T03241 |
| 1072256 | Corynebacterium uterequi T03957 |
| 858619 | Corynebacterium variabile DSM 44702 T01594 |
| 1224164 | Corynebacterium vitaeruminis DSM 20294 T03042 |
| 93934 | Coturnix japonica T04297 |
| 434923 | Coxiella burnetii CbuG Q212 T00787 |
| 434924 | Coxiella burnetii CbuK Q154 T00788 |
| 434922 | Coxiella burnetii Dugway 5J108 111 T00583 |
| 360115 | Coxiella burnetii RSA 331 T00632 |
| 227377 | Coxiella burnetii RSA 493 T00124 |
| 325775 | Coxiella endosymbiont of Amblyomma americanum T03533 |
| 29159 | Crassostrea gigas T03920 |
| 10029 | Cricetulus griseus T02813 |
| 1173022 | Crinalium epipsammum PCC 9333 T02373 |
| 216432 | Croceibacter atlanticus HTCC2559 T01256 |
| 450378 | Croceicoccus marinus T04890 |
| 1348774 | Croceicoccus naphthovorans T03964 |
| 43989 | Crocosphaera subtropica ATCC 51142 T00686 |
| 165597 | Crocosphaera watsonii WH 8501 T05097 |
| 1073999 | Cronobacter condimenti 1330 T04060 |
| 1159554 | Cronobacter dublinensis subsp dublinensis LMG 23823 T04061 |
| 1159491 | Cronobacter malonaticus LMG 23826 T04062 |
| 413503 | Cronobacter malonaticus T02955 |
| 1159613 | Cronobacter muytjensii ATCC 51329 T04067 |
| 290339 | Cronobacter sakazakii ATCC BAA 894 T00586 |
| 1138308 | Cronobacter sakazakii ES15 T02119 |
| 956149 | Cronobacter sakazakii SP291 T02461 |
| 28141 | Cronobacter sakazakii T03903 |
| 693216 | Cronobacter turicensis z3032 T00999 |
| 1074000 | Cronobacter universalis NCTC 9529 T04063 |
| 670052 | Cryobacterium arcticum T04435 |
| 1978566 | Cryobacterium sp LW097 T04908 |
| 469378 | Cryptobacterium curtum DSM 15641 T00962 |
| 367775 | Cryptococcus gattii WM276 T02232 |
| 283643 | Cryptococcus neoformans var neoformans B 3501A T01040 |
| 214684 | Cryptococcus neoformans var neoformans JEC21 T00243 |
| 353151 | Cryptosporidium hominis TU502 T00210 |
| 353152 | Cryptosporidium parvum Iowa II T00168 |
| 3656 | Cucumis melo T03195 |
| 3659 | Cucumis sativus T02486 |
| 3661 | Cucurbita maxima T05246 |
| 3662 | Cucurbita moschata T05248 |
| 3664 | Cucurbita pepo subsp pepo T05351 |
| 7176 | Culex quinquefasciatus T01079 |
| 1673428 | Cuniculiplasma divulgatum T04682 |
| 68895 | Cupriavidus basilensis T03642 |
| 1267562 | Cupriavidus gilardii CR3 T04050 |
| 367825 | Cupriavidus malaysiensis T04978 |
| 266264 | Cupriavidus metallidurans CH34 T00351 |
| 381666 | Cupriavidus necator H16 T00416 |
| 1042878 | Cupriavidus necator N 1 T01564 |
| 106590 | Cupriavidus necator T05121 |
| 82633 | Cupriavidus pauculus T05738 |
| 264198 | Cupriavidus pinatubonensis JMP134 T00268 |
| 876364 | Cupriavidus sp USMAA2 4 T05072 |
| 1389192 | Cupriavidus sp USMAHM13 T04762 |
| 977880 | Cupriavidus taiwanensis LMG 19424 T00702 |
| 1905847 | Curtobacterium sp BH 2 1 1 T04562 |
| 1561023 | Curtobacterium sp MR MD2014 T04180 |
| 909952 | Cutibacterium acnes 266 T01971 |
| 1031709 | Cutibacterium acnes 6609 T02065 |
| 1234380 | Cutibacterium acnes C1 T02330 |
| 1276648 | Cutibacterium acnes hdn 1 T03470 |
| 1134454 | Cutibacterium acnes HL096PA1 T02638 |
| 267747 | Cutibacterium acnes KPA171202 T00188 |
| 553199 | Cutibacterium acnes SK137 T01215 |
| 1091045 | Cutibacterium acnes subsp defendens ATCC 11828 T01972 |
| 1747 | Cutibacterium acnes T04624 |
| 1114967 | Cutibacterium acnes TypeIA2 P acn17 T01654 |
| 1114969 | Cutibacterium acnes TypeIA2 P acn31 T01655 |
| 1114966 | Cutibacterium acnes TypeIA2 P acn33 T01656 |
| 1170318 | Cutibacterium avidum 44067 T02632 |
| 33011 | Cutibacterium granulosum T05425 |
| 280699 | Cyanidioschyzon merolae strain 10D T00175 |
| 156563 | Cyanistes caeruleus T05602 |
| 755178 | Cyanobacterium aponinum PCC 10605 T02376 |
| 1228987 | Cyanobacterium endosymbiont of Epithemia turgida isolate EtSB Lake Yunoko T03622 |
| 1763363 | Cyanobacterium endosymbiont of Rhopalodia gibberula T05747 |
| 2054282 | Cyanobacterium sp HL 69 T05309 |
| 292563 | Cyanobacterium stanieri PCC 7202 T02377 |
| 292564 | Cyanobium gracile PCC 6307 T02359 |
| 1851505 | Cyanobium sp NIES 981 T04888 |
| 395961 | Cyanothece sp PCC 7425 T00836 |
| 320787 | Cyclobacterium amurskyense T03993 |
| 880070 | Cyclobacterium marinum DSM 745 T01592 |
| 385025 | Cycloclasticus sp P1 T02265 |
| 728003 | Cycloclasticus sp PY97N T05126 |
| 1198232 | Cycloclasticus zancles 78 ME T02780 |
| 56107 | Cylindrospermum stagnale PCC 7417 T02398 |
| 59895 | Cynara cardunculus var scolymus T05604 |
| 244447 | Cynoglossus semilaevis T04866 |
| 7962 | Cyprinus carpio T05134 |
| 43 | Cystobacter fuscus T05065 |
| 269798 | Cytophaga hutchinsonii ATCC 33406 T00385 |
| 13035 | Dactylococcopsis salina PCC 8305 T02378 |
| 278856 | Danaus plexippus plexippus T04344 |
| 7955 | Danio rerio T01004 |
| 6669 | Daphnia pulex T04347 |
| 79200 | Daucus carota subsp sativus T05350 |
| 284592 | Debaryomyces hansenii CBS767 T01026 |
| 159087 | Dechloromonas aromatica RCB T00259 |
| 2231055 | Dechloromonas sp HYN0024 T05616 |
| 639282 | Deferribacter desulfuricans SSM1 T01188 |
| 1335048 | Defluviimonas alba T04387 |
| 1006576 | Defluviitoga tunisiensis T03867 |
| 871738 | Dehalobacter restrictus DSM 9455 T02996 |
| 1131462 | Dehalobacter sp CF T02279 |
| 1147129 | Dehalobacter sp DCA T02278 |
| 243164 | Dehalococcoides mccartyi 195 T00223 |
| 216389 | Dehalococcoides mccartyi BAV1 T00518 |
| 1193806 | Dehalococcoides mccartyi BTF08 T02475 |
| 255470 | Dehalococcoides mccartyi CBDB1 T00273 |
| 1432059 | Dehalococcoides mccartyi CG1 T03647 |
| 1432060 | Dehalococcoides mccartyi CG4 T03648 |
| 1432061 | Dehalococcoides mccartyi CG5 T03649 |
| 1193807 | Dehalococcoides mccartyi DCMB5 T02476 |
| 633145 | Dehalococcoides mccartyi GT T01182 |
| 1388758 | Dehalococcoides mccartyi GY50 T02938 |
| 311424 | Dehalococcoides mccartyi VS T01136 |
| 1522671 | Dehalococcoides sp UCH007 T03880 |
| 1839801 | Dehalogenimonas formicexedens T04654 |
| 552811 | Dehalogenimonas lykanthroporepellens BL DC 9 T01264 |
| 943347 | Dehalogenimonas sp WBC 2 T03938 |
| 1768108 | Deinococcus actinosclerus T04259 |
| 546414 | Deinococcus deserti VCD115 T00880 |
| 317577 | Deinococcus ficus T05674 |
| 319795 | Deinococcus geothermalis DSM 11300 T00353 |
| 745776 | Deinococcus gobiensis I 0 T01827 |
| 2202254 | Deinococcus irradiatisoli T05530 |
| 709986 | Deinococcus maricopensis DSM 21211 T01412 |
| 937777 | Deinococcus peraridilitoris DSM 19664 T02381 |
| 693977 | Deinococcus proteolyticus MRP T01426 |
| 1182568 | Deinococcus puniceus T04339 |
| 243230 | Deinococcus radiodurans R1 T00025 |
| 1309411 | Deinococcus soli Cha et al 2016 T04233 |
| 57497 | Deinococcus swuensis T03508 |
| 980427 | Deinococcus wulumuqiensis T05579 |
| 398578 | Delftia acidovorans SPH 1 T00620 |
| 742013 | Delftia sp Cs1 4 T01497 |
| 1920191 | Delftia sp HK171 T04584 |
| 180282 | Delftia tsuruhatensis T04496 |
| 906689 | Dendrobium catenatum T05027 |
| 77166 | Dendroctonus ponderosae T04899 |
| 79604 | Denitrobacterium detoxificans T04382 |
| 522772 | Denitrovibrio acetiphilus DSM 12809 T01189 |
| 1630135 | Dermabacter vaginalis T04434 |
| 1274 | Dermacoccus nishinomiyaensis T03223 |
| 6956 | Dermatophagoides pteronyssinus T05775 |
| 1863 | Dermatophilus congolensis T05038 |
| 767817 | Desulfallas gibsoniae DSM 7213 T02657 |
| 644282 | Desulfarculus baarsii DSM 2075 T01284 |
| 218208 | Desulfatibacillum alkenivorans AK 01 T00813 |
| 756499 | Desulfitobacterium dehalogenans ATCC 51507 T02146 |
| 871963 | Desulfitobacterium dichloroeliminans LMG P 21439 T02390 |
| 272564 | Desulfitobacterium hafniense DCB 2 T00822 |
| 138119 | Desulfitobacterium hafniense Y51 T00333 |
| 871968 | Desulfitobacterium metallireducens DSM 15288 T03599 |
| 880072 | Desulfobacca acetoxidans DSM 11109 T01454 |
| 177437 | Desulfobacterium autotrophicum HRM2 T00861 |
| 651182 | Desulfobacula toluolica Tol2 T02268 |
| 1986146 | Desulfobulbus oralis T05339 |
| 577650 | Desulfobulbus propionicus DSM 2032 T01413 |
| 1167006 | Desulfocapsa sulfexigens DSM 10523 T02473 |
| 897 | Desulfococcus multivorans T04568 |
| 96561 | Desulfococcus oleovorans Hxd3 T00610 |
| 690850 | Desulfocurvibacter africanus subsp africanus str Walvis Bay T01662 |
| 485916 | Desulfofarcimen acetoxidans DSM 771 T00986 |
| 760568 | Desulfofundulus kuznetsovii DSM 6115 T01499 |
| 485915 | Desulfohalobium retbaense DSM 5692 T00989 |
| 525897 | Desulfomicrobium baculatum DSM 4028 T00963 |
| 888061 | Desulfomicrobium orale DSM 12838 T04289 |
| 706587 | Desulfomonile tiedjei DSM 6799 T02147 |
| 646529 | Desulfosporosinus acidiphilus SJ4 T02148 |
| 768704 | Desulfosporosinus meridiei DSM 13257 T02207 |
| 768706 | Desulfosporosinus orientis DSM 765 T01675 |
| 177439 | Desulfotalea psychrophila LSv54 T00192 |
| 1833852 | Desulfotomaculum ferrireducens T04823 |
| 868595 | Desulfotomaculum nigrificans CO 1 SRB T01498 |
| 349161 | Desulfotomaculum reducens MI 1 T00494 |
| 696281 | Desulfotomaculum ruminis DSM 2154 T01518 |
| 207559 | Desulfovibrio alaskensis G20 T00294 |
| 525146 | Desulfovibrio desulfuricans ATCC 27774 T00837 |
| 641491 | Desulfovibrio desulfuricans ND132 T01709 |
| 44742 | Desulfovibrio fairfieldensis T04293 |
| 241368 | Desulfovibrio ferrophilus T05829 |
| 1121448 | Desulfovibrio gigas DSM 1382 ATCC 19364 T02851 |
| 1121451 | Desulfovibrio hydrothermalis AM13 DSM 14728 T02428 |
| 573370 | Desulfovibrio magneticus RS 1 T00917 |
| 901 | Desulfovibrio piger T04638 |
| 526222 | Desulfovibrio salexigens DSM 2638 T00969 |
| 631220 | Desulfovibrio sp G11 T05183 |
| 391774 | Desulfovibrio vulgaris DP4 T00452 |
| 573059 | Desulfovibrio vulgaris RCH1 T02064 |
| 882 | Desulfovibrio vulgaris str Hildenborough T00171 |
| 883 | Desulfovibrio vulgaris str Miyazaki F T00816 |
| 694431 | Desulfurella acetivorans A63 T03007 |
| 653733 | Desulfurispirillum indicum S5 T01383 |
| 589865 | Desulfurivibrio alkaliphilus AHT 2 T01244 |
| 868864 | Desulfurobacterium thermolithotrophum DSM 11699 T01427 |
| 490899 | Desulfurococcus amylolyticus 1221n T00817 |
| 768672 | Desulfurococcus amylolyticus DSM 16532 T02136 |
| 765177 | Desulfurococcus mucosus DSM 2162 T01406 |
| 1603606 | Desulfuromonas soudanensis T04049 |
| 1823759 | Desulfuromonas sp DDH964 T04368 |
| 1643450 | Devosia sp H5989 T04003 |
| 2083786 | Devosia sp I507 T05346 |
| 39950 | Dialister pneumosintes T04459 |
| 164759 | Diaphorobacter polyhydroxybutyrativorans T04937 |
| 246195 | Dichelobacter nodosus VCS1703A T00517 |
| 732165 | Dichomitus squalens LYAD 421 SS1 T03133 |
| 561229 | Dickeya chrysanthemi Ech1591 T00930 |
| 198628 | Dickeya dadantii 3937 T01305 |
| 1306405 | Dickeya dianthicola RNS04 9 T05141 |
| 1778540 | Dickeya fangzhongdai T05277 |
| 579405 | Dickeya paradisiaca Ech703 T00927 |
| 1225786 | Dickeya solani IPO 2222 T04410 |
| 1778540 | Dickeya solani T03347 |
| 1427366 | Dickeya zeae EC1 T03567 |
| 590409 | Dickeya zeae Ech586 T01138 |
| 309799 | Dictyoglomus thermophilum H 6 12 T00774 |
| 515635 | Dictyoglomus turgidum DSM 6724 T00800 |
| 352472 | Dictyostelium discoideum AX4 T00245 |
| 5786 | Dictyostelium purpureum T02090 |
| 546160 | Dietzia lutea T05442 |
| 139021 | Dietzia psychralcaliphila T05437 |
| 2052657 | Dietzia sp JS16 p6b T05397 |
| 712270 | Dietzia sp oral taxon 368 T05390 |
| 499555 | Dietzia timorensis T04608 |
| 609295 | Dinoponera quadriceps T05610 |
| 398580 | Dinoroseobacter shibae DFL 12 DSM 16493 T00612 |
| 143948 | Diuraphis noxia T04900 |
| 1300342 | Dokdonella koreensis DS 123 T04377 |
| 1300343 | Dokdonia donghaensis DSW 1 T04309 |
| 983548 | Dokdonia sp 4H 3 7 5 T01484 |
| 2173169 | Dokdonia sp Dokd P16 T05432 |
| 313590 | Dokdonia sp MED134 T03275 |
| 1168034 | Draconibacterium orientale T03072 |
| 7217 | Drosophila ananassae T01059 |
| 7220 | Drosophila erecta T01060 |
| 7222 | Drosophila grimshawi T01061 |
| 7227 | Drosophila melanogaster T00030 |
| 7230 | Drosophila mojavensis T01062 |
| 7234 | Drosophila persimilis T01063 |
| 7237 | Drosophila pseudoobscura pseudoobscura T01032 |
| 7238 | Drosophila sechellia T01064 |
| 7240 | Drosophila simulans T01065 |
| 7244 | Drosophila virilis T01066 |
| 7260 | Drosophila willistoni T01067 |
| 7245 | Drosophila yakuba T01068 |
| 66656 | Durio zibethinus T05242 |
| 471854 | Dyadobacter fermentans DSM 18053 T00970 |
| 1217721 | Dyella japonica A8 T03215 |
| 1379159 | Dyella jiangningensis T03074 |
| 2501295 | Dyella sp M7H15 1 T05846 |
| 445710 | Dyella thiooxydans T04308 |
| 1795355 | Echinicola strongylocentroti T05506 |
| 926556 | Echinicola vietnamensis DSM 17526 T02388 |
| 6210 | Echinococcus granulosus T05553 |
| 1442136 | Ectothiorhodospira sp BSL 9 T04374 |
| 667120 | Edwardsiella anguillarum ET080813 T03245 |
| 93378 | Edwardsiella hoshinae T04508 |
| 634503 | Edwardsiella ictaluri 93 146 T00912 |
| 1288122 | Edwardsiella piscicida C07 087 T02509 |
| 1578828 | Edwardsiella sp EA181011 T03991 |
| 1650654 | Edwardsiella sp LADL05 105 T04005 |
| 1263550 | Edwardsiella tarda EIB202 T01121 |
| 718251 | Edwardsiella tarda FL6 60 T02091 |
| 479437 | Eggerthella lenta DSM 2243 T00984 |
| 502558 | Eggerthella sp YY7918 T01566 |
| 1670831 | Egibacter rhizosphaerae T05860 |
| 188379 | Egretta garzetta T05152 |
| 269484 | Ehrlichia canis str Jake T00270 |
| 205920 | Ehrlichia chaffeensis str Arkansas T00328 |
| 1249651 | Ehrlichia chaffeensis str Heartland T03085 |
| 1249652 | Ehrlichia chaffeensis str Jax T03189 |
| 1249653 | Ehrlichia chaffeensis str Liberty T03190 |
| 1249654 | Ehrlichia chaffeensis str Osceola T03191 |
| 1249655 | Ehrlichia chaffeensis str Saint Vincent T03348 |
| 1249656 | Ehrlichia chaffeensis str Wakulla T03364 |
| 1249650 | Ehrlichia chaffeensis str West Paces T03365 |
| 1423892 | Ehrlichia muris AS145 T02967 |
| 302409 | Ehrlichia ruminantium str Gardel T00232 |
| 254945 | Ehrlichia ruminantium str Welgevonden T00224 |
| 254945 | Ehrlichia ruminantium str Welgevonden T00233 |
| 391036 | Ehrlichia sp HF T03078 |
| 539 | Eikenella corrodens T05039 |
| 51953 | Elaeis guineensis T03921 |
| 8005 | Electrophorus electricus T05787 |
| 1352940 | Elizabethkingia anophelis FMS 007 T03946 |
| 1338011 | Elizabethkingia anophelis NUHP1 T03235 |
| 1117645 | Elizabethkingia anophelis T04743 |
| 1756149 | Elizabethkingia bruuniana T05037 |
| 238 | Elizabethkingia meningoseptica T04744 |
| 172045 | Elizabethkingia miricola T03757 |
| 1756150 | Elizabethkingia ursingii T05036 |
| 445932 | Elusimicrobium minutum Pei191 T00741 |
| 280463 | Emiliania huxleyi CCMP1516 T02915 |
| 929562 | Emticicia oligotrophica DSM 17448 T02272 |
| 284813 | Encephalitozoon cuniculi GB M1 T00105 |
| 907965 | Encephalitozoon hellem ATCC 50504 T02287 |
| 876142 | Encephalitozoon intestinalis ATCC 50506 T02238 |
| 1178016 | Encephalitozoon romaleae SJ 2008 T03458 |
| 1408281 | Endomicrobium proavitum T03965 |
| 86106 | Endosymbiont of Acanthamoeba sp UWC8 T03255 |
| 1303921 | Endosymbiont of Bathymodiolus septemdierum str Myojin knoll T04606 |
| 1248727 | Endosymbiont of unidentified scaly snail isolate Monju T04530 |
| 1416753 | Ensifer adhaerens OV14 T03054 |
| 106592 | Ensifer adhaerens T04507 |
| 716928 | Ensifer sojae CCBAU 05684 T05052 |
| 370354 | Entamoeba dispar SAW760 T01083 |
| 294381 | Entamoeba histolytica HM 1 IMSS T00238 |
| 370355 | Entamoeba invadens IP1 T04133 |
| 1421338 | Enterobacter asburiae L1 T03084 |
| 69218 | Enterobacter cancerogenus T05545 |
| 1333850 | Enterobacter cloacae ECNIH2 T03471 |
| 716541 | Enterobacter cloacae subsp cloacae ATCC 13047 T01231 |
| 208224 | Enterobacter cloacae subsp cloacae ENHKU01 T02315 |
| 158836 | Enterobacter cloacae subsp cloacae NCTC 9394 T02615 |
| 1104326 | Enterobacter cloacae subsp dissolvens SDM T02159 |
| 550 | Enterobacter cloacae T03429 |
| 550 | Enterobacter cloacae T03486 |
| 550 | Enterobacter cloacae T03706 |
| 1333851 | Enterobacter hormaechei subsp hoffmannii ECNIH3 T03350 |
| 1333849 | Enterobacter hormaechei subsp hoffmannii ECR091 T03378 |
| 301105 | Enterobacter hormaechei subsp hormaechei T03709 |
| 299766 | Enterobacter hormaechei subsp steigerwaltii T03708 |
| 1296536 | Enterobacter hormaechei subsp xiangfangensis T03707 |
| 1296536 | Enterobacter hormaechei subsp xiangfangensis T04821 |
| 158836 | Enterobacter hormaechei T04593 |
| 208224 | Enterobacter kobei T04686 |
| 701347 | Enterobacter lignolyticus SCF1 T01342 |
| 1334193 | Enterobacter lignolyticus T04198 |
| 299767 | Enterobacter ludwigii T01652 |
| 299767 | Enterobacter ludwigii T04719 |
| 1812935 | Enterobacter roggenkampii T04878 |
| 1812935 | Enterobacter roggenkampii T05556 |
| 885040 | Enterobacter soli T01604 |
| 399742 | Enterobacter sp 638 T00507 |
| 1560339 | Enterobacter sp E20 T04118 |
| 1692238 | Enterobacter sp FY 07 T04405 |
| 1166130 | Enterobacter sp R4 368 T02700 |
| 1920109 | Enterobacteriaceae bacterium ENNIH2 T05417 |
| 2052938 | Enterobacteriaceae bacterium S05 T05725 |
| 693444 | Enterobacteriaceae bacterium strain FGI 57 T02431 |
| 208479 | Enterocloster bolteae T05466 |
| 565655 | Enterococcus casseliflavus EC20 T02623 |
| 53345 | Enterococcus durans T04048 |
| 936153 | Enterococcus faecalis 62 T01881 |
| 1201292 | Enterococcus faecalis ATCC 29212 T03320 |
| 1206105 | Enterococcus faecalis D32 T02172 |
| 1287066 | Enterococcus faecalis DENG1 T03043 |
| 474186 | Enterococcus faecalis OG1RF T01882 |
| 1261557 | Enterococcus faecalis str Symbioflor 1 T02399 |
| 1351 | Enterococcus faecalis T02585 |
| 226185 | Enterococcus faecalis V583 T00123 |
| 1158600 | Enterococcus faecium ATCC 8459 NRRL B 2354 T02454 |
| 1155766 | Enterococcus faecium Aus0004 T01772 |
| 1305849 | Enterococcus faecium Aus0085 T02788 |
| 333849 | Enterococcus faecium DO T02120 |
| 1344042 | Enterococcus faecium T110 T03257 |
| 1353 | Enterococcus gallinarum T04148 |
| 160453 | Enterococcus gilvus T05664 |
| 768486 | Enterococcus hirae ATCC 9790 T02160 |
| 1300150 | Enterococcus mundtii QU 25 T02931 |
| 332949 | Enterococcus silesiacus T04218 |
| 417368 | Enterococcus thailandicus T05312 |
| 74700 | Entomoplasma freundtii T05222 |
| 214888 | Entomoplasma luminosum T05209 |
| 81459 | Entomoplasma melaleucae T05235 |
| 215578 | Entomoplasma somnilux T05210 |
| 2268024 | Ephemeroptericola cinctiostellae T05560 |
| 1265309 | Epibacterium mobile F1926 T04367 |
| 9793 | Equus asinus T04645 |
| 9796 | Equus caballus T01058 |
| 9798 | Equus przewalskii T04644 |
| 931890 | Eremothecium cymbalariae DBVPG 7215 T02237 |
| 284811 | Eremothecium gossypii ATCC 10895 T00167 |
| 716540 | Erwinia amylovora ATCC 49946 T01199 |
| 665029 | Erwinia amylovora CFBP1430 T01197 |
| 634500 | Erwinia billingiae Eb661 T01270 |
| 1619313 | Erwinia gerundensis T04258 |
| 55211 | Erwinia persicina T05618 |
| 644651 | Erwinia pyrifoliae DSM 12163 T02121 |
| 634499 | Erwinia pyrifoliae Ep1 96 T01000 |
| 215689 | Erwinia sp Ejp617 T01935 |
| 465817 | Erwinia tasmaniensis Et1 99 T00711 |
| 1514105 | Erysipelothrix larvae T04263 |
| 650150 | Erysipelothrix rhusiopathiae str Fujisawa T01525 |
| 1313290 | Erysipelothrix rhusiopathiae SY1027 T02688 |
| 2485784 | Erysipelothrix sp 15TAL0474 T05733 |
| 1834207 | Erysipelotrichaceae bacterium I46 T04439 |
| 1648404 | Erythrobacter atlanticus T03997 |
| 2182384 | Erythrobacter aureus T05587 |
| 192812 | Erythrobacter flavus T04986 |
| 502682 | Erythrobacter gangjinensis T04581 |
| 314225 | Erythrobacter litoralis HTCC2594 T00315 |
| 39960 | Erythrobacter litoralis T04740 |
| 2011159 | Erythrobacter sp KY5 T05511 |
| 1440052 | Escherichia albertii KF1 T03986 |
| 216592 | Escherichia coli 042 T02067 |
| 362663 | Escherichia coli 536 T00373 |
| 585055 | Escherichia coli 55989 T00826 |
| 655817 | Escherichia coli ABU 83972 T01995 |
| 405955 | Escherichia coli APEC O1 T00425 |
| 1274814 | Escherichia coli APEC O78 T02445 |
| 481805 | Escherichia coli ATCC 8739 T00697 |
| 413997 | Escherichia coli B str REL606 T00944 |
| 866768 | Escherichia coli BL21 Gold pLysS AG T00939 |
| 469008 | Escherichia coli BL21 T00931 |
| 469008 | Escherichia coli BL21 T02096 |
| 595496 | Escherichia coli BW2952 T00913 |
| 199310 | Escherichia coli CFT073 T00106 |
| 536056 | Escherichia coli DH1 T01996 |
| 536056 | Escherichia coli DH1 T02102 |
| 585397 | Escherichia coli ED1a T00827 |
| 316401 | Escherichia coli ETEC H10407 T02069 |
| 331112 | Escherichia coli HS T00591 |
| 585034 | Escherichia coli IAI1 T00828 |
| 585057 | Escherichia coli IAI39 T00829 |
| 714962 | Escherichia coli IHE3034 T01997 |
| 1355100 | Escherichia coli JJ1886 T02896 |
| 595495 | Escherichia coli KO11FL T01718 |
| 595495 | Escherichia coli KO11FL T02104 |
| 591946 | Escherichia coli LF82 T00854 |
| 1335916 | Escherichia coli LY180 T02846 |
| 1033813 | Escherichia coli NA114 T01998 |
| 585395 | Escherichia coli O103 H2 str 12009 T01096 |
| 1134782 | Escherichia coli O104 H4 str 2009EL 2050 T02316 |
| 1133853 | Escherichia coli O104 H4 str 2009EL 2071 T02256 |
| 1133852 | Escherichia coli O104 H4 str 2011C 3493 T02257 |
| 585396 | Escherichia coli O111 H str 11128 T01097 |
| 574521 | Escherichia coli O127 H6 str E2348 69 T00796 |
| 331111 | Escherichia coli O139 H28 str E24377A T00590 |
| 1248902 | Escherichia coli O145 H28 str RM13514 T03010 |
| 1248915 | Escherichia coli O145 H28 str RM13516 T03011 |
| 444450 | Escherichia coli O157 H7 str EC4115 T00778 |
| 155864 | Escherichia coli O157 H7 str EDL933 T00044 |
| 386585 | Escherichia coli O157 H7 str Sakai T00048 |
| 544404 | Escherichia coli O157 H7 str TW14359 T00949 |
| 941322 | Escherichia coli O25b H4 ST131 T04373 |
| 573235 | Escherichia coli O26 H11 str 11368 T01098 |
| 701177 | Escherichia coli O55 H7 str CB9615 T01190 |
| 1048689 | Escherichia coli O55 H7 str RM12579 T02066 |
| 1072459 | Escherichia coli O7 K1 str CE10 T01999 |
| 685038 | Escherichia coli O83 H1 str NRG 857C T02068 |
| 910348 | Escherichia coli P12b T02030 |
| 1382700 | Escherichia coli PMV 1 T02847 |
| 585035 | Escherichia coli S88 T00830 |
| 409438 | Escherichia coli SE11 T00784 |
| 431946 | Escherichia coli SE15 T02077 |
| 439855 | Escherichia coli SMS 3 5 T00672 |
| 885275 | Escherichia coli str clone D i14 T02003 |
| 885276 | Escherichia coli str clone D i2 T02004 |
| 316385 | Escherichia coli str K 12 substr DH10B T00666 |
| 1110693 | Escherichia coli str K 12 substr MDS42 T02541 |
| 511145 | Escherichia coli str K 12 substr MG1655 T00007 |
| 316407 | Escherichia coli str K 12 substr W3110 T00068 |
| 869729 | Escherichia coli UM146 T02000 |
| 585056 | Escherichia coli UMN026 T00831 |
| 696406 | Escherichia coli UMNK88 T02001 |
| 364106 | Escherichia coli UTI89 T00338 |
| 566546 | Escherichia coli W T02002 |
| 566546 | Escherichia coli W T02101 |
| 741093 | Escherichia coli Xuzhou21 T02122 |
| 585054 | Escherichia fergusonii ATCC 35469 T00811 |
| 1499973 | Escherichia marmotae T05353 |
| 8010 | Esox lucius T04894 |
| 663278 | Ethanoligenens harbinense YUAN 3 T01384 |
| 515620 | Eubacterium eligens ATCC 27750 T00908 |
| 53442 | Eubacterium limosum KIST612 T01336 |
| 515619 | Eubacterium rectale ATCC 33656 T00909 |
| 657318 | Eubacterium rectale DSM 17629 T02587 |
| 657317 | Eubacterium rectale M104 1 T02588 |
| 657319 | Eubacterium siraeum 70 3 T02590 |
| 717961 | Eubacterium siraeum V10Sc8a T02589 |
| 888727 | Eubacterium sulci ATCC 35585 T04038 |
| 71139 | Eucalyptus grandis T03547 |
| 72664 | Eutrema salsugineum T02985 |
| 1287681 | Eutypa lata UCREL1 T03103 |
| 1761453 | Euzebyella marina T05677 |
| 2652724 | Exaiptasia pallida T05080 |
| 1087448 | Exiguobacterium antarcticum B7 T02258 |
| 262543 | Exiguobacterium sibiricum 255 15 T00687 |
| 360911 | Exiguobacterium sp AT1b T00897 |
| 1399115 | Exiguobacterium sp MH3 T02903 |
| 1849031 | Exiguobacterium sp U13 1 T04561 |
| 718252 | Faecalibacterium prausnitzii L2 6 T02591 |
| 657322 | Faecalibacterium prausnitzii SL3 3 T02592 |
| 1702221 | Faecalibaculum rodentium T04268 |
| 717960 | Faecalitalea cylindroides T2 87 T02586 |
| 345164 | Falco cherrug T02911 |
| 8954 | Falco peregrinus T02856 |
| 236753 | Fastidiosipila sanguinis T05345 |
| 9685 | Felis catus T02385 |
| 1562970 | Fermentimonas caenicola T03818 |
| 550540 | Ferrimonas balearica DSM 9799 T01318 |
| 1188319 | Ferriphaselus amnicola T05631 |
| 589924 | Ferroglobus placidus DSM 10642 T01172 |
| 333146 | Ferroplasma acidarmanus fer1 T02713 |
| 74969 | Ferroplasma acidiphilum T04783 |
| 1163730 | Fervidicoccus fontis Kam940 T01936 |
| 2423 | Fervidobacterium islandicum T04215 |
| 381764 | Fervidobacterium nodosum Rt17 B1 T00579 |
| 771875 | Fervidobacterium pennivorans DSM 9078 T01808 |
| 1166018 | Fibrella aestuarina BUZ 2 T02429 |
| 1834519 | Fibrella sp ES10 3 2 2 T04805 |
| 59374 | Fibrobacter succinogenes subsp succinogenes S85 T01099 |
| 59374 | Fibrobacter succinogenes subsp succinogenes S85 T02105 |
| 59894 | Ficedula albicollis T02815 |
| 255247 | Fictibacillus arsenicus T04452 |
| 1221500 | Fictibacillus phosphorivorans T04282 |
| 546269 | Filifactor alocis ATCC 35896 T01663 |
| 477680 | Filimonas lacunae T05172 |
| 661478 | Fimbriimonas ginsengisoli Gsoil 348 T03038 |
| 334413 | Finegoldia magna ATCC 29328 T00661 |
| 1752063 | Fischerella sp NIES 3754 T04195 |
| 1383885 | Flagellimonas maritima T05505 |
| 2494373 | Flammeovirga pectinis T05771 |
| 1191459 | Flammeovirga sp MY04 T04442 |
| 1257021 | Flammeovirgaceae bacterium 311 T04058 |
| 1282737 | Flaviflexus salsibiostraticola T05752 |
| 2496867 | Flaviflexus sp H23T48 T05772 |
| 1492898 | Flavisolibacter tropicus T04392 |
| 1803846 | Flavivirga eckloniae T05284 |
| 531844 | Flavobacteriaceae bacterium 3519 10 T00953 |
| 1150389 | Flavobacteriaceae bacterium UJ101 T04720 |
| 459526 | Flavobacterium anhuiense T04718 |
| 1784713 | Flavobacterium arcticum T05621 |
| 1034807 | Flavobacterium branchiophilum FL 15 T01625 |
| 1041826 | Flavobacterium columnare ATCC 49512 T01653 |
| 1306519 | Flavobacterium commune T05006 |
| 1355330 | Flavobacterium faecale T05515 |
| 1492737 | Flavobacterium gilvum T04609 |
| 1094466 | Flavobacterium indicum GPTSA100 9 DSM 17447 T01781 |
| 376686 | Flavobacterium johnsoniae UW101 T00508 |
| 1678728 | Flavobacterium kingsejongi T05683 |
| 1452725 | Flavobacterium psychrophilum FPG101 T03281 |
| 1452724 | Flavobacterium psychrophilum FPG3 T03296 |
| 402612 | Flavobacterium psychrophilum JIP02 86 T00548 |
| 96345 | Flavobacterium psychrophilum T03254 |
| 96345 | Flavobacterium psychrophilum T03412 |
| 96345 | Flavobacterium psychrophilum T03659 |
| 96345 | Flavobacterium psychrophilum T03660 |
| 96345 | Flavobacterium psychrophilum T03661 |
| 96345 | Flavobacterium psychrophilum T04556 |
| 292800 | Flavonifractor plautii T04429 |
| 717231 | Flexistipes sinusarabici DSM 4947 T01539 |
| 755732 | Fluviicola taffensis DSM 16823 T01455 |
| 158441 | Folsomia candida T05043 |
| 694068 | Fomitiporia mediterranea MF3 22 T03132 |
| 1336794 | Formosa sp Hel1 33 131 T04465 |
| 1336795 | Formosa sp Hel3 A1 48 T04482 |
| 101020 | Fragaria vesca subsp vesca T02666 |
| 635003 | Fragilariopsis cylindrus CCMP1102 T04784 |
| 984129 | Francisella cf novicida Fx1 T01937 |
| 1542390 | Francisella frigiditurris T05636 |
| 549298 | Francisella halioticida T04972 |
| 1088883 | Francisella hispaniensis FSC454 T04700 |
| 622488 | Francisella hispaniensis T02078 |
| 1356861 | Francisella noatunensis subsp orientalis LADL 07 285A T02952 |
| 1163389 | Francisella noatunensis subsp orientalis str Toba 04 T02112 |
| 1086726 | Francisella persica ATCC VR 331 T04357 |
| 539329 | Francisella philomiragia subsp philomiragia ATCC 25015 T03764 |
| 484022 | Francisella philomiragia subsp philomiragia ATCC 25017 T00652 |
| 28110 | Francisella philomiragia T03617 |
| 28110 | Francisella philomiragia T03618 |
| 28110 | Francisella philomiragia T03619 |
| 28110 | Francisella philomiragia T03620 |
| 28110 | Francisella philomiragia T03621 |
| 573569 | Francisella salina T01560 |
| 653937 | Francisella sp FSC1006 T03403 |
| 475375 | Francisella sp MA067296 T05204 |
| 1232394 | Francisella tularensis subsp holarctica F92 T02349 |
| 351581 | Francisella tularensis subsp holarctica FSC200 T02350 |
| 458234 | Francisella tularensis subsp holarctica FTNF002 00 T00584 |
| 376619 | Francisella tularensis subsp holarctica LVS T00331 |
| 393011 | Francisella tularensis subsp holarctica OSU18 T00399 |
| 1432652 | Francisella tularensis subsp holarctica PHIT FT049 T03033 |
| 119857 | Francisella tularensis subsp holarctica T03713 |
| 119857 | Francisella tularensis subsp holarctica T03714 |
| 119857 | Francisella tularensis subsp holarctica T03715 |
| 441952 | Francisella tularensis subsp mediasiatica FSC147 T00698 |
| 1452728 | Francisella tularensis subsp novicida F6168 T03873 |
| 264 | Francisella tularensis subsp novicida T03875 |
| 401614 | Francisella tularensis subsp novicida U112 T00432 |
| 401614 | Francisella tularensis subsp novicida U112 T03874 |
| 393115 | Francisella tularensis subsp tularensis FSC198 T00381 |
| 510831 | Francisella tularensis subsp tularensis NE061598 T01938 |
| 177416 | Francisella tularensis subsp tularensis SCHU S4 T00214 |
| 1341656 | Francisella tularensis subsp tularensis str SCHU S4 substr NR 28534 T03888 |
| 1001534 | Francisella tularensis subsp tularensis TI0902 T01761 |
| 1001542 | Francisella tularensis subsp tularensis TIGB03 T01762 |
| 418136 | Francisella tularensis subsp tularensis WY96 3418 T00495 |
| 573570 | Francisella uliginis T05017 |
| 326424 | Frankia alni ACN14a T00384 |
| 106370 | Frankia casuarinae T00320 |
| 298654 | Frankia inefficax T01354 |
| 298653 | Frankia sp EAN1pec T00608 |
| 767434 | Frateuria aurantia DSM 6220 T01773 |
| 651822 | Fretibacterium fastidiosum T02602 |
| 1267021 | Frischella perrara T03525 |
| 1795630 | Frondihabitans sp PAMC 28766 T04281 |
| 1891926 | Fuerstia marisgermanicae T04678 |
| 229533 | Fusarium graminearum PH 1 T01038 |
| 426428 | Fusarium oxysporum f sp lycopersici 4287 T04882 |
| 1028729 | Fusarium pseudograminearum CS3096 T03456 |
| 334819 | Fusarium verticillioides 7600 T04869 |
| 469615 | Fusobacterium gonidiaformans ATCC 25563 T05460 |
| 1307442 | Fusobacterium hwasookii ChDC F174 T04183 |
| 469616 | Fusobacterium mortiferum ATCC 9817 T05444 |
| 469607 | Fusobacterium nucleatum subsp animalis 4 8 T02680 |
| 457405 | Fusobacterium nucleatum subsp animalis 7 1 T03612 |
| 190304 | Fusobacterium nucleatum subsp nucleatum ATCC 25586 T00077 |
| 469602 | Fusobacterium nucleatum subsp vincentii 3 1 27 T03625 |
| 469604 | Fusobacterium nucleatum subsp vincentii 3 1 36A2 T02822 |
| 2663009 | Fusobacterium periodonticum T05232 |
| 861 | Fusobacterium ulcerans T05433 |
| 856 | Fusobacterium varium T05280 |
| 130081 | Galdieria sulphuraria T02926 |
| 1005058 | Gallibacterium anatis UMN179 T01482 |
| 395494 | Gallionella capsiferriformans ES 2 T01295 |
| 9031 | Gallus gallus T01006 |
| 83406 | Gamma proteobacterium HdN1 T01300 |
| 553190 | Gardnerella vaginalis 409 05 T01146 |
| 525284 | Gardnerella vaginalis ATCC 14019 T01344 |
| 1009464 | Gardnerella vaginalis HMP9231 T01939 |
| 1173025 | Geitlerinema sp PCC 7407 T02366 |
| 146911 | Gekko japonicus T04299 |
| 29391 | Gemella morbillorum T05494 |
| 2040624 | Gemella sp ND 6198 T05584 |
| 1785995 | Gemella sp oral taxon 928 T04256 |
| 1615909 | Geminocystis sp NIES 3708 T04168 |
| 1617448 | Geminocystis sp NIES 3709 T04142 |
| 114 | Gemmata obscuriglobus T05475 |
| 1630693 | Gemmata sp SH PL17 T04358 |
| 379066 | Gemmatimonas aurantiaca T 27 T00878 |
| 1379270 | Gemmatimonas phototrophica T04580 |
| 861299 | Gemmatirosa kalamazoonesis T03025 |
| 2169400 | Gemmobacter sp HYN0069 T05407 |
| 483547 | Geoalkalibacter subterraneus T03585 |
| 1921421 | Geobacillus genomosp 3 T02798 |
| 235909 | Geobacillus kaustophilus HTA426 T00217 |
| 169283 | Geobacillus lituanicus T05193 |
| 1629723 | Geobacillus sp 12AMOR1 T03966 |
| 691437 | Geobacillus sp C56 T3 T01245 |
| 1233873 | Geobacillus sp GHH01 T02455 |
| 1813182 | Geobacillus sp JS12 T04412 |
| 1519377 | Geobacillus sp LC300 T04018 |
| 471223 | Geobacillus sp WCH70 T00915 |
| 581103 | Geobacillus sp Y4 1MC1 T01348 |
| 550542 | Geobacillus sp Y412MC52 T01399 |
| 544556 | Geobacillus sp Y412MC61 T01101 |
| 272567 | Geobacillus stearothermophilus 10 T04043 |
| 129338 | Geobacillus subterraneus T04323 |
| 33938 | Geobacillus thermocatenulatus T05178 |
| 420246 | Geobacillus thermodenitrificans NG80 2 T00496 |
| 1111068 | Geobacillus thermoleovorans CCB US3 UF5 T01676 |
| 33941 | Geobacillus thermoleovorans T04710 |
| 1340425 | Geobacter anodireducens T04399 |
| 404380 | Geobacter bemidjiensis Bem T00749 |
| 316067 | Geobacter daltonii FRC 32 T00846 |
| 398767 | Geobacter lovleyi SZ T00708 |
| 269799 | Geobacter metallireducens GS 15 T00295 |
| 345632 | Geobacter pickeringii T03568 |
| 443143 | Geobacter sp M18 T01421 |
| 443144 | Geobacter sp M21 T00933 |
| 663917 | Geobacter sulfurreducens KN400 T01940 |
| 243231 | Geobacter sulfurreducens PCA T00155 |
| 351605 | Geobacter uraniireducens Rf4 T00521 |
| 526225 | Geodermatophilus obscurus DSM 43160 T01163 |
| 565033 | Geoglobus acetivorans T03507 |
| 113653 | Geoglobus ahangari T03939 |
| 2341112 | Georhizobium profundi T05798 |
| 48883 | Geospiza fortis T03755 |
| 1424294 | Geosporobacter ferrireducens T04493 |
| 139438 | Geovibrio thiophilus T05803 |
| 184922 | Giardia lamblia ATCC 50803 T01047 |
| 929813 | Gibbsiella quercinecans T05060 |
| 1196095 | Gilliamella apicola T03066 |
| 1085623 | Glaciecola nitratireducens FR1064 T01632 |
| 983545 | Glaciecola sp 4H 3 7 YE 5 T01483 |
| 557723 | Glaesserella parasuis SH0165 T00825 |
| 738 | Glaesserella parasuis T03301 |
| 738 | Glaesserella parasuis T03312 |
| 1322346 | Glaesserella parasuis ZJ0906 T02697 |
| 2030797 | Glaesserella sp 15 184 T05643 |
| 1116229 | Glarea lozoyensis ATCC 20868 T04883 |
| 1183438 | Gloeobacter kilaueensis JS1 T02885 |
| 251221 | Gloeobacter violaceus PCC 7421 T00148 |
| 1173026 | Gloeocapsa sp PCC 7428 T02400 |
| 670483 | Gloeophyllum trabeum ATCC 11539 T03130 |
| 65393 | Gloeothece citriformis PCC 7424 T00808 |
| 497965 | Gloeothece verrucosa PCC 7822 T01310 |
| 272568 | Gluconacetobacter diazotrophicus PA1 5 T00631 |
| 272568 | Gluconacetobacter diazotrophicus PA1 5 T00798 |
| 318683 | Gluconobacter albidus T04736 |
| 290633 | Gluconobacter oxydans 621H T00230 |
| 1288313 | Gluconobacter oxydans DSM 3504 T03563 |
| 1224746 | Gluconobacter oxydans H24 T02348 |
| 861360 | Glutamicibacter arilaitensis Re117 T01320 |
| 1933880 | Glutamicibacter halophytocola T04229 |
| 3847 | Glycine max T01710 |
| 1434191 | Glycocaulis alkaliphilus T05786 |
| 84096 | Gordonia alkanivorans T05876 |
| 526226 | Gordonia bronchialis DSM 43247 T01102 |
| 2420509 | Gordonia insulae T05722 |
| 1004901 | Gordonia iterans T05370 |
| 1136941 | Gordonia phthalatica T04097 |
| 1112204 | Gordonia polyisoprenivorans VH2 T01719 |
| 36822 | Gordonia rubripertincta T05629 |
| 337191 | Gordonia sp KTR9 T02216 |
| 2059875 | Gordonia sp YC JH1 T05256 |
| 2055 | Gordonia terrae T04440 |
| 657308 | Gordonibacter pamelaeae 7 10 1 b T02609 |
| 9595 | Gorilla gorilla gorilla T02442 |
| 29729 | Gossypium arboreum T05776 |
| 3635 | Gossypium hirsutum T04793 |
| 29730 | Gossypium raimondii T04129 |
| 1128398 | Gottschalkia acidurici 9a T02255 |
| 1229726 | Gramella flava JLT2011 T04640 |
| 411154 | Gramella forsetii KT0803 T00448 |
| 2126553 | Gramella fulva T05367 |
| 1913577 | Gramella salexigens T04586 |
| 391165 | Granulibacter bethesdensis CGDNIH1 T00392 |
| 1051974 | Granulibacter bethesdensis CGDNIH2 T03083 |
| 364410 | Granulibacter bethesdensis CGDNIH3 T03155 |
| 1052095 | Granulibacter bethesdensis CGDNIH4 T03156 |
| 682795 | Granulicella mallensis MP5ACTX8 T01664 |
| 1198114 | Granulicella tundricola MP5ACTX9 T01411 |
| 1192854 | Granulosicoccus antarcticus IMCC3135 T04938 |
| 673 | Grimontia hollisae T04260 |
| 2419771 | Gryllotalpicola sp 2DFW10M 5 T05660 |
| 905079 | Guillardia theta CCMP2712 T02925 |
| 1445510 | Gynuella sunshinyii YC6258 T03690 |
| 195105 | Haematobacter massiliensis T05861 |
| 1549855 | Haematospirillum jordaniae T04366 |
| 233412 | Haemophilus ducreyi 35000HP T00135 |
| 862964 | Haemophilus influenzae 10810 T01720 |
| 281310 | Haemophilus influenzae 86 028NP T00248 |
| 1295140 | Haemophilus influenzae CGSHiCZ412602 T03436 |
| 866630 | Haemophilus influenzae F3031 T01395 |
| 935897 | Haemophilus influenzae F3047 T01400 |
| 1334187 | Haemophilus influenzae KR494 T02839 |
| 374930 | Haemophilus influenzae PittEE T00543 |
| 374931 | Haemophilus influenzae PittGG T00544 |
| 262727 | Haemophilus influenzae R2846 T01980 |
| 262728 | Haemophilus influenzae R2866 T01981 |
| 71421 | Haemophilus influenzae Rd KW20 T00001 |
| 727 | Haemophilus influenzae T03485 |
| 727 | Haemophilus influenzae T03720 |
| 727 | Haemophilus influenzae T03721 |
| 727 | Haemophilus influenzae T03722 |
| 862965 | Haemophilus parainfluenzae T3T1 T01605 |
| 712310 | Haemophilus sp oral taxon 036 T05707 |
| 1453496 | Hafnia alvei FB1 T03425 |
| 546367 | Hafnia paralvei T05667 |
| 349521 | Hahella chejuensis KCTC 2396 T00307 |
| 1628392 | Hahella sp KA22 T05793 |
| 795797 | Halalkalicoccus jeotgali B3 T01271 |
| 1604004 | Halanaeroarchaeum sulfurireducens T03927 |
| 1604004 | Halanaeroarchaeum sulfurireducens T04261 |
| 656519 | Halanaerobium hydrogeniformans T01349 |
| 572479 | Halanaerobium praevalens DSM 2228 T01941 |
| 502025 | Haliangium ochraceum DSM 14365 T01103 |
| 930805 | Halioglobus japonicus T04687 |
| 760192 | Haliscomenobacter hydrossis DSM 1100 T01491 |
| 634497 | Haloarcula hispanica ATCC 33960 T01597 |
| 1417673 | Haloarcula hispanica N601 T02949 |
| 272569 | Haloarcula marismortui ATCC 43049 T00211 |
| 1592728 | Haloarcula sp CBA1115 T03645 |
| 1932004 | Haloarcula taiwanensis T05292 |
| 1679096 | Haloarculaceae archaeon HArcel1 T05406 |
| 866895 | Halobacillus halophilus DSM 2266 T02031 |
| 45668 | Halobacillus litoralis T05828 |
| 402384 | Halobacillus mangrovi T04799 |
| 862908 | Halobacteriovorax marinus SJ T01659 |
| 2109558 | Halobacteriovorax sp BALOs 7 T05649 |
| 1407499 | Halobacterium hubeiense T04202 |
| 64091 | Halobacterium salinarum NRC 1 T00038 |
| 478009 | Halobacterium salinarum R1 T00662 |
| 751944 | Halobacterium sp DL1 T03878 |
| 748449 | Halobacteroides halobius DSM 5150 T02401 |
| 358396 | Halobiforma lacisalsi AJ5 T04669 |
| 2382161 | Halocella sp SP3 1 T05769 |
| 1579316 | Halocynthiibacter arcticus T04275 |
| 1873524 | Halodesulfurarchaeum formicicum T04517 |
| 1873524 | Halodesulfurarchaeum formicicum T04550 |
| 35746 | Haloferax gibbonsii T04025 |
| 523841 | Haloferax mediterranei ATCC 33500 T02129 |
| 309800 | Haloferax volcanii DS2 T01200 |
| 469382 | Halogeometricum borinquense DSM 11551 T01359 |
| 1073996 | Halohasta litchfieldiae T05239 |
| 485914 | Halomicrobium mukohataei DSM 12286 T00982 |
| 1641165 | Halomicronema hongdechloris C2206 T04928 |
| 1897729 | Halomonas aestuarii T04554 |
| 272774 | Halomonas alkaliphila T05720 |
| 475662 | Halomonas beimenensis T05125 |
| 213554 | Halomonas campaniensis T03198 |
| 507626 | Halomonas chromatireducens T04292 |
| 768066 | Halomonas elongata DSM 2581 T01311 |
| 1178482 | Halomonas huangheensis T04119 |
| 115561 | Halomonas hydrothermalis T05117 |
| 1883416 | Halomonas sp 1513 T04761 |
| 1118153 | Halomonas sp GFAJ 1 T05375 |
| 1504981 | Halomonas sp KO116 T03800 |
| 1610576 | Halomonas sp R57 5 T04029 |
| 2136172 | Halomonas sp SF2003 T05423 |
| 44935 | Halomonas venusta T05892 |
| 756883 | Halophilic archaeon DL31 T01603 |
| 797210 | Halopiger xanaduensis SH 6 T01532 |
| 660522 | Haloplanus aerogenes T05732 |
| 1547898 | Haloplanus sp CBA1112 T05557 |
| 1547898 | Haloplanus sp CBA1113 T05613 |
| 768065 | Haloquadratum walsbyi C23 T01942 |
| 362976 | Haloquadratum walsbyi DSM 16790 T00375 |
| 1033806 | Halorhabdus tiamatea SARL4B T02781 |
| 519442 | Halorhabdus utahensis DSM 12940 T00971 |
| 421628 | Halorhodospira halochloris str A T03059 |
| 349124 | Halorhodospira halophila SL1 T00462 |
| 1932360 | Halorientalis sp IM1011 T04693 |
| 416348 | Halorubrum lacusprofundi ATCC 49239 T00856 |
| 2497325 | Halorubrum sp BOL3 1 T05834 |
| 634157 | Halorubrum sp PV6 T05770 |
| 797299 | Halostagnicola larsenii XH 48 T03008 |
| 376489 | Halotalea alkalilenta T04333 |
| 588898 | Haloterrigena daqingensis T04679 |
| 543526 | Haloterrigena turkmenica DSM 5511 T01154 |
| 65093 | Halothece sp PCC 7418 T02379 |
| 373903 | Halothermothrix orenii H 168 T00838 |
| 555778 | Halothiobacillus neapolitanus c2 T01104 |
| 1860122 | Halothiobacillus sp LS2 T04441 |
| 797302 | Halovivax ruber XH 70 T02402 |
| 610380 | Harpegnathos saltator T03918 |
| 1482074 | Hartmannibacter diazotrophicus T05217 |
| 4232 | Helianthus annuus T05101 |
| 382638 | Helicobacter acinonychis str Sheeba T00371 |
| 135569 | Helicobacter apodemus T05516 |
| 37372 | Helicobacter bilis T04788 |
| 1002804 | Helicobacter bizzozeronii CIII 1 T01540 |
| 182217 | Helicobacter cetorum MIT 00 7128 T02032 |
| 1163745 | Helicobacter cetorum MIT 99 5656 T02033 |
| 537971 | Helicobacter cinaedi CCUG 18818 ATCC BAA 847 T02494 |
| 1172562 | Helicobacter cinaedi PAGU611 T02034 |
| 936155 | Helicobacter felis ATCC 49179 T01375 |
| 1216962 | Helicobacter heilmannii ASB1 4 T02626 |
| 235279 | Helicobacter hepaticus ATCC 51449 T00133 |
| 679897 | Helicobacter mustelae 12198 T01191 |
| 985081 | Helicobacter pylori 2017 T01913 |
| 985080 | Helicobacter pylori 2018 T01914 |
| 85962 | Helicobacter pylori 26695 T00008 |
| 85962 | Helicobacter pylori 26695 T02282 |
| 585535 | Helicobacter pylori 35A T01892 |
| 290847 | Helicobacter pylori 51 T02082 |
| 684950 | Helicobacter pylori 52 T01898 |
| 585538 | Helicobacter pylori 83 T01915 |
| 869727 | Helicobacter pylori 908 T01893 |
| 1055531 | Helicobacter pylori Aklavik117 T02352 |
| 1055532 | Helicobacter pylori Aklavik86 T02353 |
| 592205 | Helicobacter pylori B38 T00950 |
| 693745 | Helicobacter pylori B8 T01262 |
| 1407462 | Helicobacter pylori BM012A T02939 |
| 1407463 | Helicobacter pylori BM012S T02940 |
| 765964 | Helicobacter pylori Cuz20 T01894 |
| 1055527 | Helicobacter pylori ELS37 T01798 |
| 866344 | Helicobacter pylori F16 T01910 |
| 866345 | Helicobacter pylori F30 T01895 |
| 102608 | Helicobacter pylori F32 T01911 |
| 866346 | Helicobacter pylori F57 T01912 |
| 563041 | Helicobacter pylori G27 T00777 |
| 907240 | Helicobacter pylori Gambia94 24 T01917 |
| 357544 | Helicobacter pylori HPAG1 T00365 |
| 1163743 | Helicobacter pylori HUP B14 T02035 |
| 907238 | Helicobacter pylori India7 T01918 |
| 85963 | Helicobacter pylori J99 T00020 |
| 907237 | Helicobacter pylori Lithuania75 T01896 |
| 1248725 | Helicobacter pylori OK113 T02510 |
| 1248726 | Helicobacter pylori OK310 T02542 |
| 1382920 | Helicobacter pylori oki102 T03182 |
| 1382921 | Helicobacter pylori oki112 T03723 |
| 1382922 | Helicobacter pylori oki128 T03724 |
| 1382923 | Helicobacter pylori oki154 T03725 |
| 1382924 | Helicobacter pylori oki422 T03726 |
| 1382925 | Helicobacter pylori oki673 T03727 |
| 1382926 | Helicobacter pylori oki828 T03728 |
| 1382927 | Helicobacter pylori oki898 T03729 |
| 570508 | Helicobacter pylori P12 T00786 |
| 1163742 | Helicobacter pylori PeCan18 T02036 |
| 765963 | Helicobacter pylori PeCan4 T01322 |
| 1055528 | Helicobacter pylori Puno120 T01919 |
| 1055529 | Helicobacter pylori Puno135 T01920 |
| 1234365 | Helicobacter pylori Rif1 T02317 |
| 1234600 | Helicobacter pylori Rif2 T02318 |
| 794851 | Helicobacter pylori Sat464 T01897 |
| 1163740 | Helicobacter pylori Shi112 T02037 |
| 1163741 | Helicobacter pylori Shi169 T02038 |
| 1163739 | Helicobacter pylori Shi417 T02039 |
| 512562 | Helicobacter pylori Shi470 T00742 |
| 765962 | Helicobacter pylori SJM180 T01323 |
| 1055530 | Helicobacter pylori SNT49 T01916 |
| 1352356 | Helicobacter pylori SouthAfrica20 T02807 |
| 907239 | Helicobacter pylori SouthAfrica7 T01921 |
| 1311573 | Helicobacter pylori UM032 T02658 |
| 1321939 | Helicobacter pylori UM037 T02659 |
| 1321940 | Helicobacter pylori UM066 T02660 |
| 1321941 | Helicobacter pylori UM298 T02757 |
| 1321938 | Helicobacter pylori UM299 T02661 |
| 637913 | Helicobacter pylori v225d T01899 |
| 1127122 | Helicobacter pylori XZ274 T02130 |
| 76936 | Helicobacter typhlonius T04174 |
| 29058 | Helicoverpa armigera T05609 |
| 498761 | Heliobacterium modesticaldum Ice1 T00653 |
| 6412 | Helobdella robusta T03272 |
| 1262470 | Herbaspirillum hiltneri N3 T04039 |
| 1078773 | Herbaspirillum rubrisubalbicans M1 T04238 |
| 757424 | Herbaspirillum seropedicae SmR1 T01268 |
| 964 | Herbaspirillum seropedicae T04519 |
| 2025949 | Herbaspirillum sp meg3 T05024 |
| 1679721 | Herbinix luporum T04072 |
| 204773 | Herminiimonas arsenicoxydans T00490 |
| 316274 | Herpetosiphon aurantiacus DSM 785 T00614 |
| 747525 | Heterobasidion irregulare TC 32 1 T03455 |
| 10181 | Heterocephalus glaber T02812 |
| 3981 | Hevea brasiliensis T05150 |
| 760142 | Hippea maritima DSM 10411 T01456 |
| 109280 | Hippocampus comes T04969 |
| 186990 | Hipposideros armiger T04661 |
| 582402 | Hirschia baltica ATCC 49814 T00945 |
| 205914 | Histophilus somni 129PT T00386 |
| 228400 | Histophilus somni 2336 T00673 |
| 2059318 | Histoplasma capsulatum NAm1 T02229 |
| 1620421 | Hoeflea sp IMCC20628 T03926 |
| 9606 | Homo sapiens T01001 |
| 443218 | Hoyosella subflava DQS3 9A1 T01514 |
| 2282656 | Humibacter sp BT305 T05558 |
| 1834198 | Hungateiclostridiaceae bacterium KB18 T04437 |
| 720554 | Hungateiclostridium clariflavum DSM 19732 T01661 |
| 1677857 | Hungateiclostridium saccincola T05223 |
| 203119 | Hungateiclostridium thermocellum ATCC 27405 T00474 |
| 637887 | Hungateiclostridium thermocellum DSM 1313 T01933 |
| 6087 | Hydra vulgaris T01080 |
| 2231112 | Hydrogenimonas sp T05789 |
| 608538 | Hydrogenobacter thermophilus TK 6 T01167 |
| 608538 | Hydrogenobacter thermophilus TK 6 T02106 |
| 547144 | Hydrogenobaculum sp HO T02482 |
| 547146 | Hydrogenobaculum sp SN T02516 |
| 380749 | Hydrogenobaculum sp Y04AAS1 T00745 |
| 1763535 | Hydrogenophaga crassostreae T04505 |
| 795665 | Hydrogenophaga sp PBC T04491 |
| 1842537 | Hydrogenophaga sp RAC07 T04455 |
| 297 | Hydrogenophilus thermoluteolus T05630 |
| 317025 | Hydrogenovibrio crunogenus XCL 2 T00296 |
| 265883 | Hydrogenovibrio thermophilus T05808 |
| 1850093 | Hymenobacter nivis T05498 |
| 2319843 | Hymenobacter oligotrophus T05656 |
| 1411621 | Hymenobacter sedentarius T04242 |
| 1356852 | Hymenobacter sp APR13 T03213 |
| 1385663 | Hymenobacter sp DG25A T04064 |
| 1385664 | Hymenobacter sp DG25B T03500 |
| 1484116 | Hymenobacter sp PAMC 26554 T04376 |
| 1484118 | Hymenobacter sp PAMC 26628 T04961 |
| 1227739 | Hymenobacter swuensis DY53 T03055 |
| 415426 | Hyperthermus butylicus DSM 5456 T00468 |
| 670307 | Hyphomicrobium denitrificans 1NES1 T02646 |
| 582899 | Hyphomicrobium denitrificans ATCC 51888 T01265 |
| 1029756 | Hyphomicrobium nitrativorans NL23 T02943 |
| 717785 | Hyphomicrobium sp MC1 T01557 |
| 1690484 | Hyphomonadaceae bacterium UKL13 1 T04338 |
| 228405 | Hyphomonas neptunium ATCC 15444 T00397 |
| 242600 | Ichthyobacterium seriolicida T05228 |
| 7998 | Ictalurus punctatus T04658 |
| 1321370 | Idiomarina loihiensis GSL 199 T02681 |
| 283942 | Idiomarina loihiensis L2TR T00218 |
| 1096243 | Idiomarina piscisalsi T04931 |
| 2100422 | Idiomarina sp OT37 5b T05379 |
| 2055892 | Idiomarina sp X4 T05226 |
| 945713 | Ignavibacterium album JCM 16511 T01943 |
| 453591 | Ignicoccus hospitalis KIN4 I T00587 |
| 940295 | Ignicoccus islandicus DSM 13165 T04205 |
| 583356 | Ignisphaera aggregans DSM 17230 T01307 |
| 1313172 | Ilumatobacter coccineus YM16 304 T02501 |
| 572544 | Ilyobacter polytropus DSM 2926 T01337 |
| 1810504 | Immundisolibacter cernigliae T04454 |
| 2487118 | Intestinibaculum porci T05736 |
| 1297617 | Intestinimonas butyriciproducens T04182 |
| 710696 | Intrasporangium calvum DSM 43043 T01385 |
| 2496266 | Iodobacter sp H11R3 T05767 |
| 35883 | Ipomoea nil T04663 |
| 1300344 | Isoptericola dokdonensis DS 3 T04384 |
| 743718 | Isoptericola variabilis 225 T01520 |
| 575540 | Isosphaera pallida ATCC 43644 T01407 |
| 6945 | Ixodes scapularis T01131 |
| 857417 | Janibacter indicus T04569 |
| 53458 | Janibacter limosus T05853 |
| 290400 | Jannaschia sp CCS1 T00330 |
| 1349767 | Janthinobacterium agaricidamnosum NBRC 102515 DSM 9628 T03157 |
| 1644131 | Janthinobacterium sp 1 2014MBL MicDiv T04539 |
| 2497863 | Janthinobacterium sp 17J80 10 T05896 |
| 1236179 | Janthinobacterium sp B9 8 T04271 |
| 1938606 | Janthinobacterium sp LM6 T04756 |
| 375286 | Janthinobacterium sp Marseille T00561 |
| 368607 | Janthinobacterium svalbardensis T05119 |
| 180498 | Jatropha curcas T03922 |
| 1906741 | Jeongeupia sp USM3 T04552 |
| 2496265 | Jeotgalibaca ciconiae T05782 |
| 708126 | Jeotgalibaca dankookensis T04733 |
| 1903686 | Jeotgalibaca sp PTS2502 T04689 |
| 1508404 | Jeotgalibacillus malaysiensis T03570 |
| 471856 | Jonesia denitrificans DSM 20603 T00972 |
| 51240 | Juglans regia T05245 |
| 914150 | Kangiella geojedonensis T03889 |
| 523791 | Kangiella koreensis DSM 16069 T00973 |
| 1561924 | Kangiella profundi T05254 |
| 1144748 | Kangiella sediminilitoris T04467 |
| 1071382 | Kazachstania africana CBS 2517 T02487 |
| 1917421 | Ketobacter alkanivorans T05294 |
| 92947 | Ketogulonicigenium robustum T04841 |
| 759362 | Ketogulonicigenium vulgare WSH 001 T01909 |
| 880591 | Ketogulonicigenium vulgare Y25 T01338 |
| 860235 | Kibdelosporangium phytohabitans T04088 |
| 266940 | Kineococcus radiotolerans SRS30216 ATCC BAA 149 T00562 |
| 504 | Kingella kingae T04151 |
| 1307763 | Kiritimatiella glycovorans T04225 |
| 68173 | Kitasatospora albolonga T05009 |
| 1894 | Kitasatospora aureofaciens T05021 |
| 452652 | Kitasatospora setae KM 6054 T01648 |
| 2018025 | Kitasatospora sp MMS16 BH015 T05366 |
| 935296 | Klebsiella aerogenes EA1509E T02447 |
| 1028307 | Klebsiella aerogenes KCTC 2190 T01529 |
| 1191061 | Klebsiella michiganensis E718 T02173 |
| 1308980 | Klebsiella michiganensis HKOPL1 T03188 |
| 1006551 | Klebsiella michiganensis KCTC 1686 T01681 |
| 1134687 | Klebsiella michiganensis T03349 |
| 1134687 | Klebsiella michiganensis T03782 |
| 1333852 | Klebsiella oxytoca KONIH1 T03269 |
| 571 | Klebsiella oxytoca T04534 |
| 1420012 | Klebsiella pneumoniae 30660 NJST258 1 T03176 |
| 1420013 | Klebsiella pneumoniae 30684 NJST258 2 T03177 |
| 244366 | Klebsiella pneumoniae 342 T00772 |
| 1244085 | Klebsiella pneumoniae CG43 T02866 |
| 1380908 | Klebsiella pneumoniae JM45 T02799 |
| 1049565 | Klebsiella pneumoniae KCTC 2242 T01982 |
| 1193292 | Klebsiella pneumoniae subsp pneumoniae 1084 T02213 |
| 1125630 | Klebsiella pneumoniae subsp pneumoniae HS11286 T01733 |
| 1094170 | Klebsiella pneumoniae subsp pneumoniae KPNIH10 T03788 |
| 1225181 | Klebsiella pneumoniae subsp pneumoniae KPNIH24 T03369 |
| 1328324 | Klebsiella pneumoniae subsp pneumoniae KPNIH27 T03370 |
| 1328325 | Klebsiella pneumoniae subsp pneumoniae KPR0928 T03371 |
| 272620 | Klebsiella pneumoniae subsp pneumoniae MGH 78578 T00566 |
| 484021 | Klebsiella pneumoniae subsp pneumoniae NTUH K2044 T00910 |
| 72407 | Klebsiella pneumoniae subsp pneumoniae T03418 |
| 72407 | Klebsiella pneumoniae subsp pneumoniae T03465 |
| 72407 | Klebsiella pneumoniae subsp pneumoniae T03466 |
| 72407 | Klebsiella pneumoniae subsp pneumoniae T03467 |
| 72407 | Klebsiella pneumoniae subsp pneumoniae T03468 |
| 861365 | Klebsiella pneumoniae subsp rhinoscleromatis SB3432 T02793 |
| 573 | Klebsiella pneumoniae T03419 |
| 573 | Klebsiella pneumoniae T03440 |
| 573 | Klebsiella pneumoniae T03746 |
| 573 | Klebsiella pneumoniae T03747 |
| 573 | Klebsiella pneumoniae T05281 |
| 1463165 | Klebsiella quasipneumoniae T04389 |
| 2026240 | Klebsiella quasivariicola T05116 |
| 1905288 | Klebsiella sp LTGPAF 6F T05751 |
| 640131 | Klebsiella variicola At 22 T01170 |
| 244366 | Klebsiella variicola T03368 |
| 244366 | Klebsiella variicola T03531 |
| 244366 | Klebsiella variicola T03641 |
| 2774055 | Kluyvera georgiana T04930 |
| 284590 | Kluyveromyces lactis NRRL Y 1140 T01025 |
| 1003335 | Kluyveromyces marxianus DMKU3 1042 T05269 |
| 446860 | Kocuria flava T04206 |
| 1049583 | Kocuria indica T05866 |
| 71999 | Kocuria palustris T04045 |
| 378753 | Kocuria rhizophila DC2201 T00701 |
| 33995 | Komagataeibacter europaeus T04893 |
| 634177 | Komagataeibacter medellinensis NBRC 3288 T01631 |
| 265960 | Komagataeibacter nataicola T04737 |
| 265959 | Komagataeibacter saccharivorans T05634 |
| 1296990 | Komagataeibacter xylinus E25 T03044 |
| 644223 | Komagataella phaffii GS115 T01092 |
| 2282170 | Kordia sp SMS9 T05562 |
| 1300165 | Kosakonia cowanii T04691 |
| 497725 | Kosakonia oryzae T04433 |
| 283686 | Kosakonia radicincitans T04579 |
| 1235834 | Kosakonia sacchari SP1 T03601 |
| 2492396 | Kosakonia sp CCTCC M2018092 T05734 |
| 521045 | Kosmotoga olearia TBF 19 5 1 T00914 |
| 1330330 | Kosmotoga pacifica T03949 |
| 153496 | Kozakia baliensis T04547 |
| 479435 | Kribbella flavida DSM 17836 T01155 |
| 37003 | Kryptolebias marmoratus T05608 |
| 1750719 | Kurthia sp 11kri321 T04250 |
| 698828 | Kushneria konosiri T04839 |
| 157779 | Kushneria marisflavi T04840 |
| 1449976 | Kutzneria albida DSM 43870 T03039 |
| 2055160 | Kyrpidia spormannii T05215 |
| 562970 | Kyrpidia tusciae DSM 2912 T01220 |
| 478801 | Kytococcus sedentarius DSM 20547 T00974 |
| 1717717 | Labilibaculum antarcticum T05376 |
| 1391654 | Labilithrix luteola T04033 |
| 187304 | Labrenzia aggregata T04723 |
| 1674922 | Labrenzia sp CP4 T04295 |
| 2021862 | Labrenzia sp VG12 T04984 |
| 486041 | Laccaria bicolor S238N H82 T01044 |
| 37482 | Laceyella sacchari T05304 |
| 559295 | Lachancea thermotolerans CBS 6340 T01117 |
| 617123 | Lachnoanaerobaculum umeaense T05639 |
| 357809 | Lachnoclostridium phytofermentans ISDg T00619 |
| 1834196 | Lachnoclostridium sp YL32 T04430 |
| 712991 | Lachnospiraceae bacterium oral taxon 500 T05749 |
| 1526571 | Lacimicrobium alkaliphilum T04189 |
| 983544 | Lacinutrix sp 5H 3 7 4 T01533 |
| 2057808 | Lacinutrix sp Bg11 31 T05225 |
| 1486034 | Lacinutrix venerupis T04680 |
| 1600 | Lactobacillus acetotolerans T03976 |
| 89059 | Lactobacillus acidipiscis T05349 |
| 1604 | Lactobacillus acidophilus 30SC T01436 |
| 1314884 | Lactobacillus acidophilus La 14 T02647 |
| 272621 | Lactobacillus acidophilus NCFM T00231 |
| 1579 | Lactobacillus acidophilus T03681 |
| 1601 | Lactobacillus agilis T05198 |
| 1423720 | Lactobacillus alimentarius DSM 20249 T05458 |
| 1847728 | Lactobacillus allii T05413 |
| 83683 | Lactobacillus amylolyticus T05057 |
| 1423721 | Lactobacillus amylophilus DSM 20533 JCM 1125 T05031 |
| 695560 | Lactobacillus amylovorus GRL 1112 T01371 |
| 695562 | Lactobacillus amylovorus GRL1118 T01954 |
| 375175 | Lactobacillus backii T04754 |
| 387344 | Lactobacillus brevis ATCC 367 T00406 |
| 1001583 | Lactobacillus brevis KB290 T02528 |
| 511437 | Lactobacillus buchneri NRRL B 30929 T01477 |
| 1071400 | Lactobacillus buchneri subsp silagei CD034 T02244 |
| 1051650 | Lactobacillus casei 12A T03665 |
| 1597 | Lactobacillus casei BD II T01955 |
| 1597 | Lactobacillus casei BL23 T00723 |
| 1597 | Lactobacillus casei LC2W T01956 |
| 1597 | Lactobacillus casei LOCK919 T02729 |
| 1597 | Lactobacillus casei W56 T02337 |
| 1423822 | Lactobacillus coryniformis subsp torquens DSM 20004 KCTC 3535 T05341 |
| 748671 | Lactobacillus crispatus ST1 T01222 |
| 392416 | Lactobacillus crustorum T04712 |
| 1138822 | Lactobacillus curieae T04707 |
| 28038 | Lactobacillus curvatus T04808 |
| 353496 | Lactobacillus delbrueckii subsp bulgaricus 2038 T01957 |
| 390333 | Lactobacillus delbrueckii subsp bulgaricus ATCC 11842 JCM 1002 T00364 |
| 321956 | Lactobacillus delbrueckii subsp bulgaricus ATCC BAA 365 T00419 |
| 767455 | Lactobacillus delbrueckii subsp bulgaricus ND02 T01360 |
| 712938 | Lactobacillus fermentum CECT 5716 T01958 |
| 767453 | Lactobacillus fermentum F 6 T02669 |
| 334390 | Lactobacillus fermentum IFO 3956 T00699 |
| 52242 | Lactobacillus gallinarum T04115 |
| 324831 | Lactobacillus gasseri ATCC 33323 JCM 1131 T00410 |
| 1007676 | Lactobacillus ginsenosidimutans T03990 |
| 1074467 | Lactobacillus heilongjiangensis T04057 |
| 326425 | Lactobacillus helveticus CNRZ32 T02744 |
| 405566 | Lactobacillus helveticus DPC 4571 T00625 |
| 767462 | Lactobacillus helveticus H10 T01959 |
| 767456 | Lactobacillus helveticus H9 T03040 |
| 880633 | Lactobacillus helveticus R0052 T02217 |
| 1587 | Lactobacillus helveticus T03777 |
| 1291742 | Lactobacillus hokkaidonensis JCM 18461 T03598 |
| 109790 | Lactobacillus jensenii T04677 |
| 909954 | Lactobacillus johnsonii DPC 6026 T01960 |
| 633699 | Lactobacillus johnsonii FI9785 T01120 |
| 1408186 | Lactobacillus johnsonii N6 2 T02941 |
| 257314 | Lactobacillus johnsonii NCC 533 T00158 |
| 190905 | Lactobacillus kefiranofaciens ZW3 T01526 |
| 637971 | Lactobacillus koreensis T03995 |
| 148814 | Lactobacillus kunkeei T04112 |
| 53444 | Lactobacillus lindneri T04666 |
| 1130798 | Lactobacillus mucosae LM1 T03814 |
| 1632 | Lactobacillus oris T04391 |
| 152331 | Lactobacillus parabuchneri T04617 |
| 321967 | Lactobacillus paracasei ATCC 334 T00407 |
| 1446494 | Lactobacillus paracasei N1115 T03050 |
| 537973 | Lactobacillus paracasei subsp paracasei 8700 2 T02808 |
| 47714 | Lactobacillus paracasei subsp paracasei JCM 8130 T04768 |
| 1597 | Lactobacillus paracasei T01280 |
| 240427 | Lactobacillus paracollinoides T04675 |
| 2107999 | Lactobacillus paragasseri T05840 |
| 60520 | Lactobacillus paraplantarum T04156 |
| 1589 | Lactobacillus pentosus T05251 |
| 1327988 | Lactobacillus plantarum 16 T02698 |
| 644042 | Lactobacillus plantarum JDM1 T00946 |
| 767468 | Lactobacillus plantarum subsp plantarum P 8 T02670 |
| 889932 | Lactobacillus plantarum subsp plantarum ST III T01324 |
| 1590 | Lactobacillus plantarum T03680 |
| 220668 | Lactobacillus plantarum WCFS1 T00115 |
| 1284663 | Lactobacillus plantarum ZJ316 T02458 |
| 557436 | Lactobacillus reuteri DSM 20016 T00535 |
| 1340495 | Lactobacillus reuteri I5007 T02701 |
| 557433 | Lactobacillus reuteri JCM 1112 T00735 |
| 491077 | Lactobacillus reuteri SD2112 T01551 |
| 1358027 | Lactobacillus reuteri TD1 T02745 |
| 1088720 | Lactobacillus rhamnosus ATCC 8530 T01961 |
| 568703 | Lactobacillus rhamnosus GG T00987 |
| 568703 | Lactobacillus rhamnosus GG T02100 |
| 568704 | Lactobacillus rhamnosus Lc 705 T00990 |
| 1316933 | Lactobacillus rhamnosus LOCK900 T02730 |
| 1318634 | Lactobacillus rhamnosus LOCK908 T02731 |
| 1069534 | Lactobacillus ruminis ATCC 27782 T01626 |
| 314315 | Lactobacillus sakei subsp sakei 23K T00299 |
| 712961 | Lactobacillus salivarius CECT 5713 T01962 |
| 1624 | Lactobacillus salivarius T03340 |
| 362948 | Lactobacillus salivarius UCC118 T00337 |
| 714313 | Lactobacillus sanfranciscensis TMW 1 1304 T01627 |
| 1545702 | Lactobacillus sp wkB8 T03355 |
| 267363 | Lactobacillus zymae T05206 |
| 420889 | Lactococcus garvieae ATCC 49156 T01618 |
| 420890 | Lactococcus garvieae Lg2 T01963 |
| 1104322 | Lactococcus lactis subsp cremoris A76 T01965 |
| 1295826 | Lactococcus lactis subsp cremoris KW2 T02848 |
| 416870 | Lactococcus lactis subsp cremoris MG1363 T00475 |
| 746361 | Lactococcus lactis subsp cremoris NZ9000 T02131 |
| 272622 | Lactococcus lactis subsp cremoris SK11 T00420 |
| 1111678 | Lactococcus lactis subsp cremoris UC509 9 T02415 |
| 929102 | Lactococcus lactis subsp lactis CV56 T01964 |
| 272623 | Lactococcus lactis subsp lactis Il1403 T00045 |
| 1046624 | Lactococcus lactis subsp lactis IO 1 T02485 |
| 684738 | Lactococcus lactis subsp lactis KF147 T01141 |
| 1399116 | Lactococcus lactis subsp lactis KLDS 4 0325 T02882 |
| 1117941 | Lactococcus lactis subsp lactis NCDO 2118 T03437 |
| 1358 | Lactococcus lactis T03480 |
| 297352 | Lactococcus piscium MKFS47 T03909 |
| 1366 | Lactococcus raffinolactis T05231 |
| 2419773 | Lactococcus sp 1JSPR 7 T05686 |
| 4236 | Lactuca sativa T05352 |
| 1838286 | Lacunisphaera limnophila T04567 |
| 557598 | Laribacter hongkongensis HLHK9 T00884 |
| 215358 | Larimichthys crocea T04659 |
| 8187 | Lates calcarifer T04895 |
| 7897 | Latimeria chalumnae T02913 |
| 47671 | Lautropia mirabilis T05799 |
| 1528099 | Lawsonella clevelandensis T04080 |
| 1234378 | Lawsonia intracellularis N343 T02437 |
| 363253 | Lawsonia intracellularis PHE MN1 00 T00354 |
| 649349 | Leadbetterella byssophila DSM 17132 T01350 |
| 83655 | Leclercia adecarboxylata T04232 |
| 1920114 | Leclercia sp LSNIH1 T05297 |
| 1920116 | Leclercia sp LSNIH3 T05316 |
| 1867846 | Legionella clemsonensis T05016 |
| 2005262 | Legionella endosymbiont of Polyplax serrata T05191 |
| 1212491 | Legionella fallonii LLAP 10 T03771 |
| 449 | Legionella hackeliae T03809 |
| 45067 | Legionella lansingensis T05543 |
| 661367 | Legionella longbeachae NSW150 T01183 |
| 1268635 | Legionella oakridgensis ATCC 33761 DSM 21215 T03928 |
| 423212 | Legionella pneumophila 2300 99 Alcoy T01232 |
| 400673 | Legionella pneumophila str Corby T00532 |
| 297245 | Legionella pneumophila str Lens T00208 |
| 297246 | Legionella pneumophila str Paris T00209 |
| 933093 | Legionella pneumophila subsp pneumophila ATCC 43290 T01726 |
| 1312904 | Legionella pneumophila subsp pneumophila LPE509 T02511 |
| 272624 | Legionella pneumophila subsp pneumophila str Philadelphia 1 T00199 |
| 1199191 | Legionella pneumophila subsp pneumophila str Thunder Bay T02689 |
| 91891 | Legionella pneumophila subsp pneumophila T02174 |
| 91891 | Legionella pneumophila subsp pneumophila T02175 |
| 28087 | Legionella sainthelensi T05293 |
| 1389489 | Leifsonia xyli subsp cynodontis DSM 46306 T02852 |
| 281090 | Leifsonia xyli subsp xyli str CTCB07 T00190 |
| 420245 | Leishmania braziliensis MHOM BR 75 M2904 T01113 |
| 5661 | Leishmania donovani T02289 |
| 435258 | Leishmania infantum JPCM5 T01112 |
| 347515 | Leishmania major strain Friedlin T01014 |
| 929439 | Leishmania mexicana MHOM GT 2001 U1103 T02288 |
| 999552 | Leisingera methylohalidivorans DSM 14336 T02961 |
| 2508307 | Leisingera sp NJS204 T05817 |
| 61646 | Lelliottia amnigena T04400 |
| 1907578 | Lelliottia jeotgali T05035 |
| 2836161 | Lelliottia nimipressuralis T05333 |
| 2153385 | Lelliottia sp WB101 T05398 |
| 158841 | Leminorella richardii T05503 |
| 1472767 | Lentibacillus amyloliquefaciens T04204 |
| 1586287 | Lentzea guizhouensis T04448 |
| 411966 | Leptolyngbya boryana IAM M 101 T04848 |
| 1752064 | Leptolyngbya sp NIES 3755 T04199 |
| 1080068 | Leptolyngbya sp O 77 T04251 |
| 111781 | Leptolyngbya sp PCC 7376 T02361 |
| 2564040 | Leptospira alstonii T04558 |
| 456481 | Leptospira biflexa serovar Patoc strain Patoc 1 T00693 |
| 355278 | Leptospira biflexa serovar Patoc strain Patoc 1 T00712 |
| 355277 | Leptospira borgpetersenii serovar Hardjo bovis str JB197 T00404 |
| 355276 | Leptospira borgpetersenii serovar Hardjo bovis str L550 T00405 |
| 267671 | Leptospira interrogans serovar Copenhageni str Fiocruz L1 130 T00166 |
| 189518 | Leptospira interrogans serovar Lai str 56601 T00098 |
| 573825 | Leptospira interrogans serovar Lai str IPAV T01983 |
| 1395589 | Leptospira interrogans serovar Linhai str 56609 T03859 |
| 408139 | Leptospira kmetyi T05705 |
| 1137606 | Leptospira mayottensis T05598 |
| 758847 | Leptospira santarosai serovar Shermani str LT 821 T03999 |
| 1048260 | Leptospirillum ferriphilum ML 04 T02319 |
| 1441628 | Leptospirillum ferriphilum YSK T03197 |
| 1162668 | Leptospirillum ferrooxidans C2 3 T01809 |
| 1660083 | Leptospirillum sp Group II CF 1 T04007 |
| 395495 | Leptothrix cholodnii SP 6 T00681 |
| 523794 | Leptotrichia buccalis C 1013 b T00975 |
| 712357 | Leptotrichia sp oral taxon 212 T04044 |
| 712368 | Leptotrichia sp oral taxon 498 T04995 |
| 1785996 | Leptotrichia sp oral taxon 847 T04653 |
| 1935379 | Leucobacter sp DSM 101948 T05802 |
| 1784719 | Leucobacter triazinivorans T05843 |
| 1229758 | Leuconostoc carnosum JB16 T02321 |
| 349519 | Leuconostoc citreum KM20 T00663 |
| 255248 | Leuconostoc garlicum T04802 |
| 1229756 | Leuconostoc gelidum JB7 T02322 |
| 762550 | Leuconostoc gelidum subsp gasicomitatum LMG 18811 T01267 |
| 762051 | Leuconostoc kimchii IMSNU 11154 T01233 |
| 1246 | Leuconostoc lactis T04460 |
| 427140 | Leuconostoc mesenteroides KFRI MG T02997 |
| 203120 | Leuconostoc mesenteroides subsp mesenteroides ATCC 8293 T00411 |
| 1107880 | Leuconostoc mesenteroides subsp mesenteroides J18 T01768 |
| 979982 | Leuconostoc sp C2 T01567 |
| 1511761 | Leuconostoc suionicum T05489 |
| 1215343 | Liberibacter crescens BT 1 T02395 |
| 1851148 | Limihaloglobus sulfuriphilus T04749 |
| 2172103 | Limnobaculum parvum T05491 |
| 1555112 | Limnochorda pilosa T04150 |
| 1678129 | Limnohabitans sp 103DPR2 T04120 |
| 1678128 | Limnohabitans sp 63ED37 2 T04121 |
| 83485 | Linepithema humile T04898 |
| 7574 | Lingula anatina T04921 |
| 118797 | Lipotes vexillifer T03090 |
| 272626 | Listeria innocua Clip11262 T00067 |
| 881621 | Listeria ivanovii subsp ivanovii PAM 55 T01623 |
| 202751 | Listeria ivanovii subsp ivanovii T03400 |
| 202752 | Listeria ivanovii subsp londoniensis T03401 |
| 202752 | Listeria ivanovii subsp londoniensis T03402 |
| 1457190 | Listeria ivanovii WSLC3009 T03045 |
| 1126011 | Listeria monocytogenes 07PF0776 T02040 |
| 653938 | Listeria monocytogenes 08 5578 T01157 |
| 637381 | Listeria monocytogenes 08 5923 T01164 |
| 393133 | Listeria monocytogenes 10403S T01984 |
| 1288295 | Listeria monocytogenes 6179 T03159 |
| 882095 | Listeria monocytogenes ATCC 19117 T02239 |
| 169963 | Listeria monocytogenes EGD e T00066 |
| 1334565 | Listeria monocytogenes EGD T02872 |
| 393127 | Listeria monocytogenes Finland 1998 T01986 |
| 393126 | Listeria monocytogenes FSL R2 561 T01985 |
| 552536 | Listeria monocytogenes HCC23 T00801 |
| 393130 | Listeria monocytogenes J0161 T01987 |
| 930782 | Listeria monocytogenes J1 220 T02801 |
| 930781 | Listeria monocytogenes J1816 T02776 |
| 882094 | Listeria monocytogenes L312 T02247 |
| 563174 | Listeria monocytogenes L99 T01989 |
| 1234141 | Listeria monocytogenes La111 T02512 |
| 1030009 | Listeria monocytogenes M7 T01988 |
| 1234142 | Listeria monocytogenes N53 1 T02513 |
| 1437838 | Listeria monocytogenes R479a T03180 |
| 568819 | Listeria monocytogenes serotype 4b str CLIP 80459 T00898 |
| 265669 | Listeria monocytogenes serotype 4b str F2365 T00174 |
| 1230340 | Listeria monocytogenes serotype 4b str LL195 T02416 |
| 863767 | Listeria monocytogenes serotype 7 str SLCC2482 T02221 |
| 932920 | Listeria monocytogenes SLCC2372 T02220 |
| 882097 | Listeria monocytogenes SLCC2376 T02222 |
| 879088 | Listeria monocytogenes SLCC2378 T02240 |
| 882020 | Listeria monocytogenes SLCC2479 T02241 |
| 879089 | Listeria monocytogenes SLCC2540 T02242 |
| 932919 | Listeria monocytogenes SLCC2755 T02219 |
| 882096 | Listeria monocytogenes SLCC5850 T02223 |
| 879090 | Listeria monocytogenes SLCC7179 T02224 |
| 1639 | Listeria monocytogenes T03477 |
| 1639 | Listeria monocytogenes T03478 |
| 1639 | Listeria monocytogenes T03479 |
| 1457187 | Listeria monocytogenes WSLC1001 T03114 |
| 1457188 | Listeria monocytogenes WSLC1042 T03151 |
| 683837 | Listeria seeligeri serovar 1 2b str SLCC3954 T01184 |
| 1006155 | Listeria weihenstephanensis T05637 |
| 386043 | Listeria welshimeri serovar 6b str SLCC5334 T00422 |
| 718192 | Litorilituus sediminis T05852 |
| 7209 | Loa loa T02664 |
| 379508 | Lodderomyces elongisporus NRRL YB 4239 T01116 |
| 1538547 | Lokiarchaeum sp GC14 75 T04015 |
| 1082704 | Lonsdalea britannica T05623 |
| 225164 | Lottia gigantea T03273 |
| 34305 | Lotus japonicus T04348 |
| 9785 | Loxodonta africana T04351 |
| 3871 | Lupinus angustifolius T04664 |
| 1440763 | Luteibacter rhizovicinus DSM 16549 T04563 |
| 2006110 | Luteimonas sp 100111 T05077 |
| 571913 | Luteipulveratus mongoliensis T04024 |
| 1855912 | Luteitalea pratensis T04394 |
| 1622118 | Lutibacter profundi T04235 |
| 28031 | Lysinibacillus fusiformis T03655 |
| 2169540 | Lysinibacillus sp 2017 T05455 |
| 2081964 | Lysinibacillus sp B2A1 T05347 |
| 2072025 | Lysinibacillus sp YS11 T05299 |
| 444177 | Lysinibacillus sphaericus C3 41 T00656 |
| 1145276 | Lysinibacillus varians T03062 |
| 84531 | Lysobacter antibioticus T04154 |
| 84531 | Lysobacter antibioticus T04526 |
| 435897 | Lysobacter capsici T04162 |
| 69 | Lysobacter enzymogenes T04371 |
| 69 | Lysobacter enzymogenes T04598 |
| 262324 | Lysobacter gummosus T04163 |
| 1605891 | Lysobacter maris T05495 |
| 2698682 | Lysobacter oculi T05527 |
| 2290922 | Lysobacter sp TY2 98 T05586 |
| 9541 | Macaca fascicularis T02918 |
| 9544 | Macaca mulatta T01028 |
| 1855823 | Macrococcus canis T04838 |
| 458233 | Macrococcus caseolyticus JCSC5402 T00848 |
| 1898474 | Macrococcus sp IME1552 T05070 |
| 699246 | Mageeibacillus indolicus UPII9 5 T01181 |
| 156889 | Magnetococcus marinus MC 1 T00427 |
| 1288970 | Magnetospira sp QH 2 T03795 |
| 431944 | Magnetospirillum gryphiswaldense MSR 1 T05662 |
| 1430440 | Magnetospirillum gryphiswaldense MSR 1 v2 T02960 |
| 342108 | Magnetospirillum magneticum AMB 1 T00305 |
| 1639348 | Magnetospirillum sp ME 1 T04817 |
| 1663591 | Magnetospirillum sp XM 1 T04231 |
| 697281 | Mahella australiensis 50 1 BON T01494 |
| 202462 | Maize bushy stunt phytoplasma T04456 |
| 425265 | Malassezia globosa CBS 7966 T01043 |
| 3750 | Malus domestica T03194 |
| 3983 | Manihot esculenta T05761 |
| 1261126 | Mannheimia haemolytica D153 T02746 |
| 1311759 | Mannheimia haemolytica D171 T02747 |
| 1311760 | Mannheimia haemolytica D174 T02748 |
| 1316932 | Mannheimia haemolytica M42548 T02635 |
| 75985 | Mannheimia haemolytica T03749 |
| 75985 | Mannheimia haemolytica T03750 |
| 1249531 | Mannheimia haemolytica USDA ARS USMARC 183 T02523 |
| 1222034 | Mannheimia haemolytica USDA ARS USMARC 184 T03786 |
| 1249526 | Mannheimia haemolytica USDA ARS USMARC 185 T02522 |
| 1366053 | Mannheimia haemolytica USMARC 2286 T02758 |
| 1432056 | Mannheimia sp USDA ARS USMARC 1261 T03026 |
| 221988 | Mannheimia succiniciproducens MBEL55E T00201 |
| 1433287 | Mannheimia varigena USDA ARS USMARC 1296 T03027 |
| 1434214 | Mannheimia varigena USDA ARS USMARC 1312 T03028 |
| 1434215 | Mannheimia varigena USDA ARS USMARC 1388 T03029 |
| 1178778 | Maribacter cobaltidurans T05023 |
| 1644130 | Maribacter sp 1 2014MBL MicDiv T04570 |
| 313603 | Maribacter sp HTCC2170 T01306 |
| 2496865 | Maribacter sp MJ134 T05877 |
| 1836467 | Maribacter sp T28 T04597 |
| 394221 | Maricaulis maris MCS10 T00394 |
| 765910 | Marichromatium purpuratum 984 T03650 |
| 2027857 | Mariniflexile sp TRM1 10 T05592 |
| 1911586 | Marinilactibacillus sp 15R T04573 |
| 869210 | Marinithermus hydrothermalis DSM 14884 T01457 |
| 443254 | Marinitoga piezophila KA3 T01665 |
| 1545835 | Marinitoga sp 1137 T04633 |
| 225937 | Marinobacter adhaerens HP15 T01922 |
| 1163748 | Marinobacter hydrocarbonoclasticus ATCC 49840 T02083 |
| 351348 | Marinobacter hydrocarbonoclasticus VT8 T00454 |
| 330734 | Marinobacter psychrophilus T03980 |
| 1420917 | Marinobacter salarius T03593 |
| 1874317 | Marinobacter salinus T04766 |
| 1420916 | Marinobacter similis T03594 |
| 2304594 | Marinobacter sp Arc7 DN 1 T05619 |
| 490759 | Marinobacter sp BSs20148 T02323 |
| 1671721 | Marinobacter sp CP1 T04052 |
| 1749259 | Marinobacter sp LQ44 T04470 |
| 1821621 | Marinobacterium aestuarii T04398 |
| 717774 | Marinomonas mediterranea MMB 1 T01449 |
| 491952 | Marinomonas posidonica IVIA Po 181 T01508 |
| 400668 | Marinomonas sp MWYL1 T00567 |
| 988812 | Marinovum algicola DG 898 T03981 |
| 1921086 | Mariprofundus aestuarium T05212 |
| 1921087 | Mariprofundus ferrinatatus T05213 |
| 454601 | Maritalea myrionectae T05588 |
| 643867 | Marivirga tractuosa DSM 4126 T01365 |
| 1920883 | Marivivens sp JLT3646 T04589 |
| 1072389 | Marssonina brunnea f sp multigermtubi MB m1 T03105 |
| 1486262 | Martelella endophytica T03801 |
| 1122214 | Martelella mediterranea DSM 17316 T04757 |
| 686597 | Martelella sp AD 3 T04332 |
| 2072590 | Massilia armeniaca T05378 |
| 945844 | Massilia oculi T05448 |
| 1678028 | Massilia sp NR 4 1 T04026 |
| 1707785 | Massilia sp WG5 T04122 |
| 1593482 | Massilia sp YMA4 T05528 |
| 2045208 | Massilia violaceinigra T05157 |
| 106582 | Maylandia zebra T02814 |
| 3880 | Medicago truncatula T01716 |
| 657316 | Megamonas hypermegale ART12 1 T02593 |
| 1064535 | Megasphaera elsdenii DSM 20460 T01633 |
| 1675036 | Megasphaera hexanoica T05536 |
| 2144175 | Megasphaera stantonii T05599 |
| 504728 | Meiothermus ruber DSM 1279 T01193 |
| 504728 | Meiothermus ruber DSM 1279 T02636 |
| 526227 | Meiothermus silvanus DSM 9946 T01246 |
| 1339250 | Meiothermus taiwanensis WR 220 T05633 |
| 2109913 | Melaminivora sp SC2 9 T05358 |
| 747676 | Melampsora larici populina 98AG31 T03138 |
| 9103 | Meleagris gallopavo T01523 |
| 1191523 | Melioribacter roseus P3M 2 T02176 |
| 940190 | Melissococcus plutonius ATCC 35311 T01492 |
| 1090974 | Melissococcus plutonius DAT561 T01763 |
| 1294270 | Melittangium boletus DSM 14713 T05066 |
| 216427 | Mesoplasma chauliocola T05062 |
| 324078 | Mesoplasma coleopterae T05286 |
| 2149 | Mesoplasma entomophilum T05159 |
| 265311 | Mesoplasma florum L1 T00187 |
| 1406864 | Mesoplasma florum W37 T02878 |
| 81460 | Mesoplasma lactucae ATCC 49193 T05127 |
| 225999 | Mesoplasma syrphidae T05255 |
| 219745 | Mesoplasma tabanidae T05273 |
| 1082933 | Mesorhizobium amorphae CCNWGS0123 T04446 |
| 754035 | Mesorhizobium australicum WSM2073 T02417 |
| 765698 | Mesorhizobium ciceri biovar biserrulae WSM1271 T01401 |
| 266835 | Mesorhizobium japonicum MAFF 303099 T00043 |
| 935546 | Mesorhizobium loti NZP2037 T04711 |
| 1670800 | Mesorhizobium oceanicum T04603 |
| 536019 | Mesorhizobium opportunistum WSM2075 T01542 |
| 278153 | Mesorhizobium sp WSM1497 T04915 |
| 1236046 | Mesotoga infera T05565 |
| 660470 | Mesotoga prima MesG1 Ag 4 2 T02123 |
| 2487150 | Metakosakonia sp MRY16 398 T05735 |
| 1006006 | Metallosphaera cuprina Ar 4 T01467 |
| 1293036 | Metallosphaera hakonensis JCM 8857 DSM 7519 T05471 |
| 399549 | Metallosphaera sedula DSM 5348 T00509 |
| 92637 | Metarhizium acridum CQMa 102 T03104 |
| 655844 | Metarhizium robertsii ARSEF 23 T03107 |
| 118062 | Methanobacterium congolense T04503 |
| 2162 | Methanobacterium formicicum T03396 |
| 2162 | Methanobacterium formicicum T03743 |
| 877455 | Methanobacterium lacus T01437 |
| 868131 | Methanobacterium paludis T01509 |
| 2025351 | Methanobacterium sp BAmetb5 T05745 |
| 2025350 | Methanobacterium sp BRmetb2 T05692 |
| 1379702 | Methanobacterium sp MB1 T02957 |
| 1911685 | Methanobacterium sp MZ A1 T05317 |
| 59277 | Methanobacterium subterraneum T05275 |
| 230361 | Methanobrevibacter millerae T04203 |
| 294671 | Methanobrevibacter olleyae T04385 |
| 634498 | Methanobrevibacter ruminantium M1 T01166 |
| 420247 | Methanobrevibacter smithii ATCC 35061 T00539 |
| 224719 | Methanobrevibacter sp AbM4 T02687 |
| 1609968 | Methanobrevibacter sp YE315 T04279 |
| 1301915 | Methanocaldococcus bathoardescens T03253 |
| 573064 | Methanocaldococcus fervens AG86 T00976 |
| 573063 | Methanocaldococcus infernus ME T01234 |
| 243232 | Methanocaldococcus jannaschii DSM 2661 T00003 |
| 644281 | Methanocaldococcus sp FS406 22 T01175 |
| 579137 | Methanocaldococcus vulcanius M7 T01105 |
| 351160 | Methanocella arvoryzae MRE50 T00526 |
| 1041930 | Methanocella conradii HZ254 T01774 |
| 304371 | Methanocella paludicola SANAE T01142 |
| 259564 | Methanococcoides burtonii DSM 6242 T00341 |
| 1434104 | Methanococcoides methylutens MM1 T03913 |
| 419665 | Methanococcus aeolicus Nankai 3 T00552 |
| 402880 | Methanococcus maripaludis C5 T00491 |
| 444158 | Methanococcus maripaludis C6 T00615 |
| 426368 | Methanococcus maripaludis C7 T00553 |
| 267377 | Methanococcus maripaludis S2 T00164 |
| 1053692 | Methanococcus maripaludis X1 T01584 |
| 406327 | Methanococcus vannielii SB T00554 |
| 456320 | Methanococcus voltae A3 T01251 |
| 410358 | Methanocorpusculum labreanum Z T00472 |
| 1201294 | Methanoculleus bourgensis MS2 T02189 |
| 368407 | Methanoculleus marisnigri JR1 T00477 |
| 83986 | Methanoculleus sp MAB1 T04314 |
| 1495144 | Methanogenic archaeon ISO4 H5 T04272 |
| 644295 | Methanohalobium evestigatum Z 7303 T01261 |
| 2177 | Methanohalophilus halophilus T04572 |
| 547558 | Methanohalophilus mahii DSM 5219 T01211 |
| 679926 | Methanolacinia petrolearia DSM 11571 T01312 |
| 1094980 | Methanolobus psychrophilus R15 T02280 |
| 867904 | Methanomethylovorans hollandica DSM 15978 T02418 |
| 190192 | Methanopyrus kandleri AV19 T00078 |
| 456442 | Methanoregula boonei 6A8 T00578 |
| 593750 | Methanoregula formicica SMSP T02392 |
| 1110509 | Methanosaeta harundinacea 6Ac T01990 |
| 679901 | Methanosalsum zhilinae DSM 4017 T01541 |
| 188937 | Methanosarcina acetivorans C2A T00080 |
| 1434106 | Methanosarcina barkeri 227 T03914 |
| 1434107 | Methanosarcina barkeri 3 T04533 |
| 1434108 | Methanosarcina barkeri MS T03838 |
| 269797 | Methanosarcina barkeri str Fusaro T00271 |
| 1434109 | Methanosarcina barkeri str Wiesmoor T03839 |
| 1715806 | Methanosarcina flavescens T05666 |
| 1434110 | Methanosarcina horonobensis HB 1 JCM 15518 T03933 |
| 1434111 | Methanosarcina lacustris Z 7289 T03822 |
| 1434113 | Methanosarcina mazei C16 T04626 |
| 192952 | Methanosarcina mazei Go1 T00082 |
| 213585 | Methanosarcina mazei S 6 T04532 |
| 1236903 | Methanosarcina mazei Tuc01 T02477 |
| 1434118 | Methanosarcina siciliae C2J T03863 |
| 1434119 | Methanosarcina siciliae HI350 T03864 |
| 1434120 | Methanosarcina siciliae T4 M T03865 |
| 1434099 | Methanosarcina sp Kolksee T03821 |
| 1434100 | Methanosarcina sp MTP4 T03850 |
| 1434102 | Methanosarcina sp WH1 T03851 |
| 1434103 | Methanosarcina sp WWM596 T03852 |
| 1434121 | Methanosarcina thermophila CHTI 55 T03891 |
| 523844 | Methanosarcina thermophila TM 1 T03892 |
| 1434123 | Methanosarcina vacuolata Z 761 T03815 |
| 1789762 | Methanosphaera sp BMS T05504 |
| 339860 | Methanosphaera stadtmanae DSM 3091 T00313 |
| 521011 | Methanosphaerula palustris E1 9c T00823 |
| 323259 | Methanospirillum hungatei JF 1 T00325 |
| 79929 | Methanothermobacter marburgensis str Marburg T01297 |
| 866790 | Methanothermobacter sp CaT2 T04009 |
| 2017966 | Methanothermobacter sp EMTCatA1 T05328 |
| 1898379 | Methanothermobacter sp MT 2 T05694 |
| 187420 | Methanothermobacter thermautotrophicus str Delta H T00009 |
| 145261 | Methanothermobacter wolfeii T04587 |
| 647113 | Methanothermococcus okinawensis IH1 T01534 |
| 523846 | Methanothermus fervidus DSM 2088 T01351 |
| 990316 | Methanothrix soehngenii GP6 T01468 |
| 349307 | Methanothrix thermoacetophila PT T00417 |
| 880724 | Methanotorris igneus Kol 5 T01510 |
| 481448 | Methylacidiphilum infernorum V4 T00727 |
| 420662 | Methylibium petroleiphilum PM1 T00470 |
| 2082386 | Methylibium sp Pch M T05827 |
| 265072 | Methylobacillus flagellatus KT T00342 |
| 270351 | Methylobacterium aquaticum T04311 |
| 2051553 | Methylobacterium currus T05184 |
| 2202825 | Methylobacterium durans T05724 |
| 460265 | Methylobacterium nodulans ORS 2060 T00839 |
| 693986 | Methylobacterium oryzae CBMB20 T03299 |
| 418223 | Methylobacterium phyllosphaerae T04968 |
| 426355 | Methylobacterium radiotolerans JCM 2831 T00674 |
| 426117 | Methylobacterium sp 4 46 T00679 |
| 925818 | Methylobacterium sp AMS5 T04283 |
| 2067957 | Methylobacterium sp DM1 T05461 |
| 739141 | Methylobacterium sp XJLW T05508 |
| 1432792 | Methylocaldum marinum T05638 |
| 1384459 | Methyloceanibacter caenitepidi T03636 |
| 395965 | Methylocella silvestris BL2 T00802 |
| 243233 | Methylococcus capsulatus str Bath T00205 |
| 655015 | Methylocystis bryophila T04837 |
| 173366 | Methylocystis rosea T05721 |
| 187303 | Methylocystis sp SC2 T02324 |
| 1091494 | Methylomicrobium alcaliphilum 20Z T01649 |
| 1538553 | Methylomonas denitrificans T04191 |
| 702114 | Methylomonas koyamae T05118 |
| 857087 | Methylomonas methanica MC09 T01511 |
| 1727196 | Methylomonas sp DH 1 T04396 |
| 107637 | Methylomonas sp LW13 T05879 |
| 1930071 | Methylomusa anaerophila T05819 |
| 754477 | Methylophaga frappieri T02056 |
| 754476 | Methylophaga nitratireducenticrescens T02055 |
| 1662285 | Methylophilus sp TWE2 T04002 |
| 272630 | Methylorubrum extorquens AM1 T00921 |
| 440085 | Methylorubrum extorquens CM4 T00809 |
| 661410 | Methylorubrum extorquens DM4 T00947 |
| 419610 | Methylorubrum extorquens PA1 T00640 |
| 441620 | Methylorubrum populi BJ001 T00760 |
| 29429 | Methylorubrum zatmanii T04981 |
| 595536 | Methylosinus trichosporium OB3b T05155 |
| 583345 | Methylotenera mobilis JLW8 T00942 |
| 666681 | Methylotenera versatilis 301 T01247 |
| 1842540 | Methyloversatilis sp RAC08 T04449 |
| 569860 | Methylovirgula ligni T05824 |
| 582744 | Methylovorus glucosetrophus SIP3 4 T00943 |
| 887061 | Methylovorus sp MP688 T01361 |
| 1704499 | Methylovulum psychrotolerans T04929 |
| 294746 | Meyerozyma guilliermondii ATCC 6260 T01150 |
| 856793 | Micavibrio aeruginosavorus ARL 13 T01630 |
| 349215 | Micavibrio aeruginosavorus EPB T02517 |
| 36805 | Microbacterium aurum T04977 |
| 84292 | Microbacterium chocolatum T04340 |
| 104336 | Microbacterium foliorum T05657 |
| 162426 | Microbacterium hominis T05238 |
| 273677 | Microbacterium oleivorans T05832 |
| 300019 | Microbacterium paludicola T04601 |
| 1916917 | Microbacterium sp 1 5R T04912 |
| 1906742 | Microbacterium sp BH 3 3 3 T04827 |
| 1696072 | Microbacterium sp CGR1 T04046 |
| 1714373 | Microbacterium sp No 7 T04102 |
| 1795053 | Microbacterium sp PAMC 28756 T04312 |
| 367477 | Microbacterium sp XT11 T04243 |
| 979556 | Microbacterium testaceum StLB037 T01423 |
| 260552 | Microbulbifer agarilyticus T04709 |
| 1769779 | Microbulbifer aggregans T04560 |
| 359370 | Microbulbifer sp A4B17 T05421 |
| 252514 | Microbulbifer thermotolerans T04393 |
| 279828 | Microcella alkaliphila T05359 |
| 465515 | Micrococcus luteus NCTC 2665 T00918 |
| 1173027 | Microcoleus sp PCC 7113 T02374 |
| 449447 | Microcystis aeruginosa NIES 843 T00647 |
| 1638788 | Microcystis panniformis FACHB 1757 T04037 |
| 1967666 | Microcystis sp MC19 T05365 |
| 1032480 | Microlunatus phosphovorus NM 1 T01535 |
| 296587 | Micromonas commoda T02695 |
| 564608 | Micromonas pusilla CCMP1545 T02696 |
| 644283 | Micromonospora aurantiaca ATCC 27029 T01296 |
| 2201999 | Micromonospora sp B006 T05583 |
| 648999 | Micromonospora sp L5 T01379 |
| 479978 | Micromonospora tulbaghiae T05651 |
| 69319 | Microplitis demolitor T05600 |
| 75385 | Micropruina glycogenica T05338 |
| 412690 | Microterricola viridarii T04252 |
| 1882682 | Microvirga ossetica T05139 |
| 2082949 | Microvirga sp 17 mud 1 3 T05470 |
| 888845 | Minicystis rosea T04613 |
| 1658665 | Mitsuaria sp 7 T04402 |
| 665913 | Mixta calida T05419 |
| 665914 | Mixta gaviniae T05418 |
| 6573 | Mizuhopecten yessoensis T05079 |
| 548479 | Mobiluncus curtisii ATCC 43063 T01257 |
| 2732864 | Modestobacter marinus T02167 |
| 114527 | Mogibacterium diversum T05355 |
| 3673 | Momordica charantia T05045 |
| 554373 | Moniliophthora perniciosa FA553 T01089 |
| 1381753 | Moniliophthora roreri MCA 2997 T03137 |
| 13616 | Monodelphis domestica T01031 |
| 307658 | Monomorium pharaonis T05760 |
| 43700 | Monopterus albus T05607 |
| 145388 | Monoraphidium neglectum T05028 |
| 431895 | Monosiga brevicollis MX1 T01042 |
| 1458985 | Moorea producens PAL 8 15 08 1 T04499 |
| 264732 | Moorella thermoacetica ATCC 39073 T00308 |
| 476 | Moraxella bovis T05535 |
| 386891 | Moraxella bovoculi T04324 |
| 1236608 | Moraxella catarrhalis BBH18 T01224 |
| 480 | Moraxella catarrhalis T03217 |
| 480 | Moraxella catarrhalis T03381 |
| 34062 | Moraxella osloensis T04294 |
| 29433 | Moraxella ovis T04329 |
| 2283318 | Moraxellaceae bacterium HYN0046 T05575 |
| 1124991 | Morganella morganii subsp morganii KT T02496 |
| 80854 | Moritella viscosa T03770 |
| 69539 | Moritella yayanosii T05564 |
| 1550579 | Mucilaginibacter gotjawali T05131 |
| 2305508 | Mucilaginibacter sp HYN0043 T05668 |
| 1300914 | Mucilaginibacter sp PAMC 26640 T04335 |
| 1234841 | Mucilaginibacter xinganensis T05010 |
| 1433126 | Mucinivorans hirudinis T03205 |
| 1796646 | Muribaculum intestinale T04438 |
| 516051 | Muricauda lutaonensis T03884 |
| 886377 | Muricauda ruestringensis DSM 13258 T01598 |
| 2507538 | Muriicola sp MMS17 SY002 T05825 |
| 10090 | Mus musculus T01002 |
| 214687 | Musa acuminata subsp malaccensis T03447 |
| 7370 | Musca domestica T03448 |
| 2079792 | Mycetocola sp 449 T05409 |
| 1553431 | Mycoavidus cysteinexigens T05801 |
| 243243 | Mycobacterium avium 104 T00433 |
| 262316 | Mycobacterium avium subsp paratuberculosis K 10 T00156 |
| 1199187 | Mycobacterium avium subsp paratuberculosis MAP4 T02662 |
| 1770 | Mycobacterium avium subsp paratuberculosis T03787 |
| 1770 | Mycobacterium avium subsp paratuberculosis T03847 |
| 1048245 | Mycobacterium canettii CIPT 140010059 T01585 |
| 1205676 | Mycobacterium canettii CIPT 140060008 T02419 |
| 1205675 | Mycobacterium canettii CIPT 140070008 T02420 |
| 1205674 | Mycobacterium canettii CIPT 140070010 T02421 |
| 1205677 | Mycobacterium canettii CIPT 140070017 T02422 |
| 222805 | Mycobacterium chimaera T04926 |
| 482462 | Mycobacterium dioxanotrophicus T05102 |
| 1202450 | Mycobacterium haemophilum DSM 44634 T04111 |
| 487521 | Mycobacterium intracellulare ATCC 13950 T02158 |
| 1138382 | Mycobacterium intracellulare MOTT 02 T01754 |
| 1232724 | Mycobacterium intracellulare subsp intracellulare MTCC 9506 T02325 |
| 1138871 | Mycobacterium intracellulare subsp yongonense 05 1390 T02732 |
| 557599 | Mycobacterium kansasii ATCC 12478 T02887 |
| 561304 | Mycobacterium leprae Br4923 T00842 |
| 272631 | Mycobacterium leprae TN T00047 |
| 64667 | Mycobacterium lepraemurium T05534 |
| 459424 | Mycobacterium liflandii 128FXT T02444 |
| 1131442 | Mycobacterium marinum E11 T04774 |
| 216594 | Mycobacterium marinum M T00694 |
| 701042 | Mycobacterium marseillense T05049 |
| 1138383 | Mycobacterium paraintracellulare T01755 |
| 722731 | Mycobacterium shigaense T05717 |
| 1545728 | Mycobacterium sp EPa45 T03960 |
| 164757 | Mycobacterium sp JLS T00482 |
| 212767 | Mycobacterium sp JS623 T02423 |
| 189918 | Mycobacterium sp KMS T00442 |
| 164756 | Mycobacterium sp MCS T00367 |
| 1168287 | Mycobacterium sp MOTT36Y T02113 |
| 1273687 | Mycobacterium sp VKM Ac 1817D T03634 |
| 1520670 | Mycobacterium stephanolepidis T05257 |
| 1138877 | Mycobacterium tuberculosis 7199 99 T02617 |
| 1010836 | Mycobacterium tuberculosis BT1 T03152 |
| 1010835 | Mycobacterium tuberculosis BT2 T03153 |
| 1310114 | Mycobacterium tuberculosis CAS NITR204 T02652 |
| 443149 | Mycobacterium tuberculosis CCDC5079 T01991 |
| 443149 | Mycobacterium tuberculosis CCDC5079 T02679 |
| 443150 | Mycobacterium tuberculosis CCDC5180 T01992 |
| 83331 | Mycobacterium tuberculosis CDC1551 T00053 |
| 707235 | Mycobacterium tuberculosis CTRI 2 T01993 |
| 1310115 | Mycobacterium tuberculosis EAI5 NITR206 T02653 |
| 1306414 | Mycobacterium tuberculosis EAI5 T02749 |
| 336982 | Mycobacterium tuberculosis F11 T00545 |
| 419947 | Mycobacterium tuberculosis H37Ra T00540 |
| 83332 | Mycobacterium tuberculosis H37Rv T00015 |
| 83332 | Mycobacterium tuberculosis H37Rv T02178 |
| 1010834 | Mycobacterium tuberculosis HKBS1 T03154 |
| 478434 | Mycobacterium tuberculosis KZN 1435 T00940 |
| 478433 | Mycobacterium tuberculosis KZN 4207 T01690 |
| 478435 | Mycobacterium tuberculosis KZN 605 T02141 |
| 1091500 | Mycobacterium tuberculosis RGTB327 T01775 |
| 1091501 | Mycobacterium tuberculosis RGTB423 T01994 |
| 1306400 | Mycobacterium tuberculosis str Beijing NITR203 T02616 |
| 652616 | Mycobacterium tuberculosis str Erdman ATCC 35801 T02526 |
| 1304279 | Mycobacterium tuberculosis str Haarlem NITR202 T02654 |
| 395095 | Mycobacterium tuberculosis str Haarlem T02845 |
| 1097669 | Mycobacterium tuberculosis UT205 T02142 |
| 572418 | Mycobacterium tuberculosis variant africanum GM041182 T01575 |
| 233413 | Mycobacterium tuberculosis variant bovis AF2122 97 T00132 |
| 998092 | Mycobacterium tuberculosis variant bovis BCG str ATCC 35743 T03796 |
| 1206780 | Mycobacterium tuberculosis variant bovis BCG str Korea 1168P T02451 |
| 717522 | Mycobacterium tuberculosis variant bovis BCG str Mexico T01727 |
| 410289 | Mycobacterium tuberculosis variant bovis BCG str Pasteur 1173P2 T00463 |
| 561275 | Mycobacterium tuberculosis variant bovis BCG str Tokyo 172 T00864 |
| 1806 | Mycobacterium tuberculosis variant microti T04291 |
| 362242 | Mycobacterium ulcerans Agy99 T00435 |
| 561007 | Mycobacteroides abscessus ATCC 19977 T00657 |
| 1303024 | Mycobacteroides abscessus subsp bolletii 50594 T02682 |
| 1001714 | Mycobacteroides abscessus subsp massiliense CCUG 48898 JCM 15300 T03694 |
| 1198627 | Mycobacteroides abscessus subsp massiliense str GO 06 T02177 |
| 1460372 | Mycobacteroides chelonae CCUG 47445 T04665 |
| 83262 | Mycobacteroides immunogenum T04755 |
| 1578165 | Mycobacteroides saopaulense T05445 |
| 875328 | Mycolicibacter sinensis T01512 |
| 1788 | Mycolicibacter terrae T05465 |
| 710421 | Mycolicibacterium chubuense NBB4 T02150 |
| 1766 | Mycolicibacterium fortuitum T04105 |
| 350054 | Mycolicibacterium gilvum PYR GCK T00498 |
| 278137 | Mycolicibacterium gilvum Spyr1 T01376 |
| 134601 | Mycolicibacterium goodii T04008 |
| 1122247 | Mycolicibacterium hassiacum DSM 44199 T05715 |
| 758802 | Mycolicibacterium litorale T05032 |
| 700508 | Mycolicibacterium neoaurum VKM Ac 1815D T02959 |
| 1771 | Mycolicibacterium phlei T04305 |
| 710685 | Mycolicibacterium rhodesiae NBB3 T01685 |
| 246196 | Mycolicibacterium smegmatis MC2 155 T00434 |
| 246196 | Mycolicibacterium smegmatis MC2 155 T02191 |
| 246196 | Mycolicibacterium smegmatis MC2 155 T03433 |
| 1772 | Mycolicibacterium smegmatis T03409 |
| 1772 | Mycolicibacterium smegmatis T03432 |
| 1797 | Mycolicibacterium thermoresistibile T05542 |
| 1354275 | Mycolicibacterium vaccae 95051 T04356 |
| 350058 | Mycolicibacterium vanbaalenii PYR 1 T00449 |
| 347257 | Mycoplasma agalactiae PG2 T00536 |
| 2110 | Mycoplasma agalactiae T01192 |
| 2094 | Mycoplasma arginini T04176 |
| 243272 | Mycoplasma arthritidis 158L3 1 T00724 |
| 2112 | Mycoplasma bovigenitalium T05188 |
| 1316930 | Mycoplasma bovis CQ W70 T03268 |
| 767465 | Mycoplasma bovis HB0801 T02143 |
| 956483 | Mycoplasma bovis Hubei 1 T01568 |
| 289397 | Mycoplasma bovis PG45 T01366 |
| 743966 | Mycoplasma bovoculi M165 69 T03034 |
| 1397850 | Mycoplasma californicum HAZ160 1 T03651 |
| 2113 | Mycoplasma californicum T03247 |
| 29554 | Mycoplasma canadense T03615 |
| 1117644 | Mycoplasma canis PG 14 T04557 |
| 29555 | Mycoplasma canis T03936 |
| 340047 | Mycoplasma capricolum subsp capricolum ATCC 27343 T00309 |
| 1124992 | Mycoplasma capricolum subsp capripneumoniae 87001 T03705 |
| 40480 | Mycoplasma capricolum subsp capripneumoniae T03697 |
| 40480 | Mycoplasma capricolum subsp capripneumoniae T03703 |
| 40480 | Mycoplasma capricolum subsp capripneumoniae T03704 |
| 572263 | Mycoplasma conjunctivae HRC 581 T00919 |
| 512564 | Mycoplasma crocodyli MP145 T01205 |
| 1246955 | Mycoplasma cynos C142 T02424 |
| 86660 | Mycoplasma dispar T03700 |
| 637387 | Mycoplasma fermentans JER T01325 |
| 943945 | Mycoplasma fermentans M64 T01402 |
| 496833 | Mycoplasma fermentans PG18 T02594 |
| 743971 | Mycoplasma flocculare ATCC 27399 T03538 |
| 29556 | Mycoplasma gallinaceum T03898 |
| 1159203 | Mycoplasma gallisepticum CA06 2006 052 5 2P T02326 |
| 1159202 | Mycoplasma gallisepticum NC06 2006 080 5 2P T02200 |
| 1159204 | Mycoplasma gallisepticum NC08 2008 031 4 3P T02327 |
| 1159198 | Mycoplasma gallisepticum NC95 13295 2 2P T02201 |
| 1159199 | Mycoplasma gallisepticum NC96 1596 4 2P T02328 |
| 1159200 | Mycoplasma gallisepticum NY01 2001 047 5 1P T02202 |
| 1006581 | Mycoplasma gallisepticum S6 T02953 |
| 708616 | Mycoplasma gallisepticum str F T01924 |
| 710127 | Mycoplasma gallisepticum str R T00131 |
| 710128 | Mycoplasma gallisepticum str R T01923 |
| 1159197 | Mycoplasma gallisepticum VA94 7994 1 7P T02203 |
| 1159201 | Mycoplasma gallisepticum WI01 2001 043 13 2P T02204 |
| 243273 | Mycoplasma genitalium G37 T00002 |
| 662947 | Mycoplasma genitalium M2288 T02208 |
| 663918 | Mycoplasma genitalium M2321 T02209 |
| 662946 | Mycoplasma genitalium M6282 T02210 |
| 662945 | Mycoplasma genitalium M6320 T02211 |
| 1111676 | Mycoplasma haemocanis str Illinois T01666 |
| 859194 | Mycoplasma haemofelis Ohio2 T01944 |
| 941640 | Mycoplasma haemofelis str Langford 1 T01418 |
| 347256 | Mycoplasma hominis ATCC 23114 T01122 |
| 1267000 | Mycoplasma hominis ATCC 27545 T03410 |
| 1116211 | Mycoplasma hyopneumoniae 168 L T02675 |
| 907287 | Mycoplasma hyopneumoniae 168 T01925 |
| 295358 | Mycoplasma hyopneumoniae 232 T00206 |
| 754503 | Mycoplasma hyopneumoniae 7422 T02750 |
| 262722 | Mycoplasma hyopneumoniae 7448 T00264 |
| 262719 | Mycoplasma hyopneumoniae J T00260 |
| 634997 | Mycoplasma hyorhinis DBS 1050 T02927 |
| 1129369 | Mycoplasma hyorhinis GDL 1 T01730 |
| 872331 | Mycoplasma hyorhinis HUB 1 T01301 |
| 936139 | Mycoplasma hyorhinis MCLD T01945 |
| 1118964 | Mycoplasma hyorhinis SK76 T02351 |
| 29559 | Mycoplasma hyosynoviae T05673 |
| 866629 | Mycoplasma leachii 99 014 6 T02144 |
| 880447 | Mycoplasma leachii PG50 T01367 |
| 267748 | Mycoplasma mobile 163K T00176 |
| 862259 | Mycoplasma mycoides subsp capri LC str 95010 T01478 |
| 865867 | Mycoplasma mycoides subsp mycoides SC str Gladysdale T02595 |
| 272632 | Mycoplasma mycoides subsp mycoides SC str PG1 T00161 |
| 2103 | Mycoplasma mycoides subsp mycoides T03541 |
| 1415773 | Mycoplasma ovis str Michigan T02962 |
| 1403316 | Mycoplasma parvum str Indiana T02873 |
| 272633 | Mycoplasma penetrans HF 2 T00107 |
| 142651 | Mycoplasma phocidae T05577 |
| 1112856 | Mycoplasma pneumoniae 309 T01712 |
| 722438 | Mycoplasma pneumoniae FH T01926 |
| 1238993 | Mycoplasma pneumoniae M129 B7 T02596 |
| 272634 | Mycoplasma pneumoniae M129 T00006 |
| 48003 | Mycoplasma pullorum T05055 |
| 272635 | Mycoplasma pulmonis UAB CTIP T00055 |
| 743965 | Mycoplasma putrefaciens KS1 T01599 |
| 1292033 | Mycoplasma putrefaciens Mput9231 T02637 |
| 1749074 | Mycoplasma sp T04239 |
| 708248 | Mycoplasma suis KI3806 T01442 |
| 768700 | Mycoplasma suis str Illinois T01438 |
| 262723 | Mycoplasma synoviae 53 T00261 |
| 1267001 | Mycoplasma synoviae ATCC 25204 T04518 |
| 1197325 | Mycoplasma wenyonii str Massachusetts T02179 |
| 743967 | Mycoplasma yeatsii GM274B T03676 |
| 109478 | Myotis brandtii T02920 |
| 225400 | Myotis davidii T02992 |
| 76832 | Myroides odoratimimus T04190 |
| 480520 | Myroides profundi T03631 |
| 1583100 | Myroides sp A21 T03521 |
| 1458492 | Myroides sp ZB35 T04543 |
| 35 | Myxococcus fulvus HW 1 T01558 |
| 1297742 | Myxococcus hansupus T04077 |
| 1189310 | Myxococcus macrosporus DSM 14697 T05096 |
| 1278073 | Myxococcus stipitatus DSM 14675 T02436 |
| 246197 | Myxococcus xanthus DK 1622 T00366 |
| 744533 | Naegleria gruberi strain NEG M T01241 |
| 479431 | Nakamurella multipartita DSM 44233 T00991 |
| 1902245 | Nakamurella sp s14 144 T05729 |
| 1093141 | Nannochloropsis gaditana CCMP526 T02917 |
| 1026970 | Nannospalax galili T03372 |
| 228908 | Nanoarchaeum equitans Kin4 M T00152 |
| 1737403 | Nanohaloarchaea archaeon SG9 T04498 |
| 125878 | Nanorana parkeri T04897 |
| 7425 | Nasonia vitripennis T01081 |
| 457570 | Natranaerobius thermophilus JW NM WN LF T00761 |
| 547559 | Natrialba magadii ATCC 43099 T01176 |
| 797303 | Natrinema pellirubrum DSM 15624 T02425 |
| 406552 | Natrinema sp J7 2 T02184 |
| 797304 | Natronobacterium gregoryi SP2 T02383 |
| 694430 | Natronococcus occultus SP4 T02426 |
| 745377 | Natronolimnobius aegyptiacus T04999 |
| 2044521 | Natronolimnobius sulfurireducens T05593 |
| 2044521 | Natronolimnobius sulfurireducens T05614 |
| 268739 | Natronomonas moolapensis 8 8 11 T02478 |
| 348780 | Natronomonas pharaonis DSM 2160 T00279 |
| 27288 | Naumovozyma castellii CBS 4309 T02233 |
| 1071378 | Naumovozyma dairenensis CBS 421 T02234 |
| 598659 | Nautilia profundicola AmH T00860 |
| 51031 | Necator americanus T04867 |
| 660122 | Nectria haematococca mpVI 77 13 4 T01612 |
| 1853278 | Neisseria chenwenguii T05718 |
| 546263 | Neisseria elongata subsp glycolytica ATCC 29315 T03569 |
| 242231 | Neisseria gonorrhoeae FA 1090 T00237 |
| 521006 | Neisseria gonorrhoeae NCCP11945 T00728 |
| 489653 | Neisseria lactamica 020 06 T01368 |
| 374833 | Neisseria meningitidis 053442 T00633 |
| 604162 | Neisseria meningitidis 8013 T01928 |
| 662598 | Neisseria meningitidis alpha14 T00954 |
| 630588 | Neisseria meningitidis alpha710 T01932 |
| 272831 | Neisseria meningitidis FAM18 T00458 |
| 935599 | Neisseria meningitidis G2136 T01929 |
| 909420 | Neisseria meningitidis H44 76 T01946 |
| 935591 | Neisseria meningitidis M01 240149 T01930 |
| 935588 | Neisseria meningitidis M01 240355 T01947 |
| 935593 | Neisseria meningitidis M04 240196 T01948 |
| 122586 | Neisseria meningitidis MC58 T00027 |
| 935589 | Neisseria meningitidis NZ 05 33 T01949 |
| 487 | Neisseria meningitidis T03438 |
| 942513 | Neisseria meningitidis WUE 2594 T01931 |
| 122587 | Neisseria meningitidis Z2491 T00031 |
| 488 | Neisseria mucosa T05436 |
| 488 | Neisseria sicca T04958 |
| 28091 | Neisseria weaveri T04355 |
| 2067065 | Neisseriaceae bacterium DSM 100970 T05482 |
| 4432 | Nelumbo nucifera T04131 |
| 45351 | Nematostella vectensis T01037 |
| 320497 | Neoasaia chiangmaiensis T04705 |
| 1353976 | Neochlamydia sp S13 T05849 |
| 1287680 | Neofusicoccum parvum UCRNP2 T03126 |
| 556325 | Neomicrococcus aestuarii T04559 |
| 1028801 | Neorhizobium galegae bv officinalis bv officinalis str HAMBI 1141 T03208 |
| 1028800 | Neorhizobium galegae bv orientalis str HAMBI 540 T03244 |
| 1825976 | Neorhizobium sp NCHU2750 T05650 |
| 2060726 | Neorhizobium sp SOG26 T05626 |
| 1286528 | Neorickettsia helminthoeca str Oregon T03076 |
| 434131 | Neorickettsia risticii str Illinois T00951 |
| 222891 | Neorickettsia sennetsu str Miyayama T00329 |
| 367110 | Neurospora crassa OR74A T01034 |
| 510951 | Neurospora tetrasperma FGSC 2508 T03451 |
| 1176587 | Niabella ginsenosidivorans T04418 |
| 929713 | Niabella soli DSM 19437 T03682 |
| 700598 | Niastella koreensis GR20 10 T01679 |
| 49451 | Nicotiana attenuata T05870 |
| 4096 | Nicotiana sylvestris T05026 |
| 4097 | Nicotiana tabacum T04643 |
| 4098 | Nicotiana tomentosiformis T05025 |
| 110193 | Nicrophorus vespilloides T04922 |
| 128390 | Nipponia nippon T05756 |
| 749222 | Nitratifractor salsuginis DSM 16511 T01405 |
| 387092 | Nitratiruptor sp SB155 2 T00565 |
| 323097 | Nitrobacter hamburgensis X14 T00348 |
| 323098 | Nitrobacter winogradskyi Nb 255 T00276 |
| 472759 | Nitrosococcus halophilus Nc 4 T01198 |
| 323261 | Nitrosococcus oceani ATCC 19707 T00281 |
| 105559 | Nitrosococcus watsonii C 113 T01266 |
| 44574 | Nitrosomonas communis T03942 |
| 228410 | Nitrosomonas europaea ATCC 19718 T00130 |
| 335283 | Nitrosomonas eutropha C91 T00393 |
| 153948 | Nitrosomonas sp AL212 T01443 |
| 261292 | Nitrosomonas sp Is79A3 T01569 |
| 44577 | Nitrosomonas ureae T04188 |
| 1580092 | Nitrosopumilus adriaticus T03758 |
| 436308 | Nitrosopumilus maritimus SCM1 T00626 |
| 1582439 | Nitrosopumilus piranensis T03656 |
| 926571 | Nitrososphaera viennensis EN76 T03200 |
| 1288494 | Nitrosospira lacus T04907 |
| 323848 | Nitrosospira multiformis ATCC 25196 T00302 |
| 330214 | Nitrospira defluvii T01281 |
| 1325564 | Nitrospira japonica T05001 |
| 42253 | Nitrospira moscoviensis T04041 |
| 1441467 | Nitrospirillum amazonense CBAmc T04935 |
| 1612173 | Niveispirillum cyanobacteriorum T05262 |
| 1133849 | Nocardia brasiliensis ATCC 700358 T02270 |
| 1127134 | Nocardia cyriacigeorgica GUH 2 T01756 |
| 247156 | Nocardia farcinica IFM 10152 T00207 |
| 37329 | Nocardia farcinica T04524 |
| 2213200 | Nocardia mangyaensis T04582 |
| 1415166 | Nocardia nova SH22a T03032 |
| 37332 | Nocardia seriolae T04599 |
| 455432 | Nocardia terpenica T05168 |
| 1300347 | Nocardioides dokdonensis FR1436 T04431 |
| 196162 | Nocardioides sp JS614 T00443 |
| 1205910 | Nocardiopsis alba ATCC BAA 2165 T02218 |
| 446468 | Nocardiopsis dassonvillei subsp dassonvillei DSM 43111 T01248 |
| 1235441 | Nocardiopsis gilva YIM 90087 T05022 |
| 1914872 | Nodularia spumigena UHCC 0039 T05403 |
| 61853 | Nomascus leucogenys T03265 |
| 592029 | Nonlabens dokdonensis DSW 6 T02441 |
| 323273 | Nonlabens sediminis T04875 |
| 2058134 | Nonlabens sp MB 3u 79 T05253 |
| 1476901 | Nonlabens sp MIC269 T04144 |
| 2496866 | Nonlabens sp MJ115 T05856 |
| 1909395 | Nonomuraea sp ATCC 55076 T04759 |
| 578460 | Nosema ceranae BRL01 T01614 |
| 551115 | Nostoc azollae 0708 T01260 |
| 2038116 | Nostoc flagelliforme CCNUN1 T05266 |
| 63737 | Nostoc punctiforme PCC 73102 T00713 |
| 1869241 | Nostoc sp CENA543 T05285 |
| 1751286 | Nostoc sp NIES 3756 T04170 |
| 317936 | Nostoc sp PCC 7107 T02362 |
| 103690 | Nostoc sp PCC 7120 FACHB 418 T00069 |
| 28072 | Nostoc sp PCC 7524 T02363 |
| 1940762 | Nostocales cyanobacterium HT 58 2 T04905 |
| 105023 | Nothobranchius furzeri T05163 |
| 8208 | Notothenia coriiceps T04920 |
| 1471761 | Novibacillus thermophilus T04694 |
| 279238 | Novosphingobium aromaticivorans DSM 12444 T00322 |
| 1088721 | Novosphingobium pentaromativorans US6 1 T03399 |
| 158500 | Novosphingobium resinovorum T04490 |
| 1609758 | Novosphingobium sp P6W T05550 |
| 702113 | Novosphingobium sp PP1Y T01515 |
| 1016987 | Novosphingobium sp THN1 T05635 |
| 1219035 | Novosphingobium tardaugens NBRC 16725 T05741 |
| 82983 | Obesumbacterium proteus T04310 |
| 1697053 | Oblitimonas alkaliphila T04152 |
| 716816 | Oceanicoccus sagamiensis T04832 |
| 511062 | Oceanimonas sp GK1 T01667 |
| 1903694 | Oceanisphaera avium T04855 |
| 1416627 | Oceanisphaera profunda T04856 |
| 670487 | Oceanithermus profundus DSM 14977 T01369 |
| 221109 | Oceanobacillus iheyensis HTE831 T00093 |
| 2052660 | Oceanobacillus sp 160 T05570 |
| 439375 | Ochrobactrum anthropi ATCC 49188 T00568 |
| 529 | Ochrobactrum anthropi T03251 |
| 419475 | Ochrobactrum pseudogrignonense T04415 |
| 271865 | Ochrobactrum quorumnocens T05040 |
| 391626 | Octadecabacter antarcticus 307 T02538 |
| 391616 | Octadecabacter arcticus 238 T02539 |
| 1458307 | Octadecabacter temperatus T04001 |
| 37653 | Octopus bimaculoides T04127 |
| 9708 | Odobenus rosmarus divergens T05148 |
| 709991 | Odoribacter splanchnicus DSM 20712 T01428 |
| 203123 | Oenococcus oeni PSU 1 T00408 |
| 2203724 | Oenococcus sicerae T05833 |
| 2259623 | Oenococcus sp UCMA 16435 T05781 |
| 158386 | Olea europaea var sylvestris T05244 |
| 141451 | Oleiphilus messinensis T04860 |
| 698738 | Oleispira antarctica RB 8 T04028 |
| 90245 | Oligella urethralis T05343 |
| 1031710 | Oligotropha carboxidovorans OM4 T01967 |
| 504832 | Oligotropha carboxidovorans OM5 T00783 |
| 504832 | Oligotropha carboxidovorans OM5 T01617 |
| 639310 | Olleya aquimaris T05582 |
| 2058135 | Olleya sp Bg11 27 T05214 |
| 712411 | Olsenella sp oral taxon 807 T04016 |
| 633147 | Olsenella uli DSM 7084 T01285 |
| 74940 | Oncorhynchus tshawytscha T05605 |
| 262768 | Onion yellows phytoplasma OY M T00154 |
| 2015173 | Ooceraea biroi T05778 |
| 6198 | Opisthorchis viverrini T04902 |
| 794903 | Opitutaceae bacterium TAV5 T03683 |
| 452637 | Opitutus terrae PB90 1 T00689 |
| 9733 | Orcinus orca T05247 |
| 357244 | Orientia tsutsugamushi str Boryong T00531 |
| 334380 | Orientia tsutsugamushi str Ikeda T00705 |
| 2283195 | Ornithinimicrobium sp AMA3305 T05559 |
| 867902 | Ornithobacterium rhinotracheale DSM 15997 T02145 |
| 1401325 | Ornithobacterium rhinotracheale ORT UMN 88 T03303 |
| 9258 | Ornithorhynchus anatinus T01045 |
| 1851544 | Orrella dioscoreae T05133 |
| 9986 | Oryctolagus cuniculus T03373 |
| 4533 | Oryza brachyantha T02995 |
| 39947 | Oryza sativa Japonica Group T01015 |
| 39947 | Oryza sativa Japonica Group T02163 |
| 8090 | Oryzias latipes T02443 |
| 56110 | Oscillatoria acuminata PCC 6304 T02368 |
| 179408 | Oscillatoria nigro viridis PCC 7112 T02375 |
| 693746 | Oscillibacter valericigenes Sjm18 20 T01636 |
| 436017 | Ostreococcus lucimarinus CCE9901 T01029 |
| 70448 | Ostreococcus tauri T01358 |
| 2109914 | Ottowia oryzae T05363 |
| 1658672 | Ottowia sp oral taxon 894 T04031 |
| 9940 | Ovis aries T03117 |
| 926562 | Owenweeksia hongkongensis DSM 17368 T01677 |
| 847 | Oxalobacter formigenes T04835 |
| 643674 | Paenalcaligenes hominis T04704 |
| 290340 | Paenarthrobacter aurescens TC1 T00447 |
| 1126833 | Paenibacillus beijingensis T03802 |
| 160799 | Paenibacillus borealis T03324 |
| 1616788 | Paenibacillus bovis T04407 |
| 1763538 | Paenibacillus crassostreae T05196 |
| 414771 | Paenibacillus donghaensis T04971 |
| 44251 | Paenibacillus durus T03297 |
| 189425 | Paenibacillus graminis T03325 |
| 1870820 | Paenibacillus ihbetae T05154 |
| 172713 | Paenibacillus kribbensis T05249 |
| 697284 | Paenibacillus larvae subsp larvae DSM 25430 T02963 |
| 1401 | Paenibacillus lautus T05701 |
| 1116391 | Paenibacillus mucilaginosus 3016 T01757 |
| 997761 | Paenibacillus mucilaginosus K02 T02041 |
| 1036673 | Paenibacillus mucilaginosus KNP414 T01550 |
| 162209 | Paenibacillus naphthalenovorans T04167 |
| 189426 | Paenibacillus odorifer T03326 |
| 59893 | Paenibacillus peoriae T04053 |
| 1429244 | Paenibacillus polymyxa CR1 T02956 |
| 349520 | Paenibacillus polymyxa E681 T01319 |
| 1052684 | Paenibacillus polymyxa M1 T01968 |
| 886882 | Paenibacillus polymyxa SC2 T01335 |
| 1413214 | Paenibacillus polymyxa SQR 21 T03178 |
| 1406 | Paenibacillus polymyxa T03812 |
| 1073571 | Paenibacillus riograndensis SBR5 T03849 |
| 1268072 | Paenibacillus sabinae T27 T03064 |
| 1695218 | Paenibacillus sp 32O W T04219 |
| 1536774 | Paenibacillus sp FSL H7 0357 T03358 |
| 1536775 | Paenibacillus sp FSL H7 0737 T03359 |
| 1536769 | Paenibacillus sp FSL P4 0081 T03332 |
| 1536770 | Paenibacillus sp FSL R5 0345 T03333 |
| 1536771 | Paenibacillus sp FSL R5 0912 T03334 |
| 1536772 | Paenibacillus sp FSL R7 0273 T03356 |
| 1536773 | Paenibacillus sp FSL R7 0331 T03357 |
| 867076 | Paenibacillus sp IHB B 3084 T05348 |
| 1566358 | Paenibacillus sp IHBB 10380 T03836 |
| 324057 | Paenibacillus sp JDR 2 T00977 |
| 481743 | Paenibacillus sp Y412MC10 T01100 |
| 169760 | Paenibacillus stellifer T03335 |
| 1178515 | Paenibacillus swuensis T04884 |
| 985665 | Paenibacillus terrae HPL 003 T01668 |
| 715225 | Paenibacillus vortex V453 T05385 |
| 528191 | Paenibacillus xylanexedens T04676 |
| 1462996 | Paenibacillus yonginensis T04810 |
| 1505 | Paeniclostridium sordellii T05252 |
| 2320858 | Paenisporosarcina sp K2R23 3 T05647 |
| 1343739 | Palaeococcus pacificus DY20341 T03210 |
| 694427 | Paludibacter propionicigenes WB4 T01362 |
| 1387353 | Paludisphaera borealis T04622 |
| 9597 | Pan paniscus T02283 |
| 9598 | Pan troglodytes T01005 |
| 93218 | Pandoraea apista T03588 |
| 656179 | Pandoraea faecigallinarum T03972 |
| 93219 | Pandoraea norimbergensis T04226 |
| 573737 | Pandoraea oxalativorans T03840 |
| 1416914 | Pandoraea pnomenusa 3kgm T02936 |
| 93220 | Pandoraea pnomenusa T02950 |
| 93220 | Pandoraea pnomenusa T03067 |
| 93220 | Pandoraea pnomenusa T03411 |
| 93221 | Pandoraea pulmonicola T03553 |
| 93222 | Pandoraea sputorum T03554 |
| 445709 | Pandoraea thiooxydans T03947 |
| 656178 | Pandoraea vervacti T03688 |
| 310915 | Pangasianodon hypophthalmus T05759 |
| 121719 | Pannonibacter phragmitetus T04214 |
| 74533 | Panthera tigris altaica T02988 |
| 59538 | Pantholops hodgsonii T02909 |
| 549 | Pantoea agglomerans T04739 |
| 1891675 | Pantoea alhagi T05014 |
| 932677 | Pantoea ananatis AJ13355 T01969 |
| 706191 | Pantoea ananatis LMG 20103 T01195 |
| 1123863 | Pantoea ananatis LMG 5342 T01728 |
| 1095774 | Pantoea ananatis PA13 T01970 |
| 1076550 | Pantoea rwandensis T03331 |
| 592316 | Pantoea sp At 9b T01386 |
| 1484158 | Pantoea sp PSNIH1 T03464 |
| 1484157 | Pantoea sp PSNIH2 T03514 |
| 660596 | Pantoea stewartii subsp stewartii DC283 T04970 |
| 712898 | Pantoea vagans C9 1 T01326 |
| 470934 | Pantoea vagans T04302 |
| 3469 | Papaver somniferum T05758 |
| 76193 | Papilio machaon T05138 |
| 435591 | Parabacteroides distasonis ATCC 8503 T00549 |
| 2025876 | Parabacteroides sp CT06 T05011 |
| 2026199 | Paraburkholderia aromaticivorans T05169 |
| 134536 | Paraburkholderia caledonica T05740 |
| 1323664 | Paraburkholderia caribensis MBA4 T04141 |
| 134537 | Paraburkholderia fungorum T03799 |
| 60548 | Paraburkholderia graminis T05548 |
| 169430 | Paraburkholderia hospita T05305 |
| 1229205 | Paraburkholderia phenoliruptrix BR3459a T02300 |
| 391038 | Paraburkholderia phymatum STM815 T00704 |
| 398527 | Paraburkholderia phytofirmans PsJN T00765 |
| 882378 | Paraburkholderia rhizoxinica HKI 454 T01355 |
| 2654982 | Paraburkholderia sp CCGE1002 T01217 |
| 1926494 | Paraburkholderia sp SOS3 T04966 |
| 754502 | Paraburkholderia sprentiae WSM5005 T04610 |
| 311230 | Paraburkholderia terrae T05318 |
| 169427 | Paraburkholderia terricola T05812 |
| 266265 | Paraburkholderia xenovorans LB400 T00340 |
| 266265 | Paraburkholderia xenovorans LB400 T03290 |
| 765952 | Parachlamydia acanthamoebae UV 7 T01552 |
| 502780 | Paracoccidioides brasiliensis Pb18 T03549 |
| 502779 | Paracoccidioides lutzii Pb01 T02230 |
| 1367847 | Paracoccus aminophilus JCM 7686 T02792 |
| 34004 | Paracoccus aminovorans T05468 |
| 1945662 | Paracoccus contaminans T04876 |
| 318586 | Paracoccus denitrificans PD1222 T00440 |
| 2065379 | Paracoccus jeotgali T05276 |
| 1499308 | Paracoccus mutanolyticus T05551 |
| 1529068 | Paracoccus sp BM15 T05237 |
| 2259340 | Paracoccus sp SC2 6 T05723 |
| 147645 | Paracoccus yeei T04781 |
| 1077935 | Paracoccus zhejiangensis T05233 |
| 2315862 | Paraflavitalea soli T05640 |
| 634956 | Parageobacillus thermoglucosidasius C56 YS93 T01531 |
| 1426 | Parageobacillus thermoglucosidasius T04476 |
| 1129794 | Paraglaciecola psychrophila 170 T02535 |
| 5888 | Paramecium tetraurelia strain d4 2 T01055 |
| 1676925 | Paramormyrops kingsleyae T05788 |
| 1755811 | Paraphotobacterium marinum T05093 |
| 1150469 | Pararhodospirillum photometricum DSM 122 T01851 |
| 864564 | Parascardovia denticolens DSM 10105 JCM 12538 T03978 |
| 2483033 | Parasedimentitalea marina T05780 |
| 2584094 | Parasphingorhabdus flavimaris T04949 |
| 321614 | Parastagonospora nodorum SN15 T01161 |
| 2003188 | Parolsenella catena T05797 |
| 9157 | Parus major T04864 |
| 402881 | Parvibaculum lavamentivorans DS 1 T00580 |
| 33033 | Parvimonas micra T03515 |
| 314260 | Parvularcula bermudensis HTCC2503 T01298 |
| 754 | Pasteurella dagmatis T05467 |
| 1075089 | Pasteurella multocida 36950 T01729 |
| 584721 | Pasteurella multocida subsp multocida str 3480 T02057 |
| 1132496 | Pasteurella multocida subsp multocida str HN06 T01776 |
| 272843 | Pasteurella multocida subsp multocida str Pm70 T00046 |
| 747 | Pasteurella multocida T03322 |
| 1768242 | Paucibacter sp KCTC 42545 T04227 |
| 29443 | Paucimonas lemoignei T05492 |
| 218491 | Pectobacterium atrosepticum SCRI1043 T00186 |
| 29471 | Pectobacterium atrosepticum T03199 |
| 29471 | Pectobacterium atrosepticum T03293 |
| 561230 | Pectobacterium carotovorum subsp carotovorum PC1 T00934 |
| 1218933 | Pectobacterium carotovorum subsp carotovorum PCC21 T02320 |
| 78398 | Pectobacterium odoriferum T03424 |
| 1905730 | Pectobacterium parmentieri T02514 |
| 1905730 | Pectobacterium parmentieri T04513 |
| 561231 | Pectobacterium parmentieri WPP163 T01106 |
| 2042057 | Pectobacterium polaris T05195 |
| 1175631 | Pectobacterium wasabiae CFBP 3304 T04535 |
| 121224 | Pediculus humanus corporis T01223 |
| 1254 | Pediococcus acidilactici T04512 |
| 701521 | Pediococcus claussenii ATCC BAA 344 T01713 |
| 51663 | Pediococcus damnosus T04321 |
| 114090 | Pediococcus inopinatus T05337 |
| 278197 | Pediococcus pentosaceus ATCC 25745 T00409 |
| 1408206 | Pediococcus pentosaceus SL4 T02906 |
| 188932 | Pedobacter cryoconitis T04341 |
| 363852 | Pedobacter ginsengisoli T05145 |
| 485917 | Pedobacter heparinus DSM 2366 T00978 |
| 1727164 | Pedobacter sp PACM 27299 T04140 |
| 430522 | Pedobacter steynii T04469 |
| 1250539 | Pelagibaca abyssi T04681 |
| 1082931 | Pelagibacterium halotolerans B2 T01645 |
| 29542 | Pelobacter acetylenicus T04585 |
| 338963 | Pelobacter carbinolicus DSM 2380 T00285 |
| 338966 | Pelobacter propionicus DSM 2379 T00436 |
| 1842532 | Pelobacter sp SFB93 T04594 |
| 319225 | Pelodictyon luteolum DSM 273 T00291 |
| 324925 | Pelodictyon phaeoclathratiforme BU 1 T00731 |
| 13735 | Pelodiscus sinensis T02980 |
| 913107 | Pelolinea submarina T05783 |
| 1192197 | Pelosinus fermentans JBW45 T03813 |
| 484770 | Pelosinus sp UFO1 T03211 |
| 370438 | Pelotomaculum thermopropionicum SI T00519 |
| 1170230 | Penicillium digitatum Pd1 T04849 |
| 1108849 | Penicillium rubens Wisconsin 54 1255 T01091 |
| 1286171 | Peptoclostridium acidaminophilum DSM 3953 T03061 |
| 54005 | Peptoniphilus harei T05838 |
| 1912856 | Peptoniphilus sp ING2 D1G T03807 |
| 2081703 | Peptostreptococcaceae bacterium oral taxon 929 T05383 |
| 123214 | Persephonella marina EX H1 T00870 |
| 1229662 | Pestalotiopsis fici W106 1 T04924 |
| 1642646 | Petrimonas mucosa T04553 |
| 1978337 | Petrimonas sp IBARAKI T05132 |
| 2173034 | Petrocella atlantisensis T05737 |
| 403833 | Petrotoga mobilis SJ95 T00621 |
| 1286976 | Phaeoacremonium minimum UCRPA7 T03102 |
| 1423144 | Phaeobacter gallaeciensis DSM 26640 T02968 |
| 383629 | Phaeobacter inhibens 2 10 T02193 |
| 391619 | Phaeobacter inhibens DSM 17395 T02192 |
| 1580596 | Phaeobacter piscinae T05105 |
| 1844006 | Phaeobacter porticola T04595 |
| 681157 | Phaeobacter sp LSS9 T05617 |
| 556484 | Phaeodactylum tricornutum CCAP 1055 1 T01075 |
| 78828 | Phalaenopsis equestris T05601 |
| 650164 | Phanerochaete carnosa HHB 10118 sp T03128 |
| 33025 | Phascolarctobacterium faecium T05784 |
| 38626 | Phascolarctos cinereus T05867 |
| 3885 | Phaseolus vulgaris T03093 |
| 2201350 | Phenylobacterium sp HYN0004 T05485 |
| 450851 | Phenylobacterium zucineum HLK1 T00750 |
| 149040 | Phialocephala scopiformis T04865 |
| 42345 | Phoenix dactylifera T03274 |
| 85581 | Photobacterium damselae subsp damselae T04927 |
| 658445 | Photobacterium gaetbulicola Gung47 T03698 |
| 298386 | Photobacterium profundum SS9 T00173 |
| 291112 | Photorhabdus asymbiotica T00941 |
| 141679 | Photorhabdus laumondii subsp laumondii T05478 |
| 243265 | Photorhabdus laumondii subsp laumondii TTO1 T00149 |
| 230089 | Photorhabdus thracensis T03944 |
| 1868589 | Phreatobacter cathodiphilus T05356 |
| 1142394 | Phycisphaera mikurensis NBRC 102666 T01800 |
| 1867719 | Phyllobacterium zundukense T05167 |
| 3218 | Physcomitrium patens T01041 |
| 1972431 | Phytobacter ursingii T03950 |
| 403677 | Phytophthora infestans T30 4 T01333 |
| 67593 | Phytophthora sojae T03459 |
| 1122961 | Picrophilus torridus DSM 9790 T00177 |
| 64459 | Pieris rapae T05162 |
| 2488560 | Pigmentiphaga sp H8 T05719 |
| 2045 | Pimelobacter simplex T03528 |
| 1632865 | Pirellula sp SH Sr6A T04360 |
| 530564 | Pirellula staleyi DSM 6068 T01147 |
| 1227812 | Piscirickettsia salmonis LF 89 ATCC VR 1361 T03982 |
| 1632864 | Planctomyces sp SH PL14 T04354 |
| 1636152 | Planctomyces sp SH PL62 T04401 |
| 521674 | Planctopirus limnophila DSM 3776 T01225 |
| 666509 | Planktomarina temperata RCA23 T03229 |
| 282423 | Planktothrix agardhii NIES 204 T05658 |
| 1185653 | Planococcus antarcticus DSM 14505 T04428 |
| 414778 | Planococcus donghaensis T04451 |
| 1598147 | Planococcus faecalis T04955 |
| 1215089 | Planococcus halocryophilus T04473 |
| 1374 | Planococcus kocurii T04200 |
| 2213202 | Planococcus maritimus T04763 |
| 1038856 | Planococcus plakortidis T04812 |
| 200991 | Planococcus rifietoensis T04201 |
| 2058136 | Planococcus sp MB 3u 03 T05387 |
| 1526927 | Planococcus sp PAMC 21323 T03510 |
| 1302659 | Planococcus versutus T04421 |
| 2071627 | Plantactinospora sp BB1 T05391 |
| 2108470 | Plantactinospora sp BC1 T05377 |
| 2024580 | Plantactinospora sp KBS50 T05048 |
| 2480625 | Plantibacter sp PA 3 X8 T05731 |
| 5823 | Plasmodium berghei ANKA T01111 |
| 31271 | Plasmodium chabaudi chabaudi T01108 |
| 1120755 | Plasmodium cynomolgi strain B T02490 |
| 36329 | Plasmodium falciparum 3D7 T00095 |
| 57267 | Plasmodium falciparum Dd2 T01051 |
| 137071 | Plasmodium falciparum HB3 T01052 |
| 5851 | Plasmodium knowlesi strain H T01094 |
| 126793 | Plasmodium vivax Sal 1 T01073 |
| 5861 | Plasmodium yoelii T01035 |
| 891974 | Plautia stali symbiont T03051 |
| 1885025 | Pleomorphomonas sp SM30 T05766 |
| 703 | Plesiomonas shigelloides T04378 |
| 118163 | Pleurocapsa sp PCC 7327 T02367 |
| 61647 | Pluralibacter gergoviae T03300 |
| 51655 | Plutella xylostella T03915 |
| 46731 | Pocillopora damicornis T05773 |
| 515849 | Podospora anserina S mat T01048 |
| 8081 | Poecilia reticulata T05149 |
| 103695 | Pogona vitticeps T05000 |
| 144034 | Pogonomyrmex barbatus T04903 |
| 996801 | Polaribacter reichenbachii T04692 |
| 2058137 | Polaribacter sp ALD11 T05264 |
| 1529069 | Polaribacter sp BM10 T04746 |
| 313598 | Polaribacter sp MED152 T02533 |
| 1774273 | Polaribacter vadi T04504 |
| 365044 | Polaromonas naphthalenivorans CJ2 T00459 |
| 296591 | Polaromonas sp JS666 T00343 |
| 2268087 | Polaromonas sp SP1 T05689 |
| 91411 | Polistes canadensis T05611 |
| 413882 | Polyangium brachysporum T04216 |
| 991905 | Polymorphum gilvum SL003B 26A1 T01458 |
| 312153 | Polynucleobacter asymbioticus QLW P1DMWA 1 T00502 |
| 1835254 | Polynucleobacter duraquae T04207 |
| 452638 | Polynucleobacter necessarius STIR1 T00683 |
| 2527775 | Polynucleobacter paneuropaeus T05507 |
| 400727 | Pomacea canaliculata T05881 |
| 9601 | Pongo abelii T01416 |
| 323450 | Pontibacter actiniarum T05632 |
| 400092 | Pontibacter korlensis T03841 |
| 1159327 | Pontimonas salivibrio T05334 |
| 3694 | Populus trichocarpa T01077 |
| 1112 | Porphyrobacter neustonensis T04416 |
| 2023229 | Porphyrobacter sp HT 58 2 T05364 |
| 1896196 | Porphyrobacter sp LM 6 T04468 |
| 879243 | Porphyromonas asaccharolytica DSM 20707 T01485 |
| 393921 | Porphyromonas crevioricanis T05510 |
| 431947 | Porphyromonas gingivalis ATCC 33277 T00714 |
| 1030843 | Porphyromonas gingivalis TDC60 T01516 |
| 242619 | Porphyromonas gingivalis W83 T00145 |
| 561896 | Postia placenta Mad 698 R T01162 |
| 82985 | Pragia fontium T03952 |
| 530584 | Prauserella marina T04988 |
| 908937 | Prevotella dentalis DSM 3688 T02403 |
| 767031 | Prevotella denticola F0289 T01459 |
| 76123 | Prevotella enoeca T04173 |
| 1236517 | Prevotella fusca JCM 17724 T04032 |
| 246198 | Prevotella intermedia 17 T02058 |
| 1177574 | Prevotella jejuni T05287 |
| 553174 | Prevotella melaninogenica ATCC 25845 T01286 |
| 264731 | Prevotella ruminicola 23 T01212 |
| 575614 | Prevotella sp oral taxon 299 str F0039 T02809 |
| 146891 | Prochlorococcus marinus str AS9601 T00464 |
| 93059 | Prochlorococcus marinus str MIT 9211 T00616 |
| 93060 | Prochlorococcus marinus str MIT 9215 T00598 |
| 167546 | Prochlorococcus marinus str MIT 9301 T00488 |
| 59922 | Prochlorococcus marinus str MIT 9303 T00466 |
| 74546 | Prochlorococcus marinus str MIT 9312 T00297 |
| 74547 | Prochlorococcus marinus str MIT 9313 T00143 |
| 167542 | Prochlorococcus marinus str MIT 9515 T00465 |
| 167555 | Prochlorococcus marinus str NATL1A T00467 |
| 59920 | Prochlorococcus marinus str NATL2A T00267 |
| 167539 | Prochlorococcus marinus subsp marinus str CCMP1375 T00137 |
| 59919 | Prochlorococcus marinus subsp pastoris str CCMP1986 T00142 |
| 1501268 | Prochlorococcus sp MIT 0604 T03304 |
| 1501269 | Prochlorococcus sp MIT 0801 T03316 |
| 556499 | Propionibacterium acidifaciens T05713 |
| 66712 | Propionibacterium freudenreichii subsp freudenreichii T03860 |
| 754252 | Propionibacterium freudenreichii subsp shermanii CIRM BIA1 T01252 |
| 2559073 | Propionibacterium sp oral taxon 193 T04515 |
| 290512 | Prosthecochloris aestuarii DSM 271 T00732 |
| 1868325 | Prosthecochloris sp CIB 2401 T04424 |
| 281093 | Prosthecochloris sp GSB1 T04996 |
| 1974213 | Prosthecochloris sp HL 130 GSB T04847 |
| 2419774 | Protaetiibacter intestinalis T05661 |
| 1642647 | Proteiniphilum saccharofermentans T04672 |
| 183417 | Proteus hauseri T05854 |
| 1266738 | Proteus mirabilis BB2000 T02789 |
| 529507 | Proteus mirabilis HI4320 T00753 |
| 584 | Proteus mirabilis T04093 |
| 585 | Proteus vulgaris T05486 |
| 103944 | Protobothrops mucrosquamatus T05763 |
| 126385 | Providencia alcalifaciens T05107 |
| 333962 | Providencia heimbachae T05509 |
| 587 | Providencia rettgeri T04571 |
| 2027290 | Providencia sp WCHPHu000369 T05654 |
| 1157951 | Providencia stuartii MRSN 2154 T02042 |
| 588 | Providencia stuartii T03282 |
| 588 | Providencia stuartii T04734 |
| 42229 | Prunus avium T05161 |
| 102107 | Prunus mume T03196 |
| 3760 | Prunus persica T03092 |
| 685565 | Pseudanabaena sp ABRG5 3 T05820 |
| 82654 | Pseudanabaena sp PCC 7367 T02369 |
| 452863 | Pseudarthrobacter chlorophenolicus A6 T00834 |
| 930171 | Pseudarthrobacter phenanthrenivorans Sphe3 T01435 |
| 121292 | Pseudarthrobacter sulfonivorans T04210 |
| 247523 | Pseudoalteromonas aliena T04815 |
| 1117313 | Pseudoalteromonas arctica A 37 1 2 T05179 |
| 3042615 | Pseudoalteromonas atlantica T6c T00372 |
| 621376 | Pseudoalteromonas donghaensis T05890 |
| 1314869 | Pseudoalteromonas espejiana DSM 9414 T04989 |
| 326442 | Pseudoalteromonas haloplanktis TAC125 T00282 |
| 152297 | Pseudoalteromonas issachenkonii T04164 |
| 43657 | Pseudoalteromonas luteoviolacea T04577 |
| 28109 | Pseudoalteromonas nigrifaciens T05271 |
| 161398 | Pseudoalteromonas phenolica T04165 |
| 1348114 | Pseudoalteromonas piratica T03513 |
| 43662 | Pseudoalteromonas piscicida T04942 |
| 43658 | Pseudoalteromonas rubra T04179 |
| 283699 | Pseudoalteromonas sp Bsw20308 T04175 |
| 1514074 | Pseudoalteromonas sp NC201 T05464 |
| 234831 | Pseudoalteromonas sp SM9913 T01374 |
| 1117319 | Pseudoalteromonas spongiae UST010723 006 T05098 |
| 43659 | Pseudoalteromonas tetraodonis T05282 |
| 1315283 | Pseudoalteromonas translucida KMM 520 T04220 |
| 314281 | Pseudoalteromonas tunicata T05187 |
| 383855 | Pseudocercospora fijiensis CIRAD86 T03109 |
| 84032 | Pseudoclostridium thermosuccinogenes T05300 |
| 643562 | Pseudodesulfovibrio aespoeensis Aspo 2 T01382 |
| 1716143 | Pseudodesulfovibrio indicus T04404 |
| 1322246 | Pseudodesulfovibrio piezophilus C1TLV30 T02481 |
| 57320 | Pseudodesulfovibrio profundus T05258 |
| 748280 | Pseudogulbenkiania sp NH8B T01619 |
| 1249552 | Pseudohongiella spirulinae T04158 |
| 1280938 | Pseudomonas aeruginosa B136 33 T02627 |
| 1352355 | Pseudomonas aeruginosa c7447m T03098 |
| 1093787 | Pseudomonas aeruginosa DK2 T02161 |
| 1408272 | Pseudomonas aeruginosa LES431 T02970 |
| 557722 | Pseudomonas aeruginosa LESB58 T00818 |
| 941193 | Pseudomonas aeruginosa M18 T01973 |
| 1415629 | Pseudomonas aeruginosa MTB 1 T02951 |
| 1089456 | Pseudomonas aeruginosa NCGM2 S1 T01974 |
| 1279007 | Pseudomonas aeruginosa PA1 T02928 |
| 1279008 | Pseudomonas aeruginosa PA1R T02929 |
| 1407059 | Pseudomonas aeruginosa PA38182 T03031 |
| 381754 | Pseudomonas aeruginosa PA7 T00569 |
| 208964 | Pseudomonas aeruginosa PAO1 T00035 |
| 1367494 | Pseudomonas aeruginosa PAO1 VE13 T03097 |
| 1367493 | Pseudomonas aeruginosa PAO1 VE2 T03170 |
| 1352354 | Pseudomonas aeruginosa PAO581 T03171 |
| 1340851 | Pseudomonas aeruginosa RP73 T02711 |
| 1427342 | Pseudomonas aeruginosa SCV20265 T02971 |
| 287 | Pseudomonas aeruginosa T03789 |
| 208963 | Pseudomonas aeruginosa UCBPP PA14 T00401 |
| 1448140 | Pseudomonas aeruginosa YL84 T03035 |
| 43263 | Pseudomonas alcaligenes T04318 |
| 237609 | Pseudomonas alkylphenolica T03238 |
| 129138 | Pseudomonas amygdali pv morsprunorum T05354 |
| 219572 | Pseudomonas antarctica T04830 |
| 46257 | Pseudomonas avellanae T05369 |
| 47878 | Pseudomonas azotoformans T04879 |
| 1123016 | Pseudomonas balearica DSM 6083 T03573 |
| 994484 | Pseudomonas brassicacearum subsp brassicacearum NFM421 T01472 |
| 930166 | Pseudomonas brassicacearum T03174 |
| 86192 | Pseudomonas chlororaphis subsp aurantiaca T03338 |
| 587753 | Pseudomonas chlororaphis T03201 |
| 587753 | Pseudomonas chlororaphis T03768 |
| 1441629 | Pseudomonas cichorii JBC1 T03002 |
| 53408 | Pseudomonas citronellolis T04637 |
| 47879 | Pseudomonas corrugata T04842 |
| 157783 | Pseudomonas cremoricolorata T03354 |
| 384676 | Pseudomonas entomophila L48 T00362 |
| 1037911 | Pseudomonas fluorescens A506 T02114 |
| 1114970 | Pseudomonas fluorescens F113 T01734 |
| 205922 | Pseudomonas fluorescens Pf0 1 T00283 |
| 321846 | Pseudomonas fluorescens PICF7 T03767 |
| 216595 | Pseudomonas fluorescens SBW25 T00899 |
| 294 | Pseudomonas fluorescens T03319 |
| 321846 | Pseudomonas fluorescens T03766 |
| 244566 | Pseudomonas fluorescens T04730 |
| 296 | Pseudomonas fragi T04285 |
| 104087 | Pseudomonas frederiksbergensis T04786 |
| 743720 | Pseudomonas fulva 12 X T01501 |
| 1301098 | Pseudomonas knackmussii B13 T03081 |
| 198620 | Pseudomonas koreensis T04647 |
| 1147786 | Pseudomonas mandelii JR 1 T03626 |
| 1001585 | Pseudomonas mendocina NK 01 T01473 |
| 399739 | Pseudomonas mendocina ymp T00510 |
| 1435044 | Pseudomonas monteilii SB3078 T02972 |
| 1435058 | Pseudomonas monteilii SB3101 T02973 |
| 76758 | Pseudomonas orientalis T05323 |
| 47885 | Pseudomonas oryzihabitans T04267 |
| 157782 | Pseudomonas parafulva T03520 |
| 70775 | Pseudomonas plecoglossicida T03646 |
| 1282356 | Pseudomonas poae RE 1 1 14 T02456 |
| 1420599 | Pseudomonas protegens Cab57 T03613 |
| 1124983 | Pseudomonas protegens CHA0 T02671 |
| 220664 | Pseudomonas protegens Pf 5 T00249 |
| 1182590 | Pseudomonas pseudoalcaligenes CECT 5344 T03772 |
| 47885 | Pseudomonas psychrotolerans T05074 |
| 931281 | Pseudomonas putida BIRD 1 T01975 |
| 1196325 | Pseudomonas putida DOT T1E T02181 |
| 351746 | Pseudomonas putida F1 T00537 |
| 76869 | Pseudomonas putida GB 1 T00650 |
| 1331671 | Pseudomonas putida H8234 T02706 |
| 1215088 | Pseudomonas putida HB3267 T02389 |
| 160488 | Pseudomonas putida KT2440 T00114 |
| 1211579 | Pseudomonas putida NBRC 14164 T02707 |
| 231023 | Pseudomonas putida ND6 T02170 |
| 1042876 | Pseudomonas putida S16 T01570 |
| 303 | Pseudomonas putida T03439 |
| 390235 | Pseudomonas putida W619 T00675 |
| 1245471 | Pseudomonas resinovorans NBRC 106553 T02708 |
| 216142 | Pseudomonas rhizosphaerae T03339 |
| 264730 | Pseudomonas savastanoi pv phaseolicola 1448A T00266 |
| 1853130 | Pseudomonas silesiensis T05412 |
| 1306993 | Pseudomonas soli T03276 |
| 1294143 | Pseudomonas sp ATCC 13867 T02524 |
| 1649877 | Pseudomonas sp CCOS 191 T03967 |
| 1611770 | Pseudomonas sp MRSN12121 T03702 |
| 1500686 | Pseudomonas sp Os17 T04177 |
| 1028989 | Pseudomonas sp StFLB209 T03623 |
| 1856685 | Pseudomonas sp TCU HL1 T05373 |
| 1415630 | Pseudomonas sp TKP T02969 |
| 1207075 | Pseudomonas sp UW4 T02357 |
| 69328 | Pseudomonas sp VLB120 T02897 |
| 379731 | Pseudomonas stutzeri A1501 T00511 |
| 96563 | Pseudomonas stutzeri ATCC 17588 LMG 11199 T01571 |
| 1196835 | Pseudomonas stutzeri CCUG 29243 T02168 |
| 1123519 | Pseudomonas stutzeri DSM 10701 T02182 |
| 996285 | Pseudomonas stutzeri DSM 4166 T01976 |
| 644801 | Pseudomonas stutzeri RCH2 T02391 |
| 3022791 | Pseudomonas stutzeri T03366 |
| 316 | Pseudomonas stutzeri T03390 |
| 47883 | Pseudomonas synxantha T03769 |
| 1357279 | Pseudomonas syringae CC1557 T03014 |
| 205918 | Pseudomonas syringae pv syringae B728a T00246 |
| 223283 | Pseudomonas syringae pv tomato str DC3000 T00118 |
| 200450 | Pseudomonas trivialis T04006 |
| 76761 | Pseudomonas veronii T04853 |
| 1788301 | Pseudomonas versuta T04081 |
| 515393 | Pseudomonas yamanorum T05821 |
| 219809 | Pseudomyrmex gracilis T05003 |
| 675635 | Pseudonocardia dioxanivorans CB1190 T01479 |
| 445576 | Pseudonocardia sp AL041005 10 T04051 |
| 1688404 | Pseudonocardia sp EC080610 09 T04146 |
| 1096856 | Pseudonocardia sp EC080619 01 T04234 |
| 1096868 | Pseudonocardia sp EC080625 04 T04054 |
| 1641402 | Pseudonocardia sp HH130629 09 T04082 |
| 1690815 | Pseudonocardia sp HH130630 07 T04486 |
| 762903 | Pseudopedobacter saltans DSM 12145 T01430 |
| 181119 | Pseudopodoces humilis T02921 |
| 767029 | Pseudopropionibacterium propionicum F0230a T02180 |
| 1235591 | Pseudorhodoplanes sinuspersici T04834 |
| 1123384 | Pseudothermotoga hypogea DSM 11164 NBRC 106472 T04277 |
| 416591 | Pseudothermotoga lettingae TMO T00618 |
| 688269 | Pseudothermotoga thermarum DSM 5069 T01554 |
| 911045 | Pseudovibrio sp FO BEG1 T01669 |
| 1045855 | Pseudoxanthomonas spadix BD a59 T01643 |
| 743721 | Pseudoxanthomonas suwonensis 11 1 T01403 |
| 314722 | Pseudoxanthomonas suwonensis T04241 |
| 261164 | Psychrobacter alimentarius T04320 |
| 259536 | Psychrobacter arcticus 273 4 T00256 |
| 335284 | Psychrobacter cryohalolentis K5 T00350 |
| 1720344 | Psychrobacter sp AntiMn 1 T04964 |
| 1028416 | Psychrobacter sp DAB AL43B T04748 |
| 86185 | Psychrobacter sp FME2 T05473 |
| 571800 | Psychrobacter sp G T02715 |
| 1699624 | Psychrobacter sp P11G5 T04411 |
| 1699622 | Psychrobacter sp P2G3 T04386 |
| 349106 | Psychrobacter sp PRwf 1 T00541 |
| 2203895 | Psychrobacter sp YP14 T05488 |
| 45610 | Psychrobacter urativorans T04087 |
| 313595 | Psychroflexus torquis ATCC 700755 T02275 |
| 1618207 | Psychromicrobium lacuslunae T03775 |
| 357804 | Psychromonas ingrahamii 37 T00450 |
| 314282 | Psychromonas sp CNPT3 T02515 |
| 9402 | Pteropus alecto T02993 |
| 418459 | Puccinia graminis f sp tritici CRL 75 36 700 3 T02226 |
| 2116657 | Pulveribacter suum T05362 |
| 741275 | Punctularia strigosozonata HHB 11173 SS5 T03131 |
| 33203 | Purpureocillium lilacinum T05029 |
| 1007105 | Pusillimonas sp T7 7 T01486 |
| 2028345 | Pusillimonas sp ye3 T05795 |
| 861557 | Pyrenophora teres f teres 0 1 T01613 |
| 242507 | Pyricularia oryzae 70 15 T01027 |
| 178306 | Pyrobaculum aerophilum str IM2 T00073 |
| 340102 | Pyrobaculum arsenaticum DSM 13514 T00503 |
| 410359 | Pyrobaculum calidifontis JCM 11548 T00483 |
| 1104324 | Pyrobaculum ferrireducens T01670 |
| 384616 | Pyrobaculum islandicum DSM 4184 T00444 |
| 444157 | Pyrobaculum neutrophilum V24Sta T00682 |
| 698757 | Pyrobaculum oguniense TE7 T01735 |
| 1227555 | Pyrobaculum sp WP30 T04013 |
| 272844 | Pyrococcus abyssi GE5 T00024 |
| 1185654 | Pyrococcus furiosus COM1 T02183 |
| 186497 | Pyrococcus furiosus DSM 3638 T00075 |
| 70601 | Pyrococcus horikoshii OT3 T00014 |
| 1609559 | Pyrococcus kukulkanii T04365 |
| 342949 | Pyrococcus sp NA2 T01487 |
| 1183377 | Pyrococcus sp ST04 T02124 |
| 529709 | Pyrococcus yayanosii CH1 T01543 |
| 1273541 | Pyrodictium delaneyi T04114 |
| 694429 | Pyrolobus fumarii 1A T01620 |
| 225117 | Pyrus x bretschneideri T03446 |
| 176946 | Python bivittatus T03091 |
| 58331 | Quercus suber T05868 |
| 745277 | Rahnella aquatilis CIP 78 65 ATCC 33071 T01770 |
| 1151116 | Rahnella aquatilis HX2 T01850 |
| 1805933 | Rahnella sp ERMR1 05 T05331 |
| 2703885 | Rahnella sp Y9602 T01422 |
| 190721 | Ralstonia insidiosa T04471 |
| 105219 | Ralstonia mannitolilytica T03778 |
| 428406 | Ralstonia pickettii 12D T00925 |
| 402626 | Ralstonia pickettii 12J T00762 |
| 1366050 | Ralstonia pickettii DTP0602 T03172 |
| 1310165 | Ralstonia pseudosolanacearum T05645 |
| 859656 | Ralstonia solanacearum CFBP2957 T01272 |
| 859655 | Ralstonia solanacearum CMR15 T02725 |
| 1262456 | Ralstonia solanacearum FQY 4 T02751 |
| 267608 | Ralstonia solanacearum GMI1000 T00071 |
| 1031711 | Ralstonia solanacearum Po82 T02005 |
| 859657 | Ralstonia solanacearum PSI07 T01273 |
| 365046 | Ramlibacter tataouinensis TTB310 T01544 |
| 1286170 | Raoultella ornithinolytica B6 T02625 |
| 54291 | Raoultella ornithinolytica T03629 |
| 575 | Raoultella planticola T05298 |
| 2259647 | Raoultella sp X13 T05540 |
| 59737 | Rathayibacter iranicus T05878 |
| 33887 | Rathayibacter rathayi T05845 |
| 145458 | Rathayibacter toxicus T03670 |
| 145458 | Rathayibacter toxicus T04269 |
| 33888 | Rathayibacter tritici T04388 |
| 10116 | Rattus norvegicus T01003 |
| 1336806 | Reinekea forsetii T05208 |
| 288705 | Renibacterium salmoninarum ATCC 33209 T00641 |
| 89399 | Rhinolophus sinicus T04863 |
| 61621 | Rhinopithecus bieti T04641 |
| 61622 | Rhinopithecus roxellana T03989 |
| 946333 | Rhizobacter gummiphilus T04831 |
| 1862950 | Rhizobiales bacterium NRL2 T04419 |
| 1967781 | Rhizobium esperanzae T05681 |
| 1328306 | Rhizobium etli bv mimosae str Mim1 T02778 |
| 1432049 | Rhizobium etli bv phaseoli str IE4803 T03667 |
| 347834 | Rhizobium etli CFN 42 T00317 |
| 491916 | Rhizobium etli CIAT 652 T00720 |
| 348824 | Rhizobium favelukesii T03046 |
| 1041138 | Rhizobium gallicum bv gallicum R602 T03539 |
| 1312183 | Rhizobium jaguaris T05891 |
| 1033991 | Rhizobium leguminosarum bv trifolii CB782 T03015 |
| 395491 | Rhizobium leguminosarum bv trifolii WSM1325 T00926 |
| 754523 | Rhizobium leguminosarum bv trifolii WSM1689 T03009 |
| 395492 | Rhizobium leguminosarum bv trifolii WSM2304 T00779 |
| 216596 | Rhizobium leguminosarum bv viciae 3841 T00402 |
| 396 | Rhizobium phaseoli T04590 |
| 1432050 | Rhizobium sp IE4771 T03310 |
| 648995 | Rhizobium sp IRBG74 T02867 |
| 2020311 | Rhizobium sp Kim5 T05501 |
| 1703962 | Rhizobium sp N1341 T04420 |
| 1703966 | Rhizobium sp N731 T04809 |
| 1125847 | Rhizobium sp NT 26 T04765 |
| 1869170 | Rhizobium sp S41 T05451 |
| 698761 | Rhizobium tropici CIAT 899 T02432 |
| 1850238 | Rhizorhabdus dicambivorans T05084 |
| 666685 | Rhodanobacter denitrificans T02495 |
| 441209 | Rhodobaca barguzinensis T05218 |
| 1075 | Rhodobacter blasticus T05669 |
| 272942 | Rhodobacter capsulatus SB 1003 T01213 |
| 1850250 | Rhodobacter sp LPB0142 T04631 |
| 272943 | Rhodobacter sphaeroides 2 4 1 T00284 |
| 349102 | Rhodobacter sphaeroides ATCC 17025 T00512 |
| 349101 | Rhodobacter sphaeroides ATCC 17029 T00484 |
| 557760 | Rhodobacter sphaeroides KD131 T00844 |
| 2605946 | Rhodobacteraceae bacterium SH 1 T05357 |
| 2820523 | Rhodobacteraceae bacterium T05514 |
| 2026785 | Rhodobiaceae bacterium T05596 |
| 191292 | Rhodococcus aetherivorans T03876 |
| 103816 | Rhodococcus biphenylivorans T05830 |
| 1136179 | Rhodococcus erythropolis CCM2595 T02806 |
| 234621 | Rhodococcus erythropolis PR4 T00881 |
| 1833 | Rhodococcus erythropolis T03842 |
| 1051973 | Rhodococcus fascians D188 T04328 |
| 685727 | Rhodococcus hoagii 103S T01352 |
| 101510 | Rhodococcus jostii RHA1 T00382 |
| 632772 | Rhodococcus opacus B4 T00882 |
| 543736 | Rhodococcus opacus PD630 T03071 |
| 1435356 | Rhodococcus pyridinivorans SB3094 T02974 |
| 334542 | Rhodococcus qingshengii T05315 |
| 1829 | Rhodococcus rhodochrous T05521 |
| 1830 | Rhodococcus ruber T05142 |
| 1723645 | Rhodococcus sp 008 T05462 |
| 1564114 | Rhodococcus sp B7740 T03776 |
| 935199 | Rhodococcus sp p52 T05434 |
| 1653478 | Rhodococcus sp PBTS 1 T05182 |
| 1828 | Rhodococcus sp PBTS 2 T05030 |
| 679318 | Rhodococcus sp WMMA185 T04814 |
| 1898103 | Rhodocyclaceae bacterium T05394 |
| 81479 | Rhodoferax antarcticus T04655 |
| 338969 | Rhodoferax ferrireducens T118 T00334 |
| 1842727 | Rhodoferax koreense T04721 |
| 1484693 | Rhodoferax saidenbachensis T04623 |
| 529884 | Rhodoluna lacicola T03203 |
| 648757 | Rhodomicrobium vannielii ATCC 17100 T01353 |
| 243090 | Rhodopirellula baltica SH 1 T00134 |
| 674703 | Rhodoplanes sp Z2 YC6860 T04290 |
| 316055 | Rhodopseudomonas palustris BisA53 T00400 |
| 316056 | Rhodopseudomonas palustris BisB18 T00336 |
| 316057 | Rhodopseudomonas palustris BisB5 T00345 |
| 258594 | Rhodopseudomonas palustris CGA009 T00153 |
| 652103 | Rhodopseudomonas palustris DX 1 T01381 |
| 316058 | Rhodopseudomonas palustris HaA2 T00321 |
| 395960 | Rhodopseudomonas palustris TIE 1 T00722 |
| 414684 | Rhodospirillum centenum SW T00785 |
| 269796 | Rhodospirillum rubrum ATCC 11170 T00310 |
| 1036743 | Rhodospirillum rubrum F11 T02006 |
| 1779382 | Rhodothermaceae bacterium RA T04780 |
| 518766 | Rhodothermus marinus DSM 4252 T01119 |
| 762570 | Rhodothermus marinus SG0 5JP17 172 T01606 |
| 308754 | Rhodovulum sp MB263 T04771 |
| 1564506 | Rhodovulum sp P5 T04813 |
| 35806 | Rhodovulum sulfidophilum T04143 |
| 3988 | Ricinus communis T01087 |
| 347255 | Rickettsia africae ESF 5 T00979 |
| 293614 | Rickettsia akari str Hartford T00601 |
| 1105111 | Rickettsia amblyommatis str GAT 30V T01787 |
| 33989 | Rickettsia amblyommatis T04237 |
| 1105110 | Rickettsia australis str Cutlack T01783 |
| 391896 | Rickettsia bellii OSU 85 389 T00602 |
| 336407 | Rickettsia bellii RML369 C T00339 |
| 1105107 | Rickettsia canadensis str CA410 T01736 |
| 293613 | Rickettsia canadensis str McKiel T00603 |
| 272944 | Rickettsia conorii str Malish 7 T00061 |
| 315456 | Rickettsia felis URRWXCal2 T00252 |
| 1032845 | Rickettsia heilongjiangensis 054 T01587 |
| 652620 | Rickettsia japonica YH T01637 |
| 416276 | Rickettsia massiliae MTU5 T00605 |
| 1105112 | Rickettsia massiliae str AZT80 T01758 |
| 109232 | Rickettsia monacensis T03824 |
| 1105114 | Rickettsia montanensis str OSU 85 930 T01784 |
| 1105108 | Rickettsia parkeri str Portsmouth T01785 |
| 562019 | Rickettsia peacockii str Rustic T00907 |
| 481009 | Rickettsia philipii str 364D T01759 |
| 1290428 | Rickettsia prowazekii str Breinl T02543 |
| 1105096 | Rickettsia prowazekii str BuV67 CWPP T01788 |
| 1105094 | Rickettsia prowazekii str Chernikova T01789 |
| 1105097 | Rickettsia prowazekii str Dachau T01790 |
| 1105098 | Rickettsia prowazekii str GvV257 T01791 |
| 1105095 | Rickettsia prowazekii str Katsinyian T01792 |
| 272947 | Rickettsia prowazekii str Madrid E T00018 |
| 1290427 | Rickettsia prowazekii str NMRC Madrid E T02544 |
| 449216 | Rickettsia prowazekii str Rp22 T01977 |
| 1105099 | Rickettsia prowazekii str RpGvF24 T01793 |
| 1105113 | Rickettsia rhipicephali str 3 7 female6 CWPP T01786 |
| 1105105 | Rickettsia rickettsii str Arizona T01721 |
| 1105104 | Rickettsia rickettsii str Brazil T01737 |
| 1105102 | Rickettsia rickettsii str Colombia T01722 |
| 1105103 | Rickettsia rickettsii str Hauke T01723 |
| 1105100 | Rickettsia rickettsii str Hino T01738 |
| 1105101 | Rickettsia rickettsii str Hlp 2 T01739 |
| 452659 | Rickettsia rickettsii str Iowa T00643 |
| 1337396 | Rickettsia rickettsii str Morgan T03662 |
| 1338411 | Rickettsia rickettsii str R T03663 |
| 392021 | Rickettsia rickettsii str Sheila Smith T00604 |
| 941638 | Rickettsia slovaca 13 B T01671 |
| 1105109 | Rickettsia slovaca str D CWPP T01801 |
| 1182263 | Rickettsia sp MEAM1 T05695 |
| 1003202 | Rickettsia typhi str B9991CWPP T01810 |
| 1003201 | Rickettsia typhi str TH1527 T01802 |
| 257363 | Rickettsia typhi str Wilmington T00193 |
| 1528098 | Rickettsiales bacterium Ac37b T03234 |
| 2486578 | Rickettsiales endosymbiont of Stachyamoeba lipophora T05750 |
| 693978 | Riemerella anatipestifer ATCC 11845 DSM 15868 T01363 |
| 693978 | Riemerella anatipestifer ATCC 11845 DSM 15868 T02107 |
| 1354240 | Riemerella anatipestifer CH3 T03250 |
| 1228997 | Riemerella anatipestifer RA CH 1 T02331 |
| 1271752 | Riemerella anatipestifer RA CH 2 T02438 |
| 992406 | Riemerella anatipestifer RA GD T02007 |
| 41431 | Rippkaea orientalis PCC 8801 T00806 |
| 395962 | Rippkaea orientalis PCC 8802 T00968 |
| 373994 | Rivularia sp PCC 7116 T02364 |
| 313596 | Robiginitalea biformata HTCC2501 T00992 |
| 758 | Rodentibacter pneumotropicus T05800 |
| 76731 | Roseateles depolymerans T04166 |
| 585394 | Roseburia hominis A2 183 T01621 |
| 657315 | Roseburia intestinalis M50 1 T02598 |
| 718255 | Roseburia intestinalis XB6B4 T02597 |
| 1294273 | Roseibacterium elongatum DSM 19469 T03060 |
| 383372 | Roseiflexus castenholzii DSM 13941 T00585 |
| 357808 | Roseiflexus sp RS 1 T00542 |
| 1852022 | Roseitalea porphyridii T05862 |
| 375451 | Roseobacter denitrificans OCh 114 T00376 |
| 391595 | Roseobacter litoralis Och 149 T01572 |
| 257708 | Roseomonas gilardii T04615 |
| 2018065 | Roseomonas sp FDAARGOS 362 T05160 |
| 215743 | Roseovarius mucosus T04779 |
| 2203213 | Roseovarius sp AK1035 T05517 |
| 172042 | Rothia aeria T05268 |
| 762948 | Rothia dentocariosa ATCC 17931 T01343 |
| 680646 | Rothia mucilaginosa DY 18 T01144 |
| 756272 | Rubinisphaera brasiliensis DSM 5305 T01431 |
| 987059 | Rubrivivax benzoatilyticus JA2 ATCC BAA 35 T04092 |
| 983917 | Rubrivivax gelatinosus IL144 T01803 |
| 42256 | Rubrobacter radiotolerans T03080 |
| 266117 | Rubrobacter xylanophilus DSM 9941 T00368 |
| 246200 | Ruegeria pomeroyi DSS 3 T00215 |
| 2293862 | Ruegeria sp AD91A T05627 |
| 292414 | Ruegeria sp TM1040 T00359 |
| 1379909 | Rufibacter sp DG15C T04326 |
| 1379910 | Rufibacter sp DG31D T03996 |
| 512763 | Rufibacter tibetensis T04099 |
| 1565605 | Rugosibacter aromaticivorans T03689 |
| 394503 | Ruminiclostridium cellulolyticum H10 T00835 |
| 1572656 | Ruminococcaceae bacterium CPB6 T04880 |
| 697329 | Ruminococcus albus 7 DSM 20455 T01387 |
| 1160721 | Ruminococcus bicirculans T03158 |
| 213810 | Ruminococcus champanellensis 18P13 JCM 17042 T02600 |
| 657323 | Ruminococcus sp SR1 5 T02601 |
| 657313 | Ruminococcus torques L2 14 T02606 |
| 241244 | Rummeliibacillus stabekisii T04369 |
| 761193 | Runella slithyformis DSM 19594 T01553 |
| 2259595 | Runella sp HYN0085 T05537 |
| 2268026 | Runella sp SP2 T05690 |
| 555311 | Saccharolobus solfataricus 98 2 T02126 |
| 273057 | Saccharolobus solfataricus P2 T00054 |
| 2287 | Saccharolobus solfataricus T03910 |
| 2287 | Saccharolobus solfataricus T03911 |
| 2287 | Saccharolobus solfataricus T03912 |
| 471857 | Saccharomonospora viridis DSM 43017 T00980 |
| 559292 | Saccharomyces cerevisiae S288C T00005 |
| 203122 | Saccharophagus degradans 2 40 T00335 |
| 405948 | Saccharopolyspora erythraea NRRL 2338 T00492 |
| 1179773 | Saccharothrix espanaensis DSM 44229 T02404 |
| 10224 | Saccoglossus kowalevskii T04123 |
| 2009329 | Sagittula sp P11 T05219 |
| 39432 | Saimiri boliviensis boliviensis T04350 |
| 1729720 | Salegentibacter sp T436 T04614 |
| 2099786 | Salicibibacter kimchii T05571 |
| 1230341 | Salimicrobium jeotgali T04359 |
| 1333523 | Salinarchaeum sp Harcht Bsk1 T02693 |
| 309807 | Salinibacter ruber DSM 13855 T00312 |
| 761659 | Salinibacter ruber M8 T01209 |
| 2079791 | Salinibacterium sp CGMCC 1 16371 T05414 |
| 2508880 | Salinibacterium sp UTAS2018 T05816 |
| 407035 | Salinicoccus halodurans T03940 |
| 755307 | Salinigranum rubrum T05289 |
| 2303538 | Salinimonas sediminis T05624 |
| 2183582 | Saliniradius amylolyticus T05447 |
| 2183911 | Salinisphaera sp LB1 T05476 |
| 1307761 | Salinispira pacifica T03013 |
| 391037 | Salinispora arenicola CNS 205 T00613 |
| 369723 | Salinispora tropica CNB 440 T00504 |
| 1307839 | Salinivirga cyanobacteriivorans T04172 |
| 1229727 | Salipiger profundus T04656 |
| 8030 | Salmo salar T04363 |
| 1197719 | Salmonella bongori N268 08 T02733 |
| 218493 | Salmonella bongori NCTC 12419 T01579 |
| 1382510 | Salmonella bongori serovar 48 z41 str RKS3044 T03249 |
| 41514 | Salmonella enterica subsp arizonae serovar 62 z4 z23 T00627 |
| 866913 | Salmonella enterica subsp enterica serovar 4 5 12 i str 08 1736 T02738 |
| 1406860 | Salmonella enterica subsp enterica serovar Agona str 24249 T02944 |
| 454166 | Salmonella enterica subsp enterica serovar Agona str SL483 T00751 |
| 1173427 | Salmonella enterica subsp enterica serovar Bareilly str CFSAN000189 T02739 |
| 1320309 | Salmonella enterica subsp enterica serovar Bovismorbificans str 3114 T02827 |
| 321314 | Salmonella enterica subsp enterica serovar Choleraesuis str SC B67 T00240 |
| 1271863 | Salmonella enterica subsp enterica serovar Cubana str CFSAN002050 T02734 |
| 439851 | Salmonella enterica subsp enterica serovar Dublin str CT 02021853 T00757 |
| 1412460 | Salmonella enterica subsp enterica serovar Enteritidis str EC20090135 T03731 |
| 1412459 | Salmonella enterica subsp enterica serovar Enteritidis str EC20090193 T03732 |
| 1412461 | Salmonella enterica subsp enterica serovar Enteritidis str EC20090332 T03760 |
| 1412462 | Salmonella enterica subsp enterica serovar Enteritidis str EC20090531 T03761 |
| 550537 | Salmonella enterica subsp enterica serovar Enteritidis str P125109 T00776 |
| 149539 | Salmonella enterica subsp enterica serovar Enteritidis T03817 |
| 1225522 | Salmonella enterica subsp enterica serovar Gallinarum pullorum str CDC1983 67 T02824 |
| 1081093 | Salmonella enterica subsp enterica serovar Gallinarum pullorum str RKS5078 T01740 |
| 550538 | Salmonella enterica subsp enterica serovar Gallinarum str 287 91 T00770 |
| 1124936 | Salmonella enterica subsp enterica serovar Heidelberg str 41578 T02736 |
| 1160717 | Salmonella enterica subsp enterica serovar Heidelberg str B182 T02011 |
| 1271864 | Salmonella enterica subsp enterica serovar Heidelberg str CFSAN002069 T02735 |
| 454169 | Salmonella enterica subsp enterica serovar Heidelberg str SL476 T00738 |
| 1267753 | Salmonella enterica subsp enterica serovar Javiana str CFSAN001992 T02479 |
| 423368 | Salmonella enterica subsp enterica serovar Newport str SL254 T00739 |
| 877468 | Salmonella enterica subsp enterica serovar Newport str USMARC S3124 1 T02779 |
| 554290 | Salmonella enterica subsp enterica serovar Paratyphi A str AKU 12601 T00756 |
| 295319 | Salmonella enterica subsp enterica serovar Paratyphi A str ATCC 9150 T00219 |
| 1016998 | Salmonella enterica subsp enterica serovar Paratyphi B str SPB7 T00628 |
| 476213 | Salmonella enterica subsp enterica serovar Paratyphi C str RKS4594 T00862 |
| 1298917 | Salmonella enterica subsp enterica serovar Pullorum str S06004 T02790 |
| 439843 | Salmonella enterica subsp enterica serovar Schwarzengrund str CVM19633 T00743 |
| 1003191 | Salmonella enterica subsp enterica serovar Tennessee str TXSC TXSC08 19 T03730 |
| 1064551 | Salmonella enterica subsp enterica serovar Thompson str RM6836 T02868 |
| 220341 | Salmonella enterica subsp enterica serovar Typhi str CT18 T00064 |
| 1132507 | Salmonella enterica subsp enterica serovar Typhi str P stx 12 T01741 |
| 209261 | Salmonella enterica subsp enterica serovar Typhi str Ty2 T00121 |
| 527001 | Salmonella enterica subsp enterica serovar Typhi str Ty21a T02648 |
| 588858 | Salmonella enterica subsp enterica serovar Typhimurium str 14028S T01714 |
| 1008297 | Salmonella enterica subsp enterica serovar Typhimurium str 798 T01782 |
| 568708 | Salmonella enterica subsp enterica serovar Typhimurium str D23580 T01715 |
| 85569 | Salmonella enterica subsp enterica serovar Typhimurium str DT104 T02874 |
| 568709 | Salmonella enterica subsp enterica serovar Typhimurium str DT2 T02869 |
| 99287 | Salmonella enterica subsp enterica serovar Typhimurium str LT2 T00065 |
| 216597 | Salmonella enterica subsp enterica serovar Typhimurium str SL1344 T01724 |
| 909946 | Salmonella enterica subsp enterica serovar Typhimurium str ST4 74 T01760 |
| 718274 | Salmonella enterica subsp enterica serovar Typhimurium str T000240 T01742 |
| 1171376 | Salmonella enterica subsp enterica serovar Typhimurium str U288 T02639 |
| 990282 | Salmonella enterica subsp enterica serovar Typhimurium str UK 1 T01743 |
| 90371 | Salmonella enterica subsp enterica serovar Typhimurium T03493 |
| 1271862 | Salmonella enterica subsp enterica serovar Typhimurium var 5 str CFSAN001921 T02737 |
| 946362 | Salpingoeca rosetta T04136 |
| 2691554 | Salvelinus alpinus T05762 |
| 927083 | Sandaracinus amylolyticus T03935 |
| 446469 | Sanguibacter keddieii DSM 10542 T01214 |
| 695850 | Saprolegnia parasitica CBS 223 65 T04137 |
| 984262 | Saprospira grandis str Lewin T01764 |
| 9305 | Sarcophilus harrisii T02286 |
| 1150468 | Scardovia inopinata JCM 12537 T03977 |
| 52773 | Schaalia meyeri T04027 |
| 322104 | Scheffersomyces stipitis CBS 6054 T01023 |
| 6185 | Schistosoma haematobium T05004 |
| 6183 | Schistosoma mansoni T01095 |
| 578458 | Schizophyllum commune H4 8 T01331 |
| 284812 | Schizosaccharomyces pombe 972h T00076 |
| 113540 | Scleropages formosus T04919 |
| 665079 | Sclerotinia sclerotiorum 1980 UF 70 T01071 |
| 526218 | Sebaldella termitidis ATCC 33386 T01123 |
| 1199245 | Secondary endosymbiont of Ctenarytaina eucalypti T02199 |
| 134287 | Secondary endosymbiont of Heteropsylla cubana T02336 |
| 1835721 | Secondary endosymbiont of Trabutina mannipara T04494 |
| 1543721 | Sedimenticola thiotaurini T03943 |
| 1940790 | Sedimentisphaera cyanobacteriorum T04695 |
| 1941349 | Sedimentisphaera salicampi T05439 |
| 1453352 | Sediminicola sp YIK13 T04145 |
| 573413 | Sediminispirochaeta smaragdinae DSM 11293 T01287 |
| 640132 | Segniliparus rotundus DSM 44985 T01238 |
| 88036 | Selaginella moellendorffii T01496 |
| 927704 | Selenomonas ruminantium subsp lactilytica TAM6421 T01799 |
| 713030 | Selenomonas sp oral taxon 136 T04745 |
| 712538 | Selenomonas sp oral taxon 478 T04017 |
| 1884263 | Selenomonas sp oral taxon 920 T04818 |
| 546271 | Selenomonas sputigena ATCC 35185 T01474 |
| 1936081 | Seonamhaeicola sp S2 3 T04683 |
| 1758689 | Serinicoccus sp JLT9 T04436 |
| 9135 | Serinus canaria T05755 |
| 41447 | Seriola dumerili T05606 |
| 1458426 | Serpentinomonas mccroryi T03627 |
| 1458425 | Serpentinomonas raichei T03614 |
| 578457 | Serpula lacrymans var lacrymans S7 9 T03135 |
| 47917 | Serratia fonticola T04030 |
| 47917 | Serratia fonticola T04716 |
| 1346614 | Serratia liquefaciens ATCC 27592 T02740 |
| 1334564 | Serratia marcescens SM39 T03592 |
| 273526 | Serratia marcescens subsp marcescens Db11 T03985 |
| 435998 | Serratia marcescens WW4 T02448 |
| 682634 | Serratia plymuthica 4Rx13 T02710 |
| 768492 | Serratia plymuthica AS9 T01503 |
| 1154756 | Serratia plymuthica PRI 2C T04576 |
| 1348660 | Serratia plymuthica S13 T02716 |
| 399741 | Serratia proteamaculans 568 T00595 |
| 61652 | Serratia rubidaea T04417 |
| 768490 | Serratia sp AS12 T01502 |
| 768493 | Serratia sp AS13 T02012 |
| 104623 | Serratia sp ATCC 39006 T05290 |
| 671990 | Serratia sp FGI94 T02433 |
| 1327989 | Serratia sp FS14 T03313 |
| 2033438 | Serratia sp MYb239 T05477 |
| 488142 | Serratia sp SCBI T03314 |
| 568817 | Serratia symbiotica str Cinara cedri T01672 |
| 4182 | Sesamum indicum T04135 |
| 4555 | Setaria italica T02818 |
| 38313 | Shewanella algae T05622 |
| 326297 | Shewanella amazonensis SB2B T00445 |
| 693974 | Shewanella baltica BA175 T02014 |
| 693970 | Shewanella baltica OS117 T02013 |
| 325240 | Shewanella baltica OS155 T00478 |
| 402882 | Shewanella baltica OS185 T00571 |
| 399599 | Shewanella baltica OS195 T00622 |
| 407976 | Shewanella baltica OS223 T00803 |
| 693973 | Shewanella baltica OS678 T01725 |
| 2018305 | Shewanella bicestrii T05197 |
| 318161 | Shewanella denitrificans OS217 T00344 |
| 318167 | Shewanella frigidimarina NCIMB 400 T00395 |
| 458817 | Shewanella halifaxensis HAW EB4 T00654 |
| 93973 | Shewanella japonica T04959 |
| 150120 | Shewanella livingstonensis T05875 |
| 323850 | Shewanella loihica PV 4 T00489 |
| 260364 | Shewanella marisflavi T05400 |
| 211586 | Shewanella oneidensis MR 1 T00099 |
| 398579 | Shewanella pealeana ATCC 700345 T00606 |
| 225849 | Shewanella piezotolerans WP3 T00791 |
| 225848 | Shewanella psychrophila T05063 |
| 399804 | Shewanella putrefaciens 200 T02015 |
| 319224 | Shewanella putrefaciens CN 32 T00513 |
| 425104 | Shewanella sediminis HAW EB3 T00596 |
| 94122 | Shewanella sp ANA 3 T00428 |
| 1930557 | Shewanella sp FDAARGOS 354 T04944 |
| 60480 | Shewanella sp MR 4 T00388 |
| 60481 | Shewanella sp MR 7 T00389 |
| 351745 | Shewanella sp W3 18 1 T00453 |
| 2029986 | Shewanella sp WE21 T05404 |
| 637905 | Shewanella violacea DSS12 T01206 |
| 392500 | Shewanella woodyi ATCC 51908 T00676 |
| 344609 | Shigella boydii CDC 3083 94 T00700 |
| 300268 | Shigella boydii Sb227 T00301 |
| 754093 | Shigella dysenteriae 1617 T02933 |
| 300267 | Shigella dysenteriae Sd197 T00306 |
| 591020 | Shigella flexneri 2002017 T01883 |
| 1282357 | Shigella flexneri 2003036 T03267 |
| 198215 | Shigella flexneri 2a str 2457T T00128 |
| 198214 | Shigella flexneri 2a str 301 T00097 |
| 373384 | Shigella flexneri 5 str 8401 T00377 |
| 1282358 | Shigella flexneri Shi06HN006 T03315 |
| 623 | Shigella flexneri T03751 |
| 300269 | Shigella sonnei Ss046 T00274 |
| 1813821 | Shigella sp PAMC 28760 T04390 |
| 630626 | Shimwellia blattae DSM 4481 NBRC 105725 T02111 |
| 879274 | Shinella sp HZN7 T04278 |
| 1454006 | Siansivirga zeaxanthinifaciens CC SAMT 1 T03699 |
| 580332 | Sideroxydans lithotrophicus ES 1 T01196 |
| 1826607 | Silicimonas algicola T05779 |
| 1117647 | Simiduia agarivorans SA1 DSM 21679 T02281 |
| 331113 | Simkania negevensis Z T01559 |
| 641147 | Simonsiella muelleri ATCC 29453 T05344 |
| 2109915 | Simplicispira suum T05361 |
| 886293 | Singulisphaera acidiphila DSM 18658 T02393 |
| 1608454 | Sinocyclocheilus anshuiensis T04917 |
| 75366 | Sinocyclocheilus grahami T04918 |
| 307959 | Sinocyclocheilus rhinocerous T04916 |
| 37927 | Sinomonas atrocyanea T04286 |
| 194963 | Sinorhizobium americanum T04621 |
| 1117943 | Sinorhizobium fredii HH103 T01731 |
| 394 | Sinorhizobium fredii NGR234 T00888 |
| 1185652 | Sinorhizobium fredii USDA 257 T02188 |
| 366394 | Sinorhizobium medicae WSM419 T00555 |
| 266834 | Sinorhizobium meliloti 1021 T00058 |
| 1286640 | Sinorhizobium meliloti 2011 T02497 |
| 693982 | Sinorhizobium meliloti AK83 T01521 |
| 698936 | Sinorhizobium meliloti BL225C T01884 |
| 1235461 | Sinorhizobium meliloti GR4 T02405 |
| 1230587 | Sinorhizobium meliloti Rm41 T02332 |
| 707241 | Sinorhizobium meliloti SM11 T01885 |
| 382 | Sinorhizobium meliloti T03395 |
| 794846 | Sinorhizobium sp CCBAU 05631 T05069 |
| 1842534 | Sinorhizobium sp RAC02 T04514 |
| 471855 | Slackia heliotrinireducens DSM 20476 T00981 |
| 187101 | Sneathia amnii T03854 |
| 1196094 | Snodgrassella alvi wkB2 T03063 |
| 1929246 | Sodalis endosymbiont of Henestaris halophilus T05311 |
| 343509 | Sodalis glossinidius str morsitans T00316 |
| 1239307 | Sodalis praecaptivus T03004 |
| 4081 | Solanum lycopersicum T02665 |
| 28526 | Solanum pennellii T04130 |
| 4113 | Solanum tuberosum T02981 |
| 13686 | Solenopsis invicta T03916 |
| 1002809 | Solibacillus silvestris StLB046 T02162 |
| 76853 | Solibacillus silvestris T04506 |
| 2048654 | Solibacillus sp R5 41 T05144 |
| 2303331 | Solimonas sp K1W22B 7 T05591 |
| 929556 | Solitalea canadensis DSM 3403 T02084 |
| 448385 | Sorangium cellulosum So ce56 T00634 |
| 1254432 | Sorangium cellulosum So0157 2 T02718 |
| 771870 | Sordaria macrospora k hell T01610 |
| 4558 | Sorghum bicolor T01086 |
| 619300 | Spathaspora passalidarum NRRL Y 27907 T03101 |
| 479434 | Sphaerobacter thermophilus DSM 20745 T01128 |
| 760011 | Sphaerochaeta coccoides DSM 17374 T01469 |
| 158189 | Sphaerochaeta globosa str Buddy T01439 |
| 158190 | Sphaerochaeta pleomorpha str Grapes T01673 |
| 1986952 | Sphingobacteriaceae bacterium GW460 11 11 14 LB5 T05546 |
| 1010 | Sphingobacterium mizutaii T05395 |
| 743722 | Sphingobacterium sp 21 T01450 |
| 1933220 | Sphingobacterium sp B29 T04670 |
| 1538644 | Sphingobacterium sp ML3W T03258 |
| 1332080 | Sphingobium baderi T04194 |
| 690566 | Sphingobium chlorophenolicum L 1 T01522 |
| 120107 | Sphingobium cloacae T05301 |
| 121428 | Sphingobium hydrophobicum T05146 |
| 861109 | Sphingobium indicum B90A T04604 |
| 452662 | Sphingobium japonicum UT26S T01201 |
| 1855519 | Sphingobium sp EP60837 T04432 |
| 407020 | Sphingobium sp MI1205 T04383 |
| 1843368 | Sphingobium sp RAC03 T04461 |
| 627192 | Sphingobium sp SYK 6 T01622 |
| 1315974 | Sphingobium sp TKS T04965 |
| 484429 | Sphingobium sp YBL2 T03855 |
| 2082188 | Sphingobium sp YG1 T05858 |
| 13690 | Sphingobium yanoikuyae T05170 |
| 1609977 | Sphingomonas hengshuiensis T03848 |
| 93064 | Sphingomonas koreensis T04828 |
| 621456 | Sphingomonas melonis TY T04509 |
| 1560345 | Sphingomonas panacis T04760 |
| 1123269 | Sphingomonas sanxanigenens DSM 19645 NX02 T03929 |
| 1327635 | Sphingomonas sp Cra20 T05307 |
| 2219696 | Sphingomonas sp FARSPH T05685 |
| 1030157 | Sphingomonas sp KC8 T04987 |
| 1390395 | Sphingomonas sp LK11 T04954 |
| 1938607 | Sphingomonas sp LM7 T04824 |
| 745310 | Sphingomonas sp MM 1 T02498 |
| 1961362 | Sphingomonas sp NIC1 T04479 |
| 2319844 | Sphingomonas sp YZ 8 T05706 |
| 1549858 | Sphingomonas taxi T03398 |
| 1283312 | Sphingomonas wittichii DC 6 T05200 |
| 392499 | Sphingomonas wittichii RW1 T00538 |
| 317655 | Sphingopyxis alaskensis RB2256 T00361 |
| 1515612 | Sphingopyxis fribergensis T03542 |
| 267128 | Sphingopyxis granuli T04372 |
| 33050 | Sphingopyxis macrogoltabida T04104 |
| 33050 | Sphingopyxis macrogoltabida T04627 |
| 292913 | Sphingopyxis sp 113P3 T04069 |
| 1913578 | Sphingopyxis sp LPB0140 T04602 |
| 1866325 | Sphingopyxis sp MG T05382 |
| 1357916 | Sphingopyxis sp QXT 31 T04913 |
| 1219058 | Sphingopyxis terrae subsp terrae NBRC 15098 T04307 |
| 1806885 | Sphingorhabdus sp M41 T04317 |
| 2077182 | Sphingorhabdus sp YGSMI21 T05308 |
| 335406 | Sphingosinicella microcystinivorans T05811 |
| 1892855 | Sphingosinicella sp BN140058 T05826 |
| 3562 | Spinacia oleracea T05137 |
| 1335757 | Spiribacter curvatus T02888 |
| 1260251 | Spiribacter salinus M19 40 T02685 |
| 889378 | Spirochaeta africana DSM 8902 T01811 |
| 665571 | Spirochaeta thermophila DSM 6192 T01315 |
| 869211 | Spirochaeta thermophila DSM 6578 T02016 |
| 1276258 | Spiroplasma apis B31 T02945 |
| 1114980 | Spiroplasma atrichopogonis T03973 |
| 362837 | Spiroplasma cantharicola T04084 |
| 1276227 | Spiroplasma chrysopicola DF 1 T02676 |
| 2133 | Spiroplasma citri T04628 |
| 2139 | Spiroplasma clarkii T05330 |
| 216934 | Spiroplasma corruscae T05302 |
| 1276246 | Spiroplasma culicicola AES 1 T03047 |
| 1276221 | Spiroplasma diminutum CUAS 1 T02741 |
| 315358 | Spiroplasma eriocheiris T03974 |
| 1336749 | Spiroplasma floricola 23 6 T05283 |
| 216938 | Spiroplasma helicoides T04620 |
| 273035 | Spiroplasma kunkelii CR2 3x T04056 |
| 216942 | Spiroplasma litorale T04047 |
| 838561 | Spiroplasma mirum ATCC 29335 T03003 |
| 838561 | Spiroplasma mirum ATCC 29335 T03382 |
| 1276257 | Spiroplasma sabaudiense Ar 1343 T03048 |
| 1914410 | Spiroplasma sp NBRC 100390 T05859 |
| 1276229 | Spiroplasma syrphidicola EA 1 T02677 |
| 1276220 | Spiroplasma taiwanense CT 1 T02742 |
| 216946 | Spiroplasma turonicum T04034 |
| 504472 | Spirosoma linguale DSM 74 T01156 |
| 1178516 | Spirosoma montaniterrae T04702 |
| 2057025 | Spirosoma pollinicola T05236 |
| 1379870 | Spirosoma radiotolerans T03819 |
| 1620392 | Spongiibacter sp IMCC21906 T03945 |
| 269673 | Sporolactobacillus terrae T05792 |
| 1476 | Sporosarcina psychrophila T04327 |
| 1930764 | Sporosarcina sp P33 T04790 |
| 1930546 | Sporosarcina sp P37 T04826 |
| 2283194 | Sporosarcina sp PTS2304 T05572 |
| 1571 | Sporosarcina ureae T04877 |
| 1397361 | Sporothrix schenckii 1099 18 T05555 |
| 446470 | Stackebrandtia nassauensis DSM 44728 T01194 |
| 111780 | Stanieria cyanosphaera PCC 7437 T02406 |
| 1807358 | Stanieria sp NIES 3757 T05130 |
| 985762 | Staphylococcus agnetis T04155 |
| 985002 | Staphylococcus argenteus T01767 |
| 703339 | Staphylococcus aureus 04 02981 T02070 |
| 1229492 | Staphylococcus aureus 08BA02176 T02339 |
| 1321369 | Staphylococcus aureus Bmb9393 T02717 |
| 1323661 | Staphylococcus aureus CA 347 T02702 |
| 1305598 | Staphylococcus aureus M1 T02618 |
| 273036 | Staphylococcus aureus RF122 T00303 |
| 1123523 | Staphylococcus aureus subsp aureus 11819 97 T01950 |
| 585143 | Staphylococcus aureus subsp aureus 55 2053 T02810 |
| 1392476 | Staphylococcus aureus subsp aureus 6850 T02825 |
| 1155084 | Staphylococcus aureus subsp aureus 71193 T02043 |
| 1193576 | Staphylococcus aureus subsp aureus CN1 T02826 |
| 93062 | Staphylococcus aureus subsp aureus COL T00225 |
| 889933 | Staphylococcus aureus subsp aureus ECT R 2 T01887 |
| 685039 | Staphylococcus aureus subsp aureus ED133 T01869 |
| 681288 | Staphylococcus aureus subsp aureus ED98 T01124 |
| 1074252 | Staphylococcus aureus subsp aureus HO 5096 0412 T02059 |
| 359787 | Staphylococcus aureus subsp aureus JH1 T00556 |
| 359786 | Staphylococcus aureus subsp aureus JH9 T00522 |
| 869816 | Staphylococcus aureus subsp aureus JKD6159 T01870 |
| 985006 | Staphylococcus aureus subsp aureus LGA251 T02085 |
| 1118959 | Staphylococcus aureus subsp aureus M013 T01744 |
| 282458 | Staphylococcus aureus subsp aureus MRSA252 T00182 |
| 282459 | Staphylococcus aureus subsp aureus MSSA476 T00183 |
| 418127 | Staphylococcus aureus subsp aureus Mu3 T00588 |
| 158878 | Staphylococcus aureus subsp aureus Mu50 T00052 |
| 196620 | Staphylococcus aureus subsp aureus MW2 T00086 |
| 158879 | Staphylococcus aureus subsp aureus N315 T00051 |
| 93061 | Staphylococcus aureus subsp aureus NCTC 8325 T00324 |
| 1368166 | Staphylococcus aureus subsp aureus SA268 T03790 |
| 1194085 | Staphylococcus aureus subsp aureus SA40 T02849 |
| 1201010 | Staphylococcus aureus subsp aureus SA957 T02850 |
| 1074919 | Staphylococcus aureus subsp aureus ST228 T03143 |
| 1074919 | Staphylococcus aureus subsp aureus ST228 T03144 |
| 1074919 | Staphylococcus aureus subsp aureus ST228 T03145 |
| 1074919 | Staphylococcus aureus subsp aureus ST228 T03146 |
| 1074919 | Staphylococcus aureus subsp aureus ST228 T03147 |
| 1074919 | Staphylococcus aureus subsp aureus ST228 T03148 |
| 1074919 | Staphylococcus aureus subsp aureus ST228 T03149 |
| 1074919 | Staphylococcus aureus subsp aureus ST228 T03150 |
| 523796 | Staphylococcus aureus subsp aureus ST398 T02071 |
| 546342 | Staphylococcus aureus subsp aureus str JKD6008 T01886 |
| 426430 | Staphylococcus aureus subsp aureus str Newman T00557 |
| 1006543 | Staphylococcus aureus subsp aureus T0131 T01888 |
| 1280 | Staphylococcus aureus subsp aureus T03492 |
| 548473 | Staphylococcus aureus subsp aureus TCH60 T01889 |
| 663951 | Staphylococcus aureus subsp aureus TW20 T02060 |
| 451515 | Staphylococcus aureus subsp aureus USA300 FPR3757 T00323 |
| 451516 | Staphylococcus aureus subsp aureus USA300 TCH1516 T00629 |
| 1028799 | Staphylococcus aureus subsp aureus VC40 T01745 |
| 1406863 | Staphylococcus aureus subsp aureus Z172 T02886 |
| 1280 | Staphylococcus aureus T03491 |
| 1280 | Staphylococcus aureus T04800 |
| 1458279 | Staphylococcus aureus USA300 ISMMS1 T03115 |
| 72758 | Staphylococcus capitis subsp capitis T03970 |
| 396513 | Staphylococcus carnosus subsp carnosus TM300 T00863 |
| 29382 | Staphylococcus cohnii T05008 |
| 70255 | Staphylococcus condimenti T04648 |
| 176280 | Staphylococcus epidermidis ATCC 12228 T00110 |
| 1449752 | Staphylococcus epidermidis PM221 T03289 |
| 176279 | Staphylococcus epidermidis RP62A T00229 |
| 1282 | Staphylococcus epidermidis T03343 |
| 246432 | Staphylococcus equorum T04160 |
| 46127 | Staphylococcus felis T05459 |
| 279808 | Staphylococcus haemolyticus JCSC1435 T00250 |
| 1283 | Staphylococcus haemolyticus T03837 |
| 1284 | Staphylococcus hyicus T03578 |
| 29384 | Staphylococcus kloosii T05401 |
| 698737 | Staphylococcus lugdunensis HKU09 01 T01177 |
| 1034809 | Staphylococcus lugdunensis N920143 T01951 |
| 155085 | Staphylococcus lutrae T04991 |
| 214473 | Staphylococcus nepalensis T05272 |
| 1276282 | Staphylococcus pasteuri SP1 T02889 |
| 170573 | Staphylococcus pettenkoferi T04943 |
| 984892 | Staphylococcus pseudintermedius ED99 T02008 |
| 937773 | Staphylococcus pseudintermedius HKU10 03 T01396 |
| 342451 | Staphylococcus saprophyticus subsp saprophyticus ATCC 15305 NCTC 7292 T00269 |
| 1295 | Staphylococcus schleiferi T04012 |
| 1295 | Staphylococcus schleiferi T04520 |
| 1296 | Staphylococcus sciuri T05176 |
| 1286 | Staphylococcus simulans T04303 |
| 1194526 | Staphylococcus warneri SG1 T02449 |
| 1288 | Staphylococcus xylosus T03248 |
| 1288 | Staphylococcus xylosus T03311 |
| 1288 | Staphylococcus xylosus T03742 |
| 591019 | Staphylothermus hellenicus DSM 12710 T01249 |
| 399550 | Staphylothermus marinus F1 T00485 |
| 639283 | Starkeya novella DSM 506 T01258 |
| 128780 | Stenotrophomonas acidaminiphila T04107 |
| 1163399 | Stenotrophomonas maltophilia D457 T02061 |
| 868597 | Stenotrophomonas maltophilia JV3 T01596 |
| 522373 | Stenotrophomonas maltophilia K279a T00718 |
| 391008 | Stenotrophomonas maltophilia R551 3 T00733 |
| 216778 | Stenotrophomonas rhizophila T04500 |
| 1793721 | Stenotrophomonas sp KCTC 12332 T04408 |
| 1904944 | Stenotrophomonas sp LM091 T04630 |
| 1827305 | Stenotrophomonas sp MYb57 T05384 |
| 2005046 | Stenotrophomonas sp WZN 1 T04963 |
| 721885 | Stereum hirsutum FP 91666 SS1 T03127 |
| 465721 | Steroidobacter denitrificans T04296 |
| 378806 | Stigmatella aurantiaca DW4 3 1 T01425 |
| 980422 | Strawberry lethal yellows phytoplasma str NZSb11 T02674 |
| 2126346 | Streptacidiphilus bronchialis T05561 |
| 519441 | Streptobacillus moniliformis DSM 12112 T01125 |
| 1309806 | Streptococcus agalactiae 09mas018883 T02703 |
| 1417990 | Streptococcus agalactiae 138P T03183 |
| 1261567 | Streptococcus agalactiae 2 22 T02656 |
| 208435 | Streptococcus agalactiae 2603V R T00091 |
| 205921 | Streptococcus agalactiae A909 T00278 |
| 1427374 | Streptococcus agalactiae CNCTC 10 84 T03518 |
| 342616 | Streptococcus agalactiae COH1 T03434 |
| 1203670 | Streptococcus agalactiae GD201008 001 T02248 |
| 1309807 | Streptococcus agalactiae ILRI005 T02704 |
| 1318615 | Streptococcus agalactiae ILRI112 T02705 |
| 211110 | Streptococcus agalactiae NEM316 T00104 |
| 1311 | Streptococcus agalactiae T02338 |
| 1311 | Streptococcus agalactiae T03383 |
| 1311 | Streptococcus agalactiae T03516 |
| 1311 | Streptococcus agalactiae T03517 |
| 862970 | Streptococcus anginosus C1051 T02828 |
| 862971 | Streptococcus anginosus C238 T02829 |
| 1328 | Streptococcus anginosus T03658 |
| 862969 | Streptococcus constellatus subsp pharyngis C1050 T02832 |
| 696216 | Streptococcus constellatus subsp pharyngis C232 T02833 |
| 862968 | Streptococcus constellatus subsp pharyngis C818 T02834 |
| 1302863 | Streptococcus cristatus AS 1 3089 T02649 |
| 1247189 | Streptococcus dysgalactiae subsp equisimilis 167 T02870 |
| 759913 | Streptococcus dysgalactiae subsp equisimilis AC 2713 T02333 |
| 663954 | Streptococcus dysgalactiae subsp equisimilis ATCC 12394 T02017 |
| 486410 | Streptococcus dysgalactiae subsp equisimilis GGS 124 T00932 |
| 617121 | Streptococcus dysgalactiae subsp equisimilis RE378 T02269 |
| 553482 | Streptococcus equi subsp equi 4047 T00877 |
| 1051072 | Streptococcus equi subsp zooepidemicus ATCC 35246 T02355 |
| 1403449 | Streptococcus equi subsp zooepidemicus CY T03306 |
| 552526 | Streptococcus equi subsp zooepidemicus MGCS10565 T00746 |
| 40041 | Streptococcus equi subsp zooepidemicus T00871 |
| 981539 | Streptococcus gallolyticus subsp gallolyticus ATCC 43143 T02018 |
| 990317 | Streptococcus gallolyticus subsp gallolyticus ATCC BAA 2069 T01444 |
| 637909 | Streptococcus gallolyticus UCN34 T01171 |
| 467705 | Streptococcus gordonii str Challis substr CH1 T00594 |
| 1069533 | Streptococcus infantarius subsp infantarius CJ18 T01765 |
| 1318633 | Streptococcus iniae SF1 T02686 |
| 1346 | Streptococcus iniae T03285 |
| 1346 | Streptococcus iniae T03286 |
| 1346 | Streptococcus iniae T03674 |
| 862967 | Streptococcus intermedius B196 T02830 |
| 862966 | Streptococcus intermedius C270 T02831 |
| 591365 | Streptococcus intermedius JTH08 T02156 |
| 1076934 | Streptococcus lutetiensis 033 T02775 |
| 1116231 | Streptococcus macedonicus ACA DC 198 T01674 |
| 365659 | Streptococcus mitis B6 T01173 |
| 1198676 | Streptococcus mutans GS 5 T02185 |
| 1155071 | Streptococcus mutans LJ23 T02086 |
| 511691 | Streptococcus mutans NN2025 T01185 |
| 1437447 | Streptococcus mutans UA159 FR T03797 |
| 210007 | Streptococcus mutans UA159 T00100 |
| 927666 | Streptococcus oralis Uo5 T01476 |
| 1811193 | Streptococcus pantholopis T05054 |
| 760570 | Streptococcus parasanguinis ATCC 15912 T01545 |
| 1114965 | Streptococcus parasanguinis FW213 T02115 |
| 936154 | Streptococcus parauberis KCTC 11537 T01504 |
| 981540 | Streptococcus pasteurianus ATCC 43144 T01524 |
| 189423 | Streptococcus pneumoniae 670 6B T01308 |
| 488221 | Streptococcus pneumoniae 70585 T00872 |
| 1408179 | Streptococcus pneumoniae A026 T02890 |
| 574093 | Streptococcus pneumoniae AP200 T01309 |
| 561276 | Streptococcus pneumoniae ATCC 700669 T00843 |
| 516950 | Streptococcus pneumoniae CGSP14 T00690 |
| 373153 | Streptococcus pneumoniae D39 T00413 |
| 512566 | Streptococcus pneumoniae G54 T00734 |
| 697283 | Streptococcus pneumoniae gamPNI0373 T02334 |
| 487214 | Streptococcus pneumoniae Hungary19A 6 T00658 |
| 869269 | Streptococcus pneumoniae INV104 T02072 |
| 869216 | Streptococcus pneumoniae INV200 T02073 |
| 488222 | Streptococcus pneumoniae JJA T00873 |
| 869215 | Streptococcus pneumoniae OXC141 T02074 |
| 488223 | Streptococcus pneumoniae P1031 T00874 |
| 171101 | Streptococcus pneumoniae R6 T00060 |
| 869303 | Streptococcus pneumoniae SPN034156 T02628 |
| 869304 | Streptococcus pneumoniae SPN034183 T02629 |
| 869306 | Streptococcus pneumoniae SPN994038 T02630 |
| 869307 | Streptococcus pneumoniae SPN994039 T02631 |
| 869309 | Streptococcus pneumoniae SPNA45 T02245 |
| 1130804 | Streptococcus pneumoniae ST556 T02087 |
| 487213 | Streptococcus pneumoniae Taiwan19F 14 T00875 |
| 525381 | Streptococcus pneumoniae TCH8431 19A T01259 |
| 170187 | Streptococcus pneumoniae TIGR4 T00057 |
| 1054460 | Streptococcus pseudopneumoniae IS7493 T01590 |
| 1235829 | Streptococcus pyogenes A20 T02335 |
| 487215 | Streptococcus pyogenes Alab49 T02019 |
| 1336746 | Streptococcus pyogenes HSC5 T02743 |
| 1207470 | Streptococcus pyogenes M1 476 T02499 |
| 160490 | Streptococcus pyogenes M1 GAS T00050 |
| 370552 | Streptococcus pyogenes MGAS10270 T00355 |
| 286636 | Streptococcus pyogenes MGAS10394 T00191 |
| 370554 | Streptococcus pyogenes MGAS10750 T00356 |
| 798300 | Streptococcus pyogenes MGAS15252 T01794 |
| 1010840 | Streptococcus pyogenes MGAS1882 T02062 |
| 370553 | Streptococcus pyogenes MGAS2096 T00357 |
| 198466 | Streptococcus pyogenes MGAS315 T00089 |
| 293653 | Streptococcus pyogenes MGAS5005 T00262 |
| 319701 | Streptococcus pyogenes MGAS6180 T00263 |
| 186103 | Streptococcus pyogenes MGAS8232 T00079 |
| 370551 | Streptococcus pyogenes MGAS9429 T00358 |
| 471876 | Streptococcus pyogenes NZ131 T00780 |
| 193567 | Streptococcus pyogenes SSI 1 T00119 |
| 1440772 | Streptococcus pyogenes STAB901 T03472 |
| 160491 | Streptococcus pyogenes str Manfredo T00497 |
| 1046629 | Streptococcus salivarius 57 I T02088 |
| 1048332 | Streptococcus salivarius CCHSS3 T01580 |
| 347253 | Streptococcus salivarius JIM8777 T02089 |
| 1304 | Streptococcus salivarius T03511 |
| 1304 | Streptococcus salivarius T04634 |
| 388919 | Streptococcus sanguinis SK36 T00473 |
| 1310 | Streptococcus sobrinus T05567 |
| 1759399 | Streptococcus sp A12 T05522 |
| 1156431 | Streptococcus sp I G2 T02879 |
| 1156431 | Streptococcus sp I P16 T02880 |
| 1902136 | Streptococcus sp NPS 308 T05541 |
| 1419814 | Streptococcus sp VT 162 T03287 |
| 391295 | Streptococcus suis 05ZYH33 T00514 |
| 391296 | Streptococcus suis 98HAH33 T00515 |
| 993512 | Streptococcus suis A7 T02020 |
| 568814 | Streptococcus suis BM407 T00935 |
| 1004952 | Streptococcus suis D12 T02021 |
| 1005042 | Streptococcus suis D9 T02022 |
| 423211 | Streptococcus suis GZ1 T02023 |
| 945704 | Streptococcus suis JS14 T02024 |
| 218494 | Streptococcus suis P1 7 T00936 |
| 1184252 | Streptococcus suis S735 T02214 |
| 1246365 | Streptococcus suis SC070731 T02536 |
| 568813 | Streptococcus suis SC84 T00937 |
| 1005041 | Streptococcus suis SS12 T02025 |
| 1004951 | Streptococcus suis ST1 T02125 |
| 1007064 | Streptococcus suis ST3 T01470 |
| 1340847 | Streptococcus suis T15 T02891 |
| 1276647 | Streptococcus suis TL13 T02672 |
| 1380773 | Streptococcus suis YB51 T03173 |
| 1408178 | Streptococcus thermophilus ASCC 1275 T03360 |
| 299768 | Streptococcus thermophilus CNRZ1066 T00212 |
| 1051074 | Streptococcus thermophilus JIM 8232 T02026 |
| 322159 | Streptococcus thermophilus LMD 9 T00414 |
| 264199 | Streptococcus thermophilus LMG 18311 T00213 |
| 1187956 | Streptococcus thermophilus MN ZLW 002 T02116 |
| 767463 | Streptococcus thermophilus ND03 T01978 |
| 1308 | Streptococcus thermophilus T04785 |
| 218495 | Streptococcus uberis 0140J T00849 |
| 2498135 | Streptomonospora sp M2 T05863 |
| 1886 | Streptomyces albidoflavus T02545 |
| 1940 | Streptomyces albireticuli T05230 |
| 67267 | Streptomyces alboflavus T05221 |
| 1971 | Streptomyces albulus T03082 |
| 1434306 | Streptomyces albulus ZPM T03896 |
| 1888 | Streptomyces albus T03823 |
| 1642299 | Streptomyces alfalfae T05110 |
| 278992 | Streptomyces ambofaciens ATCC 23877 T04055 |
| 75293 | Streptomyces autolyticus T04980 |
| 227882 | Streptomyces avermitilis MA 4680 NBRC 14893 T00126 |
| 749414 | Streptomyces bingchenggensis BCW 1 T01678 |
| 1003195 | Streptomyces cattleya NRRL 8057 DSM 46488 T01646 |
| 1003195 | Streptomyces cattleya NRRL 8057 DSM 46488 T02095 |
| 1901 | Streptomyces clavuligerus T04717 |
| 100226 | Streptomyces coelicolor A3 T00085 |
| 1214242 | Streptomyces collinus Tu 365 T02787 |
| 477245 | Streptomyces cyaneogriseus subsp noncyanogenus T03687 |
| 1214101 | Streptomyces davaonensis JCM 4913 T02518 |
| 1616117 | Streptomyces formicae T05416 |
| 1303692 | Streptomyces fulvissimus DSM 40593 T02650 |
| 553510 | Streptomyces gilvosporeus T05165 |
| 1907 | Streptomyces glaucescens T03327 |
| 1172567 | Streptomyces globisporus C 1027 T04209 |
| 68214 | Streptomyces griseochromogenes T04729 |
| 455632 | Streptomyces griseus subsp griseus NBRC 13350 T00691 |
| 1133850 | Streptomyces hygroscopicus subsp jinggangensis 5008 T02063 |
| 1203460 | Streptomyces hygroscopicus subsp jinggangensis TL01 T02531 |
| 39478 | Streptomyces laurentii T05205 |
| 58340 | Streptomyces lavendulae subsp lavendulae T05314 |
| 1437453 | Streptomyces leeuwenhoekii T04414 |
| 1915 | Streptomyces lincolnensis T04801 |
| 457428 | Streptomyces lividans TK24 T03222 |
| 47763 | Streptomyces lydicus T03756 |
| 47763 | Streptomyces lydicus T04699 |
| 92644 | Streptomyces malaysiensis T05189 |
| 1827580 | Streptomyces nigra T05839 |
| 193462 | Streptomyces niveus T04887 |
| 316284 | Streptomyces noursei ATCC 11455 T04806 |
| 146923 | Streptomyces parvulus T04685 |
| 1355015 | Streptomyces pluripotens T04845 |
| 591167 | Streptomyces pratensis ATCC 33331 T01647 |
| 38300 | Streptomyces pristinaespiralis T04073 |
| 1904616 | Streptomyces puniciscabiei T05140 |
| 1783515 | Streptomyces qaidamensis T04777 |
| 1343740 | Streptomyces rapamycinicus NRRL 5491 T02907 |
| 1926 | Streptomyces reticuli T04246 |
| 285473 | Streptomyces rubrolavendulae T04616 |
| 680198 | Streptomyces scabiei 87 22 T01187 |
| 1751294 | Streptomyces sp 4F T04265 |
| 1262452 | Streptomyces sp 769 T03587 |
| 1561022 | Streptomyces sp CCM MD2014 T04862 |
| 1725411 | Streptomyces sp CdTB01 T04240 |
| 1649184 | Streptomyces sp CFMR 7 T04074 |
| 444103 | Streptomyces sp CNQ 509 T03968 |
| 465541 | Streptomyces sp Mg1 T03958 |
| 1961713 | Streptomyces sp MOE7 T05374 |
| 1265601 | Streptomyces sp PAMC 26508 T02619 |
| 1849967 | Streptomyces sp SAT1 T04706 |
| 862751 | Streptomyces sp SirexAA E T01601 |
| 953739 | Streptomyces venezuelae ATCC 10712 T02274 |
| 362257 | Streptomyces vietnamensis T03586 |
| 1935 | Streptomyces violaceoruber T05124 |
| 653045 | Streptomyces violaceusniger Tu 4113 T01602 |
| 408015 | Streptomyces xiamenensis T03904 |
| 479432 | Streptosporangium roseum DSM 43021 T01137 |
| 7668 | Strongylocentrotus purpuratus T01019 |
| 50429 | Stylophora pistillata T05774 |
| 796027 | Sugiyamaella lignohabitans T05270 |
| 2036206 | Suicoccus acidiformans T05652 |
| 421000 | Sulfitobacter donghicola T05893 |
| 1402135 | Sulfitobacter pseudonitzschiae T04974 |
| 1917485 | Sulfitobacter sp AM1 D1 T04555 |
| 1968541 | Sulfitobacter sp D7 T05714 |
| 2070369 | Sulfitobacter sp JL08 T05585 |
| 1389005 | Sulfitobacter sp SK012 T05563 |
| 679936 | Sulfobacillus acidophilus DSM 10332 T01746 |
| 1051632 | Sulfobacillus acidophilus TPY T01576 |
| 1670455 | Sulfodiicoccus acidiphilus T05813 |
| 330779 | Sulfolobus acidocaldarius DSM 639 T00251 |
| 1028566 | Sulfolobus acidocaldarius N8 T02452 |
| 1028567 | Sulfolobus acidocaldarius Ron12 I T02453 |
| 1435377 | Sulfolobus acidocaldarius SUSAZ T02975 |
| 930943 | Sulfolobus islandicus HVE10 4 T01952 |
| 425944 | Sulfolobus islandicus L D 8 5 T01158 |
| 429572 | Sulfolobus islandicus L S 2 15 T00889 |
| 1241935 | Sulfolobus islandicus LAL14 1 T02620 |
| 427317 | Sulfolobus islandicus M 14 25 T00890 |
| 427318 | Sulfolobus islandicus M 16 27 T00900 |
| 426118 | Sulfolobus islandicus M 16 4 T00906 |
| 930945 | Sulfolobus islandicus REY15A T01953 |
| 439386 | Sulfolobus islandicus Y G 57 14 T00901 |
| 419942 | Sulfolobus islandicus Y N 15 51 T00902 |
| 1891280 | Sulfolobus sp A20 T04516 |
| 1620215 | Sulfuricaulis limicola T05241 |
| 1163617 | Sulfuricella denitrificans skB26 T02857 |
| 709032 | Sulfuricurvum kujiense DSM 16994 T01370 |
| 1985873 | Sulfuriferula sp AH1 T04857 |
| 1675686 | Sulfurifustis variabilis T05321 |
| 204536 | Sulfurihydrogenibium azorense Az Fu1 T00876 |
| 436114 | Sulfurihydrogenibium sp YO3AOP1 T00715 |
| 563040 | Sulfurimonas autotrophica DSM 16294 T01316 |
| 326298 | Sulfurimonas denitrificans DSM 1251 T00298 |
| 273063 | Sulfurisphaera tokodaii str 7 T00062 |
| 1223802 | Sulfuritalea hydrogenivorans sk43H T03591 |
| 760154 | Sulfurospirillum barnesii SES 3 T02139 |
| 525898 | Sulfurospirillum deleyianum DSM 6946 T01126 |
| 1193502 | Sulfurospirillum halorespirans DSM 13726 T04475 |
| 1150621 | Sulfurospirillum multivorans DSM 12446 T03049 |
| 1854492 | Sulfurospirillum sp JPD 1 T05109 |
| 1854492 | Sulfurospirillum sp SL2 1 T04910 |
| 206403 | Sulfurovum lithotrophicum T03877 |
| 387093 | Sulfurovum sp NBC37 1 T00572 |
| 9823 | Sus scrofa T01009 |
| 2494234 | Sutterella megalosphaeroides T05697 |
| 292459 | Symbiobacterium thermophilum IAM 14863 T00196 |
| 269084 | Synechococcus elongatus PCC 6301 T00163 |
| 1140 | Synechococcus elongatus PCC 7942 FACHB 805 T00300 |
| 1917166 | Synechococcus lividus PCC 6715 T05303 |
| 64471 | Synechococcus sp CC9311 T00390 |
| 110662 | Synechococcus sp CC9605 T00292 |
| 316279 | Synechococcus sp CC9902 T00293 |
| 321332 | Synechococcus sp JA 2 3B a T00319 |
| 321327 | Synechococcus sp JA 3 3Ab T00318 |
| 1280380 | Synechococcus sp KORDI 100 T03344 |
| 585423 | Synechococcus sp KORDI 49 T03345 |
| 585425 | Synechococcus sp KORDI 52 T03346 |
| 195253 | Synechococcus sp PCC 6312 T02370 |
| 32049 | Synechococcus sp PCC 7002 T00664 |
| 374982 | Synechococcus sp PCC 73109 T05693 |
| 1173263 | Synechococcus sp PCC 7502 T02371 |
| 316278 | Synechococcus sp RCC307 T00523 |
| 1350461 | Synechococcus sp UTEX 2973 T03581 |
| 32051 | Synechococcus sp WH 7803 T00524 |
| 84588 | Synechococcus sp WH 8102 T00144 |
| 29410 | Synechococcus sp WH 8103 T04859 |
| 166314 | Synechococcus sp WH 8109 T03783 |
| 2116702 | Synechocystis sp IPPAS B 1465 T05368 |
| 1147 | Synechocystis sp PCC 6714 T03386 |
| 1080228 | Synechocystis sp PCC 6803 substr GT I T01777 |
| 1080229 | Synechocystis sp PCC 6803 substr PCC N T01778 |
| 1080230 | Synechocystis sp PCC 6803 substr PCC P T01891 |
| 1148 | Synechocystis sp PCC 6803 T00004 |
| 1148 | Synechocystis sp PCC 6803 T02098 |
| 1148 | Synechocystis sp PCC 6803 T02468 |
| 335543 | Syntrophobacter fumaroxidans MPOB T00418 |
| 645991 | Syntrophobotulus glycolicus DSM 8271 T01429 |
| 335541 | Syntrophomonas wolfei subsp wolfei str Goettingen G311 T00396 |
| 643648 | Syntrophothermus lipocalidus DSM 12680 T01253 |
| 56780 | Syntrophus aciditrophicus SB T00346 |
| 2494374 | Tabrizicola piscis T05742 |
| 59729 | Taeniopygia guttata T01082 |
| 31033 | Takifugu rubripes T02386 |
| 2069432 | Tamlana sp UJ94 T05295 |
| 203275 | Tannerella forsythia 92A2 T01682 |
| 712710 | Tannerella sp oral taxon HOT 286 T04474 |
| 1921510 | Tardibacter chloracetimidivorans T04967 |
| 28532 | Tarenaya hassleriana T04125 |
| 299262 | Tateyamaria omphalii T04652 |
| 451 | Tatlockia micdadei T03810 |
| 53336 | Tatumella citrea T04873 |
| 82987 | Tatumella ptyseos T05493 |
| 1091495 | Taylorella asinigenitalis 14 45 T02603 |
| 1008459 | Taylorella asinigenitalis MCE3 T01634 |
| 1091497 | Taylorella equigenitalis 14 56 T02604 |
| 743973 | Taylorella equigenitalis ATCC 35865 T02186 |
| 937774 | Taylorella equigenitalis MCE9 T01390 |
| 669041 | Tenacibaculum dicentrarchi T04208 |
| 584609 | Tenacibaculum jejuense T05041 |
| 1349785 | Tenacibaculum maritimum NCIMB 2154 T05240 |
| 1850252 | Tenacibaculum todarodis T04588 |
| 1911684 | Tenericutes bacterium MO XQ T05457 |
| 1911683 | Tenericutes bacterium MZ XQ T05490 |
| 1209989 | Tepidanaerobacter acetatoxydans Re1 T01495 |
| 1209989 | Tepidanaerobacter acetatoxydans Re1 T02434 |
| 377629 | Teredinibacter turnerae T7901 T00952 |
| 386490 | Terribacillus goriensis T03216 |
| 926566 | Terriglobus roseus DSM 18391 T02140 |
| 401053 | Terriglobus saanensis SP1PR4 T01408 |
| 1332264 | Tessaracoccus aquimaris T04732 |
| 399497 | Tessaracoccus flavescens T04703 |
| 1610493 | Tessaracoccus flavus T04688 |
| 1909732 | Tessaracoccus sp T2 5 30 T04747 |
| 945021 | Tetragenococcus halophilus NBRC 12172 T01640 |
| 290335 | Tetragenococcus koreensis T05710 |
| 526944 | Tetragenococcus osmophilus T05711 |
| 312017 | Tetrahymena thermophila SB210 T01020 |
| 32264 | Tetranychus urticae T04901 |
| 99883 | Tetraodon nigroviridis T04346 |
| 1071380 | Tetrapisispora blattae CBS 6284 T02489 |
| 1071381 | Tetrapisispora phaffii CBS 4417 T02235 |
| 296543 | Thalassiosira pseudonana CCMP1335 T01078 |
| 2017482 | Thalassococcus sp S3 T05844 |
| 1298593 | Thalassolituus oleivorans MIL 1 T02529 |
| 1208320 | Thalassolituus oleivorans R6 15 T03056 |
| 1891279 | Thalassospira indica T05581 |
| 2048283 | Thalassospira marina T05224 |
| 1123366 | Thalassospira xiamenensis M 5 DSM 17429 T03639 |
| 44139 | Thauera aromatica K172 T05381 |
| 96773 | Thauera chlorobenzoica T04612 |
| 1134435 | Thauera humireducens T04211 |
| 2005884 | Thauera sp K11 T05106 |
| 164330 | Thauera sp MZ1T T00804 |
| 353154 | Theileria annulata strain Ankara T00253 |
| 1537102 | Theileria equi strain WA T02820 |
| 869250 | Theileria orientalis strain Shintoku T03460 |
| 333668 | Theileria parva strain Muguga T00254 |
| 3641 | Theobroma cacao T02994 |
| 1089553 | Thermacetogenium phaeum DSM 12270 T02276 |
| 644966 | Thermaerobacter marianensis DSM 12885 T01388 |
| 525903 | Thermanaerovibrio acidaminovorans DSM 6589 T01129 |
| 635013 | Thermincola potens JR T01226 |
| 2026 | Thermoactinomyces vulgaris T05864 |
| 509193 | Thermoanaerobacter brockii subsp finnii Ako 1 T01409 |
| 580331 | Thermoanaerobacter italicus Ab9 T01178 |
| 2325 | Thermoanaerobacter kivui T03397 |
| 583358 | Thermoanaerobacter mathranii subsp mathranii str A3 T01250 |
| 340099 | Thermoanaerobacter pseudethanolicus ATCC 33223 T00649 |
| 573062 | Thermoanaerobacter sp X513 T01317 |
| 399726 | Thermoanaerobacter sp X514 T00648 |
| 697303 | Thermoanaerobacter wiegelii Rt8 B1 T01607 |
| 1094508 | Thermoanaerobacterium saccharolyticum JW SL YS485 T02137 |
| 580327 | Thermoanaerobacterium thermosaccharolyticum DSM 571 T01299 |
| 698948 | Thermoanaerobacterium thermosaccharolyticum M0795 T02407 |
| 858215 | Thermoanaerobacterium xylanolyticum LX 11 T01505 |
| 717605 | Thermobacillus composti KWC4 T02394 |
| 525904 | Thermobaculum terrenum ATCC BAA 798 T01134 |
| 269800 | Thermobifida fusca YX T00265 |
| 469371 | Thermobispora bispora DSM 43833 T01236 |
| 1121335 | Thermoclostridium stercorarium subsp stercorarium DSM 8532 T02440 |
| 1121335 | Thermoclostridium stercorarium subsp stercorarium DSM 8532 T02527 |
| 391623 | Thermococcus barophilus MP T01377 |
| 54077 | Thermococcus barossii T05081 |
| 1293037 | Thermococcus celer Vu 13 JCM 8558 T05076 |
| 54262 | Thermococcus chitonophagus T04536 |
| 163003 | Thermococcus cleftensis T02169 |
| 1505907 | Thermococcus eurythermalis T03421 |
| 593117 | Thermococcus gammatolerans EJ3 T00920 |
| 71997 | Thermococcus gorgonarius T05047 |
| 1432656 | Thermococcus guaymasensis DSM 11113 T04184 |
| 69014 | Thermococcus kodakarensis KOD1 T00226 |
| 523849 | Thermococcus litoralis DSM 5473 T02800 |
| 195522 | Thermococcus nautili T03065 |
| 523850 | Thermococcus onnurineus NA1 T00789 |
| 71998 | Thermococcus pacificus T05192 |
| 582419 | Thermococcus paralvinellae T03005 |
| 53952 | Thermococcus peptonophilus T04728 |
| 1712654 | Thermococcus piezophilus T04941 |
| 49899 | Thermococcus profundus T05153 |
| 187880 | Thermococcus radiotolerans T05177 |
| 604354 | Thermococcus sibiricus MM 739 T00928 |
| 72803 | Thermococcus siculi T05103 |
| 1674923 | Thermococcus sp 2319x1 T04245 |
| 1042877 | Thermococcus sp 4557 T01588 |
| 2008440 | Thermococcus sp 5 4 T05088 |
| 246969 | Thermococcus sp AM4 T01638 |
| 122420 | Thermococcus sp P6 T05739 |
| 277988 | Thermococcus thioreducens T05104 |
| 638303 | Thermocrinis albus DSM 14484 T01179 |
| 75906 | Thermocrinis ruber DSM 12173 T03001 |
| 667014 | Thermodesulfatator indicus DSM 15286 T01546 |
| 289377 | Thermodesulfobacterium commune DSM 2178 T03212 |
| 795359 | Thermodesulfobacterium geofontis OPF15 T01547 |
| 1794699 | Thermodesulfobium acidiphilum T05396 |
| 747365 | Thermodesulfobium narugense DSM 14796 T01488 |
| 289376 | Thermodesulfovibrio yellowstonii DSM 11347 T00775 |
| 1365176 | Thermofilum adornatum T02802 |
| 697581 | Thermofilum adornatus 1505 T03526 |
| 368408 | Thermofilum pendens Hrk 5 T00446 |
| 1550241 | Thermofilum uzonense T03924 |
| 1184251 | Thermogladius calderae 1633 T02127 |
| 1331910 | Thermogutta terrifontis T05034 |
| 309801 | Thermomicrobium roseum DSM 5159 T00845 |
| 471852 | Thermomonospora curvata DSM 43183 T01127 |
| 273075 | Thermoplasma acidophilum DSM 1728 T00037 |
| 273116 | Thermoplasma volcanium GSS1 T00042 |
| 1054217 | Thermoplasmatales archaeon BRNA1 T02532 |
| 768679 | Thermoproteus tenax Kra 1 T01641 |
| 999630 | Thermoproteus uzoniensis 768 20 T01460 |
| 555079 | Thermosediminibacter oceani DSM 16646 T01289 |
| 484019 | Thermosipho africanus TCF52B T00797 |
| 391009 | Thermosipho melanesiensis BI429 T00550 |
| 1462747 | Thermosipho sp 1063 T04625 |
| 1437364 | Thermosipho sp 1070 T04909 |
| 633148 | Thermosphaera aggregans DSM 11486 T01237 |
| 1298851 | Thermosulfidibacter takaii ABI70S6 T04192 |
| 197221 | Thermosynechococcus elongatus BP 1 T00090 |
| 1394889 | Thermosynechococcus sp NK55a T02977 |
| 573729 | Thermothelomyces thermophilus ATCC 42464 T02231 |
| 578455 | Thermothielavioides terrestris NRRL 8126 T02228 |
| 243274 | Thermotoga maritima MSB8 T00022 |
| 243274 | Thermotoga maritima MSB8 T02663 |
| 243274 | Thermotoga maritima MSB8 T02976 |
| 243274 | Thermotoga maritima MSB8 T03902 |
| 2336 | Thermotoga maritima T03900 |
| 2336 | Thermotoga maritima T03901 |
| 590168 | Thermotoga naphthophila RKU 10 T01139 |
| 309803 | Thermotoga neapolitana DSM 4359 T00847 |
| 390874 | Thermotoga petrophila RKU 1 T00525 |
| 1157948 | Thermotoga sp 2812B T03494 |
| 1157947 | Thermotoga sp Cell2 T03495 |
| 126740 | Thermotoga sp RQ2 T00706 |
| 126738 | Thermotoga sp RQ7 T03669 |
| 648996 | Thermovibrio ammonificans HB 1 T01391 |
| 580340 | Thermovirga lienii DSM 17291 T01644 |
| 498848 | Thermus aquaticus Y51MC23 T04117 |
| 56956 | Thermus brockianus T04600 |
| 751945 | Thermus oshimai JL 2 T02346 |
| 456163 | Thermus parvatiensis T04274 |
| 743525 | Thermus scotoductus SA 01 T01414 |
| 1111069 | Thermus sp CCB US3 UF1 T01866 |
| 262724 | Thermus thermophilus HB27 T00170 |
| 300852 | Thermus thermophilus HB8 T00220 |
| 798128 | Thermus thermophilus JL 18 T02009 |
| 762633 | Thermus thermophilus SG0 5JP17 16 T01867 |
| 1255043 | Thioalkalivibrio nitratireducens DSM 14787 T02408 |
| 713585 | Thioalkalivibrio paradoxus ARh 1 T03590 |
| 396595 | Thioalkalivibrio sp K90mix T01174 |
| 396588 | Thioalkalivibrio sulfidiphilus HL EbGr7 T00840 |
| 106634 | Thioalkalivibrio versutus T03959 |
| 292415 | Thiobacillus denitrificans ATCC 25259 T00275 |
| 1915078 | Thioclava nitratireducens T04697 |
| 765911 | Thiocystis violascens DSM 198 T02138 |
| 765912 | Thioflavicoccus mobilis 8321 T02397 |
| 585455 | Thiohalobacter thiocyanaticus T05229 |
| 1076588 | Thiolapillus brandeum T03600 |
| 717772 | Thiomicrospira aerophila AL3 T03589 |
| 717773 | Thiomicrospira cyclica ALM1 T01517 |
| 1803865 | Thiomicrospira sp S5 T05796 |
| 426114 | Thiomonas arsenitoxydans T02347 |
| 75379 | Thiomonas intermedia K12 T01227 |
| 40754 | Thioploca ingrica T03616 |
| 2507161 | Tissierellia sp JN 28 T05805 |
| 1110502 | Tistrella mobilis KA081020 065 T02128 |
| 595494 | Tolumonas auensis DSM 9187 T00904 |
| 4950 | Torulaspora delbrueckii T02236 |
| 508771 | Toxoplasma gondii ME49 T01093 |
| 717944 | Trametes versicolor FP 101664 SS1 T04795 |
| 578456 | Tremella mesenterica DSM 1558 T03139 |
| 545695 | Treponema azotonutricium ZAS 9 T01506 |
| 906968 | Treponema brennaborense DSM 12168 T01489 |
| 744872 | Treponema caldarium DSM 7334 T01573 |
| 243275 | Treponema denticola ATCC 35405 T00157 |
| 1155775 | Treponema pallidum str Fribourg Blanc T02651 |
| 491081 | Treponema pallidum subsp pallidum DAL 1 T01748 |
| 455434 | Treponema pallidum subsp pallidum SS14 T00716 |
| 666714 | Treponema pallidum subsp pallidum str Chicago T01868 |
| 686990 | Treponema pallidum subsp pallidum str Mexico A T02273 |
| 243276 | Treponema pallidum subsp pallidum str Nichols T00016 |
| 243276 | Treponema pallidum subsp pallidum str Nichols T02709 |
| 1095955 | Treponema pallidum subsp pallidum str Sea 81 4 T03185 |
| 491079 | Treponema pallidum subsp pertenue str CDC2 T01749 |
| 491080 | Treponema pallidum subsp pertenue str Gauthier T01750 |
| 491078 | Treponema pallidum subsp pertenue str SamoaD T01751 |
| 545776 | Treponema paraluiscuniculi Cuniculi A T01555 |
| 1291379 | Treponema pedis str T A4 T02803 |
| 545694 | Treponema primitia ZAS 2 T01507 |
| 221027 | Treponema putidum T03278 |
| 1539298 | Treponema sp OMZ 838 T03454 |
| 869209 | Treponema succinifaciens DSM 2489 T01461 |
| 7070 | Tribolium castaneum T01046 |
| 127582 | Trichechus manatus latirostris T05135 |
| 6334 | Trichinella spiralis T01609 |
| 431241 | Trichoderma reesei QM6a T02991 |
| 203124 | Trichodesmium erythraeum IMS101 T00387 |
| 412133 | Trichomonas vaginalis G3 T01022 |
| 663331 | Trichophyton benhamiae CBS 112371 T01611 |
| 663202 | Trichophyton verrucosum HKI 0517 T01615 |
| 10228 | Trichoplax adhaerens T01069 |
| 7111 | Trichoplusia ni T05757 |
| 240292 | Trichormus variabilis ATCC 29413 T00277 |
| 203267 | Tropheryma whipplei str Twist T00116 |
| 218496 | Tropheryma whipplei TW08 27 T00117 |
| 649638 | Truepera radiovictrix DSM 17093 T01254 |
| 1661 | Trueperella pyogenes T03070 |
| 1435056 | Trueperella pyogenes TP8 T03579 |
| 185431 | Trypanosoma brucei brucei TREU927 T01013 |
| 353153 | Trypanosoma cruzi strain CL Brener T01012 |
| 521096 | Tsukamurella paurometabola DSM 20162 T01228 |
| 57704 | Tsukamurella tyrosinosolvens T05267 |
| 656061 | Tuber melanosporum Mel28 T01282 |
| 1214604 | Tumebacillus algifaecis T04994 |
| 1903704 | Tumebacillus avium T04874 |
| 246437 | Tupaia chinensis T02978 |
| 1712675 | Turicibacter sp H121 T04254 |
| 869212 | Turneriella parva DSM 21527 T02157 |
| 336963 | Uncinocarpus reesii 1704 T01132 |
| 310581 | Uncultured Sphingopyxis sp T05531 |
| 1408204 | Uncultured Termite group 1 bacterium phylotype Rs D17 T00755 |
| 401471 | Undibacterium parvum T05754 |
| 505682 | Ureaplasma parvum serovar 3 str ATCC 27815 T00680 |
| 273119 | Ureaplasma parvum serovar 3 str ATCC 700970 T00040 |
| 38504 | Ureaplasma parvum serovar 3 T03673 |
| 565575 | Ureaplasma urealyticum serovar 10 str ATCC 33699 T00781 |
| 1850246 | Urechidicola croceus T04497 |
| 29073 | Ursus maritimus T03271 |
| 237631 | Ustilago maydis 521 T01021 |
| 633807 | Vagococcus penaei T04715 |
| 519472 | Vagococcus teuberi T04578 |
| 1930593 | Vampirococcus sp LiM T05804 |
| 436907 | Vanderwaltozyma polyspora DSM 70294 T01049 |
| 1333996 | Variibacter gotjawalensis T05320 |
| 436515 | Variovorax boronicumulans T05068 |
| 1246301 | Variovorax paradoxus B4 T02835 |
| 595537 | Variovorax paradoxus EPS T01392 |
| 543728 | Variovorax paradoxus S110 T00916 |
| 1795631 | Variovorax sp PAMC 28711 T04325 |
| 2126319 | Variovorax sp PMC12 T05380 |
| 39777 | Veillonella atypica T04798 |
| 39778 | Veillonella dispar T05848 |
| 479436 | Veillonella parvula DSM 2008 T01130 |
| 248315 | Veillonella rodentium T05216 |
| 391735 | Verminephrobacter eiseniae EF01 2 T00460 |
| 1637999 | Verrucomicrobia bacterium IMCC26134 T03830 |
| 2576891 | Verrucomicrobia bacterium T05426 |
| 263358 | Verrucosispora maris AB 18 032 T01471 |
| 526221 | Verticillium alfalfae VaMs 102 T02227 |
| 498257 | Verticillium dahliae VdLs 17 T03450 |
| 1074311 | Vibrio alfacsensis T05841 |
| 1219076 | Vibrio alginolyticus NBRC 15630 ATCC 17749 T02840 |
| 882102 | Vibrio anguillarum 775 T01536 |
| 882944 | Vibrio anguillarum M3 T02823 |
| 55601 | Vibrio anguillarum T03657 |
| 150340 | Vibrio antiquarius T01107 |
| 553239 | Vibrio breoganii T04649 |
| 2902295 | Vibrio campbellii ATCC BAA 1116 T00589 |
| 2902295 | Vibrio campbellii ATCC BAA 1116 T02837 |
| 1134456 | Vibrio cholerae IEC224 T01766 |
| 935297 | Vibrio cholerae LMA3984 4 T01871 |
| 579112 | Vibrio cholerae M66 2 T00891 |
| 593588 | Vibrio cholerae MJ 1236 T00903 |
| 1420885 | Vibrio cholerae MS6 T03666 |
| 243277 | Vibrio cholerae O1 biovar El Tor str N16961 T00034 |
| 686 | Vibrio cholerae O1 biovar El Tor T03827 |
| 914149 | Vibrio cholerae O1 str 2010EL 1786 T01650 |
| 345073 | Vibrio cholerae O395 T00520 |
| 345073 | Vibrio cholerae O395 T02099 |
| 666 | Vibrio cholerae T03389 |
| 666 | Vibrio cholerae T03828 |
| 666 | Vibrio cholerae T03829 |
| 190893 | Vibrio coralliilyticus T03444 |
| 190893 | Vibrio coralliilyticus T03497 |
| 50719 | Vibrio diabolicus T05332 |
| 676 | Vibrio fluvialis T04379 |
| 903510 | Vibrio furnissii NCTC 11218 T01680 |
| 687 | Vibrio gazogenes T04933 |
| 669 | Vibrio harveyi T04380 |
| 689 | Vibrio mediterranei T04936 |
| 674 | Vibrio mimicus T04381 |
| 1219067 | Vibrio natriegens NBRC 15636 ATCC 14048 DSM 759 T04636 |
| 28173 | Vibrio nigripulchritudo T02871 |
| 696485 | Vibrio owensii T04844 |
| 1211705 | Vibrio parahaemolyticus BB22OP T02409 |
| 1338032 | Vibrio parahaemolyticus O1 K33 str CDC K4557 T02760 |
| 1338034 | Vibrio parahaemolyticus O1 Kuk str FDA R31 T02761 |
| 223926 | Vibrio parahaemolyticus RIMD 2210633 T00120 |
| 1429044 | Vibrio parahaemolyticus UCM V493 T03116 |
| 2025808 | Vibrio qinghaiensis T05259 |
| 190895 | Vibrio rotiferianus T05115 |
| 45658 | Vibrio scophthalmi T04650 |
| 1116375 | Vibrio sp EJY3 T01683 |
| 1671868 | Vibrio tapetis subsp tapetis T05424 |
| 693153 | Vibrio tasmaniensis LGP32 T00812 |
| 1051646 | Vibrio tubiashii ATCC 19109 T03445 |
| 216895 | Vibrio vulnificus CMCP6 T00108 |
| 914127 | Vibrio vulnificus MO6 24 O T01415 |
| 672 | Vibrio vulnificus T03294 |
| 196600 | Vibrio vulnificus YJ016 T00150 |
| 2094242 | Victivallales bacterium CCUG 44730 T05502 |
| 3914 | Vigna angularis T04660 |
| 3916 | Vigna radiata var radiata T04126 |
| 302167 | Virgibacillus dokdonensis T05261 |
| 1482 | Virgibacillus halodenitrificans T04548 |
| 163877 | Virgibacillus necropolis T04976 |
| 2017483 | Virgibacillus phasianinus T04951 |
| 1911587 | Virgibacillus sp 6R T04596 |
| 403957 | Virgibacillus sp SK37 T03219 |
| 29760 | Vitis vinifera T01084 |
| 63 | Vitreoscilla filiformis T04960 |
| 96942 | Vitreoscilla sp C1 T05336 |
| 3068 | Volvox carteri f nagariensis T01330 |
| 572478 | Vulcanisaeta distributa DSM 14429 T01313 |
| 985053 | Vulcanisaeta moutnovskia 768 28 T01440 |
| 1391653 | Vulgatibacter incomptus T04035 |
| 716544 | Waddlia chondrophila WSU 86 1044 T01255 |
| 1299270 | Wallemia ichthyophaga EXF 994 T03457 |
| 671144 | Wallemia mellicola CBS 633 66 T03142 |
| 865938 | Weeksella virosa DSM 16922 T01441 |
| 887702 | Weissella ceti T03228 |
| 759620 | Weissella ceti T03280 |
| 759620 | Weissella ceti T03295 |
| 137591 | Weissella cibaria T04106 |
| 1631871 | Weissella jogaejeotgali T04632 |
| 1045854 | Weissella koreensis KACC 15510 T01577 |
| 1249 | Weissella paramesenteroides T05120 |
| 1790137 | Wenyingzhuangia fucanilytica T04444 |
| 1579979 | Wenzhouxiangella marina T04011 |
| 36870 | Wigglesworthia glossinidia endosymbiont of Glossina brevipalpis T00101 |
| 1142511 | Wigglesworthia glossinidia endosymbiont of Glossina morsitans morsitans T01747 |
| 1936080 | Winogradskyella sp J14 2 T04714 |
| 754409 | Winogradskyella sp PG 2 T03603 |
| 1548547 | Woeseia oceani T04427 |
| 246273 | Wolbachia endosymbiont of Cimex lectularius T03628 |
| 570417 | Wolbachia endosymbiont of Culex quinquefasciatus Pel T00719 |
| 163164 | Wolbachia endosymbiont of Drosophila melanogaster T00160 |
| 1236909 | Wolbachia endosymbiont of Drosophila simulans wHa T02640 |
| 1236908 | Wolbachia endosymbiont of Drosophila simulans wNo T02641 |
| 169402 | Wolbachia endosymbiont of Folsomia candida T05726 |
| 100901 | Wolbachia endosymbiont of Onchocerca ochengi T02212 |
| 292805 | Wolbachia endosymbiont strain TRS of Brugia malayi T00234 |
| 1116230 | Wolbachia pipientis wAlbB T05842 |
| 66084 | Wolbachia sp wRi T00866 |
| 273121 | Wolinella succinogenes DSM 1740 T00146 |
| 78245 | Xanthobacter autotrophicus Py2 T00581 |
| 380358 | Xanthomonas albilineans GPE PC73 T01160 |
| 981368 | Xanthomonas axonopodis pv citrumelo F1 T01616 |
| 1304892 | Xanthomonas axonopodis Xac29 1 T02500 |
| 314565 | Xanthomonas campestris pv campestris str 8004 T00247 |
| 190485 | Xanthomonas campestris pv campestris str ATCC 33913 T00083 |
| 340 | Xanthomonas campestris pv campestris T00759 |
| 990315 | Xanthomonas campestris pv raphani 756C T01872 |
| 316273 | Xanthomonas campestris pv vesicatoria str 85 10 T00288 |
| 190486 | Xanthomonas citri pv citri str 306 T00084 |
| 611301 | Xanthomonas citri pv citri T03733 |
| 611301 | Xanthomonas citri pv citri T03734 |
| 611301 | Xanthomonas citri pv citri T03735 |
| 611301 | Xanthomonas citri pv citri T03779 |
| 611301 | Xanthomonas citri pv citri T03780 |
| 611301 | Xanthomonas citri pv citri T03781 |
| 366649 | Xanthomonas citri pv fuscans T03094 |
| 1308541 | Xanthomonas citri subsp citri A306 T03584 |
| 1137651 | Xanthomonas citri subsp citri Aw12879 T02519 |
| 1308548 | Xanthomonas citri subsp citri UI6 T03894 |
| 48664 | Xanthomonas fragariae T04667 |
| 2754056 | Xanthomonas gardneri T04668 |
| 56454 | Xanthomonas hortorum T05082 |
| 291331 | Xanthomonas oryzae pv oryzae KACC 10331 T00235 |
| 342109 | Xanthomonas oryzae pv oryzae MAFF 311018 T00360 |
| 1458476 | Xanthomonas oryzae pv oryzae PXO86 T03765 |
| 360094 | Xanthomonas oryzae pv oryzae PXO99A T00763 |
| 383407 | Xanthomonas oryzae pv oryzicola BLS256 T01873 |
| 129394 | Xanthomonas oryzae pv oryzicola T03868 |
| 442694 | Xanthomonas perforans T04778 |
| 317013 | Xanthomonas phaseoli pv phaseoli T05156 |
| 56458 | Xanthomonas sacchari T03566 |
| 487909 | Xanthomonas translucens pv undulosa T03961 |
| 325776 | Xanthomonas vasicola pv vasculorum T05388 |
| 925775 | Xanthomonas vesicatoria ATCC 35937 T04741 |
| 8355 | Xenopus laevis T01010 |
| 8364 | Xenopus tropicalis T01011 |
| 406818 | Xenorhabdus bovienii SS 2004 T01186 |
| 40576 | Xenorhabdus bovienii T03845 |
| 351671 | Xenorhabdus doucetiae T03784 |
| 351679 | Xenorhabdus hominickii T04538 |
| 1437823 | Xenorhabdus nematophila AN6 1 T03752 |
| 406817 | Xenorhabdus nematophila ATCC 19061 T01274 |
| 1354304 | Xenorhabdus poinarii G6 T03805 |
| 8083 | Xiphophorus maculatus T02923 |
| 2509457 | Xylanimicrobium sp FW10M 9 T05815 |
| 2509459 | Xylanimonas allomyrinae T05814 |
| 446471 | Xylanimonas cellulosilytica DSM 15894 T01135 |
| 160492 | Xylella fastidiosa 9a5c T00033 |
| 405440 | Xylella fastidiosa M12 T00677 |
| 405441 | Xylella fastidiosa M23 T00692 |
| 1401256 | Xylella fastidiosa MUL0034 T03317 |
| 788929 | Xylella fastidiosa subsp fastidiosa GB514 T01979 |
| 155920 | Xylella fastidiosa subsp sandyi Ann 1 T03318 |
| 2371 | Xylella fastidiosa T04925 |
| 183190 | Xylella fastidiosa Temecula1 T00113 |
| 1444770 | Xylella taiwanensis T05568 |
| 2315449 | Yamadazyma tenuis ATCC 10573 T03099 |
| 311180 | Yangia pacifica T05431 |
| 1792508 | Yangia sp CCB MM3 T04443 |
| 284591 | Yarrowia lipolytica CLIB122 T01033 |
| 1453495 | Yersinia aldovae 670 83 T03605 |
| 263819 | Yersinia aleksiciae T03994 |
| 2339259 | Yersinia enterocolitica LC20 T03179 |
| 1262462 | Yersinia enterocolitica str YE53 03 T03739 |
| 393305 | Yersinia enterocolitica subsp enterocolitica 8081 T00469 |
| 994476 | Yersinia enterocolitica subsp palearctica 105 5R T01480 |
| 930944 | Yersinia enterocolitica subsp palearctica Y11 T02010 |
| 630 | Yersinia enterocolitica T03643 |
| 630 | Yersinia enterocolitica T03644 |
| 630 | Yersinia enterocolitica T03890 |
| 1454377 | Yersinia frederiksenii Y225 T03606 |
| 631 | Yersinia intermedia T03608 |
| 1604335 | Yersinia kristensenii T03609 |
| 419257 | Yersinia massiliensis T05402 |
| 1035377 | Yersinia pestis A1122 T01831 |
| 349746 | Yersinia pestis Angola T00635 |
| 360102 | Yersinia pestis Antiqua T00369 |
| 547048 | Yersinia pestis biovar Medievalis str Harbin 35 T01874 |
| 229193 | Yersinia pestis biovar Microtus str 91001 T00165 |
| 214092 | Yersinia pestis CO92 T00063 |
| 637382 | Yersinia pestis D106004 T01832 |
| 637385 | Yersinia pestis D182038 T01833 |
| 187410 | Yersinia pestis KIM10 T00092 |
| 377628 | Yersinia pestis Nepal516 T00370 |
| 386656 | Yersinia pestis Pestoides F T00505 |
| 632 | Yersinia pestis T03716 |
| 632 | Yersinia pestis T03717 |
| 632 | Yersinia pestis T03718 |
| 632 | Yersinia pestis T03719 |
| 637386 | Yersinia pestis Z176003 T01210 |
| 349747 | Yersinia pseudotuberculosis IP 31758 T00570 |
| 273123 | Yersinia pseudotuberculosis IP 32953 T00195 |
| 273123 | Yersinia pseudotuberculosis IP 32953 T03832 |
| 502801 | Yersinia pseudotuberculosis PB1 T00744 |
| 748672 | Yersinia pseudotuberculosis str PA3606 T03846 |
| 633 | Yersinia pseudotuberculosis T03307 |
| 633 | Yersinia pseudotuberculosis T03677 |
| 633 | Yersinia pseudotuberculosis T03678 |
| 633 | Yersinia pseudotuberculosis T03679 |
| 502800 | Yersinia pseudotuberculosis YPIII T00665 |
| 29485 | Yersinia rohdei T03610 |
| 29486 | Yersinia ruckeri T03611 |
| 29486 | Yersinia ruckeri T03887 |
| 367190 | Yersinia similis T03057 |
| 245188 | Yoonia vestfoldensis T04854 |
| 4577 | Zea mays T01088 |
| 1470434 | Zhongshania aliphaticivorans T04315 |
| 326968 | Ziziphus jujuba T04794 |
| 347534 | Zobellella denitrificans T05100 |
| 63186 | Zobellia galactanivorans T01586 |
| 2080469 | Zoogloeaceae bacteirum Par f 2 T05440 |
| 136037 | Zootermopsis nevadensis T05136 |
| 655815 | Zunongwangia profunda SM A87 T01216 |
| 559307 | Zygosaccharomyces rouxii CBS 732 T01118 |
| 33074 | Zymobacter palmae T05646 |
| 555217 | Zymomonas mobilis subsp mobilis ATCC 10988 T01875 |
| 627344 | Zymomonas mobilis subsp mobilis ATCC 29191 T02187 |
| 622759 | Zymomonas mobilis subsp mobilis NCIMB 11163 T01110 |
| 1194424 | Zymomonas mobilis subsp mobilis NRRL B 12526 T03160 |
| 627343 | Zymomonas mobilis subsp mobilis str CP4 NRRL B 14023 T02932 |
| 627343 | Zymomonas mobilis subsp mobilis str CP4 NRRL B 14023 T03385 |
| 264203 | Zymomonas mobilis subsp mobilis ZM4 ATCC 31821 T00221 |
| 579138 | Zymomonas mobilis subsp pomaceae ATCC 29192 T01556 |
| 336722 | Zymoseptoria tritici IPO323 T02285 |
